# A versatile toolbox for knock-in gene targeting based on the Multisite Gateway technology

**DOI:** 10.1371/journal.pone.0221164

**Published:** 2019-08-27

**Authors:** Sho Yoshimatsu, Takefumi Sone, Mayutaka Nakajima, Tsukika Sato, Ryotaro Okochi, Mitsuru Ishikawa, Mari Nakamura, Erika Sasaki, Seiji Shiozawa, Hideyuki Okano

**Affiliations:** 1 Department of Physiology, School of Medicine, Keio University, Shinjuku-ku, Tokyo, Japan; 2 Laboratory for Proteolytic Neuroscience, RIKEN Center for Brain Science, Wako City, Saitama, Japan; 3 Central Institute for Experimental Animals, Kawasaki City, Kanagawa, Japan; 4 Laboratory for Marmoset Neural Architecture, RIKEN Center for Brain Science, Wako City, Saitama, Japan; Chinese Academy of Sciences, CHINA

## Abstract

Knock-in (KI) gene targeting can be employed for a wide range of applications in stem cell research. However, vectors for KI require multiple complicated processes for construction, including multiple times of digestion/ligation steps and extensive restriction mapping, which has imposed limitations for the robust applicability of KI gene targeting. To circumvent this issue, here we introduce versatile and systematic methods for generating KI vectors by molecular cloning. In this approach, we employed the Multisite Gateway technology, an efficient *in vitro* DNA recombination system using proprietary sequences and enzymes. KI vector construction exploiting these methods requires only efficient steps, such as PCR and recombination, enabling robust KI gene targeting. We show that combinatorial usage of the KI vectors generated using this method and site-specific nucleases enabled the precise integration of fluorescent protein genes in multiple loci of human and common marmoset (marmoset; *Callithrix jacchus*) pluripotent stem cells. The methods described here will facilitate the usage of KI technology and ultimately help to accelerate stem cell research.

## Introduction

Knock-in (KI) gene targeting, a gene engineering method mediated by homologous recombination (HR), can be employed for a wide range of applications in stem cell research. Conventional KI methods intended for use in mammalian pluripotent stem cells (PSCs) required long homology arms of over 5−10 kb [1, 2]. Recently, to improve KI efficiency, site specific-endonucleases (SNs), such as zinc finger nucleases (ZFNs), transcription activator-like effector nucleases (TALENs), and clustered regularly interspaced short palindromic repeats/CRISPR associated protein 9 (CRISPR/Cas9) have been utilized and validated in mammalian PSCs [3–5]. Although only short homology arms of 0.5−1 kb are required for KI with SNs, multiple complicated processes, including multiple digestion/ligation steps and extensive restriction mapping, must be taken to generate KI vectors harboring two homology arms of the 5' side and 3' side, and a drug-resistance cassette flanked by the arms to concentrate the homologous recombinants [3, 4, 6]. So far, these time-consuming and cumbersome steps have imposed limitations for the robust applicability of KI gene targeting.

To address this issue, here we introduce versatile and systematic methods for the generation of KI vectors using the Multisite Gateway technology. The Gateway technology, originating from a DNA recombination system of the Enterobacteria phage λ, utilizes proprietary sequences (*att* sequences) and recombination enzymes (BP clonase and LR clonase) for specific recombination [7]. Distinct from commonly used restriction enzymes that recognize 6−8 bp DNA sequences, the recombination enzymes recognize specific sequences (a core 21 bp sequence in each *att* site), which renders it unnecessary to perform extensive restriction mapping for generating long or complicated vectors. This system involves two of the following reactions for specific recombination between *att* sites: (1) the BP reaction (*att*B × *att*P → *att*L + *att*R), which is mediated by the integrase (Int) and integration host factor (IHF) proteins (so called BP clonase), and (2) the LR reaction (*att*L × *att*R → *att*B + *att*P), which is mediated by the Int, IHF and excisionase (Xis) enzymes (so called LR clonase). In addition, because there are six mutant sequences available for each *att* sequence (*att*B1−6, *att*P1−6, *att*L1−6 and *att*R1−6; each of these *att*Bx or *att*Lx (x: 1−6) is specifically recombined with the corresponding *att*Px or *att*Rx by BP or LR reaction, respectively), multiple recombination cloning can be performed in a specific manner using this system (termed Multisite Gateway technology), the utility of which has been validated in mammalian and plant cells [8, 9].

In the current study, we employ our methods to generate KI vectors based on the Multisite Gateway technology. Although similar attempts have been conducted for mammalian cells previously [6, 10], we developed novel methods for generating KI vectors for (1) the introduction and correction of mutations, and (2) the integration of fluorescent/luminescent reporter gene(s) in specific loci, leaving only minimal residual sequence in the genome following KI and cassette excision. Furthermore, we demonstrate that the latter can be used in both human and common marmoset (marmoset; *Callithrix jacchus*) PSCs.

## Materials and methods

### Cell culture and transfection

A human induced pluripotent stem cell (hiPSC) line 201B7 was used in the current study, which was previously established [11] and kindly provided by Shinya Yamanaka (Kyoto University). The hiPSCs were cultured on Laminin-511 (Nippi) in StemFit AK02N medium (AK02N; Ajinomoto). For passaging, the hiPSCs were pre-treated with 10 μM ROCK inhibitor, Y-27632 (Merck) in AK02N at 37°C for one hour. The cells were next dissociated into single cells by TrypLE Select (Thermo Fisher), and were plated to Laminin-511-coated plates in AK02N supplemented with 10 μM Y-27632. Transfection and positive/negative selection of hiPSCs were performed as described previously [12, 13]. In brief, hiPSCs were dissociated into single cells by TrypLE Select, and 10 μg of each KI vector with 5 μg of each Cas9-gRNA vector (for KI) or 10 μg of pCAGS-PBx (for cassette excision) was transfected into 1 × 10^6^ hiPSCs by electroporation using a NEPA21 electroporator (NEPA Gene) at an optimized poring pulse condition of 125 mV for 5 ms. The cells were immediately plated onto new Laminin-511-coated plates in AK02N supplemented with 10 μM Y-27632 (Day 0). On day 1, the medium was changed to AK02N without Y-27632. From day 3, cells were treated with 1 μg/ml puromycin (Sigma) or 10 μM ganciclovir (Nacalai Tesque) with 10 μM Y-27632 for positive or negative selection, respectively. From day 5, Y-27632 was removed from the medium.

A marmoset embryonic stem cell (cjESC) line, CMES40 was used in the current study, which was previously established [14]. The cjESCs were cultured on 30 Gy-irradiated mouse embryonic fibroblasts (MEFs) in ES medium (ESM). ESM consisted of 1× Knockout Dulbecco's modified Eagle's medium (Thermo Fisher) supplemented with 20% Knockout Serum Replacement (KSR; Thermo Fisher), 0.1 mM MEM Non-Essential Amino Acids Solution (NEAA; Sigma), 1 mM L-glutamine (L-glu; Thermo Fisher), 0.2 mM β-mercaptoethanol (2ME; Thermo Fisher) and 10 ng/ml human recombinant basic fibroblast growth factor (Thermo Fisher). For passaging, the cjESCs were pre-treated with 10 μM Y-27632 in ESM at 37°C for one hour. The cells were then dissociated by 0.25% trypsin-ethylenediaminetetraacetic acid (Nacalai Tesque), and mechanically separated from MEFs. After removing MEFs, cjESCs were plated onto new MEFs. Irradiated MEFs were prepared on gelatin-coated 100-mm dishes or 6-well plates, at a density of 2.5 × 10^6^ cells / 100-mm dish or 6-well plate prior to seeding cjESCs. Transfection and positive/negative selection of cjESCs were performed as described previously [15, 16]. In brief, Lipofectamine LTX with PLUS reagent (Thermo Fisher) was used for transfection according to the manufacturer’s instructions. For selection, 25 μg/ml hygromycin (Sigma), 1 μg/ml puromycin, 50 μg/ml G418 or 10 μM ganciclovir was used.

### Differentiation of PSCs

Spontaneous differentiation of cjESCs was performed by embryoid body (EB) formation, as previously described [14, 16].

Hippocampal differentiation of hiPSCs was performed as previously described [17] with slight modifications. In brief, sub-confluent hiPSCs were dissociated into single cells using TrypLE Select, and transferred into conical V-bottom-wells of low-cell-adhesion 96-well plates (Sumitomo Bakelite) at a density of 10,000 cells per well in 100 μl differentiation medium (Day 0). The differentiation medium consisted of Glasgow's MEM (Thermo Fisher) supplemented with 20% KSR, 0.1 mM NEAA, 1 mM L-glu, 1 mM Sodium pyruvate (Sigma), 0.1 mM 2ME, 3 μM IWR1e (Calbiochem), 5 μM SB431542 (Tocris) and 20 μM Y-27632. The medium was changed every few days until day 18. On day 18, the EBs were collected, and transferred to wells of Ultra-low attachment surface 6-well plates (Corning) in induction medium. The induction medium consisted of DMEM/F-12 (Sigma), 1% N2 supplement (Thermo Fisher), 0.1 mM NEAA, 1 mM L-glu supplemented with 3 μM CHIR99021 (Cayman), 8 ng/ml human recombinant BMP4 (R&D Systems) and 10% FBS. On day 30, the EBs were briefly dissociated by pipetting, and plated onto each well of 8-well chamber slides (Iwaki) which were coated with poly-l-ornithine/fibronectin (Sigma) in neuronal medium consisting of 1× Neurobasal medium (Thermo Fisher) supplemented with 10% FBS, 1 mM L-glu, 1% N2 supplement and 10 ng/ml recombinant human BDNF (Thermo Fisher).

### FACS analysis

Before FACS analysis, cells were incubated in Accutase (Nacalai Tesque) at 37°C for an hour for single-cell dissociation. Following dissociation, the cells were suspended in FACS buffer consisting of 1% inactivated fetal bovine serum (FBS), 5 mM EDTA (Sigma) and 10 μM Y-27632 in 1× PBS buffer, supplemented with 0.1% Propidium iodide staining solution (Sigma) for removing dead cells. FACS analysis was performed using the FACSAria III cell sorter (BD) with the FACSuite software (BD) following the manufacturer’s instructions.

### Immunochemistry

For immunochemical analysis, cells were fixed with 4% paraformaldehyde or 100% ethanol for 15–30 min at room temperature. After incubating with blocking buffer (PBS containing 0.05% Tween 20 (Sigma) and 10% goat or donkey serum) for 30–60 min at room temperature, the cells were incubated with primary antibodies at 4°C overnight. After incubation with primary antibodies, the cells were washed with PBS three times, and incubated with Alexa 488, 555 and 647-conjugated secondary antibodies (Thermo Fisher) and 10 mg/ml Hoechst 33258 (Sigma) for one hour at room temperature. Primary antibodies used in the current study are as follows: anti-NANOG (1:500; 1E6C4; Cell Signaling), SSEA4 (1:500; MAB4304; Merck), TRA-1-60 (1:500; MAB4360; Merck), βIII-tubulin (1:400; 2G10; Abcam), αSMA (1:1000; 1A4; Sigma), SOX17 (1:200; AF1924; R&D Systems), HNF3β/FOXA2 (1:500; D5606; Cell Signaling), RFP (1:500, ab62341, Abcam) and VGLUT2 (1:500, ab79157, Abcam).

### Vector construction

**Fig 1:**
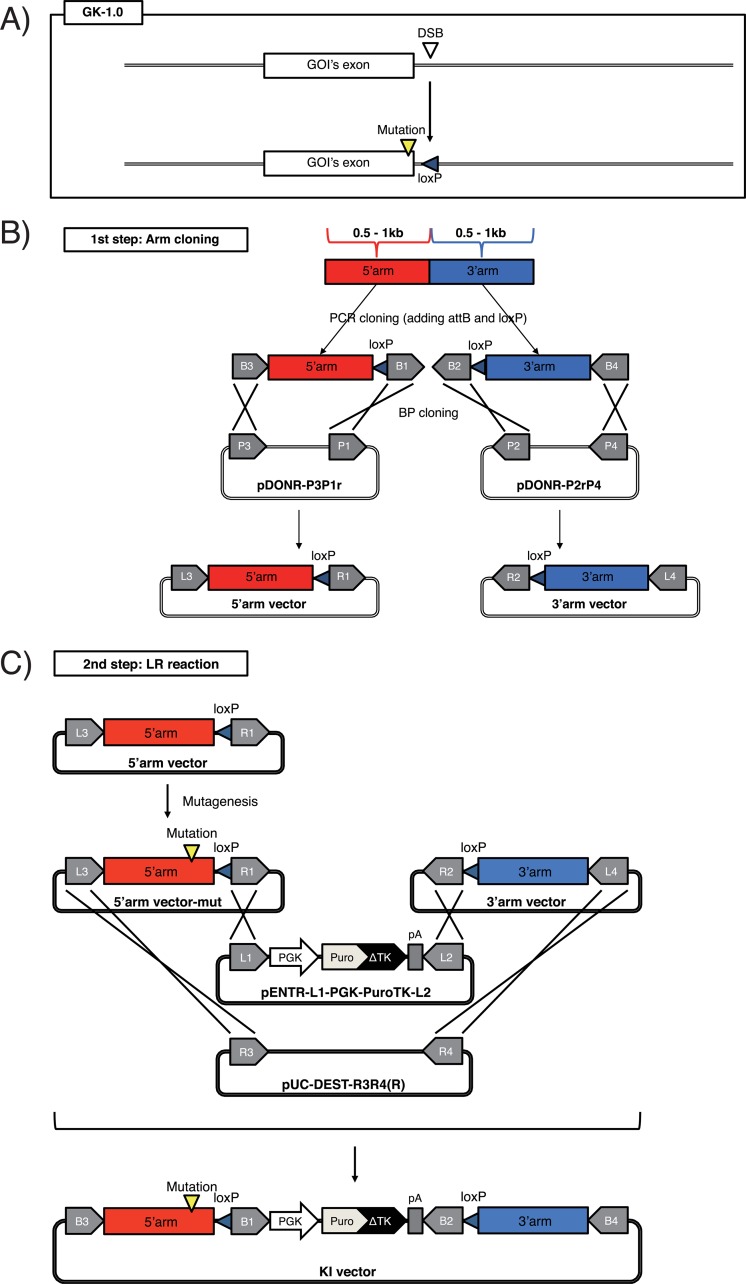
Schematic of the GKI-1.0 method. (A) Schematic of the GKI-1.0 method. DSB, double strand break; GOI, gene of interest. (B) The 1st step of GKI-1.0. A 5'-side homology arm (red box) and 3'-side homology arm (blue box) are subcloned into pDONR-P3P1r and pDONR-P2rP4 by PCR and BP cloning, respectively. X-marks indicate recombination that occurs between each corresponding *att*B and *att*P during the BP reaction. (C) The 2nd step of GKI-1.0. X-marks indicate recombination that occurs between each corresponding *att*L and *att*R during the LR reaction. *Chloramphenicol resistance gene (CmR)* and *ccdB* gene are located between *att*R3 and *att*R4 in pUC-DEST-R3R4(R) (not described) for the positive/negative selection of transformants harboring the vector itself or the desired KI vector. PGK, murine *phosphoglycerate Kinase 1 (Pgk-1)* promoter; PuroTK, a bifunctional fusion protein gene between *puromycin-N-acetyltransferase gene (Puro)* and a N-terminal shortened version of herpes simplex virus type 1 *thymidine kinase (ΔTK)* [25]; pA, polyadenylation signal sequence. The entire sequences of pDONR-P3P1r, pDONR-P2rP4 and pUC-DEST-R3R4(R) are attached in the S1 sequence.

**Fig 2:**
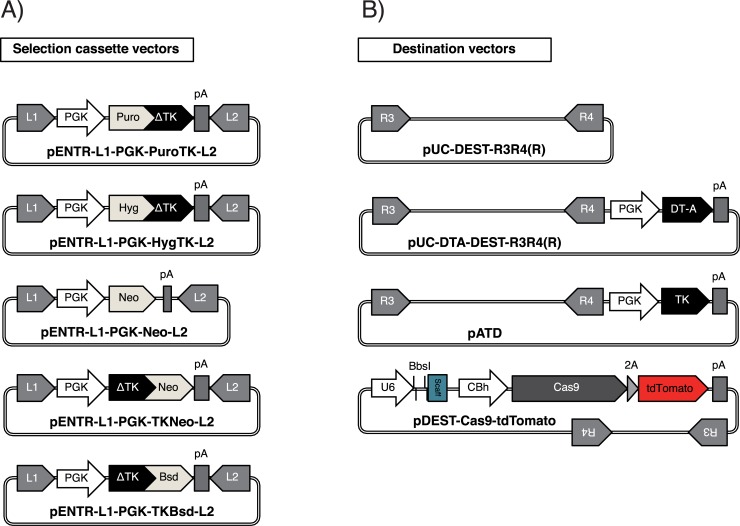
Module vectors for GKI methods. (A) Selection cassette vectors intended for use at the 2nd step of the GKI methods. (B) Destination vectors intended for use at the 2nd step of the GKI methods. The *CmR* and *ccdB* gene are located between *att*R3 and *att*R4 in each destination vector (not described) for the positive/negative selection of transformants harboring the vector itself or the desired KI vector. DT-A, *diphtheria toxin A*; TK, Herpes Simplex Virus-1 *Thymidine Kinase*; U6, mammalian U6 type III RNA polymerase III promoter; BbsI, a sgRNA cloning site (mediated by *Bbs*I digestion); Scaff, scaffold sequence of sgRNA followed by TTTTTT (poly-T terminal signal of RNA polymerase III); CBh, cytomegalovirus enhancer fused to the chicken beta-actin promoter and hybrid intron; Cas9, *spCas9*; 2A, *Thosea asigna* virus 2A peptide sequence (T2A). Whole sequences for all vectors described are provided in the S1 sequence.

**Fig 3:**
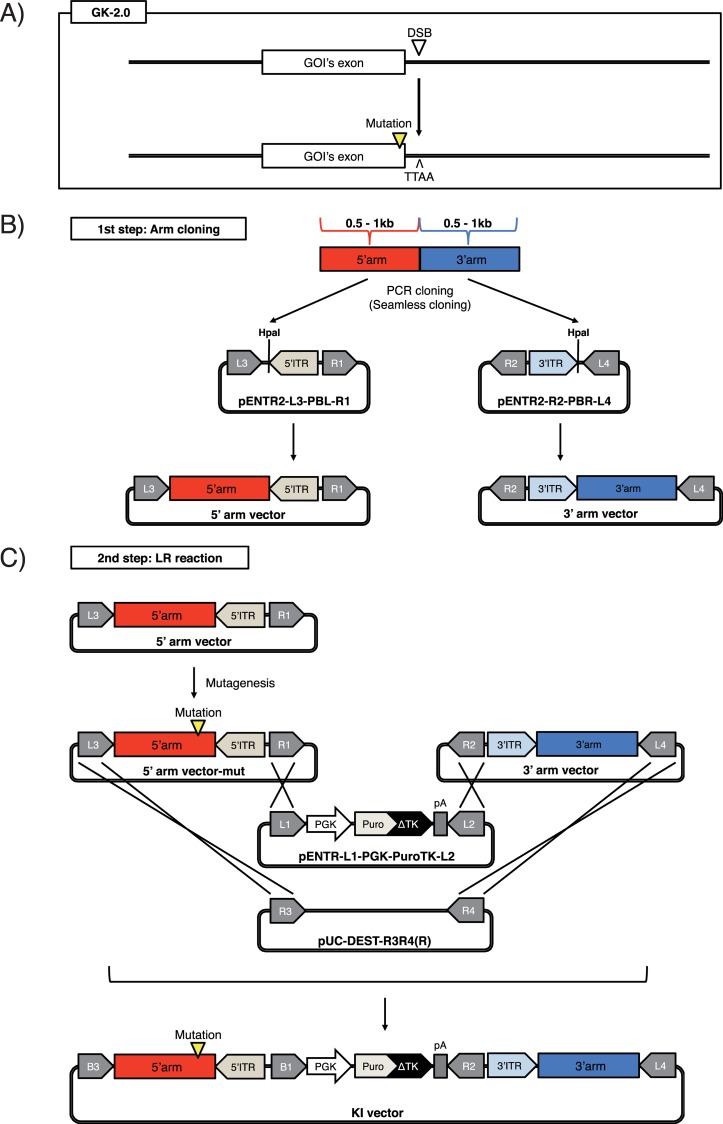
Schematic of the GKI-2.0 method. (A) Schematic of the GKI-2.0 method. (B) The 1st step of GKI-2.0. A 5'-side homology arm (red box) and 3'-side homology (blue box) arm are subcloned into pENTR2-L3-PBL-R1 and pENTR2-R2-PBR-L4 by PCR and seamless cloning, respectively. HpaI, *Hpa*I restriction enzyme site; 5'ITR and 3'ITR, 5'-side and 3'-side of *Piggybac* inverted terminal repeats. (C) The 2nd step of GKI-2.0. X-marks indicate recombination that occurs between each corresponding *att*L and *att*R during the LR reaction. The entire sequences of pENTR2-L3-PBL-R1 and pENTR2-R2-PBR-L4 are attached in the S1 sequence.

**Fig 4:**
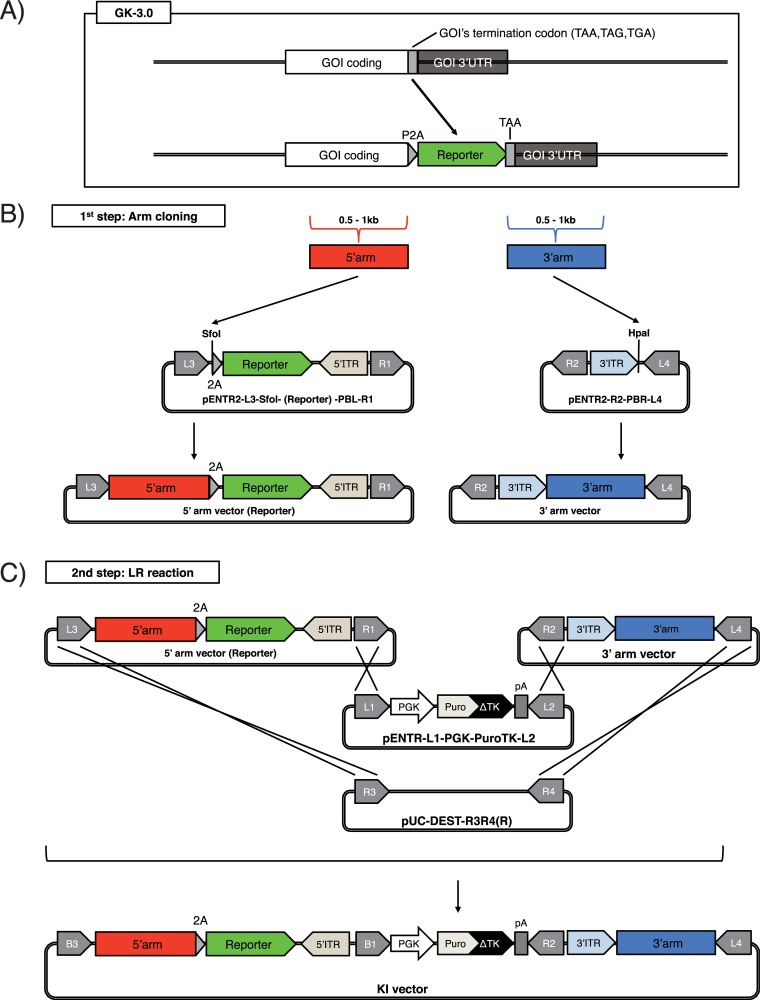
Schematic of the GKI-3.0 method. (A) Schematic of the GKI-3.0 method. P2A, porcine teschovirus-1 2A peptide sequence. (B) The 1st step of GKI-3.0. A 5'-side homology arm (red box) and 3'-side homology arm (blue box) are subcloned into each pENTR2-L3-SfoI-PBL-R1 reporter vector (described in Fig 5) and pENTR2-R2-PBR-L4 by PCR and seamless cloning, respectively. SfoI, *Sfo*I restriction enzyme site; HpaI, *Hpa*I restriction enzyme site. (C) The 2nd step of GKI-3.0. X-marks indicate recombination that occurs between each corresponding *att*L and *att*R during the LR reaction.

**Fig 5:**
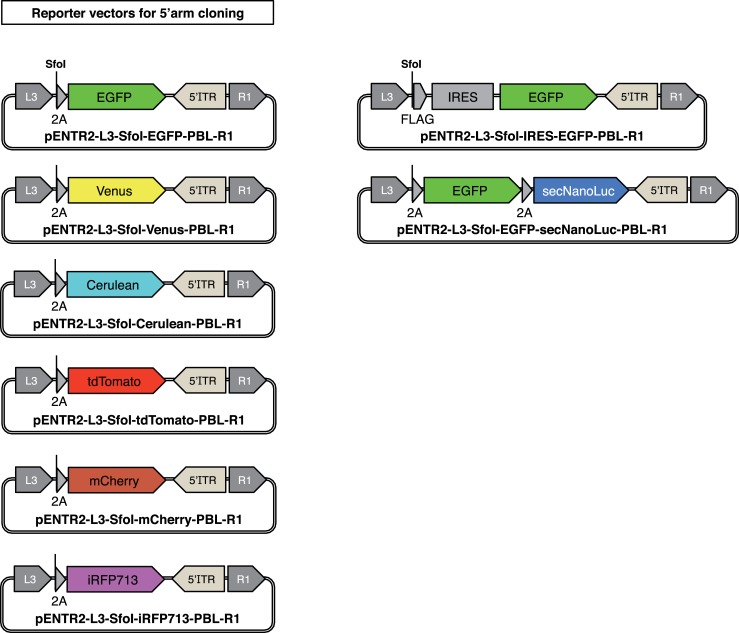
Reporter vectors for 5'arm cloning in the GKI-3.0 method. Reporter vectors generated for use in 5' arm cloning with the GK-3.0 method. 2A, porcine teschovirus-1 2A peptide sequence (*P2A*); FLAG, FLAG epitope tag; IRES, internal ribosomal entry site. The entire sequences of the described vectors are attached in the S1 sequence.

**Fig 6:**
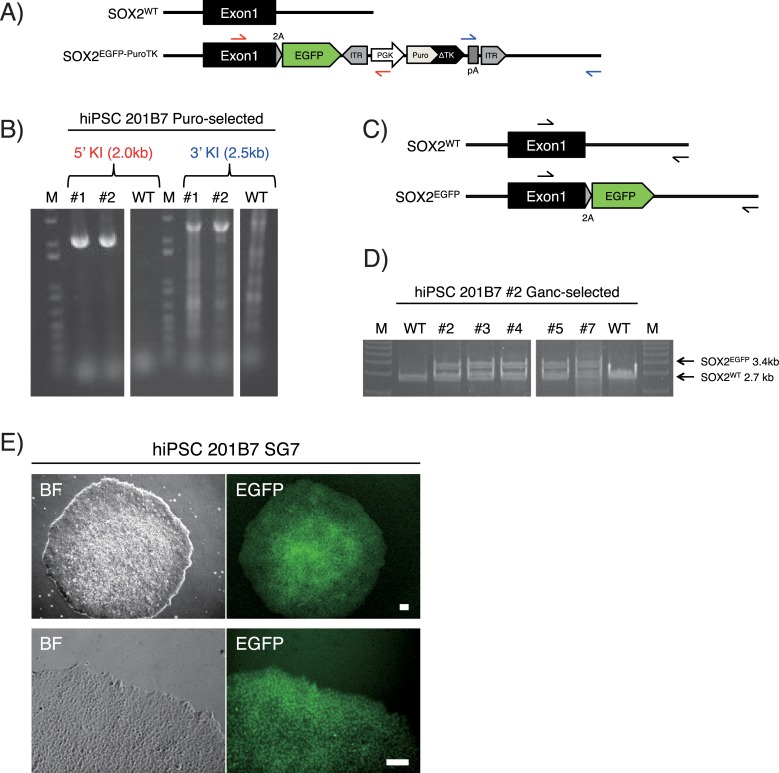
Generation of SOX2-EGFP hiPSCs. (A−B) Genotyping PCR analysis of SOX2-EGFP KI hiPSCs. Red arrows indicate the 5'-side genotyping PCR primers recognizing the 5' external region (hSOX2-forward) and the inner selection cassette (PGK-reverse). Blue arrows indicate the 3'-side genotyping PCR primers recognizing the inner selection cassette (pA-forward) and the 3' external region (hSOX2-reverse). By using these primer sets, specific PCR bands (2.0 kb and 2.5 kb) appeared in KI hiPSCs (#1 and #2) following transfection of the SOX2-EGFP KI vector and puromycin (Puro) selection. Separated images were cropped from the same gel. M, DNA marker; WT, wild-type 201B7 hiPSCs. (C−D) Genotyping PCR analysis of *PuroTK* cassette excision. Black arrows indicate 5' and 3' external primers (hSOX2-forward and hSOX2-reverse) for the analysis. Following transfection of *PBx* and ganciclovir (Ganc) selection, all of the five analyzed clones (#2, 3, 4, 5 and 7) had the cassette excised. Separated images were cropped from the same gel. (E) Representative images of hiPSCs harboring *SOX2*^*+/EGFP*^ *alleles* (SG7 clone) in bright field (BF) and green fluorescence (EGFP) at low (upper) and high (lower) magnification. Scale bars, 100 μm.

**Fig 7:**
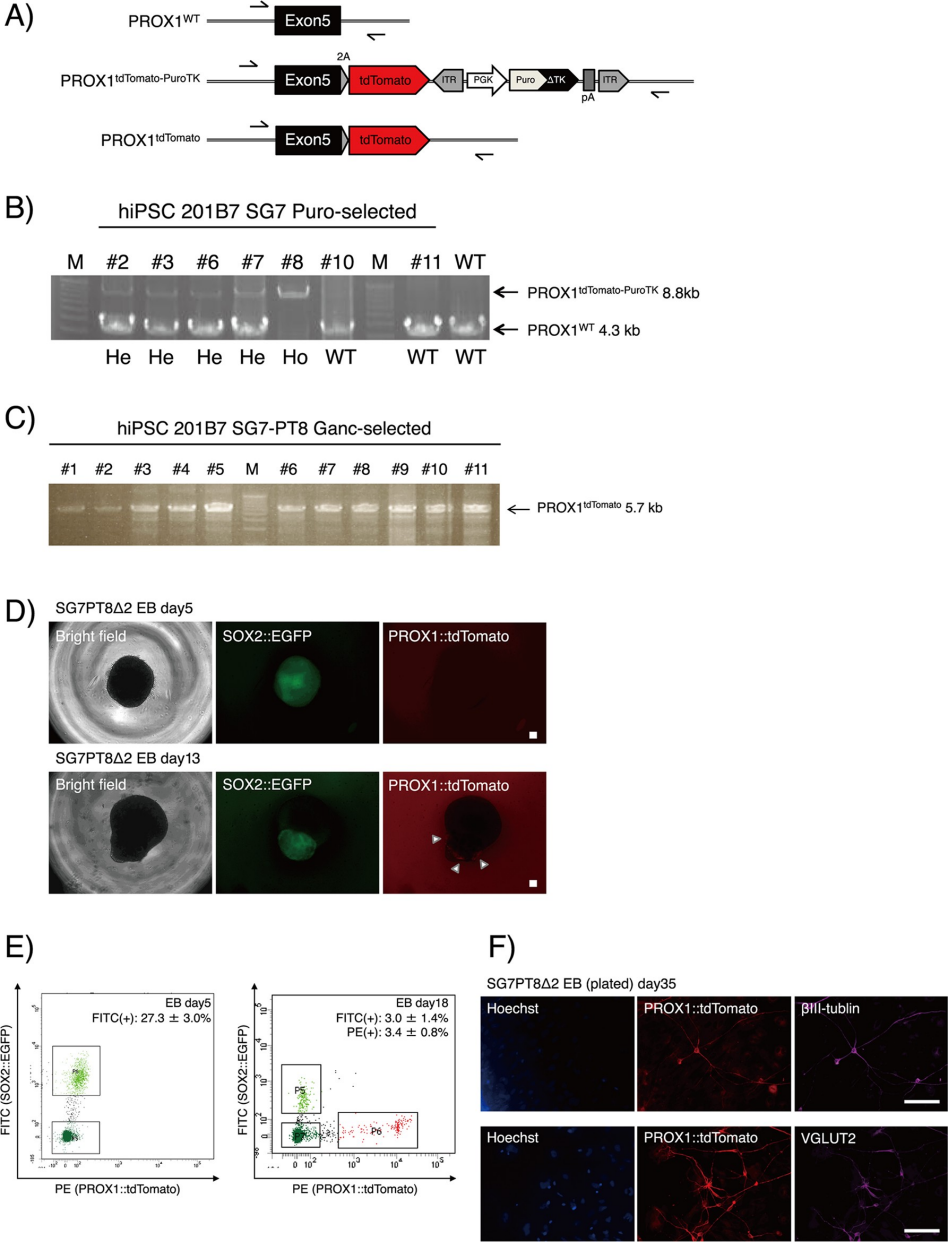
Generation and validation of SOX2-EGFP/PROX1-tdTomato hiPSCs. (A) Graphical schematic of genotyping PCR analysis of PROX1-tdTomato KI hiPSCs. Black arrows indicate the 5' and 3' external primers (hPROX1-forward and hPROX1-reverse) used for the analysis. (B) Genotyping PCR of PROX1-tdtomato KI. In total, we obtained four heterozygous-KI clones (#2, 3, 6 and 7) and one homozygous-KI clone (#8) among seven analyzed clones following puromycin selection. He, heterozygous-KI; Ho, homozygous-KI. (C) Genotyping PCR for the evaluation of *PuroTK* cassette excision. Following the transfection of the *PBx* vector into SG7-PT8 hiPSCs and ganciclovir selection, all of the eleven analyzed clones had the cassette excised homozygously. (D) Representative images of live EBs derived from SG7-PT8-Δ2 hiPSCs during hippocampal differentiation. White arrowheads indicate tdTomato-positive cells located at the surface of the protrusion. Scale bars, 100 μm. (E) FACS analysis of dissociated EBs on day 5 and day 18 during hippocampal differentiation. Doublets and dead cells were removed from the analysis with gate P2-P4 (not shown). This analysis was biologically and technically triplicated. (F) Representative images of immunochemical analysis of the differentiated SG7-PT8-Δ2 hiPSCs (day 35). Scale bars, 100 μm.

**Fig 8:**
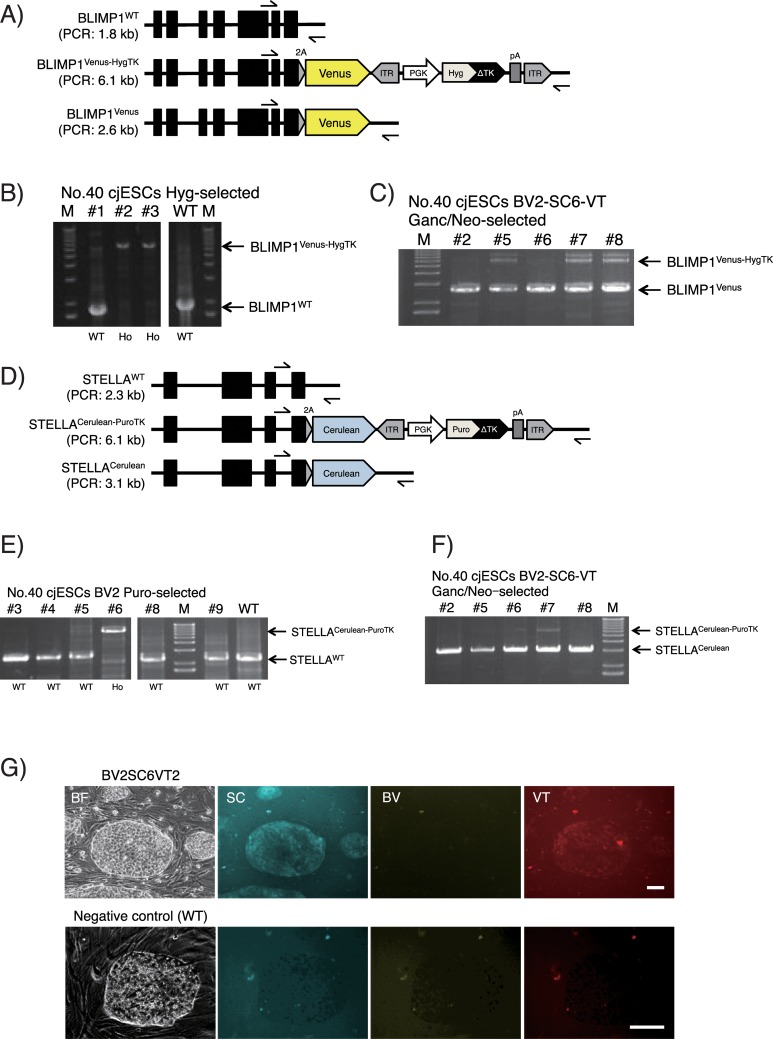
Generation of cjESCs harboring PGC-specific reporters. (A−C) Genotyping PCR analysis of the *BLIMP1* locus in cjESCs. Black arrows indicate the 5' and 3' external primers (cjBLIMP1-forward and cjBLIMP1-reverse) used for the analysis. Separated images were cropped from the same gel. (D−F) Genotyping PCR analysis of the *STELLA* locus in cjESCs. Black arrows indicate the 5' and 3' external primers (cjSTELLA-forward and cjSTELLA-reverse) for the analysis. Separated images were cropped from the same gel. (G) Representative images of undifferentiated BV2-SC6-VT2 cjESCs (upper) and WT cjESCs (lower) in bright field (BF), cyan fluorescence (SC), yellow fluorescence (BV) and red fluorescence (VT). Scale bars, 100 μm.

**Fig 9:**
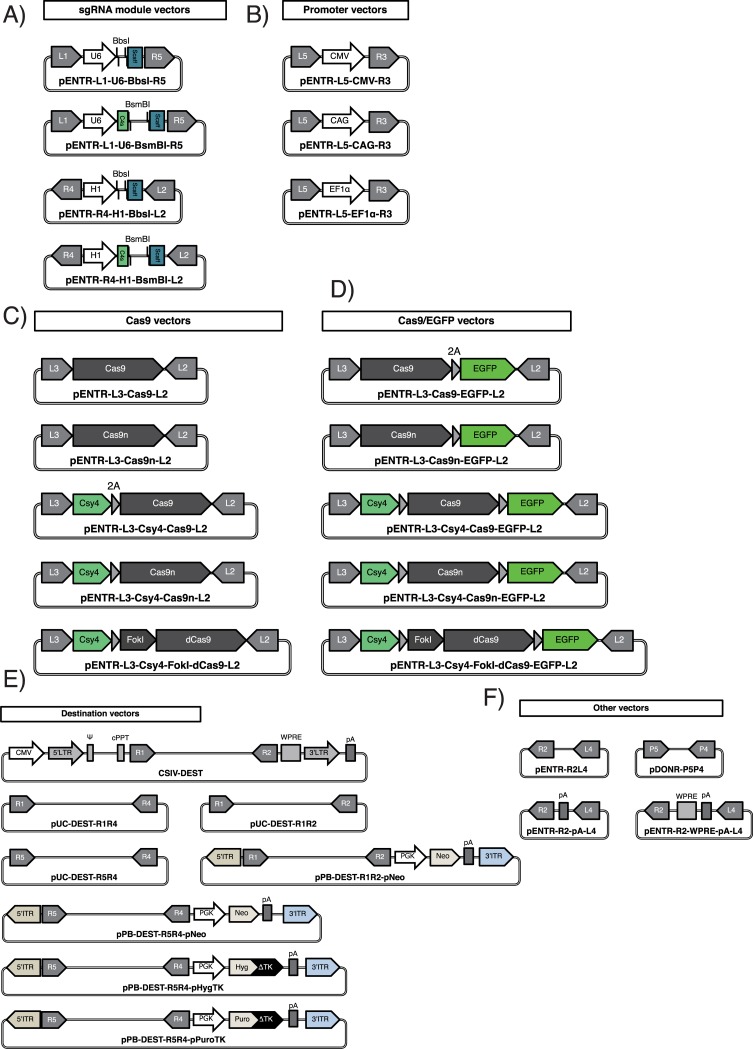
Multisite Gateway-mediated Cas9/gRNA expression system (GCas method). (A) sgRNA module vectors harboring the *att* sites. U6, mammalian U6 type III RNA polymerase III promoter; H1, H1 RNA polymerase III promoter; BbsI, a single sgRNA cloning site mediated by *Bbs*I digestion; BsmBI, a dual sgRNA cloning site mediated by *BsmB*I digestion; Scaff, scaffold sequence of sgRNA followed by TTTTTT (poly-T terminal signal of RNA polymerase III); C4s, a Csy4-mediated RNA cleavage site. See Figure E in S1 Supporting Information and the corresponding legend for the cloning of sgRNA(s) into the sgRNA module vectors. (B) Promoter vectors harboring *att*L5 and *att*R3. CMV, cytomegalovirus promoter; CAG, cytomegalovirus enhancer fused to the chicken *beta-actin* promoter and introns of chicken *beta-actin* and rabbit *beta-globin*; EF1α, *Elongation factor 1-alpha* promoter. (C−D) Vectors harboring Cas9 alone or Cas9/EGFP harboring *att*L3 and *att*L2. 2As are illustrated as right-pointing triangles. Cas9, *spCas9*; 2A, *Thosea asigna* virus 2A peptide sequence (*T2A*); Csy4, *Csy4* RNase of *Pseudomonas aeruginosa*; FokI, dimerization-dependent wild-type *Fok*I nuclease-domain; Cas9n, mutated *Cas9* nickase (D10A); dCas9, catalytically inactive *Cas9*. (E) Destination vectors for the GCas method. The *CmR* and *ccdB* gene are located between *att*R1 and *att*R2 in each destination vector (not described) for the positive/negative selection of transformants harboring the vector itself or the desired Cas9/gRNA expression vector. Gray arrows and boxes in CSIV-DEST indicate regulatory sequences for lentivirus production such as 5'LTR (5'-side long terminal repeat), Ψ (Packaging signal), cPPT (central polypurine tract), WPRE (woodchuck hepatitis virus posttranscriptional regulatory element) and 3'LTR (3'-side long terminal repeat). 5'ITR and 3'ITR, 5'-side and 3'-side of *Piggybac* inverted terminal repeats. The entire sequences of the vectors described are attached in the S1 sequence. (F) Other module vectors for the GCas method. pA, simian virus 40 polyadenylation signal sequence.

Plasmid vectors used and described in the current study were listed in the Table A in S1 Supporting Information, and schematic drawings are shown in Figs 1–5, 6A, 7A, 8A and 9. All the vectors will be provided by the corresponding authors upon request. We attached entire DNA sequences of the vectors in the S1 sequence. Recombinant DNA experiments were performed in accordance with institutional guidelines on Recombinant DNA experiments at Keio University, and approved by the Recombinant DNA Experiment Safety Committee of Keio University (approval number: 27–023 and 27–034).

### Gateway vectors

pUC-DEST-R3R4(R) (Fig 2B), pDONR-P3P1r (Fig 1B, left), pDONR-P2rP4 (Fig 1B, right), pENTR-L1-PGK-PuroTK-L2 (Fig 2A), pENTR2-L3-PBL-R1 (Fig 3B, left) and pENTR2-R2-PBR-L4 (Fig 3B, right) were generated and described previously [8, 12, 18–20].

pENTR-L1-PGK-HygTK-L2 (Fig 2A), pENTR-L1-PGK-Neo-L2 (Fig 2A), pENTR-L1-PGK-TKNeo-L2 (Fig 2A), pENTR-L1-PGK-TKBsd-L2 (Fig 2A), pUC-DEST-DTA-DEST-R3R4(R) (Fig 2B) were constructed from the corresponding vectors, such as pENTR-L1-PGK-PuroTK-L2 (Fig 2A) or pUC-DEST-R3R4(R) (Fig 2B).

pATD (Fig 2B) was constructed based on pUC-DEST-R3R4(R) and pSINTK.

pENTR2-L3-SfoI-EGFP-PBL-R1 (Fig 5, left), pENTR2-L3-SfoI-Venus-PBL-R1 (Fig 5, left), pENTR2-L3-SfoI-Cerulean-PBL-R1 (Fig 5, left), pENTR2-L3-SfoI-tdTomato-PBL-R1 (Fig 5, left), pENTR2-L3-SfoI-mCherry-PBL-R1 (Fig 5, left), pENTR2-L3-SfoI-iRFP713-PBL-R1 (Fig 5, left), pENTR2-L3-SfoI-IRES-EGFP-PBL-R1 (Fig 5, right) and pENTR2-L3-SfoI-EGFP-secNanoLuc-PBL-R1 (Fig 5, right) were constructed by inserting PCR-amplified fluorescent/luminescent protein genes into an *Hpa*I-digested pENTR2-L3-SfoI-R1 by seamless cloning with the GeneArt Seamless Cloning and Assembly Enzyme Mix (Thermo Fisher). To construct pENTR2-L3-SfoI-R1, a bacterial backbone of PX330 (a gift from Feng Zhang (Addgene plasmid # 42230)) was replaced with that of pENTR2-L3-PBL-R1 (Fig 3B, left), and the resultant vector was digested by *Age*I (NEB) and *EcoR*I (NEB), followed by ligation with an *Age*I and *Eco*RI-digested custom DNA fragment (IDT) which harbors the *att*L3 sequence, a *Sfo*I site, a *P2A* self-cleaving peptide sequence, an *Hpa*I site, a *Piggybac* 5'ITR and the *att*R1 sequence (reverse), in order from upstream to downstream.

To construct pDEST-spCas9-tdTomato, the T2A-tdTomato (silent mutations were introduced in the *tdTomato* gene for deleting two *Bbs*I sites) sequence (IDT custom oligo) was amplified and inserted into an *Eco*RI-digested PX330 by seamless cloning. The resultant vector was digested by *Not*I and *Sfo*I, and the *att*R3-CmR-ccdB-*att*R4 (reverse) fragment, amplified from pUC-DEST-R3R4(R), was inserted into the vector by seamless cloning.

### Cas9-gRNA vectors

For the Cas9-gRNA vector, we used pSpCas9(BB)-2A-Puro (PX459) (a gift from Feng Zhang (Addgene plasmid # 48139)).

For designing gRNA sequences, we used CRISPRdirect [21]. The gRNA target sequences (not including the PAM sequences) used in the current study are as follows: CCCCCCTCCAGTTCGCTGTC (for human *SOX2*), TGAAGAGTAGCAGTCCCCTT (for human *PROX1*), GGAAAATCTTAAGGATCCAT (for marmoset *BLIMP1*) and AAAGCTACTCTCTGCTCATA (for marmoset *STELLA*).

### KI vectors

To generate the human SOX2-EGFP KI vector, a 713 bp fragment directly upstream of the termination codon of the human *SOX2* gene was amplified from the human genomic DNA by PCR, and inserted into a *Sfo*I-digested pENTR2-L3-SfoI-EGFP-PBL-R1 (Fig 4B, left) by seamless cloning. Similarly, a 616 bp fragment directly downstream of the termination codon of the human *SOX2* gene was amplified and inserted into an *Hpa*I-digested pENTR2-R2-PBR-L4 by seamless cloning (Fig 4B, right). The resultant vectors were named pENTR2-hSOX2-5'arm-EGFP and pENTR2-hSOX2-3'arm, respectively. In addition, to make the latter unrecognizable by its corresponding gRNA, we performed mutagenesis by PCR using the following primers: TGAGGGTTGGACAGCGAACTGGAGGG and CTGTCCAACCCTCATTAACCCTAGAA. The resultant vector was named pENTR2-hSOX2-3'arm-mut. Then, pUC-DEST-R3R4(R), pENTR-L1-PGK-PuroTK-L2, pENTR2-hSOX2-5'arm-EGFP and pENTR2-hSOX2-3'arm-mut were mixed together and incubated with LR clonase II (Thermo Fisher) at room temperature overnight for LR reaction, and transformed into Stbl3 chemically competent *E*. *coli* (Stbl3; Thermo Fisher). The resultant vector was named pUC-DEST-hSOX2-EGFP-PuroTK (Fig 4C).

To generate a human PROX1-tdTomato KI vector, a 707 bp fragment directly upstream of the termination codon of the human *PROX1* gene was amplified from the human genomic DNA by PCR and inserted into a *Sfo*I-digested pENTR2-L3-SfoI-tdTomato-PBL-R1 by seamless cloning (Fig 4B, left). Similarly, a 736 bp fragment directly downstream of the termination codon of the human *PROX1* gene was amplified and inserted into an *Hpa*I-digested pENTR2-R2-PBR-L4 by seamless cloning (Fig 4B, right). The resultant vectors were named pENTR2-hPROX1-5'arm-tdTomato and pENTR2-hPROX1-3'arm, respectively. In addition, to make the latter unrecognizable by its corresponding gRNA, we performed mutagenesis by PCR using the following primers: CCCTTTAAATGTCCAAGTTATAT and GGACATTTAAAGGGGACTGCTAC. The resultant vector was named pENTR2-hPROX1-3'arm-mut. Then, pUC-DEST-R3R4(R), pENTR-L1-PGK-PuroTK-L2, pENTR2-hPROX1-5'arm-tdTomato and pENTR2-hPROX1-3'arm-mut were mixed together and incubated with LR clonase II at room temperature overnight for LR reaction and transformed into Stbl3. The resultant vector was named pUC-DEST-hPROX1-tdTomato-PuroTK (Fig 4C).

To generate a marmoset BLIMP1-Venus KI vector, a 611 bp fragment directly upstream of the termination codon of marmoset *BLIMP1* gene was amplified from the marmoset genomic DNA by PCR, and inserted into a *Sfo*I-digested pENTR2-L3-SfoI-Venus-PBL-R1 by seamless cloning (Fig 4B, left). Similarly, a 532 bp fragment directly downstream of the termination codon of the marmoset *BLIMP1* gene was amplified and inserted into an *Hpa*I-digested pENTR2-R2-PBR-L4 by seamless cloning (Fig 4B, right). The resultant vectors were named pENTR2-cjBLIMP1-5'arm-Venus and pENTR2-cjBLIMP1-3'arm, respectively. Then, pUC-DEST-R3R4(R), pENTR-L1-PGK-HygTK-L2, pENTR2-cjBLIMP1-5'arm-Venus and pENTR2-cjBLIMP1-3'arm were mixed together and incubated with LR clonase II at room temperature overnight for LR reaction, and transformed into Stbl3. The resultant vector was named pUC-DEST-cjBLIMP1-Venus-HygTK (Fig 4C).

To generate a marmoset STELLA-Cerulean KI vector, a 500 bp fragment directly upstream of the termination codon of the marmoset *STELLA* gene was amplified from the marmoset genomic DNA by PCR, and inserted into a *Sfo*I-digested pENTR2-L3-SfoI-Cerulean-PBL-R1 by seamless cloning (Fig 4B, left). Similarly, a 539 bp fragment directly downstream of the termination codon of the marmoset *STELLA* gene was amplified and inserted into an *Hpa*I-digested pENTR2-R2-PBR-L4 by seamless cloning (Fig 4B, right). The resultant vectors were named pENTR2-cjSTELLA-5'arm-Cerulean and pENTR2-cjSTELLA3-3'arm, respectively. In addition, to make the latter unrecognizable by its corresponding gRNA, we performed mutagenesis by PCR using the following primers: CTCATATAAATTATATATCAATAATTTTGA and ATATATAATTTATATGAGCAGAGAGTAGCTTTC. The resultant vector was named pENTR2-cjSTELLA-3'arm-mut. Then, pUC-DEST-R3R4(R), pENTR-L1-PGK-PuroTK-L2, pENTR2-cjSTELLA-5'arm-Cerulean and pENTR2-cjSTELLA-3'arm-mut were mixed together and incubated with LR clonase II at room temperature overnight for LR reaction, and transformed into Stbl3. The resultant vector was named pUC-DEST-cjSTELLA-mCerulean-PuroTK (Fig 4C).

### Piggybac vectors

For *Piggybac* transposase expression, we used pCMV-HyPBase (kindly provided by Kosuke Yusa and Allan Bradley (Sanger institute)) or pCAGS-PBx (System Bioscience).

To construct a *Piggybac*-based *VASA* reporter vector, a 3011 bp fragment directly upstream of the initiation codon of human *DDX4* (*VASA*) gene was amplified by PCR and inserted into pPB-tdTomato-pNeo. The resultant vector was named pPB-hVASA-tdTomato-pNeo.

### Primers for arm cloning, BP reaction and LR reaction in GKI methods

Abbreviations for the vector construction methods in the current study (GKI-1.0, 2.0 and 3.0) are described in the Results section. For GKI-1.0, to amplify the 0.5–1 kb 5'arm sequence containing *att*B1 in the 5' terminus, as well as *att*B3 and *lox*P in the 3' terminus (Fig 1B, left), we performed PCR using three primers as follows: GGGGCAACTTTGTATAATAAAGTTGNNNNNNNNNNNNNNNNNN (forward), TTCGTATAATGTATGCTATACGAAGTTATTNNNNNNNNNNNNNNNNNN (reverse) and GGGCTGCTTTTTTGTACAAACTTGATAACTTCGTATAATGTATGCTATAC, followed by BP reaction with pDONR-P3P1r. N (18 nt) indicates any nucleotide base.

Similarly, to amplify a 0.5–1 kb 3'arm sequence containing *att*B2 and *lox*P in the 5' terminus, as well as *att*B4 in the 3' terminus (Fig 1B, right), we performed PCR using three primers as follows: TTCGTATAGCATACATTATACGAAGTTATNNNNNNNNNNNNNNNNNN (forward), GGGGCAACTTTGTATAGAAAAGTTGNNNNNNNNNNNNNNNNNN (reverse) and GGGCAGCTTTCTTGTACAAAGTGGATAACTTCGTATAGCATACATTATAC, followed by BP reaction with pDONR-P2rP4.

For GKI-2.0, to amplify a 0.5–1 kb 5'arm sequence into an *Hpa*I-digested pENTR2-L3-PBL-R1 (Fig 3B, left), we performed PCR using CTTTGTATAATAAAGTTGTTNNNNNNNNNNNNNNNNNN (forward) and ATGATTATCTTTCTAGGGTTAANNNNNNNNNNNNNNNNNN (reverse), which was followed by seamless cloning. Similarly, to amplify a 0.5–1 kb 3'arm sequence into an *Hpa*I-digested pENTR2-R2-PBR-L4 (Fig 3B, right), we performed PCR using CAGACTATCTTTCTAGGGTTAANNNNNNNNNNNNNNNNNN (forward) and CAACTTTGTATAGAAAAGTTGTTNNNNNNNNNNNNNNNNNN (reverse), which was followed by seamless cloning.

For GKI-3.0, to amplify a 0.5–1 kb 5'arm sequence into each *Sfo*I-digested pCBh-SfoI reporter vector (Fig 4B, left), we performed PCR using AAGTTGTTGCCACCATGGGCNNNNNNNNNNNNNNNNNN (forward) and AACAGAGAGAAGTTCGTGGCNNNNNNNNNNNNNNNNNN (reverse), followed by seamless cloning. Amplification and seamless cloning of the 0.5–1 kb 3'arm sequence in GKI-3.0 was performed similarly as in GKI-2.0.

### Genotyping

For genotyping PCR analysis, cells were lysed overnight at 55°C in cell lysis buffer consisting of Tris-HCl (0.2 M), EDTA (10 mM), SDS (0.2%) and NaCl (0.2 M) in nuclease-free water with proteinase K (10 μg/ml). Genomic DNA was purified using a standard method with phenol-chloroform and ethanol. PrimeSTAR Max DNA polymerase (Takara Bio) was used for genotyping PCR, according to the manufacturer’s instructions. PCR was performed as follows: 30 s at 94°C; 35 cycles of 10 s at 98°C and 10 min at 68°C; then 10 min at 68°C; and a final incubation at 4°C. Following PCR reaction, gel electrophoresis (100 V, 30 min) was performed using 1% agarose gels and an 1 Kb Plus DNA Ladder (Thermo Fisher) as the DNA size marker. Primer sequences for genotyping PCR used in the current study are as follows: hSOX2-forward AAGATGCACAACTCGGAGATCAG, PGK-reverse TGCTGTCCATCTGCACGAGACTAGTGAG, pA-forward CGTCCGAGGGCAAAGGAATAGTCGAGA, hSOX2-reverse GGCTACAGGCTTGGACCCGC, hPROX1-forward TGGGCAGTTGCTCCGCTTCA, hPROX1-reverse CCCAGTGGCACACTGACACGA, cjBLIMP1-forward AGCACCATTCTGAAGACACGAAG, cjBLIMP1-reverse GGCAATACCCAAACCTCATTTTC, cjSTELLA-forward GCCACCTCTACCAATCAACATCA, cjSTELLA-reverse AGGATGCTTCCACAAAAACACAA.

### DNA sequencing

DNA sequencing analysis was performed using the BigDye Terminator v1.1 cycle sequencing kit (Thermo Fisher) with the 3130xl Genetic Analyzer (Applied Biosystems). The sequencing data presented in the figures were illustrated using the Snap Gene software (GSL Biotech). Primer sequences for DNA sequencing are as follows: cjBLIMP1-seq GAGGACGTGGAGGATGAC, cjSTELLA-seq TGTGTTCCCTGTTCCAACAT.

## Results

### Construction of KI vectors for the introduction or correction of mutations

Using the Multisite Gateway technology, we first established a method for the construction of vectors aimed at introducing or correcting mutations in specific loci. For this purpose, we built upon our previous methods for constructing protein expression vectors using the Multisite Gateway technology [8] to establish a new method for the generation of KI vectors harboring a bipotential selection marker cassette PGK-Puro-ΔTK (*PuroTK)* flanked by two *lox*P sequences and homology arms. This method was named the GKI-1.0 method (Multisite Gateway-mediated KI vector construction-version 1.0). Although we have previously reported successful KI using vectors generated by GKI-1.0 [12, 13, 16, 18], we have never described the detailed methodology and the multiple types of module vectors intended for the method, which we will do below.

The schematic for the method is shown in Fig 1A. To introduce or correct mutations in a gene of interest (GOI), SNs are targeted to induce double strand break (DSB) at the vicinity of the mutation site. In addition, to ensure that proper gene expression and splicing are maintained, the selection cassette is excised following homologous recombination, which results in one residual *lox*P sequence (34 bp) in the genome (Fig 1A, bottom).

As shown in Fig 1B and 1C, the GKI-1.0 method consists of two steps: (1) Arm cloning and (2) LR reaction. In the 1st step, 0.5−1 kb homology arms that are either directly upstream or downstream of the DSB site (the 5' arm and 3' arm, respectively) are amplified from the genome by PCR, and inserted into the pDONR vectors (pDONR-P3P1r and pDONR-P2rP4) by BP reaction (Fig 1B). For the PCR process, the following sequences are included at the 5' terminus of the primers to add the respective sequences to the corresponding arms (see Materials and methods): *att*B3 and *att*B1 with *lox*P for the 5' arm, and *att*B2 with *lox*P and *att*B4 for the 3' arm (Fig 1B, top). Following the BP reaction, as a result, the 5'arm vector harbors *att*L3 and *att*R1, and the 3' arm vector harbors *att*R2 and *att*L4 (Fig 1B, bottom).

Prior to the 2nd step, the 5' arm or 3'arm vector may need to undergo mutagenesis PCR depending on the desired construct. In the 2nd step, the 5' arm and 3'arm vectors, one of which harbors the mutation, a pENTR vector harboring the *PuroTK* cassette (pENTR-L1-PGK-PuroTK-L2), and a destination vector (pUC-DEST-R3R4(R)) undergo LR reaction (Fig 1C), after which the desired KI vector is finally obtained.

Furthermore, to enhance the utility of the method for further KI experiments, we generated multiple module vectors for use at the 2nd step of GKI-1.0 (Fig 2A and 2B). Using these module vectors, multiple different types of positive or negative selection markers can now be incorporated into the KI vectors, such as *Hygromycin resistance gene (Hyg)* fused to *ΔTK* (*HygTK*), *Neomycin resistance gene* (*Neo*), *Neo* fused to *ΔTK* (*TKNeo*) and *Blasticidin resistance gene (Bsd)* fused to *ΔTK* (*TKBsd*), which were previously validated for the selection of mammalian cells [22–24] (Fig 2A). In addition, we constructed destination vectors harboring negative selection markers for mammalian cells, such as *diphtheria toxin A (DT-A)* and Herpes Simplex Virus-1 *Thymidine Kinase (TK)*, which can be helpful for concentrating the homologous recombinants (Fig 2B). Furthermore, we constructed a destination vector whose backbone harbors expression cassettes of *Streptococcus pyogenes* Cas9 (spCas9) with tdTomato and a single-guide RNA (sgRNA) cloning site under the U6 promoter, for the construction of an all-in-one KI vector (Fig 2B, bottom). In Figs 1, 2 and 4, pUC-L1-PGK-PuroTK-L2 and pUC-DEST-R3R4(R) are just drawn as a representative selection cassette vector and a representative destination vector. They could be replaced with any other selection cassette vector and destination vector shown in Fig 2A and 2B.

### Minimization of residual sequences following KI and cassette excision

Although the generation of KI vectors using GKI-1.0 with multiple module vectors may facilitate KI experiments, the residual *lox*P sequence (34 bp length) in the genome may be problematic in some cases. For the introduction or correction of mutations in a GOI without leaving the *lox*P sequence in the genome, we slightly modified the method by replacing the *lox*P system with *piggyBac* transposon-based cassette excision system. The modified method was named GKI-2.0 (Fig 3A–3C). In the *piggyBac* system, the *piggyBac* transposase recognizes the transposon-specific inverted terminal repeats (ITRs) that are inserted on both ends of the target contents, which allows the transposase to move the contents from the TTAA site from its original position to different TTAA sites on other chromosomes or vectors [26]. As a result, no residual sequences remain and the TTAA sequence is left intact in the original position. Additionally, in an experimental setting, exploiting an excision-competent, integration-deficient *piggyBac* transposase (PBx) [27] enables the complete excision of the selection marker cassettes from the genome following homologous recombination, leaving only the short TTAA sequence behind in the genome (Fig 3A). Again, although we have previously reported successful KI using vectors generated by GKI-2.0 [19, 20], we have not described the detailed methodology, which we do below.

In a similar manner to GKI-1.0, the GKI-2.0 method consists of two steps: (1) Arm cloning and (2) LR reaction (Fig 3B and 3C). In the 1st step, 0.5−1 kb homology arms directly upstream or downstream of the DSB site (the 5'arm and 3'arm, respectively) are amplified from the genome by PCR, and inserted into *Hpa*I-digested pENTR2 vectors (pENTR2-L3-PBL-R1 and pENTR2-R2-PBR-L4) by seamless cloning (Fig 3B). Primer sequences for seamless cloning are described in Materials and Methods.

Prior to the 2nd step, the 5' arm or 3' arm vector may need to undergo mutagenesis PCR, depending on the desired construct. In the 2nd step, the 5'arm and 3'arm vectors, one of which harbors the mutation, a pENTR vector harboring the *PuroTK* cassette (pENTR-L1-PGK-PuroTK-L2), and a destination vector (pUC-DEST-R3R4(R)) undergo LR reaction (Fig 3C), after which the desired KI vector is obtained.

### Construction of KI vectors for the integration of fluorescent/luminescent protein genes

Precise integration of fluorescent/luminescent protein genes in specific loci enables adequate and faithful marking of specific cells. When exploiting the bi-cistronic expression system of the self-cleaving 2A peptide or the internal ribosomal entry site (*IRES*) [28], it is possible that the integration of reporter genes does not disrupt the expression of the targeted endogenous genes. Therefore, we established a new method based on GKI-2.0 to generate KI vectors for the precise integration of reporter genes using the 2A peptide sequence, with minimal effects on targeted endogenous genes. We named this method GKI-3.0.

To integrate reporter genes into a GOI locus without disrupting the expression of the gene, SNs are targeted to induce DSB at the vicinity of the termination codon of the gene (Fig 4A). Additionally, reporter genes are fused to the last amino acid-coding codon of the GOI via a 2A peptide sequence. Selection cassettes for concentrating homologous recombinants are excised by the *piggyBac* system following homologous recombination, similarly as in GKI-2.0.

The GKI-3.0 method consists of two steps: (1) Arm cloning and (2) LR reaction (Fig 4B and 4C). In the 1st step, a 0.5−1 kb 5'-side homology arm which is directly upstream of the termination codon of the GOI is amplified from the genome by PCR, and inserted into each *Sfo*I-digested pENTR2-L3-SfoI-PBL-R1 reporter vector by seamless cloning (Fig 4B, left). As shown in Fig 5, we constructed multiple reporter vectors containing fluorescent/luminescent reporter genes intended for use with the GKI-3.0 method, such as *Enhanced green fluorescent protein* (*EGFP*), variant of *Yellow fluorescent protein Venus*, *Cyan fluorescent protein Cerulean*, tandem dimer of *dTomato* (*tdTomato*), *monomeric Cherry fluorescent protein* (*mCherry*), *near-infrared fluorescent protein iRFP713* and *NanoLuc* fused to a secretion signal (*secNanoLuc*) [29].

On the other hand, a 0.5−1 kb 3' side homology arm, which contains the termination codon of the GOI and the direct downstream sequence of the termination codon, is amplified from the genome by PCR, and inserted into an *Hpa*I-digested pENTR2-R2-PBR-L4 (Fig 4B, right), similarly as in GKI-2.0 (Fig 3B, right).

In the 2nd step, the 5' arm vector harboring the reporter genes and the 3' arm vector, a pENTR vector-harboring a selection cassette, and a destination vector that undergoes LR reaction (Fig 4C), after which the desired KI vector is obtained.

### Application of the KI vectors generated with GKI-3.0 in human PSCs

To demonstrate the application of the GKI-3.0 method for stem cell research, we constructed two KI vectors, a SOX2-EGFP vector and a PROX1-tdTomato vector, with this method for transfection into human PSCs. In addition, we constructed Cas9-gRNA vectors (PX459 backbone [30]) containing guide RNA sequences targeting the vicinity of the termination codon of each target gene.

*Sex determining region Y-box 2 (SOX2)* is an indispensable factor for the maintenance of pluripotency and neuroectodermal differentiation from PSCs [31]. A previous study has shown that the depletion of *SOX2* expression in human PSCs leads to altered stem cell marker expression and trophectodermal differentiation [32]. *SOX2* is thus considered to be an important indicator for the pluripotency and differentiation states of PSCs, so we decided that the *SOX2* gene is a suitable target for the application of GKI-3.0.

After transfecting the hSOX2-EGFP KI vector with a corresponding Cas9-gRNA vector into 201B7 hiPSCs [11], we were able to obtain two heterozygous-KI clones (#1 and #2) following puromycin selection. KI was confirmed by genotyping PCR analysis (Fig 6A and 6B). Additionally, transfection of an expression vector of *PBx* into the #2 hiPSCs and ganciclovir selection resulted in the excision of the *PuroTK* cassette in all of the five analyzed clones (#2, 3, 4, 5 and 7; Fig 6C and 6D). Representative images of the #7 hiPSC clone (SG7) harboring the *SOX2*^*+/EGFP*^ alleles are shown in Fig 6E, showing a uniform intensity of EGFP fluorescence within the hiPSC colony.

We then attempted to perform an additional KI in the SG7 hiPSCs. *Prospero homeobox protein 1* (*PROX1*) has a crucial role in the development of lymphatic vessels and the dentate gyrus (DG) of the hippocampus [33, 34]. Hippocampal neurogenesis in the DG region, which gives rise to PROX1-positive excitatory granule neurons from SOX2-positive neural stem cells constantly throughout a lifetime [35], has an important role in learning and memory formation, and is known to be affected in neurodegenerative diseases such as Alzheimer's disease [36, 37]. In this context, hiPSCs harboring the double reporters of *SOX2* and *PROX1* could be used for the study of the hippocampal neurogenesis.

After transfecting the hPROX1-tdTomato KI vector with a corresponding Cas9-gRNA vector, we were able to obtain four heterozygous-KI clones (#2, 3, 6 and 7) and one homozygous-KI clone (#8) following puromycin selection. KI was confirmed by genotyping PCR analysis (Fig 7A and 7B). In addition, transfecting the PBx vector into the #8 hiPSCs and ganciclovir selection resulted in the excision of the *PuroTK* cassette in all of the eleven analyzed clones (Fig 7C).

Using one clone obtained following ganciclovir selection (#2 in Fig 7C, named SG7-PT8-Δ2), we differentiated the hiPSCs into hippocampal cells using the SFEBq method [17]. After differentiation for five days, the aggregated EBs showed strong, uniform EGFP fluorescence, while the tdTomato fluorescence could not be detected (Fig 7D, top). We were able to isolate EGFP-positive cells by FACS, which revealed that 27.3 ± 3.0% cells were EGFP-positive (Fig 7E, left), while there were few (less than 0.1%) tdTomato-positive cells. On day 13, strong EGFP fluorescence became restricted to the protrusive region of the EBs, while tdTomato-positive cells began to emerge at the surface of the protrusion (Fig 7D, bottom). On day 18, we quantified EGFP- and tdTomato-positive cells by FACS, which revealed that 3.0 ± 1.4% or 3.4 ± 0.8% cells were EGFP or tdTomato-positive (Fig 7E, right), respectively, with less than 0.1% EGFP/tdTomato-double-positive cells (Fig 7E, right). Our data reveal that during the initial days of differentiation, the percentage of tdTomato-positive cells increased, while that of EGFP-positive cells decreased, which suggests that the cells were indeed differentiating from its pluripotent/neuroectodermal state into hippocampal neurons.

Next, the EBs were collected, and transferred to a suspension culture for further differentiation, followed by a 5-day adherent culture. On day 35, we performed immunochemical analysis of the plated EBs, and observed that tdTomato-positive cells expressed a pan-neuronal marker βIII-tubulin and an excitatory neuronal marker VGLUT2 (Fig 7F), confirming that the cells are hippocampal neurons. Taken together, these results show that the vectors describe here can be used for tracking the differentiation of PSCs into hippocampal neurons, indicating the utility of this double-reporter system using the GKI-3.0 method in human PSCs.

### Application of KI vectors generated with GKI-3.0 in marmoset PSCs

In addition to the human PSC system, we performed KI in marmoset PSCs using vectors generated with GKI-3.0. *B-lymphocyte-induced maturation protein-1* (*BLIMP1*) is a critical factor for the specification of primordial germ cells (PGCs) in rodents and primates [38, 39]. In mice, derivation of BLIMP1/STELLA double-positive cells (termed PGC-like cells) from the PSCs resulted in the induction of functional gametes [40–42]. However, the function and expression pattern of *STELLA* in primates is yet to be elucidated [43]. Therefore, monitoring *BLIMP1* and *STELLA* expression is important for the verification of successful induction of germ cells. For this purpose, we constructed two KI vectors harboring BLIMP1-Venus (BV) and STELLA-Cerulean (SC).

After transfecting the BV-KI vector with the corresponding Cas9-gRNA vector into CMES40 cjESCs [14], we were able to obtain two homozygous-KI clones (#2 and 3; BV2 and BV3, respectively) among the three clones analyzed, following hygromycin selection (Fig 8A and 8B). DNA sequencing analysis revealed precise homozygous KI (Figure A1 in S1 Supporting Information).

We further performed an additional KI in the BV2 cjESCs with the SC-KI vector and the corresponding Cas9-gRNA vector. Following transfection and puromycin selection, we were able to obtain one homozygous-KI clone (#6, named BV2-SC6) among the six clones analyzed (Fig 8D and 8E). DNA sequencing analysis revealed precise homozygous KI (Figure B1 in S1 Supporting Information). We were also able to successfully generate a SC-KI cjESC clone named SC1 after transfecting the SC-KI vector into wild-type cjESCs (Figure A2 in S1 Supporting Information).

However, the efficiency of the excision of selection cassettes by PBx expression was low in the cjESC system. Upon excising selection cassettes from the SC1 cjESCs, four PBx transfections and ganciclovir selections were required to obtain cjESCs harboring homozygous *STELLA*^*Cerulean*^ alleles (SC1-Δ3, 5, 6 and 8; Figure B2 in S1 Supporting Information). Moreover, we did not obtain cjESCs harboring homozygous *BLIMP1*^*Venus*^ alleles from the BV2 and BV2-SC6 cjESCs within the four PBx transfections and ganciclovir selections (Figure C in S1 Supporting Information).

In order to obtain cassette-excised cjESCs more efficiently, we decided to use an expression vector of hyperactive integration-competent *piggyBac* transposase (*HyPBase*) [44] instead of *PBx*, along with an additional *piggyBac*-based vector to enable positive selection of the transposase-expressing cells. For this additional vector, we used pPB-hVASA-tdTomato-pNeo (VT), which contains a coding sequence of *tdTomato* under the control of the 3-kb human *VASA* promoter and a selection cassette of neomycin flanked by *piggyBac* ITRs (Figure A4 in S1 Supporting Information). Since *VASA* expression significantly increases from the late migrating stage of PGCs following specification and colonization of PGCs [45], the *VASA* reporter construct enables the monitoring of the maturation of PGCs following specification.

After one-time transfection of the VT reporter vector into the BV2-SC6 cjESCs, we isolated five colonies showing a dim tdTomato fluorescence following ganciclovir and G418 selection, since VASA is normally very weakly expressed in primate PSCs [46]. We demonstrated efficient excision of the selection cassettes in both the *BLIMP1* and *STELLA* loci in these isolated clones (2/5 and 3/5 analyzed clones; Fig 8C and 8F). As a result, we were able to obtain a cjESC line harboring homozygous *BLIMP1*^*Venus*^ and *STELLA*^*Cerulean*^ alleles, along with the VT transgene. This line was named BV2-SC6-VT2 (#2 in Fig 8C and 8F). The cjESCs harboring the *STELLA*^*Cerulean*^ alleles showed a weak fluorescence of Cerulean (Fig 8G and Figure C2 in S1 Supporting Information), although we failed to detect the Venus fluorescence from *BLIMP1*^*Venus*^ in the same cells (Fig 8G). Immunochemical analysis of the BV2-SC6-VT2 cjESCs using pluripotency markers (NANOG, SSEA4 and TRA-1-60) showed that the cells retained an undifferentiated state even after the integration of the triple (BV, SC and VT) reporters (Figure B4 in S1 Supporting Information), as well as a differentiation potential into all the three germ layers following EB formation (Figure C4 in S1 Supporting Information). Thus, the dual (positive and negative) selection strategy enables efficient cassette excision in marmoset PSCs, which facilitates the utility of the GKI-3.0 method in the system.

### Multisite Gateway-mediated Cas9/gRNA expression system

Up to this point, we have utilized the PX459-based Cas9/gRNA expression vectors [30] for validating the GKI-3.0 method in human and marmoset PSCs. However, an all-in-one Cas9/gRNA expression vector system with multiple modules would be more useful for KI experiments in mammalian cells. Therefore, we established a new Multisite Gateway-based construction method named GCas, and constructed novel vectors for this method (Fig 9). In this method, one or two sgRNAs are incorporated into the corresponding cloning sites (*Bbs*I for single sgRNA [30] and *Bsm*BI for dual sgRNAs [47]) of the sgRNA module vectors, *att*L1−*att*R5 or *att*R4−*att*L2, which are under the control of either the U6 or H1 promoter, respectively (Fig 9A and Figure E in S1 Supporting Information). Also, in addition to the *att*L3-*att*L2 module vectors containing the wildtype *spCas9* nuclease, we constructed module vectors carrying the nickase mutant (*Cas9n*), a catalytically dead mutant fused to the *Fok*I endonuclease (*FokI-dCas9*) [30, 47] (Fig 9C and 9D), and high-fidelity *spCas9* nucleases such as SpCas9-HF1, SpCas9-HF2, SpCas9-HF4, eSpCas9(1.1) and eSpCas9n(1.1) [48, 49] (Table A in S1 Supporting Information).

To construct a standard Cas9/gRNA expression vector, each *att*L1−*att*R5 sgRNA module vector, *att*L5−*att*R3 promoter vector (Fig 9B), a polyA vector pENTR-R2-pA-L4 (Fig 9F) and a destination vector pUC-DEST-R1R4 (Fig 9E), along with either *att*L3−*att*L2 Cas9 alone (Fig 9C) or Cas9/EGFP vector (Fig 9D), undergo LR reaction, after which the desired Cas9/gRNA vector is obtained. See Figure F in S1 Supporting Information for further applications of the GCas method.

## Discussion

In the present study, we introduced new methods, GKI-1.0, 2.0 and 3.0, for generating KI vectors, which are all based on the Multisite Gateway technology. Although SNs greatly improve the KI efficiency in various cells and animals [50], it is still necessary to perform positive selection for concentrating cells in which precise-KI occurred. In this context, since PSCs harbor a prominent potential of unlimited proliferation and differentiation into a plenty of cell types including those from all the three germ layers, it is important to validate the KI vectors generated by the GKI methods in PSCs. Thus, since the application of the KI vectors has been validated in human and marmoset PSCs in the present and previous studies [12, 13, 16, 18–20], we conclude that the methods can be robustly used in cell research.

Two previous studies, which described a KI vector construction method using the Multisite Gateway technology for murine ESCs or a human cell line [6, 10], exploited the Cre-*lox*P system for the excision of the positive selection cassette, which ends up with a 34 bp *lox*P sequence left behind in the genome. However, in our GKI-2.0 and 3.0 methods, we used an alternative approach of cassette excision using the *piggyBac* transposase system [26], which resulted in only a 4 bp (TTAA) residual sequence in the genome following excision. In this approach, when temporary integration of the selection cassette is targeted to a native TTAA sequence in the genome, it enables “footprint-free” KI following cassette excision. Since eukaryotic introns that have not been extensively characterized may in fact harbor functional regions [51, 52], our approach is ideal for reverse genetics.

Additionally, our module vectors for the GKI methods (Fig 2), in particular, the variety of positive/negative selection cassettes in hand (Fig 2A), would be useful for generating PSCs harboring homozygous or multiple KI alleles.

Mammalian adult neurogenesis continues throughout life only in two brain regions, such as DG in the hippocampus and subventricular zone in the cerebral cortex [53]. The spatial restriction of the phenomenon is due to the restricted stem cell niche in the adult brain, which is supported by unique microenvironments of the regions or unique cellular properties of the stem cells located in the regions [37]. Our double reporter system of *SOX2* and *PROX1* in human PSCs, which we generated and validated in the current study, would facilitate the *in vitro* study of this interesting phenomenon.

Although functional gametes have previously been derived from murine PSCs by transplantation into gonads or an *in vitro* long-term culture in reconstituted ovaries [40–42, 54], this has never been achieved in any other mammalian species. Two recent studies reported the successful induction of PGC-like cells from human PSCs using faithful KI reporters of PGC-specific genes such as *BLIMP1*, *TFAP2C* or *NANOS3* [39, 46]. However, because of the ethical difficulties, the human PSC system cannot be used for the functional evaluation of matured post-meiotic gametes, which may be obtained following the emergence of VASA-positive pre-meiotic gonocytes [55, 56]. Even in the murine systems [40–42, 54], embryonic gonadal niches or embryonic gonadal somatic cells are required for obtaining mature germ cells from PSCs, which is most likely accomplished through unidentified cellular or humoral signals [43]. In this context, because the use of human embryonic tissues is limited by ethical and technical issues, generating PSCs harboring faithful PGC reporters from non-human primate species is useful for studying primate gametogenesis. Furthermore, since marmosets have many advantages in reproduction research, including short gestation and sex maturation periods [57], the cjESCs harboring PGC-specific reporters may facilitate germ cell research.

## Supporting information

S1 Supporting information Including supporting methods, figures (Figs A-F), figure legends, a table, references.
(PDF)

S1 sequence Entire vector sequences in the current study. (DOCX)
