# Supplementary material for: A versatile toolbox for knock-in gene targeting based on the Multisite Gateway technology
Source: PLoS One. 2019 Aug 27;14(8):e0221164. doi: 10.1371/journal.pone.0221164 (PMC6711506; doi:10.1371/journal.pone.0221164)
Supplement: S1 Supporting information — Including supporting methods, figures (Figs A-F), figure legends, a table, references. (PDF) [file pone.0221164.s001.pdf]

Vector construction for the GCas method

pENTR-L1-U6-BbsI-R5 and pENTR-L1-U6-BsmBI-R5 (Fig 9A, top) were constructed from pDONR-P1P5r by inserting the U6-sgRNA cloning site/scaffold or U6-multiple sgRNA cloning site/scaffold amplified from PX459 [1] or pSQT1313 [2] (a gift from Keith Joung (Addgene plasmid # 53370)), respectively by BP cloning. Unwanted *Bbs*I and *Bsm*BI sites in the vector backbone were removed by site-directed mutagenesis.

pENTR-R4-H1-BbsI-L2 and pENTR-R4-H1-BsmBI-L2 (Fig 9A, bottom) were constructed from pENTR4-H1 (RIKEN BRC DNA BANK) by inserting the sgRNA cloning site/scaffold or multiple sgRNA cloning site/scaffold obtained from PX459 [1] or pSQT1313 [2] (a gift from Keith Joung (Addgene plasmid # 53370)), respectively. Then, the *att*L1 signal of pENTR4-H1 was replaced by the *att*R4 signal from pENTR-R4-L2 by seamless cloning. The unwanted *Bsm*BI site in the vector backbone was removed by site-directed mutagenesis.

Cas9 and Cas9/EGFP vectors (Figs 9C−D) were constructed by inserting the corresponding coding sequences obtained from PX458 [1] (a gift from Feng Zhang (Addgene plasmid # 48138)), PX461 [1] (a gift from Feng Zhang (Addgene plasmid # 48140)), pSQT834 [2] (a gift from Keith Joung (Addgene plasmid # 53371)), pNW3 [2] (a gift from Keith Joung (Addgene plasmid # 53372)), pSQT1601 [2] (a gift from Keith Joung (Addgene plasmid # 53369)), VP12 (a gift from Keith Joung (Addgene plasmid # 72247)), MSP2133 (a gift from Keith Joung (Addgene plasmid # 72249)), MSP2135 (a gift from Keith Joung (Addgene plasmid # 72248)) [3] and eSpCas9(1.1) (a gift from Feng Zhang (Addgene plasmid # 71814)) [4].

pENTR-R2-pA-L4 (Fig 9F) was constructed by inserting a simian virus 40 polyadenylation signal sequence between *att*R2 and *att*L4 of pENTR-R2L4. pENTR-R2-WPRE-pA-L4 (Fig 9F) was constructed by inserting a WPRE sequence amplified from pCXLE-EGFP (a gift from Shinya Yamanaka (Addgene plasmid # 27082) [5] by seamless cloning.

CSIV-DEST (Fig 9E) was constructed by removing the TRE promoter sequence from CSIV-TRE-RfA [6].

The construction of the remaining vectors were described previously [7].

S1 Fig

A)

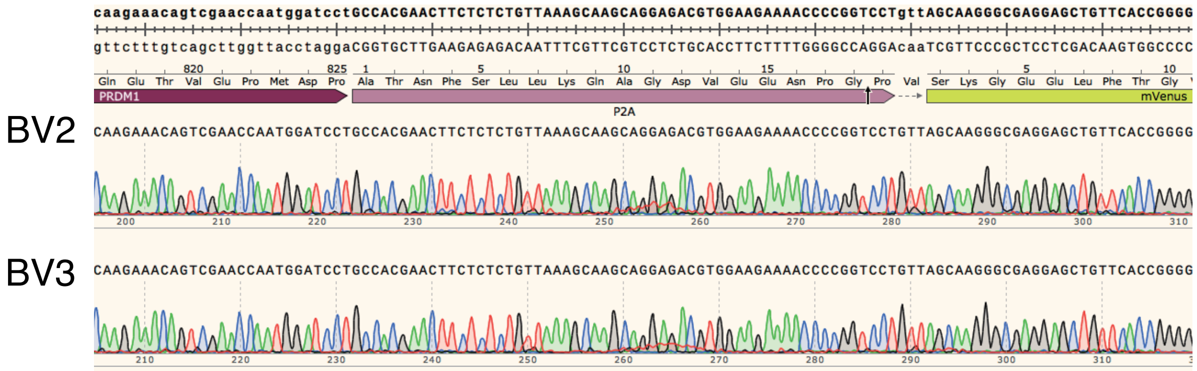

B)

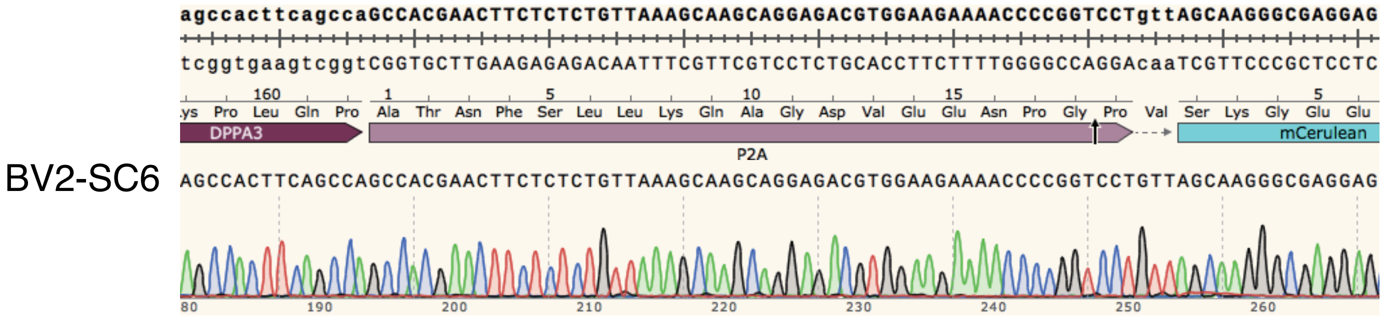

S2 Fig

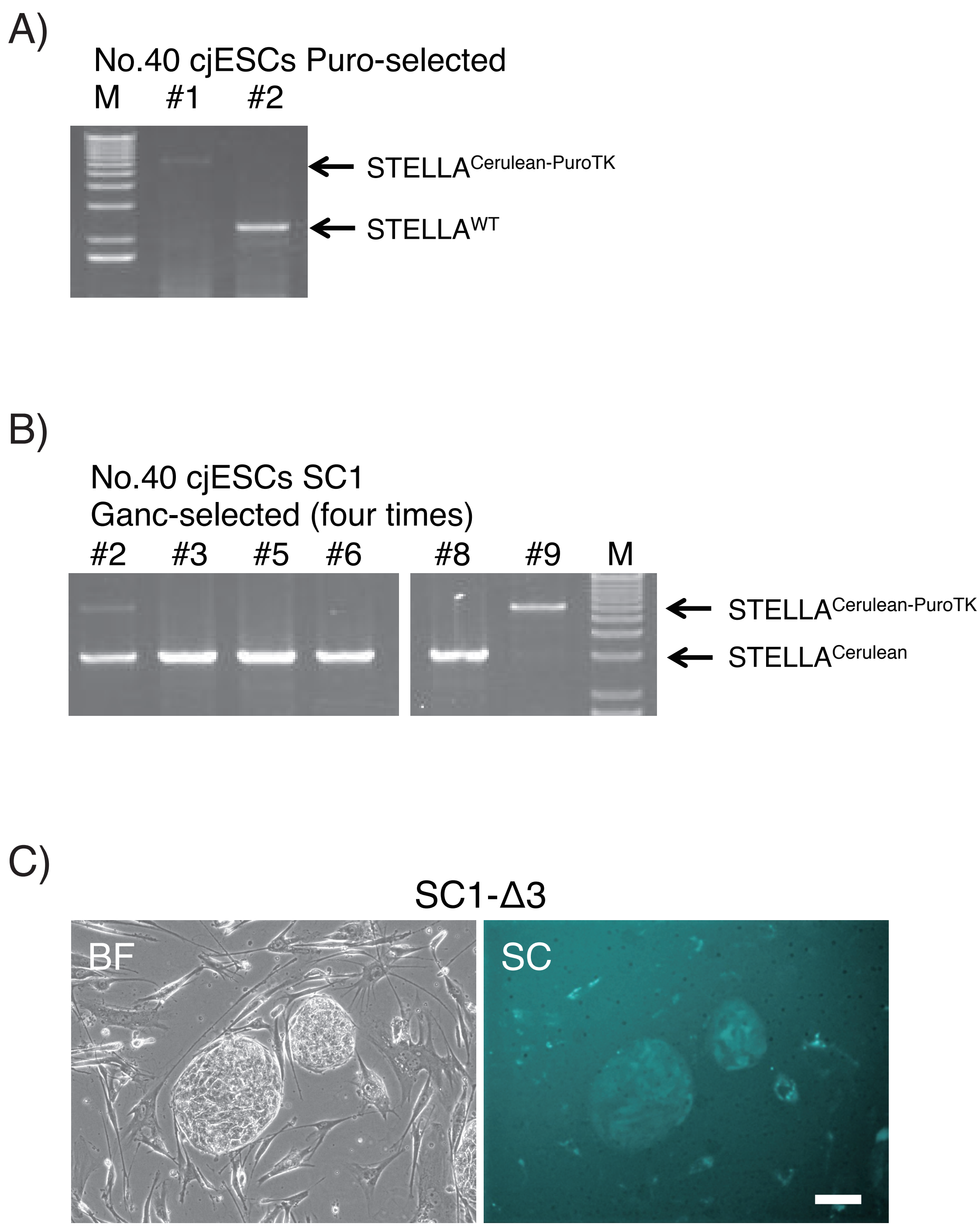

A)
No.40 cjESCs Puro-selected
M #1 #2
STELLA^Cerulean-PuroTK
STELLA^WT
B)
No.40 cjESCs SC1
Ganc-selected (four times)
#2 #3 #5 #6 #8 #9 M
STELLA^Cerulean-PuroTK
STELLA^Cerulean
C)
SC1-Δ3
BF
SC

S3 Fig

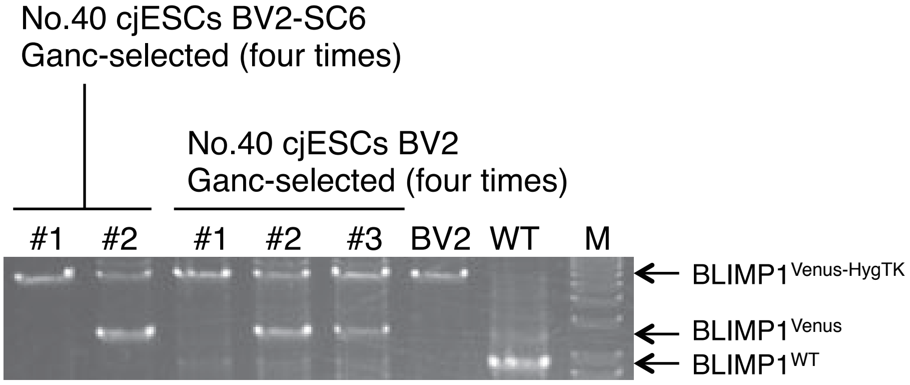

No.40 cjESCs BV2-SC6
Ganc-selected (four times)
No.40 cjESCs BV2
Ganc-selected (four times)
#1 #2 #1 #2 #3 BV2 WT M
BLIMP1Venus-HygTK
BLIMP1Venus
BLIMP1WT

S4 Fig

A)

pPB-VASA-tdTomato-pNeo (VT)

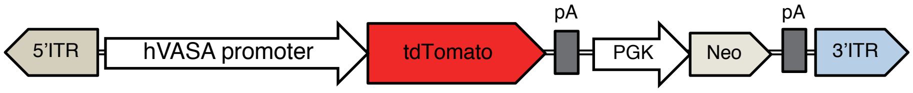

B)

No.40 cjESCs BV2-SC6-VT2

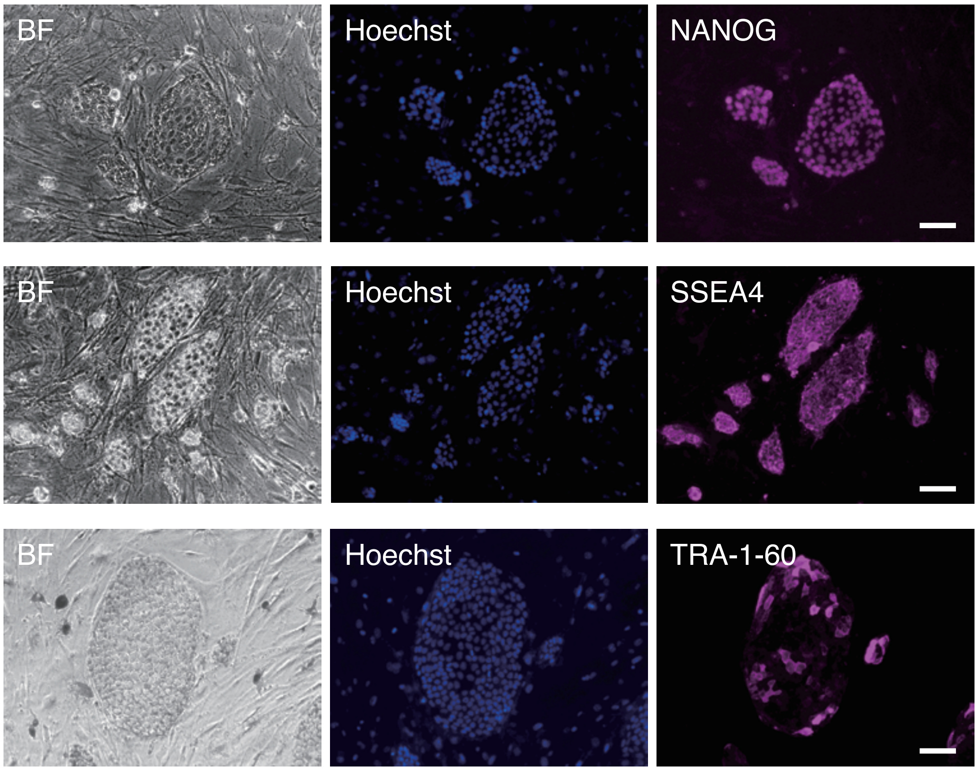

C)

No.40 cjESCs BV2-SC6-VT2

Ectoderm — Hoechst/βIII-tublin

Mesoderm — Hoechst/αSMA

Endoderm — Hoechst/HNF3β

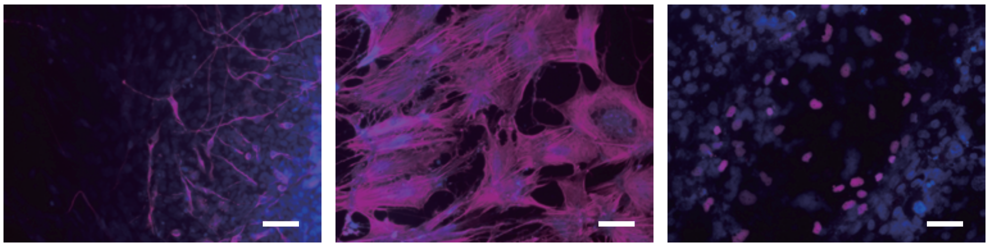

S5 Fig

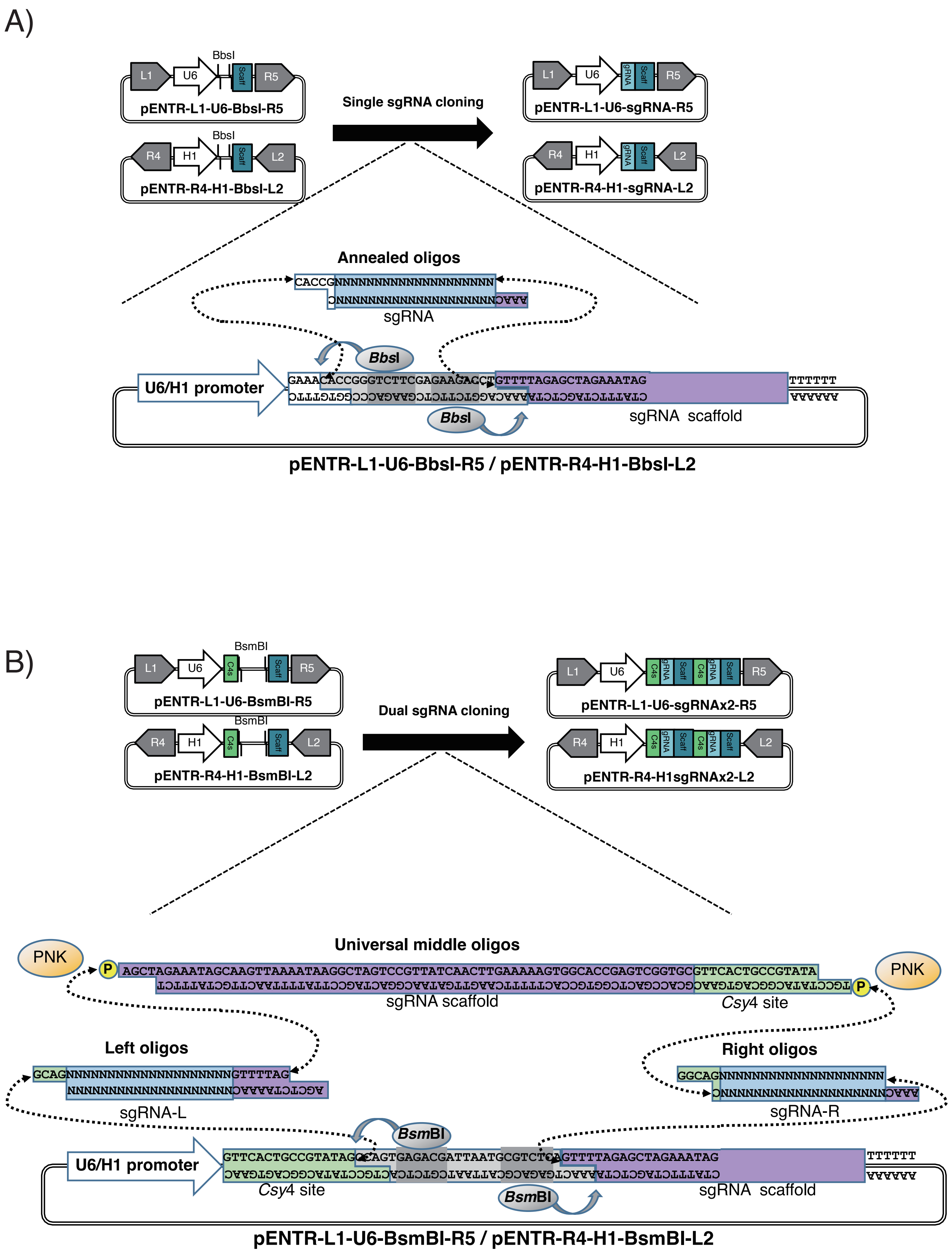
A)
BbsI
L1
U6
Scaff
R5
pENTR-L1-U6-BbsI-R5
R4
H1
L2
pENTR-R4-H1-BbsI-L2
Single sgRNA cloning
gRNA
pENTR-L1-U6-sgRNA-R5
pENTR-R4-H1-sgRNA-L2
Annealed oligos
CACCGNNNNNNNNNNNNNNNNNNNN
sgRNA
BbsI
U6/H1 promoter
GAAACACCGGGTCTTCGAGAAGACCTGTTTTAGAGCTAGAAATAG
TTTTTT
sgRNA scaffold
pENTR-L1-U6-BbsI-R5 / pENTR-R4-H1-BbsI-L2
B)
BsmBI
C4s
pENTR-L1-U6-BsmBI-R5
pENTR-R4-H1-BsmBI-L2
Dual sgRNA cloning
pENTR-L1-U6-sgRNAx2-R5
pENTR-R4-H1sgRNAx2-L2
Universal middle oligos
PNK
P
AGCTAGAAATAGCAAGTTAAAATAAGGCTAGTCCGTTATCAACTTGAAAAAGTGGCACCGAGTCGGTGCGTTCACTGCCGTATA
sgRNA scaffold
Csy4 site
Left oligos
GCAGNNNNNNNNNNNNNNNNNNNNGTTTTAG
sgRNA-L
Right oligos
GGCAGNNNNNNNNNNNNNNNNNNNN
sgRNA-R
BsmBI
U6/H1 promoter
GTTCACTGCCGTATAGGCAGTGAGACGATTAATGCGTCTCAGTTTTAGAGCTAGAAATAG
TTTTTT
Csy4 site
sgRNA scaffold
pENTR-L1-U6-BsmBI-R5 / pENTR-R4-H1-BsmBI-L2

S6 Fig

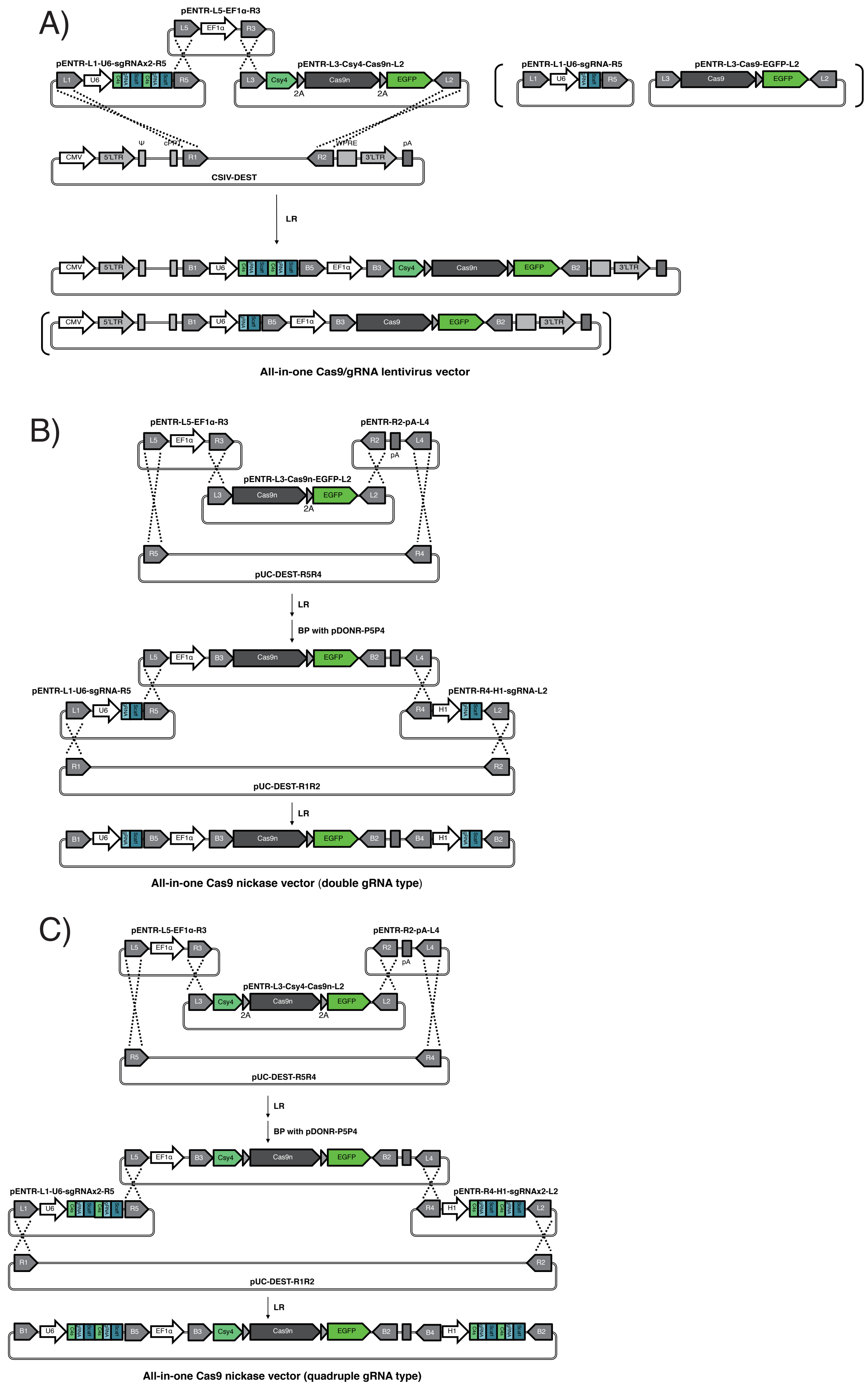
A)
pENTR-L5-EF1α-R3
pENTR-L1-U6-sgRNAx2-R5
pENTR-L3-Csy4-Cas9n-L2
pENTR-L1-U6-sgRNA-R5
pENTR-L3-Cas9-EGFP-L2
CSIV-DEST
LR
All-in-one Cas9/gRNA lentivirus vector
B)
pENTR-L5-EF1α-R3
pENTR-R2-pA-L4
pENTR-L3-Cas9n-EGFP-L2
pUC-DEST-R5R4
LR
BP with pDONR-P5P4
pENTR-L1-U6-sgRNA-R5
pENTR-R4-H1-sgRNA-L2
pUC-DEST-R1R2
LR
All-in-one Cas9 nickase vector (double gRNA type)
C)
pENTR-L5-EF1α-R3
pENTR-R2-pA-L4
pENTR-L3-Csy4-Cas9n-L2
pUC-DEST-R5R4
LR
BP with pDONR-P5P4
pENTR-L1-U6-sgRNAx2-R5
pENTR-R4-H1-sgRNAx2-L2
pUC-DEST-R1R2
LR
All-in-one Cas9 nickase vector (quadruple gRNA type)

S1 Fig (related to Fig 8). DNA sequencing analysis. (A) DNA sequencing analysis of BV cjESCs (BV2 and BV3). Genotyping PCR bands of the KI alleles (*BLIMP1*$^{Venus\text{-}HygTK}$) were extracted from the gel and used for the analysis. (B) DNA sequencing analysis of SC cjESCs (BV2-SC6). Genotyping PCR bands of the KI alleles (*STELLA*$^{Cerulean\text{-}PuroTK}$) were extracted from the gel and used for the analysis.

S2 Fig (related to Fig 8). Generation of SC cjESCs. (A−B) Genotyping PCR analysis of the *STELLA* locus in cjESCs. Separated images were cropped from the same gel. (C) Representative images of the cjESCs harboring homozygous *STELLA*^{Cerulean} alleles (SC1-Δ3) in bright field (BF) and cyan fluorescence (SC). Scale bars, 100 μm.

S3 Fig (related to Fig 8). Excision of selection cassettes from BV2/BV2-SC6 cjESCs. Genotyping PCR analysis of *BLIMP1* locus in cjESCs. Following four transfections of *PBx* and ganciclovir (Ganc) selections, we failed to obtain cjESCs harboring homozygous *BLIMP1*^{Venus} alleles.

S4 Fig (related to Fig 8). Generation and quality control of BV2-SC6-VT2 cjESCs. (A) Schematic of the *VASA* reporter vector used in the current study. 5'ITR and 3'ITR, 5'-side and 3'-side of *Piggybac* inverted terminal repeats; CMV, cytomegalovirus immediate early enhancer and promoter. (B) BV2-SC6-VT2 cjESCs were analyzed by immunochemistry using primary antibodies of pluripotency markers such as NANOG, SSEA4 and TRA-1-60, with Alexa 647-conjugated secondary antibodies. Scale bars, 100 μm. (C) Following EB formation, spontaneously differentiated BV2-SC6-VT2 cjESCs were analyzed by immunochemistry using primary antibodies of βIII-tublin (ectoderm), αSMA (medoderm) and HNF3β (endoderm) with Alexa 647-conjugated secondary antibodies. Scale bars, 100 μm.

S5 Fig (related to Fig 9A). Cloning of sgRNA(s) into sgRNA module vectors. (A) For single sgRNA cloning into the corresponding vectors (Fig 9A), two synthesized oligonucleotides (oligos) were annealed and ligated into each *Bbs*I-digested vector. The sequences of the required oligos were as follows: CACCGNNNNNNNNNNNNNNNNNNNN and AAACNNNNNNNNNNNNNNNNNNNNC. N (20 nt) indicates the sgRNA target

sequence directly upstream of the PAM sequence. (B) For dual sgRNA cloning into the corresponding vectors (Fig 9A), six synthesized oligonucleotides (oligos) were annealed and ligated into each *Bsm*BI-digested vector. For the left (upstream-side) sgRNA, the sequences of the required oligos were as follows: GCAGNNNNNNNNNNNNNNNNNNNNGTTTTAG and AGCTCTAAAACNNNNNNNNNNNNNNNNNNNN. For the right (downstream-side) sgRNA, the sequences of the required oligos were as follows: GGCAGNNNNNNNNNNNNNNNNNNNN and AAACNNNNNNNNNNNNNNNNNNNNC. In addition, the sequences of the middle oligos were as follows: AGCTAGAAATAGCAAGTTAAAATAAGGCTAGTCCGTTATCAACTTGAAAAAG TGGCACCGAGTCGGTGCGTTCACTGCCGTATA and TGCCTATACGGCAGTGAACGCACCGACTCGGTGCCACTTTTTCAAGTTGATA ACGGACTAGCCTTATTTTAACTTGCTATTTCT. Middle oligos were required for 5'-terminus phosphorylation with Polynucleotide Kinase (PNK) before annealing and ligation.

S6 Fig (related to Fig 9). Representative applications of the GCas method. (A) Lentirival Cas9/gRNA vector construction by the GCas method. For constructing an all-in-one lentiviral Cas9/gRNA expression vector, each *att*L1−*att*R5 sgRNA module vector (Fig 9A and S5 Fig), *att*L5−*att*R3 promoter vector (Fig 9B), *att*L3−*att*L2 Cas9 alone (Fig 9C) or Cas9/EGFP vector (Fig 9D) and a lentiviral destination vector CSIV-DEST (Fig 9E) underwent LR reaction, after which the desired Cas9/gRNA vector was obtained. A polyA vector was not used since it prevents lentivirus production when it is located between LTRs. In this figure, pENTR-L1-U6-sgRNA2-R5, pENTR-L5-EF1α-R3, pENTR-L3-Csy4-Cas9n-EGFP-L2 and CSIV-DEST (in parentheses: pENTR-L1-U6-sgRNA-R5 and pENTR-L3-Cas9-EGFP-L2) were used as an example.

(B) Construction of an all-in-one sgRNA/Cas9 vector harboring two sgRNAs at both ends using the GCas method. For construction, each *att*L5−*att*R3 promoter vector (Fig 9B), *att*L3−*att*L2 Cas9 alone (Fig 9C) or Cas9/EGFP vector (Fig 9D), a polyA vector pENTR-R2-pA-L4 (Fig 9F) and a destination vector pUC-DEST-R5R4 (Fig 9E) underwent LR reaction, after which an intermediate vector was obtained. Afterward, the intermediate vector underwent BP reaction with pDONR-P5P4 (Fig 9F), followed by LR reaction with

sgRNA sequence-incorporated pENTR-L1-U6-sgRNA-R5, pENTR-R4-H1-sgRNA-L2 (Fig 9A and S5A Fig) and pUC-DEST-R1R2 (Fig 9E). As a result, the desired all-in-one Cas9/gRNA vector was obtained. In this figure, pENTR-L5-EF1α-R3, pENTR-L3-Cas9n-EGFP-L2, pENTR-R2-pA-L4, pDONR-P5P4, pENTR-L1-U6-sgRNA-R5, pENTR-R4-H1-sgRNA-L2 and pUC-DEST-R1R2 were used as an example.

(C) Construction of an all-in-one sgRNA/Cas9 vector harboring four sgRNAs at both ends using the GCas method. For construction, each *att*L5−*att*R3 promoter vector (Fig 9B), *att*L3−*att*L2 Cas9 alone (Fig 9C) or Cas9/EGFP vector (Fig 9D), a polyA vector pENTR-R2-pA-L4 (Fig 9F) and a destination vector pUC-DEST-R5R4 (Fig 9E) underwent LR reaction, after which an intermediate vector was obtained. Afterward, the intermediate vector underwent BP reaction with pDONR-P5P4 (Fig 9F), followed by LR reaction with two sgRNA sequences-incorporated pENTR-L1-U6-sgRNA2-R5, pENTR-R4-H1-sgRNA2-L2 (Fig 9A and S5B Fig) and pUC-DEST-R1R2 (Fig 9E). As a result, the desired Cas9/gRNA vector was obtained. In this figure, pENTR-L5-EF1α-R3, pENTR-L3-Csy4-Cas9n-EGFP-L2, pENTR-R2-pA-L4, pDONR-P5P4, pENTR-L1-U6-sgRNA2-R5, pENTR-R4-H1-sgRNA2-L2 and pUC-DEST-R1R2 were used as an example.

S1 Table. Plasmid vector list.

Plasmid vector name	*att* sites	Size (bp)	Bacterial selection marker(s)
pENTR-L1-PGK-PuroTK-L2	L1, L2	4749	KanR
pENTR-L1-PGK-HygTK-L2	L1, L2	5225	KanR
pENTR-L1-PGK-Neo-L2	L1, L2	3766	KanR
pENTR-L1-PGK-TKNeo-L2	L1, L2	4953	KanR
pENTR-L1-PGK-TKBsd-L2	L1, L2	4546	KanR
pUC-DEST-R3R4(R)	R3, R4	4420	AmpR, CmR, ccdB
pUC-DTA-DEST-R3R4(R)	R3, R4	5981	AmpR, CmR, ccdB
pATD	R3, R4	5413	AmpR, CmR, ccdB
pDEST-Cas9-tdTomato	R3, R4	11687	AmpR, CmR, ccdB
pDONR-P3P1r	P3, P1	4793	KanR, CmR, ccdB
pDONR-P2rP4	P2, P4	4456	KanR, CmR, ccdB

pENTR2-L3-PBL-R1	L3, R1	2858	KanR
pENTR2-R2-HpaI-L4	R2, L4	2644	KanR
pENTR2-L3-SfoI-EGFP-PBL-R1	L3, R1	4572	KanR
pENTR2-L3-SfoI-Venus-PBL-R1	L3, R1	4572	KanR
pENTR2-L3-SfoI-Cerulean-PBL-R1	L3, R1	4572	KanR
pENTR2-L3-SfoI-tdTomato-PBL-R1	L3, R1	5283	KanR
pENTR2-L3-SfoI-mCherry-PBL-R1	L3, R1	4563	KanR
pENTR2-L3-SfoI-iRFP713-PBL-R1	L3, R1	4806	KanR
pENTR2-L3-SfoI-IRES-EGFP-PBL-R1	L3, R1	5184	KanR
pENTR2-L3-SfoI-EGFP-secNanoLuc-PBL-R1	L3, R1	5217	KanR
pUC-DEST-hSOX2-EGFP-PuroTK	B3, B1, B2, B4	7977	AmpR
pUC-DEST-hPROX1-tdTomato-PuroTK	B3, B1, B2, B4	8849	AmpR
pUC-DEST-cjBLIMP1-Venus-HygTK	B3, B1, B2, B4	8267	AmpR
pUC-DEST-cjSTELLA-mCerulean-PuroTK	B3, B1, B2, B4	7687	AmpR
pPB-VASA-tdTomato-pNeo	-	9854	AmpR
pENTR-L1-U6-BbsI-R5	L3, R5	2473	KanR
pENTR-L1-U6-BsmBI-R5	L3, R5	2483	KanR
pENTR-R4-H1-BbsI-L2	R4, L2	2326	KanR
pENTR-R4-H1-BsmBI-L2	R4, L2	2424	KanR
pENTR-L5-CMV-R3	L5, R3	2907	KanR
pENTR-L5-CAG-R3	L5, R3	4048	KanR
pENTR-L5-EF1α-R3	L5, R3	3627	KanR
pENTR-L3-Cas9-L2	L3, L2	6550	KanR
pENTR-L3-Cas9n-L2	L3, L2	6550	KanR
pENTR-L3-Csy4-Cas9-L2	L3, L2	7113	KanR
pENTR-L3-Csy4-Cas9n-L2	L3, L2	7113	KanR
pENTR-L3-Csy4-FokI-dCas9-L2	L3, L2	7746	KanR
pENTR-L3-Cas9-EGFP-L2	L3, L2	7354	KanR

pENTR-L3-Cas9n-EGFP-L2	L3, L2	7354	KanR
pENTR-L3-Csy4-Cas9-EGFP-L2	L3, L2	7848	KanR
pENTR-L3-Csy4-Cas9n-EGFP-L2	L3, L2	7848	KanR
pENTR-L3-Csy4-FokI-dCas9-EGFP-L2	L3, L2	8481	KanR
pENTR-L3-SpCas9-HF1-2A-EGFP-L2	L3, L2	7219	KanR
pENTR-L3-SpCas9-HF4-2A-EGFP-L2	L3, L2	7219	KanR
pENTR-L3-SpCas9-HF2-2A-EGFP-L2	L3, L2	7219	KanR
pENTR-L3-eSpCas9(1.1)-2A-EGFP-L2	L3, L2	7354	KanR
pENTR-L3-eSpCas9n(1.1)-2A-EGFP-L2	L3, L2	7354	KanR
CSIV-DEST	R1, R2	9397	AmpR, CmR, ccdB
pUC-DEST-R1R4	R1, R4	4957	AmpR, CmR, ccdB
pUC-DEST-R5R4	R5, R4	4957	AmpR, CmR, ccdB
pUC-DEST-R1R2	R1, R2	4458	AmpR, CmR, ccdB
pPB-DEST-R1R2-pNeo	R1, R2	6918	AmpR, CmR, ccdB
pPB-DEST-R5R4-pNeo	R5, R4	7474	AmpR, CmR, ccdB
pPB-DEST-R5R4-pHygTK	R5, R4	8868	AmpR, CmR, ccdB
pPB-DEST-R5R4-pPuroTK	R5, R4	8391	AmpR, CmR, ccdB
pENTR-R2L4	R2, L4	2342	KanR
pENTR-R2-pA-L4	R2, L4	2738	KanR
pENTR-R2-WPRE-pA-L4	R2, L4	3344	KanR
pDONR-P5P4	P5, P4	4422	KanR, CmR, ccdB

AmpR, *Ampicillin resistance gene*; KanR, *Kanamycin resistance gene*; CmR, *Chloramphenicol resistance gene*.

See also a reference [7] for detailed methods of molecular cloning in *E.coli*.

1. Ran FA, Hsu PD, Wright J, Agarwala V, Scott DA, Zhang F. Genome engineering using the CRISPR-Cas9 system. Nat Protoc. 2013;8(11):2281-308. Epub 2013/10/26. https://doi.org/10.1038/nprot.2013.143. PMID: 24157548

2. Tsai SQ, Wyvekens N, Khayter C, Foden JA, Thapar V, Reyon D, et al. Dimeric CRISPR RNA-guided FokI nucleases for highly specific genome editing. Nature Biotechnology. 2014;32(6):569-76. https://doi.org/10.1038/nbt.2908.

3. Kleinstiver BP, Pattanayak V, Prew MS, Tsai SQ, Nguyen NT, Zheng Z, et al. High-fidelity CRISPR-Cas9 nucleases with no detectable genome-wide off-target effects. Nature. 2016;529(7587):490-5. Epub 2016/01/07. https://doi.org/10.1038/nature16526. PMID: 26735016

4. Slaymaker IM, Gao L, Zetsche B, Scott DA, Yan WX, Zhang F. Rationally engineered Cas9 nucleases with improved specificity. Science. 2016;351(6268):84-8. Epub 2015/12/03. https://doi.org/10.1126/science.aad5227. PMID: 26628643

5. Okita K, Yamakawa T, Matsumura Y, Sato Y, Amano N, Watanabe A, et al. An efficient nonviral method to generate integration-free human-induced pluripotent stem cells from cord blood and peripheral blood cells. Stem Cells. 2013;31(3):458-66. Epub 2012/11/30. https://doi.org/10.1002/stem.1293. PMID: 23193063

6. Zhou Z, Kohda K, Ibata K, Kohyama J, Akamatsu W, Yuzaki M, et al. Reprogramming non-human primate somatic cells into functional neuronal cells by defined factors. Mol Brain. 2014;7:24. Epub 2014/04/04. https://doi.org/10.1186/1756-6606-7-24. PMID: 24694048

7. Sone T, Imamoto F. Methods for constructing clones for protein expression in mammalian cells. Methods Mol Biol. 2012;801:227-50. Epub 2011/10/12. https://doi.org/10.1007/978-1-61779-352-3_15. PMID: 21987257
