# Supplementary material for: A versatile toolbox for knock-in gene targeting based on the Multisite Gateway technology
Source: PLoS One. 2019 Aug 27;14(8):e0221164. doi: 10.1371/journal.pone.0221164 (PMC6711506; doi:10.1371/journal.pone.0221164)
Supplement: S1 sequence — (DOCX) [file pone.0221164.s002.docx]

pENTR-L1-PGK-PuroTK-L2

tccgggccgttgcttcacaacgttcaaatccgctcccggcggatttgtcctactcaggagagcgttcaccgacaaacaacagataaaacgaaaggcccagtcttccgactgagcctttcgttttatttgatgcctggcagttccctactctcgcgttaacgctagcatggatctcgggccccaaataatgattttattttgactgatagtgacctgttcgttgcaacaaattgatgagcaatgcttttttataatgccaactttgtacaaaaaagcaggaattctaccgggtaggggaggcgcttttcccaaggcagtctggagcatgcgctttagcagccccgctgggcacttggcgctacacaagtggcctctggcctcgcacacattccacatccaccggtaggcgccaaccggctccgttctttggtggccccttcgcgccaccttctactcctcccctagtcaggaagttcccccccgccccgcagctcgcgtcgtgcaggacgtgacaaatggaagtagcacgtctcactagtctcgtgcagatggacagcaccgctgagcaatggaagcgggtaggcctttggggcagcggccaatagcagctttgctccttcgctttctgggctcagaggctgggaaggggtgggtccgggggcgggctcaggggcgggctcaggggcggggcgggcgcccgaaggtcctccggaggcccggcattctgcacgcttcaaaagcgcacgtctgccgcgctgttctcctcttcctcatctccgggcctttcgaccgatcatcaagcttgatcctcatgaccgagtacaagcccacggtgcgcctcgccacccgcgacgacgtccccagggccgtacgcaccctcgccgccgcgttcgccgactaccccgccacgcgccacaccgtcgatccggaccgccacatcgagcgggtcaccgagctgcaagaactcttcctcacgcgcgtcgggctcgacatcggcaaggtgtgggtcgcggacgacggcgccgcggtggcggtctggaccacgccggagagcgtcgaagcgggggcggtgttcgccgagatcggcccgcgcatggccgagttgagcggttcccggctggccgcgcagcaacagatggaaggcctcctggcgccgcaccggcccaaggagcccgcgtggttcctggccaccgtcggcgtctcgcccgaccaccagggcaagggtctgggcagcgccgtcgtgctccccggagtggaggcggccgagcgcgccggggtgcccgccttcctggagacctccgcgccccgcaacctccccttctacgagcggctcggcttcaccgtcaccgccgacgtcgaggtgcccgaaggaccgcgcacctggtgcatgacccgcaagcccggtgccggatccatgcccacgctactgcgggtttatatagacggtccccacgggatggggaaaaccaccaccacgcaactgctggtggccctgggttcgcgcgacgatatcgtctacgtacccgagccgatgacttactggcgggtgctgggggcttccgagacaatcgcgaacatctacaccacacaacaccgcctcgaccagggtgagatatcggccggggacgcggcggtggtaatgacaagcgcccagataacaatgggcatgccttatgccgtgaccgacgccgttctggctcctcatatcgggggggaggctgggagctcacatgccccgcccccggccctcaccctcatcttcgaccgccatcccatcgccgccctcctgtgctacccggccgcgcggtaccttatgggcagcatgaccccccaggccgtgctggcgttcgtggccctcatcccgccgaccttgcccggcaccaacatcgtgcttggggcccttccggaggacagacacatcgaccgcctggccaaacgccagcgccccggcgagcggctggacctggctatgctggctgcgattcgccgcgtttacgggctacttgccaatacggtgcggtatctgcagtgcggcgggtcgtggcgggaggactggggacagctttcggggacggccgtgccgccccagggtgccgagccccagagcaacgcgggcccacgaccccatatcggggacacgttatttaccctgtttcgggcccccgagttgctggcccccaacggcgacctgtataacgtgtttgcctgggccttggacgtcttggccaaacgcctccgttccatgcacgtctttatcctggattacgaccaatcgcccgccggctgccgggacgccctgctgcaacttacctccgggatggtccagacccacgtcaccacccccggctccataccgacgatatgcgacctggcgcgcacgtttgcccgggagatgggggaggctaactgagctctagcttatcgactcgccgatagtggaaaccgacgccccagcactcgtccgagggcaaaggaatagtcgagaaattgatgatctattaaacaataaagatgtccacatggaagtttttcctgtcatactttgttaagaagggtgagaacagagtacctacattttgaatggaaggattggagctacgggggtgggggtggggtgggattagataaatgcctgctctttactgaaggctctttactattgctttatgataatgtttcatagttggatatcataatttaaacaagcaaaaccaaattaagggccagctcattcctcccactcatgatctatagatcctctagagtcgagggctgcagcagctttcttgtacaaagttggcattataagaaagcattgcttatcaatttgttgcaacgaacaggtcactatcagtcaaaataaaatcattatttgccatccagctgcagctctggcccgtgtctcaaaatctctgatgttacattgcacaagataaaaatatatcatcatgaacaataaaactgtctgcttacataaacagtaatacaaggggtgttatgagccatattcaacgggaaacgtcgaggccgcgattaaattccaacatggatgctgatttatatgggtataaatgggctcgcgataatgtcgggcaatcaggtgcgacaatctatcgcttgtatgggaagcccgatgcgccagagttgtttctgaaacatggcaaaggtagcgttgccaatgatgttacagatgagatggtcagactaaactggctgacggaatttatgcctcttccgaccatcaagcattttatccgtactcctgatgatgcatggttactcaccactgcgatccccggaaaaacagcattccaggtattagaagaatatcctgattcaggtgaaaatattgttgatgcgctggcagtgttcctgcgccggttgcattcgattcctgtttgtaattgtccttttaacagcgatcgcgtatttcgtctcgctcaggcgcaatcacgaatgaataacggtttggttgatgcgagtgattttgatgacgagcgtaatggctggcctgttgaacaagtctggaaagaaatgcataaacttttgccattctcaccggattcagtcgtcactcatggtgatttctcacttgataaccttatttttgacgaggggaaattaataggttgtattgatgttggacgagtcggaatcgcagaccgataccaggatcttgccatcctatggaactgcctcggtgagttttctccttcattacagaaacggctttttcaaaaatatggtattgataatcctgatatgaataaattgcagtttcatttgatgctcgatgagtttttctaatcagaattggttaattggttgtaacactggcagagcattacgctgacttgacgggacggcgcaagctcatgaccaaaatcccttaacgtgagttttcgttccactgagcgtcagaccccgtagaaaagatcaaaggatcttcttgagatcctttttttctgcgcgtaatctgctgcttgcaaacaaaaaaaccaccgctaccagcggtggtttgtttgccggatcaagagctaccaactctttttccgaaggtaactggcttcagcagagcgcagataccaaatactgtccttctagtgtagccgtagttaggccaccacttcaagaactctgtagcaccgcctacatacctcgctctgctaatcctgttaccagtggctgctgccagtggcgataagtcgtgtcttaccgggttggactcaagacgatagttaccggataaggcgcagcggtcgggctgaacggggggttcgtgcacacagcccagcttggagcgaacgacctacaccgaactgagatacctacagcgtgagctatgagaaagcgccacgcttcccgaagggagaaaggcggacaggtatccggtaagcggcagggtcggaacaggagagcgcacgagggagcttccagggggaaacgcctggtatctttatagtcctgtcgggtttcgccacctctgacttgagcgtcgatttttgtgatgctcgtcaggggggcggagcctatggaaaaacgccagcaacgcggcctttttacggttcctggccttttgctggccttttgctcacatgttctttcctgcgttatcccctgattctgtggataaccgtattaccgctagccaggaagagtttgtagaaacgcaaaaaggccatccgtcaggatggccttctgcttagtttgatgcctggcagtttatggcgggcgtcctgcccgccaccc

pENTR-L1-PGK-HygTK-L2

tccgggccgttgcttcacaacgttcaaatccgctcccggcggatttgtcctactcaggagagcgttcaccgacaaacaacagataaaacgaaaggcccagtcttccgactgagcctttcgttttatttgatgcctggcagttccctactctcgcgttaacgctagcatggatctcgggccccaaataatgattttattttgactgatagtgacctgttcgttgcaacaaattgatgagcaatgcttttttataatgccaactttgtacaaaaaagcagaattctaccgggtaggggaggcgcttttcccaaggcagtctggagcatgcgctttagcagccccgctgggcacttggcgctacacaagtggcctctggcctcgcacacattccacatccaccggtaggcgccaaccggctccgttctttggtggccccttcgcgccaccttctactcctcccctagtcaggaagttcccccccgccccgcagctcgcgtcgtgcaggacgtgacaaatggaagtagcacgtctcactagtctcgtgcagatggacagcaccgctgagcaatggaagcgggtaggcctttggggcagcggccaatagcagctttgctccttcgctttctgggctcagaggctgggaaggggtgggtccgggggcgggctcaggggcgggctcaggggcggggcgggcgcccgaaggtcctccggaggcccggcattctgcacgcttcaaaagcgcacgtctgccgcgctgttctcctcttcctcatctccgggcctttcgaccgatcatcaagcttgatcctcatgaaaaagcctgaactcaccgcgacgtctgtcgagaagtttctgatcgaaaagttcgacagcgtctccgacctgatgcagctctcggagggcgaagaatctcgtgctttcagcttcgatgtaggagggcGtggatatgtcctgcgggtaaatagctgcgccgatggtttctacaaagatcgttatgtttatcggcactttgcatcggccgcgctcccgattccggaagtgcttgacattggggaattcagcgagagcctgacctattgcatctcccgccgtgcacagggtgtcacgttgcaagacctgcctgaaaccgaactgcccgctgttctgcagccggtcgcggaggccatggatgcgatcgctgcggccgatcttagccagacgagcgggttcggcccattcggaccgcaaggaatcggtcaatacactacatggcgtgatttcatatgcgcgattgctgatccccatgtgtatcactggcaaactgtgatggacgacaccgtcagtgcgtccgtcgcgcaggctctcgatgagctgatgctttgggccgaggactgccccgaagtccggcacctcgtgcacgcggatttcggctccaacaatgtcctgacggacaatggccgcataacagcggtcattgactggagcgaggcgatgttcggggattcccaatacgaggtcgccaacatcttcttctggaggccgtggttggcttgtatggagcagcagacgcgctacttcgagcggaggcatccggagcttgcaggatcgccgcggctccgggcgtatatgctccccattggtcttgaccaactctatcagagcttggttgacggcaatttcgatgatgcagcttgggcgcagggtcgatgcgacgcaatcgtccgatccggagccgggactgtcgggcgtacacaaatcgcccgcagaagcgcggccgtctggaccgatggctgtgtagaagtcgcgtctgcgttcgaccaggctgcgcgttctcgcggccatagcaaccgacgtacggcgttgcgccctcgccggcagcaagaagccacggaagtccgcccggagcagaaaatgcccacgctactgcgggtttatatagacggtccccacgggatggggaaaaccaccaccacgcaactgctggtggccctgggttcgcgcgacgatatcgtctacgtacccgagccgatgacttactggcgggtgctgggggcttccgagacaatcgcgaacatctacaccacacaacaccgcctcgaccagggtgagatatcggccggggacgcggcggtggtaatgacaagcgcccagataacaatgggcatgccttatgccgtgaccgacgccgttctggctcctcatatcgggggggaggctgggagctcacatgccccgcccccggccctcaccctcatcttcgaccgccatcccatcgccgccctcctgtgctacccggccgcgcggtaccttatgggcagcatgaccccccaggccgtgctggcgttcgtggccctcatcccgccgaccttgcccggcaccaacatcgtgcttggggcccttccggaggacagacacatcgaccgcctggccaaacgccagcgccccggcgagcggctggacctggctatgctggctgcgattcgccgcgtttacgggctacttgccaatacggtgcggtatctgcagtgcggcgggtcgtggcgggaggactggggacagctttcggggacggccgtgccgccccagggtgccgagccccagagcaacgcgggcccacgaccccatatcggggacacgttatttaccctgtttcgggcccccgagttgctggcccccaacggcgacctgtataacgtgtttgcctgggccttggacgtcttggccaaacgcctccgttccatgcacgtctttatcctggattacgaccaatcgcccgccggctgccgggacgccctgctgcaacttacctccgggatggtccagacccacgtcaccacccccggctccataccgacgatatgcgacctggcgcgcacgtttgcccgggagatgggggaggctaactgagctctagcttatcgactcgccgatagtggaaaccgacgccccagcactcgtccgagggcaaaggaatagtcgagaaattgatgatctattaaacaataaagatgtccacatggaagtttttcctgtcatactttgttaagaagggtgagaacagagtacctacattttgaatggaaggattggagctacgggggtgggggtggggtgggattagataaatgcctgctctttactgaaggctctttactattgctttatgataatgtttcatagttggatatcataatttaaacaagcaaaaccaaattaagggccagctcattcctcccactcatgatctatagatcctctagagtcgagggctgcagcagctttcttgtacaaagttggcattataagaaagcattgcttatcaatttgttgcaacgaacaggtcactatcagtcaaaataaaatcattatttgccatccagctgcagctctggcccgtgtctcaaaatctctgatgttacattgcacaagataaaaatatatcatcatgaacaataaaactgtctgcttacataaacagtaatacaaggggtgttatgagccatattcaacgggaaacgtcgaggccgcgattaaattccaacatggatgctgatttatatgggtataaatgggctcgcgataatgtcgggcaatcaggtgcgacaatctatcgcttgtatgggaagcccgatgcgccagagttgtttctgaaacatggcaaaggtagcgttgccaatgatgttacagatgagatggtcagactaaactggctgacggaatttatgcctcttccgaccatcaagcattttatccgtactcctgatgatgcatggttactcaccactgcgatccccggaaaaacagcattccaggtattagaagaatatcctgattcaggtgaaaatattgttgatgcgctggcagtgttcctgcgccggttgcattcgattcctgtttgtaattgtccttttaacagcgatcgcgtatttcgtctcgctcaggcgcaatcacgaatgaataacggtttggttgatgcgagtgattttgatgacgagcgtaatggctggcctgttgaacaagtctggaaagaaatgcataaacttttgccattctcaccggattcagtcgtcactcatggtgatttctcacttgataaccttatttttgacgaggggaaattaataggttgtattgatgttggacgagtcggaatcgcagaccgataccaggatcttgccatcctatggaactgcctcggtgagttttctccttcattacagaaacggctttttcaaaaatatggtattgataatcctgatatgaataaattgcagtttcatttgatgctcgatgagtttttctaatcagaattggttaattggttgtaacactggcagagcattacgctgacttgacgggacggcgcaagctcatgaccaaaatcccttaacgtgagttttcgttccactgagcgtcagaccccgtagaaaagatcaaaggatcttcttgagatcctttttttctgcgcgtaatctgctgcttgcaaacaaaaaaaccaccgctaccagcggtggtttgtttgccggatcaagagctaccaactctttttccgaaggtaactggcttcagcagagcgcagataccaaatactgtccttctagtgtagccgtagttaggccaccacttcaagaactctgtagcaccgcctacatacctcgctctgctaatcctgttaccagtggctgctgccagtggcgataagtcgtgtcttaccgggttggactcaagacgatagttaccggataaggcgcagcggtcgggctgaacggggggttcgtgcacacagcccagcttggagcgaacgacctacaccgaactgagatacctacagcgtgagctatgagaaagcgccacgcttcccgaagggagaaaggcggacaggtatccggtaagcggcagggtcggaacaggagagcgcacgagggagcttccagggggaaacgcctggtatctttatagtcctgtcgggtttcgccacctctgacttgagcgtcgatttttgtgatgctcgtcaggggggcggagcctatggaaaaacgccagcaacgcggcctttttacggttcctggccttttgctggccttttgctcacatgttctttcctgcgttatcccctgattctgtggataaccgtattaccgctagccaggaagagtttgtagaaacgcaaaaaggccatccgtcaggatggccttctgcttagtttgatgcctggcagtttatggcgggcgtcctgcccgccaccc

pENTR-L1-PGK-Neo-L2

atatcatcatgaacaataaaactgtctgcttacataaacagtaatacaaggggtgttatgagccatattcaacgggaaacgtcgaggccgcgattaaattccaacatggatgctgatttatatgggtataaatgggctcgcgataatgtcgggcaatcaggtgcgacaatctatcgcttgtatgggaagcccgatgcgccagagttgtttctgaaacatggcaaaggtagcgttgccaatgatgttacagatgagatggtcagactaaactggctgacggaatttatgcctcttccgaccatcaagcattttatccgtactcctgatgatgcatggttactcaccactgcgatccccggaaaaacagcattccaggtattagaagaatatcctgattcaggtgaaaatattgttgatgcgctggcagtgttcctgcgccggttgcattcgattcctgtttgtaattgtccttttaacagcgatcgcgtatttcgtctcgctcaggcgcaatcacgaatgaataacggtttggttgatgcgagtgattttgatgacgagcgtaatggctggcctgttgaacaagtctggaaagaaatgcataaacttttgccattctcaccggattcagtcgtcactcatggtgatttctcacttgataaccttatttttgacgaggggaaattaataggttgtattgatgttggacgagtcggaatcgcagaccgataccaggatcttgccatcctatggaactgcctcggtgagttttctccttcattacagaaacggctttttcaaaaatatggtattgataatcctgatatgaataaattgcagtttcatttgatgctcgatgagtttttctaatcagaattggttaattggttgtaacactggcagagcattacgctgacttgacgggacggcgcaagctcatgaccaaaatcccttaacgtgagttttcgttccactgagcgtcagaccccgtagaaaagatcaaaggatcttcttgagatcctttttttctgcgcgtaatctgctgcttgcaaacaaaaaaaccaccgctaccagcggtggtttgtttgccggatcaagagctaccaactctttttccgaaggtaactggcttcagcagagcgcagataccaaatactgtccttctagtgtagccgtagttaggccaccacttcaagaactctgtagcaccgcctacatacctcgctctgctaatcctgttaccagtggctgctgccagtggcgataagtcgtgtcttaccgggttggactcaagacgatagttaccggataaggcgcagcggtcgggctgaacggggggttcgtgcacacagcccagcttggagcgaacgacctacaccgaactgagatacctacagcgtgagctatgagaaagcgccacgcttcccgaagggagaaaggcggacaggtatccggtaagcggcagggtcggaacaggagagcgcacgagggagcttccagggggaaacgcctggtatctttatagtcctgtcgggtttcgccacctctgacttgagcgtcgatttttgtgatgctcgtcaggggggcggagcctatggaaaaacgccagcaacgcggcctttttacggttcctggccttttgctggccttttgctcacatgttctttcctgcgttatcccctgattctgtggataaccgtattaccgctagccaggaagagtttgtagaaacgcaaaaaggccatccgtcaggatggccttctgcttagtttgatgcctggcagtttatggcgggcgtcctgcccgccaccctccgggccgttgcttcacaacgttcaaatccgctcccggcggatttgtcctactcaggagagcgttcaccgacaaacaacagataaaacgaaaggcccagtcttccgactgagcctttcgttttatttgatgcctggcagttccctactctcgcgttaacgctagcatggatctcgggccccaaataatgattttattttgactgatagtgacctgttcgttgcaacaaattgatgagcaatgcttttttataatgccaactttgtacaaaaaagcagaattctaccgggtaggggaggcgcttttcccaaggcagtctggagcatgcgctttagcagccccgctgggcacttggcgctacacaagtggcctctggcctcgcacacattccacatccaccggtaggcgccaaccggctccgttctttggtggccccgtcgcgccaccttctactcctcccctagtcaggaagttcccccccgccccgcagctcgcgtcgtgcaggacgtgacaaatggaagtagcacgtctcactagtctcgtgcagatggacagcaccgctgagcaatggaagcgggtaggcctttggggcagcggccaatagcagctttgctccttcgctttctgggctcagaggctgggaaggggtgggtccgggggcgggctcaggggcgggctcaggggcggggcgggcgcccgaaggtcctccggaggcccggcattctgcacgcttcaaaagcgcacgtctgccgcgctgttctcctcttcctcatctccgggcctttcgacctgcagcagcacgtgttgacaattaatcatcggcatagtatatcggcatagtataatacgacaaggtgaggaactaaaccatgggatcggccattgaacaagatggattgcacgcaggttctccggccgcttgggtggagaggctattcggctatgactgggcacaacagacgatcggctgctctgatgccgccgtgttccggctgtcagcgcaggggcgcccggttctttttgtcaagaccgacctgtccggtgccctgaatgaactgcaggacgaggcagcgcggctatcgtggctggccacgacgggcgttccttgcgcagctgtgctcgacgttgtcactgaagcgggaagggactggctgctattgggcgaagtgccggggcaggatctcctgtcatctcaccttgctcctgccgagaaagtatccatcatggctgatgcaatgcggcggctgcatacgcttgatccggctacctgcccattcgaccaccaagcgaaacatcgcatcgagcgagcacgtactcggatggaagccggtcttgtcgatcaggatgatctggacgaagagcatcaggggctcgcgccagccgaactgttcgccaggctcaaggcgcgcatgcccgacggcgaggatctcgtcgtgacccatggcgatgcctgcttgccgaatatcatggtggaaaatggccgcttttctggattcatcgactgtggccggctgggtgtggcggaccgctatcaggacatagcgttggctacccgtgatattgctgaagagcttggcggcgaatgggctgaccgcttcctcgtgctttacggtatcgccgcccccgattcgcagcgcatcgccttctatcgccttcttgacgagttcttctgagcgggactctggggttcgaataaagaccgaccaagcgacgtctgagagctccctggcgaattcggtaccaataaaagagctttattttcatgatctgtgtgttggtttttgtgtgcggcgcgcagctttcttgtacaaagttggcattataagaaagcattgcttatcaatttgttgcaacgaacaggtcactatcagtcaaaataaaatcattatttgccatccagctgcagctctggcccgtgtctcaaaatctctgatgttacattgcacaagataaaaat

pENTR-L1-PGK-TKNeo-L2

gatcgatgggatcggccattgaacaagatggattgcacgcaggttctccggccgcttgggtggagaggctattcggctatgactgggcacaacagacgatcggctgctctgatgccgccgtgttccggctgtcagcgcaggggcgcccggttctttttgtcaagaccgacctgtccggtgccctgaatgaactgcaggacgaggcagcgcggctatcgtggctggccacgacgggcgttccttgcgcagctgtgctcgacgttgtcactgaagcgggaagggactggctgctattgggcgaagtgccggggcaggatctcctgtcatctcaccttgctcctgccgagaaagtatccatcatggctgatgcaatgcggcggctgcatacgcttgatccggctacctgcccattcgaccaccaagcgaaacatcgcatcgagcgagcacgtactcggatggaagccggtcttgtcgatcaggatgatctggacgaagagcatcaggggctcgcgccagccgaactgttcgccaggctcaaggcgcgcatgcccgacggcgaggatctcgtcgtgacccatggcgatgcctgcttgccgaatatcatggtggaaaatggccgcttttctggattcatcgactgtggccggctgggtgtggcggaccgctatcaggacatagcgttggctacccgtgatattgctgaagagcttggcggcgaatgggctgaccgcttcctcgtgctttacggtatcgccgcccccgattcgcagcgcatcgccttctatcgccttcttgacgagttcttctgagcgggagatgggggaggctaactgagctctagcttatcgactcgccgatagtggaaaccgacgccccagcactcgtccgagggcaaaggaatagtcgagaaattgatgatctattaaacaataaagatgtccacatggaagtttttcctgtcatactttgttaagaagggtgagaacagagtacctacattttgaatggaaggattggagctacgggggtgggggtggggtgggattagataaatgcctgctctttactgaaggctctttactattgctttatgataatgtttcatagttggatatcataatttaaacaagcaaaaccaaattaagggccagctcattcctcccactcatgatctatagatcctctagagtcgagggctgcagcagctttcttgtacaaagttggcattataagaaagcattgcttatcaatttgttgcaacgaacaggtcactatcagtcaaaataaaatcattatttgccatccagctgcagctctggcccgtgtctcaaaatctctgatgttacattgcacaagataaaaatatatcatcatgaacaataaaactgtctgcttacataaacagtaatacaaggggtgttatgagccatattcaacgggaaacgtcgaggccgcgattaaattccaacatggatgctgatttatatgggtataaatgggctcgcgataatgtcgggcaatcaggtgcgacaatctatcgcttgtatgggaagcccgatgcgccagagttgtttctgaaacatggcaaaggtagcgttgccaatgatgttacagatgagatggtcagactaaactggctgacggaatttatgcctcttccgaccatcaagcattttatccgtactcctgatgatgcatggttactcaccactgcgatccccggaaaaacagcattccaggtattagaagaatatcctgattcaggtgaaaatattgttgatgcgctggcagtgttcctgcgccggttgcattcgattcctgtttgtaattgtccttttaacagcgatcgcgtatttcgtctcgctcaggcgcaatcacgaatgaataacggtttggttgatgcgagtgattttgatgacgagcgtaatggctggcctgttgaacaagtctggaaagaaatgcataaacttttgccattctcaccggattcagtcgtcactcatggtgatttctcacttgataaccttatttttgacgaggggaaattaataggttgtattgatgttggacgagtcggaatcgcagaccgataccaggatcttgccatcctatggaactgcctcggtgagttttctccttcattacagaaacggctttttcaaaaatatggtattgataatcctgatatgaataaattgcagtttcatttgatgctcgatgagtttttctaatcagaattggttaattggttgtaacactggcagagcattacgctgacttgacgggacggcgcaagctcatgaccaaaatcccttaacgtgagttttcgttccactgagcgtcagaccccgtagaaaagatcaaaggatcttcttgagatcctttttttctgcgcgtaatctgctgcttgcaaacaaaaaaaccaccgctaccagcggtggtttgtttgccggatcaagagctaccaactctttttccgaaggtaactggcttcagcagagcgcagataccaaatactgtccttctagtgtagccgtagttaggccaccacttcaagaactctgtagcaccgcctacatacctcgctctgctaatcctgttaccagtggctgctgccagtggcgataagtcgtgtcttaccgggttggactcaagacgatagttaccggataaggcgcagcggtcgggctgaacggggggttcgtgcacacagcccagcttggagcgaacgacctacaccgaactgagatacctacagcgtgagctatgagaaagcgccacgcttcccgaagggagaaaggcggacaggtatccggtaagcggcagggtcggaacaggagagcgcacgagggagcttccagggggaaacgcctggtatctttatagtcctgtcgggtttcgccacctctgacttgagcgtcgatttttgtgatgctcgtcaggggggcggagcctatggaaaaacgccagcaacgcggcctttttacggttcctggccttttgctggccttttgctcacatgttctttcctgcgttatcccctgattctgtggataaccgtattaccgctagccaggaagagtttgtagaaacgcaaaaaggccatccgtcaggatggccttctgcttagtttgatgcctggcagtttatggcgggcgtcctgcccgccaccctccgggccgttgcttcacaacgttcaaatccgctcccggcggatttgtcctactcaggagagcgttcaccgacaaacaacagataaaacgaaaggcccagtcttccgactgagcctttcgttttatttgatgcctggcagttccctactctcgcgttaacgctagcatggatctcgggccccaaataatgattttattttgactgatagtgacctgttcgttgcaacaaattgatgagcaatgcttttttataatgccaactttgtacaaaaaagcagaattctaccgggtaggggaggcgcttttcccaaggcagtctggagcatgcgctttagcagccccgctgggcacttggcgctacacaagtggcctctggcctcgcacacattccacatccaccggtaggcgccaaccggctccgttctttggtggccccttcgcgccaccttctactcctcccctagtcaggaagttcccccccgccccgcagctcgcgtcgtgcaggacgtgacaaatggaagtagcacgtctcactagtctcgtgcagatggacagcaccgctgagcaatggaagcgggtaggcctttggggcagcggccaatagcagctttgctccttcgctttctgggctcagaggctgggaaggggtgggtccgggggcgggctcaggggcgggctcaggggcggggcgggcgcccgaaggtcctccggaggcccggcattctgcacgcttcaaaagcgcacgtctgccgcgctgttctcctcttcctcatctccgggcctttcgaccgatcatcaagctgatccatgcccacgctactgcgggtttatatagacggtccccacgggatggggaaaaccaccaccacgcaactgctggtggccctgggttcgcgcgacgatatcgtctacgtacccgagccgatgacttactggcgggtgctgggggcttccgagacaatcgcgaacatctacaccacacaacaccgcctcgaccagggtgagatatcggccggggacgcggcggtggtaatgacaagcgcccagataacaatgggcatgccttatgccgtgaccgacgccgttctggctcctcatatcgggggggaggctgggagctcacatgccccgcccccggccctcaccctcatcttcgaccgccatcccatcgccgccctcctgtgctacccggccgcgcggtaccttatgggcagcatgaccccccaggccgtgctggcgttcgtggccctcatcccgccgaccttgcccggcaccaacatcgtgcttggggcccttccggaggacagacacatcgaccgcctggccaaacgccagcgccccggcgagcggctggacctggctatgctggctgcgattcgccgcgtttacgggctacttgccaatacggtgcggtatctgcagtgcggcgggtcgtggcgggaggactggggacagctttcggggacggccgtgccgccccagggtgccgagccccagagcaacgcgggcccacgaccccatatcggggacacgttatttaccctgtttcgggcccccgagttgctggcccccaacggcgacctgtataacgtgtttgcctgggccttggacgtcttggccaaacgcctccgttccatgcacgtctttatcctggattacgaccaatcgcccgccggctgccgggacgccctgctgcaacttacctccgggatggtccagacccacgtcaccacccccggctccataccgacgatatgcgacctggcgcgcacgtttgccc

pENTR-L1-PGK-TKBsd-L2

gatcgatggccaagcctttgtctcaagaagaatccaccctcattgaaagagcaacggctacaatcaacagcatccccatctctgaagactacagcgtcgccagcgcagctctctctagcgacggccgcatcttcactggtgtcaatgtatatcattttactgggggaccttgtgcagaactcgtggtgctgggcactgctgctgctgcggcagctggcaacctgacttgtatcgtcgcgatcggaaatgagaacaggggcatcttgagcccctgcggacggtgccgacaggtgcttctcgatctgcatcctgggatcaaagccatagtgaaggacagtgatggacagccgacggcagttgggattcgtgaattgctgccctctggttatgtgtgggagggctaagggagatgggggaggctaactgagctctagcttatcgactcgccgatagtggaaaccgacgccccagcactcgtccgagggcaaaggaatagtcgagaaattgatgatctattaaacaataaagatgtccacatggaagtttttcctgtcatactttgttaagaagggtgagaacagagtacctacattttgaatggaaggattggagctacgggggtgggggtggggtgggattagataaatgcctgctctttactgaaggctctttactattgctttatgataatgtttcatagttggatatcataatttaaacaagcaaaaccaaattaagggccagctcattcctcccactcatgatctatagatcctctagagtcgagggctgcagcagctttcttgtacaaagttggcattataagaaagcattgcttatcaatttgttgcaacgaacaggtcactatcagtcaaaataaaatcattatttgccatccagctgcagctctggcccgtgtctcaaaatctctgatgttacattgcacaagataaaaatatatcatcatgaacaataaaactgtctgcttacataaacagtaatacaaggggtgttatgagccatattcaacgggaaacgtcgaggccgcgattaaattccaacatggatgctgatttatatgggtataaatgggctcgcgataatgtcgggcaatcaggtgcgacaatctatcgcttgtatgggaagcccgatgcgccagagttgtttctgaaacatggcaaaggtagcgttgccaatgatgttacagatgagatggtcagactaaactggctgacggaatttatgcctcttccgaccatcaagcattttatccgtactcctgatgatgcatggttactcaccactgcgatccccggaaaaacagcattccaggtattagaagaatatcctgattcaggtgaaaatattgttgatgcgctggcagtgttcctgcgccggttgcattcgattcctgtttgtaattgtccttttaacagcgatcgcgtatttcgtctcgctcaggcgcaatcacgaatgaataacggtttggttgatgcgagtgattttgatgacgagcgtaatggctggcctgttgaacaagtctggaaagaaatgcataaacttttgccattctcaccggattcagtcgtcactcatggtgatttctcacttgataaccttatttttgacgaggggaaattaataggttgtattgatgttggacgagtcggaatcgcagaccgataccaggatcttgccatcctatggaactgcctcggtgagttttctccttcattacagaaacggctttttcaaaaatatggtattgataatcctgatatgaataaattgcagtttcatttgatgctcgatgagtttttctaatcagaattggttaattggttgtaacactggcagagcattacgctgacttgacgggacggcgcaagctcatgaccaaaatcccttaacgtgagttttcgttccactgagcgtcagaccccgtagaaaagatcaaaggatcttcttgagatcctttttttctgcgcgtaatctgctgcttgcaaacaaaaaaaccaccgctaccagcggtggtttgtttgccggatcaagagctaccaactctttttccgaaggtaactggcttcagcagagcgcagataccaaatactgtccttctagtgtagccgtagttaggccaccacttcaagaactctgtagcaccgcctacatacctcgctctgctaatcctgttaccagtggctgctgccagtggcgataagtcgtgtcttaccgggttggactcaagacgatagttaccggataaggcgcagcggtcgggctgaacggggggttcgtgcacacagcccagcttggagcgaacgacctacaccgaactgagatacctacagcgtgagctatgagaaagcgccacgcttcccgaagggagaaaggcggacaggtatccggtaagcggcagggtcggaacaggagagcgcacgagggagcttccagggggaaacgcctggtatctttatagtcctgtcgggtttcgccacctctgacttgagcgtcgatttttgtgatgctcgtcaggggggcggagcctatggaaaaacgccagcaacgcggcctttttacggttcctggccttttgctggccttttgctcacatgttctttcctgcgttatcccctgattctgtggataaccgtattaccgctagccaggaagagtttgtagaaacgcaaaaaggccatccgtcaggatggccttctgcttagtttgatgcctggcagtttatggcgggcgtcctgcccgccaccctccgggccgttgcttcacaacgttcaaatccgctcccggcggatttgtcctactcaggagagcgttcaccgacaaacaacagataaaacgaaaggcccagtcttccgactgagcctttcgttttatttgatgcctggcagttccctactctcgcgttaacgctagcatggatctcgggccccaaataatgattttattttgactgatagtgacctgttcgttgcaacaaattgatgagcaatgcttttttataatgccaactttgtacaaaaaagcagaattctaccgggtaggggaggcgcttttcccaaggcagtctggagcatgcgctttagcagccccgctgggcacttggcgctacacaagtggcctctggcctcgcacacattccacatccaccggtaggcgccaaccggctccgttctttggtggccccttcgcgccaccttctactcctcccctagtcaggaagttcccccccgccccgcagctcgcgtcgtgcaggacgtgacaaatggaagtagcacgtctcactagtctcgtgcagatggacagcaccgctgagcaatggaagcgggtaggcctttggggcagcggccaatagcagctttgctccttcgctttctgggctcagaggctgggaaggggtgggtccgggggcgggctcaggggcgggctcaggggcggggcgggcgcccgaaggtcctccggaggcccggcattctgcacgcttcaaaagcgcacgtctgccgcgctgttctcctcttcctcatctccgggcctttcgaccgatcatcaagctgatccatgcccacgctactgcgggtttatatagacggtccccacgggatggggaaaaccaccaccacgcaactgctggtggccctgggttcgcgcgacgatatcgtctacgtacccgagccgatgacttactggcgggtgctgggggcttccgagacaatcgcgaacatctacaccacacaacaccgcctcgaccagggtgagatatcggccggggacgcggcggtggtaatgacaagcgcccagataacaatgggcatgccttatgccgtgaccgacgccgttctggctcctcatatcgggggggaggctgggagctcacatgccccgcccccggccctcaccctcatcttcgaccgccatcccatcgccgccctcctgtgctacccggccgcgcggtaccttatgggcagcatgaccccccaggccgtgctggcgttcgtggccctcatcccgccgaccttgcccggcaccaacatcgtgcttggggcccttccggaggacagacacatcgaccgcctggccaaacgccagcgccccggcgagcggctggacctggctatgctggctgcgattcgccgcgtttacgggctacttgccaatacggtgcggtatctgcagtgcggcgggtcgtggcgggaggactggggacagctttcggggacggccgtgccgccccagggtgccgagccccagagcaacgcgggcccacgaccccatatcggggacacgttatttaccctgtttcgggcccccgagttgctggcccccaacggcgacctgtataacgtgtttgcctgggccttggacgtcttggccaaacgcctccgttccatgcacgtctttatcctggattacgaccaatcgcccgccggctgccgggacgccctgctgcaacttacctccgggatggtccagacccacgtcaccacccccggctccataccgacgatatgcgacctggcgcgcacgtttgccc

pUC-DEST-R3R4(R)

cccagtcacgacgttgtaaaacgacggccagtgccaagcttgcatgcctgcaggtcgggatatctgaattatcaactttgtataataaagttgaacgagaaacgtaaaatgatataaatatcaatatattaaattagattttgcataaaaaacagactacataatactgtaaaacacaacatatccagtcactatggcggccgcattaggcaccccaggctttacactttatgcttccggctcgtataatgtgtggattttgagttaggatccgtcgagattttcaggagctaaggaagctaaaatggagaaaaaaatcactggatataccaccgttgatatatcccaatggcatcgtaaagaacattttgaggcatttcagtcagttgctcaatgtacctataaccagaccgttcagctggatattacggcctttttaaagaccgtaaagaaaaataagcacaagttttatccggcctttattcacattcttgcccgcctgatgaatgctcatccggaattccgtatggcaatgaaagacggtgagctggtgatatgggatagtgttcacccttgttacaccgttttccatgagcaaactgaaacgttttcatcgctctggagtgaataccacgacgatttccggcagtttctacacatatattcgcaagatgtggcgtgttacggtgaaaacctggcctatttccctaaagggtttattgagaatatgtttttcgtctcagccaatccctgggtgagtttcaccagttttgatttaaacgtggccaatatggacaacttcttcgcccccgttttcaccatgggcaaatattatacgcaaggcgacaaggtgctgatgccgctggcgattcaggttcatcatgccgtttgtgatggcttccatgtcggcagaatgcttaatgaattacaacagtactgcgatgagtggcaggcggggcgtaatctagaggatccggcttactaaaagccagataacagtatgcgtatttgcgcgctgatttttgcggtataagaatatatactgatatgtatacccgaagtatgtcaaaaagaggtatgctatgaagcagcgtattacagtgacagttgacagcgacagctatcagttgctcaaggcatatatgatgtcaatatctccggtctggtaagcacaaccatgcagaatgaagcccgtcgtctgcgtgccgaacgctggaaagcggaaaatcaggaagggatggctgaggtcgcccggtttattgaaatgaacggctcttttgctgacgagaacaggggctggtgaaatgcagtttaaggtttacacctataaaagagagagccgttatcgtctgtttgtggatgtacagagtgatattattgacacgcccgggcgacggatggtgatccccctggccagtgcacgtctgctgtcagataaagtctcccgtgaactttacccggtggtgcatatcggggatgaaagctggcgcatgatgaccaccgatatggccagtgtgccggtctccgttatcggggaagaagtggctgatctcagccaccgcgaaaatgacatcaaaaacgccattaacctgatgttctggggaatataaatgtcaggctcccttatacacagccagtctgcaggtcgaccatagtgactggatatgttgtgttttacagtattatgtagtctgttttttatgcaaaatctaatttaatatattgatatttatatcattttacgtttctcgttcaacttttctatacaaagttgatagcttgatatcaatgactctagaggatccccgggtaccgagctcgaattcgtaatcatggtcatagctgtttcctgtgtgaaattgttatccgctcacaattccacacaacatacgagccggaagcataaagtgtaaagcctggggtgcctaatgagtgagctaactcacattaattgcgttgcgctcactgcccgctttccagtcgggaaacctgtcgtgccagctgcattaatgaatcggccaacgcgcggggagaggcggtttgcgtattgggcgctcttccgcttcctcgctcactgactcgctgcgctcggtcgttcggctgcggcgagcggtatcagctcactcaaaggcggtaatacggttatccacagaatcaggggataacgcaggaaagaacatgtgagcaaaaggccagcaaaaggccaggaaccgtaaaaaggccgcgttgctggcgtttttccataggctccgcccccctgacgagcatcacaaaaatcgacgctcaagtcagaggtggcgaaacccgacaggactataaagataccaggcgtttccccctggaagctccctcgtgcgctctcctgttccgaccctgccgcttaccggatacctgtccgcctttctcccttcgggaagcgtggcgctttctcatagctcacgctgtaggtatctcagttcggtgtaggtcgttcgctccaagctgggctgtgtgcacgaaccccccgttcagcccgaccgctgcgccttatccggtaactatcgtcttgagtccaacccggtaagacacgacttatcgccactggcagcagccactggtaacaggattagcagagcgaggtatgtaggcggtgctacagagttcttgaagtggtggcctaactacggctacactagaaggacagtatttggtatctgcgctctgctgaagccagttaccttcggaaaaagagttggtagctcttgatccggcaaacaaaccaccgctggtagcggtggtttttttgtttgcaagcagcagattacgcgcagaaaaaaaggatctcaagaagatcctttgatcttttctacggggtctgacgctcagtggaacgaaaactcacgttaagggattttggtcatgagattatcaaaaaggatcttcacctagatccttttaaattaaaaatgaagttttaaatcaatctaaagtatatatgagtaaacttggtctgacagttaccaatgcttaatcagtgaggcacctatctcagcgatctgtctatttcgttcatccatagttgcctgactccccgtcgtgtagataactacgatacgggagggcttaccatctggccccagtgctgcaatgataccgcgagacccacgctcaccggctccagatttatcagcaataaaccagccagccggaagggccgagcgcagaagtggtcctgcaactttatccgcctccatccagtctattaattgttgccgggaagctagagtaagtagttcgccagttaatagtttgcgcaacgttgttgccattgctacaggcatcgtggtgtcacgctcgtcgtttggtatggcttcattcagctccggttcccaacgatcaaggcgagttacatgatcccccatgttgtgcaaaaaagcggttagctccttcggtcctccgatcgttgtcagaagtaagttggccgcagtgttatcactcatggttatggcagcactgcataattctcttactgtcatgccatccgtaagatgcttttctgtgactggtgagtactcaaccaagtcattctgagaatagtgtatgcggcgaccgagttgctcttgcccggcgtcaatacgggataataccgcgccacatagcagaactttaaaagtgctcatcattggaaaacgttcttcggggcgaaaactctcaaggatcttaccgctgttgagatccagttcgatgtaacccactcgtgcacccaactgatcttcagcatcttttactttcaccagcgtttctgggtgagcaaaaacaggaaggcaaaatgccgcaaaaaagggaataagggcgacacggaaatgttgaatactcatactcttcctttttcaatattattgaagcatttatcagggttattgtctcatgagcggatacatatttgaatgtatttagaaaaataaacaaataggggttccgcgcacatttccccgaaaagtgccacctgacgtctaagaaaccattattatcatgacattaacctataaaaataggcgtatcacgaggccctttcgtctcgcgcgtttcggtgatgacggtgaaaacctctgacacatgcagctcccggagacggtcacagcttgtctgtaagcggatgccgggagcagacaagcccgtcagggcgcgtcagcgggtgttggcgggtgtcggggctggcttaactatgcggcatcagagcagattgtactgagagtgcaccatatgcggtgtgaaataccgcacagatgcgtaaggagaaaataccgcatcaggcgccattcgccattcaggctgcgcaactgttgggaagggcgatcggtgcgggcctcttcgctattacgccagctggcgaaagggggatgtgctgcaaggcgattaagttgggtaacgccagggtttt

pUC-DTA-DEST-R3R4(R)

atcgaattctaccgggtaggggaggcgcttttcccaaggcagtctggagcatgcgctttagcagccccgctgggcacttggcgctacacaagtggcctctggcctcgcacacattccacatccaccggtaggcgccaaccggctccgttctttggtggccccttcgcgccaccttctactcctcccctagtcaggaagttcccccccgccccgcagctcgcgtcgtgcaggacgtgacaaatggaagtagcacgtctcactagtctcgtgcagatggacagcaccgctgagcaatggaagcgggtaggcctttggggcagcggccaatagcagctttgctccttcgctttctgggctcagaggctgggaaggggtgggtccgggggcgggctcaggggcgggctcaggggcggggcgggcgcccgaaggtcctccggaggcccggcattctgcacgcttcaaaagcgcacgtctgccgcgctgttctcctcttcctcatctccgggcctttcgacctgcaggtcctcgccatggatcctgatgatgttgttgattcttctaaatcttttgtgatggaaaacttttcttcgtaccacgggactaaacctggttatgtagattccattcaaaaaggtatacaaaagccaaaatctggtacacaaggaaattatgacgatgattggaaagggttttatagtaccgacaataaatacgacgctgcgggatactctgtagataatgaaaacccgctctctggaaaagctggaggcgtggtcaaagtgacgtatccaggactgacgaaggttctcgcactaaaagtggataatgccgaaactattaagaaagagttaggtttaagtctcactgaaccgttgatggagcaagtcggaacggaagagtttatcaaaaggttcggtgatggtgcttcgcgtgtagtgctcagccttcccttcgctgaggggagttctagcgttgaatatattaataactgggaacaggcgaaagcgttaagcgtagaacttgagattaattttgaaacccgtggaaaacgtggccaagatgcgatgtatgagtatatggctcaagcctgtgcaggaaatcgtgtcaggcgatctctttgtgaaggaaccttacttctgtggtgtgacataattggacaaactacctacagagatttaaagctctaaggtaaatataaaatttttaagtgtataatgtgttaaactactgattctaattgtttgtgtattttagattccaacctatggaactgatgaatgggagcagtggtggaatgcagatcctagagctcgctgatcagcctcgactgtgccttctagttgccagccatctgttgtttgcccctcccccgtgccttccttgaccctggaaggtgccactcccactgtcctttcctaataaaatgaggaaattgcatcgcattgtctgagtaggtgtcattctattctggggggtggggtggggcaggacagcaagggggaggattgggaagacaatagcaggcatgctggggatgcggtgggctctatggcttctgaggcggaaagaaccagctggggctcgacctcgagggggggcccggtaccgagctcgaattcgtaatcatggtcatagctgtttcctgtgtgaaattgttatccgctcacaattccacacaacatacgagccggaagcataaagtgtaaagcctggggtgcctaatgagtgagctaactcacattaattgcgttgcgctcactgcccgctttccagtcgggaaacctgtcgtgccagctgcattaatgaatcggccaacgcgcggggagaggcggtttgcgtattgggcgctcttccgcttcctcgctcactgactcgctgcgctcggtcgttcggctgcggcgagcggtatcagctcactcaaaggcggtaatacggttatccacagaatcaggggataacgcaggaaagaacatgtgagcaaaaggccagcaaaaggccaggaaccgtaaaaaggccgcgttgctggcgtttttccataggctccgcccccctgacgagcatcacaaaaatcgacgctcaagtcagaggtggcgaaacccgacaggactataaagataccaggcgtttccccctggaagctccctcgtgcgctctcctgttccgaccctgccgcttaccggatacctgtccgcctttctcccttcgggaagcgtggcgctttctcatagctcacgctgtaggtatctcagttcggtgtaggtcgttcgctccaagctgggctgtgtgcacgaaccccccgttcagcccgaccgctgcgccttatccggtaactatcgtcttgagtccaacccggtaagacacgacttatcgccactggcagcagccactggtaacaggattagcagagcgaggtatgtaggcggtgctacagagttcttgaagtggtggcctaactacggctacactagaaggacagtatttggtatctgcgctctgctgaagccagttaccttcggaaaaagagttggtagctcttgatccggcaaacaaaccaccgctggtagcggtggtttttttgtttgcaagcagcagattacgcgcagaaaaaaaggatctcaagaagatcctttgatcttttctacggggtctgacgctcagtggaacgaaaactcacgttaagggattttggtcatgagattatcaaaaaggatcttcacctagatccttttaaattaaaaatgaagttttaaatcaatctaaagtatatatgagtaaacttggtctgacagttaccaatgcttaatcagtgaggcacctatctcagcgatctgtctatttcgttcatccatagttgcctgactccccgtcgtgtagataactacgatacgggagggcttaccatctggccccagtgctgcaatgataccgcgagacccacgctcaccggctccagatttatcagcaataaaccagccagccggaagggccgagcgcagaagtggtcctgcaactttatccgcctccatccagtctattaattgttgccgggaagctagagtaagtagttcgccagttaatagtttgcgcaacgttgttgccattgctacaggcatcgtggtgtcacgctcgtcgtttggtatggcttcattcagctccggttcccaacgatcaaggcgagttacatgatcccccatgttgtgcaaaaaagcggttagctccttcggtcctccgatcgttgtcagaagtaagttggccgcagtgttatcactcatggttatggcagcactgcataattctcttactgtcatgccatccgtaagatgcttttctgtgactggtgagtactcaaccaagtcattctgagaatagtgtatgcggcgaccgagttgctcttgcccggcgtcaatacgggataataccgcgccacatagcagaactttaaaagtgctcatcattggaaaacgttcttcggggcgaaaactctcaaggatcttaccgctgttgagatccagttcgatgtaacccactcgtgcacccaactgatcttcagcatcttttactttcaccagcgtttctgggtgagcaaaaacaggaaggcaaaatgccgcaaaaaagggaataagggcgacacggaaatgttgaatactcatactcttcctttttcaatattattgaagcatttatcagggttattgtctcatgagcggatacatatttgaatgtatttagaaaaataaacaaataggggttccgcgcacatttccccgaaaagtgccacctgacgtctaagaaaccattattatcatgacattaacctataaaaataggcgtatcacgaggccctttcgtctcgcgcgtttcggtgatgacggtgaaaacctctgacacatgcagctcccggagacggtcacagcttgtctgtaagcggatgccgggagcagacaagcccgtcagggcgcgtcagcgggtgttggcgggtgtcggggctggcttaactatgcggcatcagagcagattgtactgagagtgcaccatatgcggtgtgaaataccgcacagatgcgtaaggagaaaataccgcatcaggcgccattcgccattcaggctgcgcaactgttgggaagggcgatcggtgcgggcctcttcgctattacgccagctggcgaaagggggatgtgctgcaaggcgattaagttgggtaacgccagggttttcccagtcacgacgttgtaaaacgacggccagtgccaagcttgatatctgaattatcaactttgtataataaagttgaacgagaaacgtaaaatgatataaatatcaatatattaaattagattttgcataaaaaacagactacataatactgtaaaacacaacatatccagtcactatggcggccgcattaggcaccccaggctttacactttatgcttccggctcgtataatgtgtggattttgagttaggatccgtcgagattttcaggagctaaggaagctaaaatggagaaaaaaatcactggatataccaccgttgatatatcccaatggcatcgtaaagaacattttgaggcatttcagtcagttgctcaatgtacctataaccagaccgttcagctggatattacggcctttttaaagaccgtaaagaaaaataagcacaagttttatccggcctttattcacattcttgcccgcctgatgaatgctcatccggaattccgtatggcaatgaaagacggtgagctggtgatatgggatagtgttcacccttgttacaccgttttccatgagcaaactgaaacgttttcatcgctctggagtgaataccacgacgatttccggcagtttctacacatatattcgcaagatgtggcgtgttacggtgaaaacctggcctatttccctaaagggtttattgagaatatgtttttcgtctcagccaatccctgggtgagtttcaccagttttgatttaaacgtggccaatatggacaacttcttcgcccccgttttcaccatgggcaaatattatacgcaaggcgacaaggtgctgatgccgctggcgattcaggttcatcatgccgtttgtgatggcttccatgtcggcagaatgcttaatgaattacaacagtactgcgatgagtggcagggcggggcgtaatctagaggatccggcttactaaaagccagataacagtatgcgtatttgcgcgctgatttttgcggtataagaatatatactgatatgtatacccgaagtatgtcaaaaagaggtatgctatgaagcagcgtattacagtgacagttgacagcgacagctatcagttgctcaaggcatatatgatgtcaatatctccggtctggtaagcacaaccatgcagaatgaagcccgtcgtctgcgtgccgaacgctggaaagcggaaaatcaggaagggatggctgaggtcgcccggtttattgaaatgaacggctcttttgctgacgagaacaggggctggtgaaatgcagtttaaggtttacacctataaaagagagagccgttatcgtctgtttgtggatgtacagagtgatattattgacacgcccgggcgacggatggtgatccccctggccagtgcacgtctgctgtcagataaagtctcccgtgaactttacccggtggtgcatatcggggatgaaagctggcgcatgatgaccaccgatatggccagtgtgccggtctccgttatcggggaagaagtggctgatctcagccaccgcgaaaatgacatcaaaaacgccattaacctgatgttctggggaatataaatgtcaggctcccttatacacagccagtctgcaggtcgaccatagtgactggatatgttgtgttttacagtattatgtagtctgttttttatgcaaaatctaatttaatatattgatatttatatcattttacgtttctcgttcaacttttctatacaaagttgatagcttgat

pATD

ctcatgagacaataaccctgataaatgcttcaataatattgaaaaaggaagagtatgagtattcaacatttccgtgtcgcccttattcccttttttgcggcattttgccttcctgtttttgctcacccagaaacgctggtgaaagtaaaagatgctgaagatcagttgggtgcacgagtgggttacatcgaactggatctcaacagcggtaagatccttgagagttttcgccccgaagaacgttttccaatgatgagcacttttaaagttctgctatgtggcgcggtattatcccgtattgacgccgggcaagagcaactcggtcgccgcatacactattctcagaatgacttggttgagtactcaccagtcacagaaaagcatcttacggatggcatgacagtaagagaattatgcagtgctgccataaccatgagtgataacactgcggccaacttacttctgacaacgatcggaggaccgaaggagctaaccgcttttttgcacaacatgggggatcatgtaactcgccttgatcgttgggaaccggagctgaatgaagccataccaaacgacgagcgtgacaccacgatgcctgtagcaatggcaacaacgttgcgcaaactattaactggcgaactacttactctagcttcccggcaacaattaatagactggatggaggcggataaagttgcaggaccacttctgcgctcggcccttccggctggctggtttattgctgataaatctggagccggtgagcgtgggtctcgcggtatcattgcagcactggggccagatggtaagccctcccgtatcgtagttatctacacgacggggagtcaggcaactatggatgaacgaaatagacagatcgctgagataggtgcctcactgattaagcattggtaactgtcagaccaagtttactcatatatactttagattgatttaaaacttcatttttaatttaaaaggatctaggtgaagatcctttttgataatctcatgaccaaaatcccttaacgtgagttttcgttccactgagcgtcagaccccgtagaaaagatcaaaggatcttcttgagatcctttttttctgcgcgtaatctgctgcttgcaaacaaaaaaaccaccgctaccagcggtggtttgtttgccggatcaagagctaccaactctttttccgaaggtaactggcttcagcagagcgcagataccaaatactgttcttctagtgtagccgtagttaggccaccacttcaagaactctgtagcaccgcctacatacctcgctctgctaatcctgttaccagtggctgctgccagtggcgataagtcgtgtcttaccgggttggactcaagacgatagttaccggataaggcgcagcggtcgggctgaacggggggttcgtgcacacagcccagcttggagcgaacgacctacaccgaactgagatacctacagcgtgagctatgagaaagcgccacgcttcccgaagggagaaaggcggacaggtatccggtaagcggcagggtcggaacaggagagcgcacgagggagcttccagggggaaacgcctggtatctttatagtcctgtcgggtttcgccacctctgacttgagcgtcgatttttgtgatgctcgtcaggggggcggagcctatggaaaaacgccagcaacgcggcctttttacggttcctggccttttgctggccttttgctcaCATGTGCGGCCGCGCCGGCATGcctgcaggtcgggatatctgaattatcaactttgtataataaagttgaacgagaaacgtaaaatgatataaatatcaatatattaaattagattttgcataaaaaacagactacataatactgtaaaacacaacatatccagtcactatggcggccgcattaggcaccccaggctttacactttatgcttccggctcgtataatgtgtggattttgagttaggatccgtcgagattttcaggagctaaggaagctaaaatggagaaaaaaatcactggatataccaccgttgatatatcccaatggcatcgtaaagaacattttgaggcatttcagtcagttgctcaatgtacctataaccagaccgttcagctggatattacggcctttttaaagaccgtaaagaaaaataagcacaagttttatccggcctttattcacattcttgcccgcctgatgaatgctcatccggaattccgtatggcaatgaaagacggtgagctggtgatatgggatagtgttcacccttgttacaccgttttccatgagcaaactgaaacgttttcatcgctctggagtgaataccacgacgatttccggcagtttctacacatatattcgcaagatgtggcgtgttacggtgaaaacctggcctatttccctaaagggtttattgagaatatgtttttcgtctcagccaatccctgggtgagtttcaccagttttgatttaaacgtggccaatatggacaacttcttcgcccccgttttcaccatgggcaaatattatacgcaaggcgacaaggtgctgatgccgctggcgattcaggttcatcatgccgtttgtgatggcttccatgtcggcagaatgcttaatgaattacaacagtactgcgatgagtggcaggcggggcgtaatctagaggatccggcttactaaaagccagataacagtatgcgtatttgcgcgctgatttttgcggtataagaatatatactgatatgtatacccgaagtatgtcaaaaagaggtatgctatgaagcagcgtattacagtgacagttgacagcgacagctatcagttgctcaaggcatatatgatgtcaatatctccggtctggtaagcacaaccatgcagaatgaagcccgtcgtctgcgtgccgaacgctggaaagcggaaaatcaggaagggatggctgaggtcgcccggtttattgaaatgaacggctcttttgctgacgagaacaggggctggtgaaatgcagtttaaggtttacacctataaaagagagagccgttatcgtctgtttgtggatgtacagagtgatattattgacacgcccgggcgacggatggtgatccccctggccagtgcacgtctgctgtcagataaagtctcccgtgaactttacccggtggtgcatatcggggatgaaagctggcgcatgatgaccaccgatatggccagtgtgccggtctccgttatcggggaagaagtggctgatctcagccaccgcgaaaatgacatcaaaaacgccattaacctgatgttctggggaatataaatgtcaggctcccttatacacagccagtctgcaggtcgaccatagtgactggatatgttgtgttttacagtattatgtagtctgttttttatgcaaaatctaatttaatatattgatatttatatcattttacgtttctcgttcaacttttctatacaaagttgatagcttgatatcaatactcTAGAGAAACTCGAGgggtaggggaggcgcttttcccaaggcagtctggagcatgcgctttagcagccccgctgggcacttggcgctacacaagtggcctctggcctcgcacacattccacatccaccggtaggcgccaaccggctccgttctttggtggccccttcgcgccaccttctactcctcccctagtcaggaagttcccccccgccccgcagctcgcgtcgtgcaggacgtgacaaatggaagtagcacgtctcactagtctcgtgcagatggacagcaccgctgagcaatggaagcgggtaggcctttggggcagcggccaatagcagctttgctccttcgctttctgggctcagaggctgggaaggggtgggtccgggggcgggctcaggggcgggctcaggggcggggcgggcgcccgaaggtcctccggaggcccggcattctgcacgcttcaaaagcgcacgtctgccgcgctgttctcctcttcctcatctccgggcctttcgacctgcagcgacccgcttaacagcgtcaacagcgtgccgcagatcttggtggcgtgaaactcccgcacctcttcggcaagcgccttgtagaagcgcgtatggcttcgtacccctgccatcaacacgcgtctgcgttcgaccaggctgcgcgttctcgcggccatagcaaccgacgtacggcgttgcgccctcgccggcagcaagaagccacggaagtccgcctggagcagaaaatgcccacgctactgcgggtttatatagacggtcctcacgggatggggaaaaccaccaccacgcaactgctggtggccctgggttcgcgcgacgatatcgtctacgtacccgagccgatgacttactggcaggtgctgggggcttccgagacaatcgcgaacatctacaccacacaacaccgcctcgaccagggtgagatatcggccggggacgcggcggtggtaatgacaagcgcccagataacaatgggcatgccttatgccgtgaccgacgccgttctggctcctcatatcgggggggaggctgggagctcacatgccccgcccccggccctcaccctcatcttcgaccgccatcccatcgccgccctcctgtgctacccggccgcgcgataccttatgggcagcatgaccccccaggccgtgctggcgttcgtggccctcatcccgccgaccttgcccggcacaaacatcgtgttgggggcccttccggaggacagacacatcgaccgcctggccaaacgccagcgccccggcgagcggcttgacctggctatgctggccgcgattcgccgcgtttacgggctgcttgccaatacggtgcggtatctgcagggcggcgggtcgtggcgggaggattggggacagctttcggggacggccgtgccgccccagggtgccgagccccagagcaacgcgggcccacgaccccatatcggggacacgttatttaccctgtttcgggcccccgagttgctggcccccaacggcgacctgtacaacgtgtttgcctgggccttggacgtcttggccaaacgcctccgtcccatgcacgtctttatcctggattacgaccaatcgcccgccggctgccgggacgccctgctgcaacttacctccgggatggtccagacccacgtcaccacccccggctccataccgacgatctgcgacctggcgcgcacgtttgcccgggagatgggggaggctaactgaaacacggaaggagacaataccggaaggaacccgcgctatgacggcaataaaaagacagaataaaacgcacgggtgttgggtcgtttgttcCCTcAGGtttCCTAGGCACgtaGTGtttTcgcggaacccctatttgtttatttttctaaatacattcaaatatgtatccg

pDEST-Cas9-tdTomato

gagggcctatttcccatgattccttcatatttgcatatacgatacaaggctgttagagagataattggaattaatttgactgtaaacacaaagatattagtacaaaatacgtgacgtagaaagtaataatttcttgggtagtttgcagttttaaaattatgttttaaaatggactatcatatgcttaccgtaacttgaaagtatttcgatttcttggctttatatatcttGTGGAAAGGACGAAACACCggGTCTTCgaGAAGACctgttttagagctaGAAAtagcaagttaaaataaggctagtccgttatcaacttgaaaaagtggcaccgagtcggtgcTTTTTTgttttagagctagaaatagcaagttaaaataaggctagtccgtTTTTagcgcgtgcgccaattctgcagacaaatggctctagaggtacccgttacataacttacggtaaatggcccgcctggctgaccgcccaacgacccccgcccattgacgtcaatagtaacgccaatagggactttccattgacgtcaatgggtggagtatttacggtaaactgcccacttggcagtacatcaagtgtatcatatgccaagtacgccccctattgacgtcaatgacggtaaatggcccgcctggcattGtgcccagtacatgaccttatgggactttcctacttggcagtacatctacgtattagtcatcgctattaccatggtcgaggtgagccccacgttctgcttcactctccccatctcccccccctccccacccccaattttgtatttatttattttttaattattttgtgcagcgatgggggcggggggggggggggggcgcgcgccaggcggggcggggcggggcgaggggcggggcggggcgaggcggagaggtgcggcggcagccaatcagagcggcgcgctccgaaagtttccttttatggcgaggcggcggcggcggcggccctataaaaagcgaagcgcgcggcgggcgggagtcgctgcgacgctgccttcgccccgtgccccgctccgccgccgcctcgcgccgcccgccccggctctgactgaccgcgttactcccacaggtgagcgggcgggacggcccttctcctccgggctgtaattagctgagcaagaggtaagggtttaagggatggttggttggtggggtattaatgtttaattacctggagcacctgcctgaaatcactttttttcaggttGGaccggtgccaccATGGACTATAAGGACCACGACGGAGACTACAAGGATCATGATATTGATTACAAAGACGATGACGATAAGATGGCCCCAAAGAAGAAGCGGAAGGTCGGTATCCACGGAGTCCCAGCAGCCGACAAGAAGTACAGCATCGGCCTGGACATCGGCACCAACTCTGTGGGCTGGGCCGTGATCACCGACGAGTACAAGGTGCCCAGCAAGAAATTCAAGGTGCTGGGCAACACCGACCGGCACAGCATCAAGAAGAACCTGATCGGAGCCCTGCTGTTCGACAGCGGCGAAACAGCCGAGGCCACCCGGCTGAAGAGAACCGCCAGAAGAAGATACACCAGACGGAAGAACCGGATCTGCTATCTGCAAGAGATCTTCAGCAACGAGATGGCCAAGGTGGACGACAGCTTCTTCCACAGACTGGAAGAGTCCTTCCTGGTGGAAGAGGATAAGAAGCACGAGCGGCACCCCATCTTCGGCAACATCGTGGACGAGGTGGCCTACCACGAGAAGTACCCCACCATCTACCACCTGAGAAAGAAACTGGTGGACAGCACCGACAAGGCCGACCTGCGGCTGATCTATCTGGCCCTGGCCCACATGATCAAGTTCCGGGGCCACTTCCTGATCGAGGGCGACCTGAACCCCGACAACAGCGACGTGGACAAGCTGTTCATCCAGCTGGTGCAGACCTACAACCAGCTGTTCGAGGAAAACCCCATCAACGCCAGCGGCGTGGACGCCAAGGCCATCCTGTCTGCCAGACTGAGCAAGAGCAGACGGCTGGAAAATCTGATCGCCCAGCTGCCCGGCGAGAAGAAGAATGGCCTGTTCGGAAACCTGATTGCCCTGAGCCTGGGCCTGACCCCCAACTTCAAGAGCAACTTCGACCTGGCCGAGGATGCCAAACTGCAGCTGAGCAAGGACACCTACGACGACGACCTGGACAACCTGCTGGCCCAGATCGGCGACCAGTACGCCGACCTGTTTCTGGCCGCCAAGAACCTGTCCGACGCCATCCTGCTGAGCGACATCCTGAGAGTGAACACCGAGATCACCAAGGCCCCCCTGAGCGCCTCTATGATCAAGAGATACGACGAGCACCACCAGGACCTGACCCTGCTGAAAGCTCTCGTGCGGCAGCAGCTGCCTGAGAAGTACAAAGAGATTTTCTTCGACCAGAGCAAGAACGGCTACGCCGGCTACATTGACGGCGGAGCCAGCCAGGAAGAGTTCTACAAGTTCATCAAGCCCATCCTGGAAAAGATGGACGGCACCGAGGAACTGCTCGTGAAGCTGAACAGAGAGGACCTGCTGCGGAAGCAGCGGACCTTCGACAACGGCAGCATCCCCCACCAGATCCACCTGGGAGAGCTGCACGCCATTCTGCGGCGGCAGGAAGATTTTTACCCATTCCTGAAGGACAACCGGGAAAAGATCGAGAAGATCCTGACCTTCCGCATCCCCTACTACGTGGGCCCTCTGGCCAGGGGAAACAGCAGATTCGCCTGGATGACCAGAAAGAGCGAGGAAACCATCACCCCCTGGAACTTCGAGGAAGTGGTGGACAAGGGCGCTTCCGCCCAGAGCTTCATCGAGCGGATGACCAACTTCGATAAGAACCTGCCCAACGAGAAGGTGCTGCCCAAGCACAGCCTGCTGTACGAGTACTTCACCGTGTATAACGAGCTGACCAAAGTGAAATACGTGACCGAGGGAATGAGAAAGCCCGCCTTCCTGAGCGGCGAGCAGAAAAAGGCCATCGTGGACCTGCTGTTCAAGACCAACCGGAAAGTGACCGTGAAGCAGCTGAAAGAGGACTACTTCAAGAAAATCGAGTGCTTCGACTCCGTGGAAATCTCCGGCGTGGAAGATCGGTTCAACGCCTCCCTGGGCACATACCACGATCTGCTGAAAATTATCAAGGACAAGGACTTCCTGGACAATGAGGAAAACGAGGACATTCTGGAAGATATCGTGCTGACCCTGACACTGTTTGAGGACAGAGAGATGATCGAGGAACGGCTGAAAACCTATGCCCACCTGTTCGACGACAAAGTGATGAAGCAGCTGAAGCGGCGGAGATACACCGGCTGGGGCAGGCTGAGCCGGAAGCTGATCAACGGCATCCGGGACAAGCAGTCCGGCAAGACAATCCTGGATTTCCTGAAGTCCGACGGCTTCGCCAACAGAAACTTCATGCAGCTGATCCACGACGACAGCCTGACCTTTAAAGAGGACATCCAGAAAGCCCAGGTGTCCGGCCAGGGCGATAGCCTGCACGAGCACATTGCCAATCTGGCCGGCAGCCCCGCCATTAAGAAGGGCATCCTGCAGACAGTGAAGGTGGTGGACGAGCTCGTGAAAGTGATGGGCCGGCACAAGCCCGAGAACATCGTGATCGAAATGGCCAGAGAGAACCAGACCACCCAGAAGGGACAGAAGAACAGCCGCGAGAGAATGAAGCGGATCGAAGAGGGCATCAAAGAGCTGGGCAGCCAGATCCTGAAAGAACACCCCGTGGAAAACACCCAGCTGCAGAACGAGAAGCTGTACCTGTACTACCTGCAGAATGGGCGGGATATGTACGTGGACCAGGAACTGGACATCAACCGGCTGTCCGACTACGATGTGGACCATATCGTGCCTCAGAGCTTTCTGAAGGACGACTCCATCGACAACAAGGTGCTGACCAGAAGCGACAAGAACCGGGGCAAGAGCGACAACGTGCCCTCCGAAGAGGTCGTGAAGAAGATGAAGAACTACTGGCGGCAGCTGCTGAACGCCAAGCTGATTACCCAGAGAAAGTTCGACAATCTGACCAAGGCCGAGAGAGGCGGCCTGAGCGAACTGGATAAGGCCGGCTTCATCAAGAGACAGCTGGTGGAAACCCGGCAGATCACAAAGCACGTGGCACAGATCCTGGACTCCCGGATGAACACTAAGTACGACGAGAATGACAAGCTGATCCGGGAAGTGAAAGTGATCACCCTGAAGTCCAAGCTGGTGTCCGATTTCCGGAAGGATTTCCAGTTTTACAAAGTGCGCGAGATCAACAACTACCACCACGCCCACGACGCCTACCTGAACGCCGTCGTGGGAACCGCCCTGATCAAAAAGTACCCTAAGCTGGAAAGCGAGTTCGTGTACGGCGACTACAAGGTGTACGACGTGCGGAAGATGATCGCCAAGAGCGAGCAGGAAATCGGCAAGGCTACCGCCAAGTACTTCTTCTACAGCAACATCATGAACTTTTTCAAGACCGAGATTACCCTGGCCAACGGCGAGATCCGGAAGCGGCCTCTGATCGAGACAAACGGCGAAACCGGGGAGATCGTGTGGGATAAGGGCCGGGATTTTGCCACCGTGCGGAAAGTGCTGAGCATGCCCCAAGTGAATATCGTGAAAAAGACCGAGGTGCAGACAGGCGGCTTCAGCAAAGAGTCTATCCTGCCCAAGAGGAACAGCGATAAGCTGATCGCCAGAAAGAAGGACTGGGACCCTAAGAAGTACGGCGGCTTCGACAGCCCCACCGTGGCCTATTCTGTGCTGGTGGTGGCCAAAGTGGAAAAGGGCAAGTCCAAGAAACTGAAGAGTGTGAAAGAGCTGCTGGGGATCACCATCATGGAAAGAAGCAGCTTCGAGAAGAATCCCATCGACTTTCTGGAAGCCAAGGGCTACAAAGAAGTGAAAAAGGACCTGATCATCAAGCTGCCTAAGTACTCCCTGTTCGAGCTGGAAAACGGCCGGAAGAGAATGCTGGCCTCTGCCGGCGAACTGCAGAAGGGAAACGAACTGGCCCTGCCCTCCAAATATGTGAACTTCCTGTACCTGGCCAGCCACTATGAGAAGCTGAAGGGCTCCCCCGAGGATAATGAGCAGAAACAGCTGTTTGTGGAACAGCACAAGCACTACCTGGACGAGATCATCGAGCAGATCAGCGAGTTCTCCAAGAGAGTGATCCTGGCCGACGCTAATCTGGACAAAGTGCTGTCCGCCTACAACAAGCACCGGGATAAGCCCATCAGAGAGCAGGCCGAGAATATCATCCACCTGTTTACCCTGACCAATCTGGGAGCCCCTGCCGCCTTCAAGTACTTTGACACCACCATCGACCGGAAGAGGTACACCAGCACCAAAGAGGTGCTGGACGCCACCCTGATCCACCAGAGCATCACCGGCCTGTACGAGACACGGATCGACCTGTCTCAGCTGGGAGGCGACAAAAGGCCGGCGGCCACGAAAAAGGCCGGCCAGGCAAAAAAGAAAAAGgaattcGGCAGTGGAGAGGGCAGAGGAAGTCTGCTAACATGCGGTGACGTCGAGGAGAATCCTGGCCCAGTGAGCAAGGGCGAGGAGGTCATCAAAGAGTTCATGCGCTTCAAGGTGCGCATGGAGGGCTCCATGAACGGCCACGAGTTCGAGATCGAGGGCGAGGGCGAGGGCCGCCCCTACGAGGGCACCCAGACCGCCAAGCTGAAGGTGACCAAGGGCGGCCCCCTGCCCTTCGCCTGGGACATCCTGTCCCCCCAGTTCATGTACGGCTCCAAGGCGTACGTGAAGCACCCCGCCGACATCCCCGATTACAAGAAGCTGTCCTTCCCCGAGGGCTTCAAGTGGGAGCGCGTGATGAACTTCGAGGACGGCGGTCTGGTGACCGTGACCCAGGACTCCTCCCTGCAGGACGGCACGCTGATCTACAAGGTGAAGATGCGCGGCACCAACTTCCCCCCCGACGGCCCCGTAATGCAGAAaAAGACCATGGGCTGGGAGGCCTCCACCGAGCGCCTGTACCCCCGCGACGGCGTGCTGAAGGGCGAGATCCACCAGGCCCTGAAGCTGAAGGACGGCGGCCACTACCTGGTGGAGTTCAAGACCATCTACATGGCCAAGAAGCCCGTGCAACTGCCCGGCTACTACTACGTGGACACCAAGCTGGACATCACCTCCCACAACGAGGACTACACCATCGTGGAACAGTACGAGCGCTCCGAGGGCCGCCACCACCTGTTCCTGGGGCATGGCACCGGCAGCACCGGCAGCGGCAGCTCCGGCACCGCCTCCTCCGAGGACAACAACATGGCCGTCATCAAAGAGTTCATGCGCTTCAAGGTGCGCATGGAGGGCTCCATGAACGGCCACGAGTTCGAGATCGAGGGCGAGGGCGAGGGCCGCCCCTACGAGGGCACCCAGACCGCCAAGCTGAAGGTGACCAAGGGCGGCCCCCTGCCCTTCGCCTGGGACATCCTGTCCCCCCAGTTCATGTACGGCTCCAAGGCGTACGTGAAGCACCCCGCCGACATCCCCGATTACAAGAAGCTGTCCTTCCCCGAGGGCTTCAAGTGGGAGCGCGTGATGAACTTCGAGGACGGCGGTCTGGTGACCGTGACCCAGGACTCCTCCCTGCAGGACGGCACGCTGATCTACAAGGTGAAGATGCGCGGCACCAACTTCCCCCCCGACGGCCCCGTAATGCAGAAaAAGACCATGGGCTGGGAGGCCTCCACCGAGCGCCTGTACCCCCGCGACGGCGTGCTGAAGGGCGAGATCCACCAGGCCCTGAAGCTGAAGGACGGCGGCCACTACCTGGTGGAGTTCAAGACCATCTACATGGCCAAGAAGCCCGTGCAACTGCCCGGCTACTACTACGTGGACACCAAGCTGGACATCACCTCCCACAACGAGGACTACACCATCGTGGAACAGTACGAGCGCTCCGAGGGCCGCCACCACCTGTTCCTGTACGGCATGGACGAGCTGTACAAGgaattctaaCTAGAGCTCGCTGATCAGCCTCGACTGTGCCTTCTAGTTGCCAGCCATCTGTTGTTTGCCCCTCCCCCGTGCCTTCCTTGACCCTGGAAGGTGCCACTCCCACTGTCCTTTCCTAATAAAATGAGGAAATTGCATCGCATTGTCTGAGTAGGTGTCATTCTATTCTGGGGGGTGGGGTGGGGCAGGACAGCAAGGGGGAGGATTGGGAAGAgAATAGCAGGCATGCTGGGGAgcgtaaaacgacggccagtgccaagcttgcatgcctgcaggtcgggatatctgaattatcaactttgtataataaagttgaacgagaaacgtaaaatgatataaatatcaatatattaaattagattttgcataaaaaacagactacataatactgtaaaacacaacatatccagtcactatggcggccgcattaggcaccccaggctttacactttatgcttccggctcgtataatgtgtggattttgagttaggatccgtcgagattttcaggagctaaggaagctaaaatggagaaaaaaatcactggatataccaccgttgatatatcccaatggcatcgtaaagaacattttgaggcatttcagtcagttgctcaatgtacctataaccagaccgttcagctggatattacggcctttttaaagaccgtaaagaaaaataagcacaagttttatccggcctttattcacattcttgcccgcctgatgaatgctcatccggaattccgtatggcaatgaaagacggtgagctggtgatatgggatagtgttcacccttgttacaccgttttccatgagcaaactgaaacgttttcatcgctctggagtgaataccacgacgatttccggcagtttctacacatatattcgcaagatgtggcgtgttacggtgaaaacctggcctatttccctaaagggtttattgagaatatgtttttcgtctcagccaatccctgggtgagtttcaccagttttgatttaaacgtggccaatatggacaacttcttcgcccccgttttcaccatgggcaaatattatacgcaaggcgacaaggtgctgatgccgctggcgattcaggttcatcatgccgtttgtgatggcttccatgtcggcagaatgcttaatgaattacaacagtactgcgatgagtggcaggcggggcgtaatctagaggatccggcttactaaaagccagataacagtatgcgtatttgcgcgctgatttttgcggtataagaatatatactgatatgtatacccgaagtatgtcaaaaagaggtatgctatgaagcagcgtattacagtgacagttgacagcgacagctatcagttgctcaaggcatatatgatgtcaatatctccggtctggtaagcacaaccatgcagaatgaagcccgtcgtctgcgtgccgaacgctggaaagcggaaaatcaggaagggatggctgaggtcgcccggtttattgaaatgaacggctcttttgctgacgagaacaggggctggtgaaatgcagtttaaggtttacacctataaaagagagagccgttatcgtctgtttgtggatgtacagagtgatattattgacacgcccgggcgacggatggtgatccccctggccagtgcacgtctgctgtcagataaagtctcccgtgaactttacccggtggtgcatatcggggatgaaagctggcgcatgatgaccaccgatatggccagtgtgccggtctccgttatcggggaagaagtggctgatctcagccaccgcgaaaatgacatcaaaaacgccattaacctgatgttctggggaatataaatgtcaggctcccttatacacagccagtctgcaggtcgaccatagtgactggatatgttgtgttttacagtattatgtagtctgttttttatgcaaaatctaatttaatatattgatatttatatcattttacgtttctcgttcaacttttctatacaaagttgatagcttgatatcaatgactctagaggatccccgggtaccgagctcgaattcgtaatcatggtcatagctgtttcctggcctgatgcggtattttctccttacgcatctgtgcggtatttcacaccgcatacgtcaaagcaaccatagtacgcgccctgtagcggcgcattaagcgcggcgggtgtggtggttacgcgcagcgtgaccgctacacttgccagcgccctagcgcccgctcctttcgctttcttcccttcctttctcgccacgttcgccggctttccccgtcaagctctaaatcgggggctccctttagggttccgatttagtgctttacggcacctcgaccccaaaaaacttgatttgggtgatggttcacgtagtgggccatcgccctgatagacggtttttcgccctttgacgttggagtccacgttctttaatagtggactcttgttccaaactggaacaacactcaaccctatctcgggctattcttttgatttataagggattttgccgatttcggcctattggttaaaaaatgagctgatttaacaaaaatttaacgcgaattttaacaaaatattaacgtttacaattttatggtgcactctcagtacaatctgctctgatgccgcatagttaagccagccccgacacccgccaacacccgctgacgcgccctgacgggcttgtctgctcccggcatccgcttacagacaagctgtgaccgtctccgggagctgcatgtgtcagaggttttcaccgtcatcaccgaaacgcgcgagacgaaagggcctcgtgatacgcctatttttataggttaatgtcatgataataatggtttcttagacgtcaggtggcacttttcggggaaatgtgcgcggaacccctatttgtttatttttctaaatacattcaaatatgtatccgctcatgagacaataaccctgataaatgcttcaataatattgaaaaaggaagagtatgagtattcaacatttccgtgtcgcccttattcccttttttgcggcattttgccttcctgtttttgctcacccagaaacgctggtgaaagtaaaagatgctgaagatcagttgggtgcacgagtgggttacatcgaactggatctcaacagcggtaagatccttgagagttttcgccccgaagaacgttttccaatgatgagcacttttaaagttctgctatgtggcgcggtattatcccgtattgacgccgggcaagagcaactcggtcgccgcatacactattctcagaatgacttggttgagtactcaccagtcacagaaaagcatcttacggatggcatgacagtaagagaattatgcagtgctgccataaccatgagtgataacactgcggccaacttacttctgacaacgatcggaggaccgaaggagctaaccgcttttttgcacaacatgggggatcatgtaactcgccttgatcgttgggaaccggagctgaatgaagccataccaaacgacgagcgtgacaccacgatgcctgtagcaatggcaacaacgttgcgcaaactattaactggcgaactacttactctagcttcccggcaacaattaatagactggatggaggcggataaagttgcaggaccacttctgcgctcggcccttccggctggctggtttattgctgataaatctggagccggtgagcgtggaagccgcggtatcattgcagcactggggccagatggtaagccctcccgtatcgtagttatctacacgacggggagtcaggcaactatggatgaacgaaatagacagatcgctgagataggtgcctcactgattaagcattggtaactgtcagaccaagtttactcatatatactttagattgatttaaaacttcatttttaatttaaaaggatctaggtgaagatcctttttgataatctcatgaccaaaatcccttaacgtgagttttcgttccactgagcgtcagaccccgtagaaaagatcaaaggatcttcttgagatcctttttttctgcgcgtaatctgctgcttgcaaacaaaaaaaccaccgctaccagcggtggtttgtttgccggatcaagagctaccaactctttttccgaaggtaactggcttcagcagagcgcagataccaaatactgtccttctagtgtagccgtagttaggccaccacttcaagaactctgtagcaccgcctacatacctcgctctgctaatcctgttaccagtggctgctgccagtggcgataagtcgtgtcttaccgggttggactcaagacgatagttaccggataaggcgcagcggtcgggctgaacggggggttcgtgcacacagcccagcttggagcgaacgacctacaccgaactgagatacctacagcgtgagctatgagaaagcgccacgcttcccgaagggagaaaggcggacaggtatccggtaagcggcagggtcggaacaggagagcgcacgagggagcttccagggggaaacgcctggtatctttatagtcctgtcgggtttcgccacctctgacttgagcgtcgatttttgtgatgctcgtcaggggggcggagcctatggaaaaacgccagcaacgcggcctttttacggttcctggccttttgctggccttttgctcacatgt

pDONR-P3P1r

ccaaataatgattttattttgactgatagtgacctgttcgttgcaacaaattgatgagcaatgcttttttataatgccaactttgtataataaagttgaacgagaaacgtaaaatgatataaatatcaatatattaaattagattttgcataaaaaacagactacataatactgtaaaacacaacatatccagtcactatgaatcaactacttagatggtattagtgacctgtagtcgaccgacagccttccaaatgttcttcgggtgatgctgccaacttagtcgaccgacagccttccaaatgttcttctcaaacggaatcgtcgtatccagcctactcgctattgtcctcaatgccgtattaaatcataaaaagaaataagaaaaagaggtgcgagcctcttttttgtgtgacaaaataaaaacatctacctattcatatacgctagtgtcatagtcctgaaaatcatctgcatcaagaacaatttcacaactcttatacttttctcttacaagtcgttcggcttcatctggattttcagcctctatacttactaaacgtgataaagtttctgtaatttctactgtatcgacctgcagactggctgtgtataagggagcctgacatttatattccccagaacatcaggttaatggcgtttttgatgtcattttcgcggtggctgagatcagccacttcttccccgataacggagaccggcacactggccatatcggtggtcatcatgcgccagctttcatccccgatatgcaccaccgggtaaagttcacgggagactttatctgacagcagacgtgcactggccagggggatcaccatccgtcgcccgggcgtgtcaataatatcactctgtacatccacaaacagacgataacggctctctcttttataggtgtaaaccttaaactgcatttcaccagtccctgttctcgtcagcaaaagagccgttcatttcaataaaccgggcgacctcagccatcccttcctgattttccgctttccagcgttcggcacgcagacgacgggcttcattctgcatggttgtgcttaccagaccggagatattgacatcatatatgccttgagcaactgatagctgtcgctgtcaactgtcactgtaatacgctgcttcatagcacacctctttttgacatacttcgggtatacatatcagtatatattcttataccgcaaaaatcagcgcgcaaatacgcatactgttatctggcttttagtaagccggatccacgcgattacgccccgccctgccactcatcgcagtactgttgtaattcattaagcattctgccgacatggaagccatcacagacggcatgatgaacctgaatcgccagcggcatcagcaccttgtcgccttgcgtataatatttgcccatggtgaaaacgggggcgaagaagttgtccatattggccacgtttaaatcaaaactggtgaaactcacccagggattggctgagacgaaaaacatattctcaataaaccctttagggaaataggccaggttttcaccgtaacacgccacatcttgcgaatatatgtgtagaaactgccggaaatcgtcgtggtattcactccagagcgatgaaaacgtttcagtttgctcatggaaaacggtgtaacaagggtgaacactatcccatatcaccagctcaccgtctttcattgccatacggaattccggatgagcattcatcaggcgggcaagaatgtgaataaaggccggataaaacttgtgcttatttttctttacggtctttaaaaaggccgtaatatccagctgaacggtctggttataggtacattgagcaactgactgaaatgcctcaaaatgttctttacgatgccattgggatatatcaacggtggtatatccagtgatttttttctccattttagcttccttagctcctgaaaatctcgataactcaaaaaatacgcccggtagtgatcttatttcattatggtgaaagttggaacctcttacgtgccgatcaacgtctcattttcgccaaaagttggcccagggcttcccggtatcaacagggacaccaggatttatttattctgcgaagtgatcttccgtcacaggtatttattcggcgcaaagtgcgtcgggtgatgctgccaacttagtcgacgctctagagctccaaataatgattttattttgactgatagtgacctgttcgttgcaacaaattgataagcaatgcttttttataatgccaactttgtacaaaaaagttgaacgagaaacgtaaaatgatataaatatcaatatattaaattagattttgcataaaaaacagactacataatactgtaaaacacaacatatgcagtcactatgaatcaactacttagatggtattagtgacctgtagaattcgagctctagagctgcagggcggccgcgatatcccctatagtgagtcgtattacatggtcatagctgtttcctggcagctctggcccgtgtctcaaaatctctgatgttacattgcacaagataaaaatatatcatcatgaacaataaaactgtctgcttacataaacagtaatacaaggggtgttatgagccatattcaacgggaaacgtcgaggccgcgattaaattccaacatggatgctgatttatatgggtataaatgggctcgcgataatgtcgggcaatcaggtgcgacaatctatcgcttgtatgggaagcccgatgcgccagagttgtttctgaaacatggcaaaggtagcgttgccaatgatgttacagatgagatggtcagactaaactggctgacggaatttatgcctcttccgaccatcaagcattttatccgtactcctgatgatgcatggttactcaccactgcgatccccggaaaaacagcattccaggtattagaagaatatcctgattcaggtgaaaatattgttgatgcgctggcagtgttcctgcgccggttgcattcgattcctgtttgtaattgtccttttaacagcgatcgcgtatttcgtctcgctcaggcgcaatcacgaatgaataacggtttggttgatgcgagtgattttgatgacgagcgtaatggctggcctgttgaacaagtctggaaagaaatgcataaacttttgccattctcaccggattcagtcgtcactcatggtgatttctcacttgataaccttatttttgacgaggggaaattaataggttgtattgatgttggacgagtcggaatcgcagaccgataccaggatcttgccatcctatggaactgcctcggtgagttttctccttcattacagaaacggctttttcaaaaatatggtattgataatcctgatatgaataaattgcagtttcatttgatgctcgatgagtttttctaatcagaattggttaattggttgtaacactggcagagcattacgctgacttgacgggacggcgcaagctcatgaccaaaatcccttaacgtgagttacgcgtcgttccactgagcgtcagaccccgtagaaaagatcaaaggatcttcttgagatcctttttttctgcgcgtaatctgctgcttgcaaacaaaaaaaccaccgctaccagcggtggtttgtttgccggatcaagagctaccaactctttttccgaaggtaactggcttcagcagagcgcagataccaaatactgtccttctagtgtagccgtagttaggccaccacttcaagaactctgtagcaccgcctacatacctcgctctgctaatcctgttaccagtggctgctgccagtggcgataagtcgtgtcttaccgggttggactcaagacgatagttaccggataaggcgcagcggtcgggctgaacggggggttcgtgcacacagcccagcttggagcgaacgacctacaccgaactgagatacctacagcgtgagcattgagaaagcgccacgcttcccgaagggagaaaggcggacaggtatccggtaagcggcagggtcggaacaggagagcgcacgagggagcttccagggggaaacgcctggtatctttatagtcctgtcgggtttcgccacctctgacttgagcgtcgatttttgtgatgctcgtcaggggggcggagcctatggaaaaacgccagcaacgcggcctttttacggttcctggccttttgctggccttttgctcacatgttctttcctgcgttatcccctgattctgtggataaccgtattaccgcctttgagtgagctgataccgctcgccgcagccgaacgaccgagcgcagcgagtcagtgagcgaggaagcggaagagcgcccaatacgcaaaccgcctctccccgcgcgttggccgattcattaatgcagctggcacgacaggtttcccgactggaaagcgggcagtgagcgcaacgcaattaatacgcgtaccgctagccaggaagagtttgtagaaacgcaaaaaggccatccgtcaggatggccttctgcttagtttgatgcctggcagtttatggcgggcgtcctgcccgccaccctccgggccgttgcttcacaacgttcaaatccgctcccggcggatttgtcctactcaggagagcgttcaccgacaaacaacagataaaacgaaaggcccagtcttccgactgagcctttcgttttatttgatgcctggcagttccctactctcgcgttaacgctagcatggatgttttcccagtcacgacgttgtaaaacgacggccagtcttaagctcgggcc

pDONR-P2rP4

ctgcagctctagagctcgaattctacaggtcactaataccatctaagtagttggttcatagtgactgcatatgttgtgttttacagtattatgtagtctgttttttatgcaaaatctaatttaatatattgatatttatatcattttacgtttctcgttcaactttcttgtacaaagttggcattataaaaaagcattgcttatcaatttgttgcaacgaacaggtcactatcagtcaaaataaaatcattatttggagctctagagcgtcgaccgacagccttccaaatgttcttctcaaacggaatcgtcgtatccagcctactcgctattgtcctcaatgccgtattaaatcataaaaagaaataagaaaaagaggtgcgagcctcttttttgtgtgacaaaataaaaacatctacctattcatatacgctagtgtcatagtcctgaaaatcatctgcatcaagaacaatttcacaactcttatacttttctcttacaagtcgttcggcttcatctggattttcagcctctatacttactaaacgtgataaagtttctgtaatttctactgtatcgacctgcagactggctgtgtataagggagcctgacatttatattccccagaacatcaggttaatggcgtttttgatgtcattttcgcggtggctgagatcagccacttcttccccgataacggagaccggcacactggccatatcggtggtcatcatgcgccagctttcatccccgatatgcaccaccgggtaaagttcacgggagactttatctgacagcagacgtgcactggccagggggatcaccatccgtcgcccgggcgtgtcaataatatcactctgtacatccacaaacagacgataacggctctctcttttataggtgtaaaccttaaactgcatttcaccagtccctgttctcgtcagcaaaagagccgttcatttcaataaaccgggcgacctcagccatcccttcctgattttccgctttccagcgttcggcacgcagacgacgggcttcattctgcatggttgtgcttaccagaccggagatattgacatcatatatgccttgagcaactgatagctgtcgctgtcaactgtcactgtaatacgctgcttcatagcacacctctttttgacatacttcgggtatacatatcagtatatattcttataccgcaaaaatcagcgcgcaaatacgcatactgttatctggcttttagtaagccggatccacgcgattacgccccgccctgccactcatcgcagtactgttgtaattcattaagcattctgccgacatggaagccatcacagacggcatgatgaacctgaatcgccagcggcatcagcaccttgtcgccttgcgtataatatttgcccatggtgaaaacgggggcgaagaagttgtccatattggccacgtttaaatcaaaactggtgaaactcacccagggattggctgagacgaaaaacatattctcaataaaccctttagggaaataggccaggttttcaccgtaacacgccacatcttgcgaatatatgtgtagaaactgccggaaatcgtcgtggtattcactccagagcgatgaaaacgtttcagtttgctcatggaaaacggtgtaacaagggtgaacactatcccatatcaccagctcaccgtctttcattgccatacggaattccggatgagcattcatcaggcgggcaagaatgtgaataaaggccggataaaacttgtgcttatttttctttacggtctttaaaaaggccgtaatatccagctgaacggtctggttataggtacattgagcaactgactgaaatgcctcaaaatgttctttacgatgccattgggatatatcaacggtggtatatccagtgatttttttctccattttagcttccttagctcctgaaaatctcgataactcaaaaaatacgcccggtagtgatcttatttcattatggtgaaagttggaacctcttacgtgccgatcaacgtctcattttcgccaaaagttggcccagggcttcccggtatcaacagggacaccaggatttatttattctgcgaagtgatcttccgtcacaggtatttattcggcgcaaagtgcgtcgggtgatgctgccaacttagtcgactacaggtcactaataccatctaagtagttgattcatagtgactggatatgttgtgttttacagtattatgtagtctgttttttatgcaaaatctaatttaatatattgatatttatatcattttacgtttctcgttcaacttttctatacaaagttggcattataagaaagcattgcttatcaatttgttgcaacgaacaggtcactatcagtcaaaataaaatcattatttgccatccagctgcagctctggcccgtgtctcaaaatctctgatgttacattgcacaagataaaaatatatcatcatgaacaataaaactgtctgcttacataaacagtaatacaaggggtgttatgagccatattcaacgggaaacgtcgaggccgcgattaaattccaacatggatgctgatttatatgggtataaatgggctcgcgataatgtcgggcaatcaggtgcgacaatctatcgcttgtatgggaagcccgatgcgccagagttgtttctgaaacatggcaaaggtagcgttgccaatgatgttacagatgagatggtcagactaaactggctgacggaatttatgcctcttccgaccatcaagcattttatccgtactcctgatgatgcatggttactcaccactgcgatccccggaaaaacagcattccaggtattagaagaatatcctgattcaggtgaaaatattgttgatgcgctggcagtgttcctgcgccggttgcattcgattcctgtttgtaattgtccttttaacagcgatcgcgtatttcgtctcgctcaggcgcaatcacgaatgaataacggtttggttgatgcgagtgattttgatgacgagcgtaatggctggcctgttgaacaagtctggaaagaaatgcataaacttttgccattctcaccggattcagtcgtcactcatggtgatttctcacttgataaccttatttttgacgaggggaaattaataggttgtattgatgttggacgagtcggaatcgcagaccgataccaggatcttgccatcctatggaactgcctcggtgagttttctccttcattacagaaacggctttttcaaaaatatggtattgataatcctgatatgaataaattgcagtttcatttgatgctcgatgagtttttctaatcagaattggttaattggttgtaacactggcagagcattacgctgacttgacgggacggcgcaagctcatgaccaaaatcccttaacgtgagttttcgttccactgagcgtcagaccccgtagaaaagatcaaaggatcttcttgagatcctttttttctgcgcgtaatctgctgcttgcaaacaaaaaaaccaccgctaccagcggtggtttgtttgccggatcaagagctaccaactctttttccgaaggtaactggcttcagcagagcgcagataccaaatactgtccttctagtgtagccgtagttaggccaccacttcaagaactctgtagcaccgcctacatacctcgctctgctaatcctgttaccagtggctgctgccagtggcgataagtcgtgtcttaccgggttggactcaagacgatagttaccggataaggcgcagcggtcgggctgaacggggggttcgtgcacacagcccagcttggagcgaacgacctacaccgaactgagatacctacagcgtgagctatgagaaagcgccacgcttcccgaagggagaaaggcggacaggtatccggtaagcggcagggtcggaacaggagagcgcacgagggagcttccagggggaaacgcctggtatctttatagtcctgtcgggtttcgccacctctgacttgagcgtcgatttttgtgatgctcgtcaggggggcggagcctatggaaaaacgccagcaacgcggcctttttacggttcctggccttttgctggccttttgctcacatgttctttcctgcgttatcccctgattctgtggataaccgtattaccgctagccaggaagagtttgtagaaacgcaaaaaggccatccgtcaggatggccttctgcttagtttgatgcctggcagtttatggcgggcgtcctgcccgccaccctccgggccgttgcttcacaacgttcaaatccgctcccggcggatttgtcctactcaggagagcgttcaccgacaaacaacagataaaacgaaaggcccagtcttccgactgagcctttcgttttatttgatgcctggcagttccctactctcgcgttaacgctagcatggatctcgggcc

pENTR2-L3-PBL-R1

gtaaaacgacggccagtcttaagctcgggccccaaataatgattttattttgactgatagtgacctgttcgttgcaacaaattgatgagcaatgcttttttataatgccaactttgtataataaagttgttaaccctagaaagataatcatattgtgacgtacgttaaagataatcatgcgtaaaattgacgcatgtgttttatcggtctgtatatcgaggtcccgtgaggcgtttgaatagatattaagttttattatatttacacttacatactaataataaattcaacaaacaattcttttacgtgacttttaagatttaactcaaacaaaaactcaaaatttcttctataaagtaacaaaactcaagtttgtacaaaaaagttgaacgagaaacgtaaaatgatataaatatcaatatattaaattagattttgcataaaaaacagactacataatactgtaaaacacaacatatgcagtcactatgaatcaactacttagatggtattagtgacctgtagaattcgagctctagagctgcagggcggccgcgatatcccctatagtgagtcgtattacatggtcatagctgtttcctggcagctctggcccgtgtctcaaaatctctgatgttacattgcacaagataaaaatatatcatcatgaacaataaaactgtctgcttacataaacagtaatacaaggggtgttatgagccatattcaacgggaaacgtcgaggccgcgattaaattccaacatggatgctgatttatatgggtataaatgggctcgcgataatgtcgggcaatcaggtgcgacaatctatcgcttgtatgggaagcccgatgcgccagagttgtttctgaaacatggcaaaggtagcgttgccaatgatgttacagatgagatggtcagactaaactggctgacggaatttatgcctcttccgaccatcaagcattttatccgtactcctgatgatgcatggttactcaccactgcgatccccggaaaaacagcattccaggtattagaagaatatcctgattcaggtgaaaatattgttgatgcgctggcagtgttcctgcgccggttgcattcgattcctgtttgtaattgtccttttaacagcgatcgcgtatttcgtctcgctcaggcgcaatcacgaatgaataacggtttggttgatgcgagtgattttgatgacgagcgtaatggctggcctgttgaacaagtctggaaagaaatgcataaacttttgccattctcaccggattcagtcgtcactcatggtgatttctcacttgataaccttatttttgacgaggggaaattaataggttgtattgatgttggacgagtcggaatcgcagaccgataccaggatcttgccatcctatggaactgcctcggtgagttttctccttcattacagaaacggctttttcaaaaatatggtattgataatcctgatatgaataaattgcagtttcatttgatgctcgatgagtttttctaatcagaattggttaattggttgtaacactggcagagcattacgctgacttgacgggacggcgcaagctcatgaccaaaatcccttaacgtgagttacgcgtcgttccactgagcgtcagaccccgtagaaaagatcaaaggatcttcttgagatcctttttttctgcgcgtaatctgctgcttgcaaacaaaaaaaccaccgctaccagcggtggtttgtttgccggatcaagagctaccaactctttttccgaaggtaactggcttcagcagagcgcagataccaaatactgtccttctagtgtagccgtagttaggccaccacttcaagaactctgtagcaccgcctacatacctcgctctgctaatcctgttaccagtggctgctgccagtggcgataagtcgtgtcttaccgggttggactcaagacgatagttaccggataaggcgcagcggtcgggctgaacggggggttcgtgcacacagcccagcttggagcgaacgacctacaccgaactgagatacctacagcgtgagcattgagaaagcgccacgcttcccgaagggagaaaggcggacaggtatccggtaagcggcagggtcggaacaggagagcgcacgagggagcttccagggggaaacgcctggtatctttatagtcctgtcgggtttcgccacctctgacttgagcgtcgatttttgtgatgctcgtcaggggggcggagcctatggaaaaacgccagcaacgcggcctttttacggttcctggccttttgctggccttttgctcacatgttctttcctgcgttatcccctgattctgtggataaccgtattaccgcctttgagtgagctgataccgctcgccgcagccgaacgaccgagcgcagcgagtcagtgagcgaggaagcggaagagcgcccaatacgcaaaccgcctctccccgcgcgttggccgattcattaatgcagctggcacgacaggtttcccgactggaaagcgggcagtgagcgcaacgcaattaatacgcgtaccgctagccaggaagagtttgtagaaacgcaaaaaggccatccgtcaggatggccttctgcttagtttgatgcctggcagtttatggcgggcgtcctgcccgccaccctccgggccgttgcttcacaacgttcaaatccgctcccggcggatttgtcctactcaggagagcgttcaccgacaaacaacagataaaacgaaaggcccagtcttccgactgagcctttcgttttatttgatgcctggcagttccctactctcgcgtcaacgctagcatggatgttttcccagtcacgacgtt

pENTR2-R2-PBR-L4

atgcctggcagttccctactctcgcgtcaacgctagcatggatctcgggccctgcagctctagagctcgaattctacaggtcactaataccatctaagtagttggttcatagtgactgcatatgttgtgttttacagtattatgtagtctgttttttatgcaaaatctaatttaatatattgatatttatatcattttacgtttctcgttcaactttcttgtacaaagtgggatatctataacaagaaaatatatatataataagttatcacgtaagtagaacatgaaataacaatataattatcgtatgagttaaatcttaaaagtcacgtaaaagataatcatgcgtcattttgactcacgcggtcgttatagttcaaaatcagtgacacttaccgcattgacaagcacgcctcacgggagctccaagcggcgactgagatgtcctaaatgcacagcgacggattcgcgctatttagaaagagagagcaatatttcaagaatgcatgcgtcaattttacgcagactatctttctagggttaacaacttttctatacaaagttggcattataagaaagcattgcttatcaatttgttgcaacgaacaggtcactatcagtcaaaataaaatcattatttgccatccagctgcagctctggcccgtgtctcaaaatctctgatgttacattgcacaagataaaaatatatcatcatgaacaataaaactgtctgcttacataaacagtaatacaaggggtgttatgagccatattcaacgggaaacgtcgaggccgcgattaaattccaacatggatgctgatttatatgggtataaatgggctcgcgataatgtcgggcaatcaggtgcgacaatctatcgcttgtatgggaagcccgatgcgccagagttgtttctgaaacatggcaaaggtagcgttgccaatgatgttacagatgagatggtcagactaaactggctgacggaatttatgcctcttccgaccatcaagcattttatccgtactcctgatgatgcatggttactcaccactgcgatccccggaaaaacagcattccaggtattagaagaatatcctgattcaggtgaaaatattgttgatgcgctggcagtgttcctgcgccggttgcattcgattcctgtttgtaattgtccttttaacagcgatcgcgtatttcgtctcgctcaggcgcaatcacgaatgaataacggtttggttgatgcgagtgattttgatgacgagcgtaatggctggcctgttgaacaagtctggaaagaaatgcataaacttttgccattctcaccggattcagtcgtcactcatggtgatttctcacttgataaccttatttttgacgaggggaaattaataggttgtattgatgttggacgagtcggaatcgcagaccgataccaggatcttgccatcctatggaactgcctcggtgagttttctccttcattacagaaacggctttttcaaaaatatggtattgataatcctgatatgaataaattgcagtttcatttgatgctcgatgagtttttctaatcagaattggttaattggttgtaacactggcagagcattacgctgacttgacgggacggcgcaagctcatgaccaaaatcccttaacgtgagttttcgttccactgagcgtcagaccccgtagaaaagatcaaaggatcttcttgagatcctttttttctgcgcgtaatctgctgcttgcaaacaaaaaaaccaccgctaccagcggtggtttgtttgccggatcaagagctaccaactctttttccgaaggtaactggcttcagcagagcgcagataccaaatactgtccttctagtgtagccgtagttaggccaccacttcaagaactctgtagcaccgcctacatacctcgctctgctaatcctgttaccagtggctgctgccagtggcgataagtcgtgtcttaccgggttggactcaagacgatagttaccggataaggcgcagcggtcgggctgaacggggggttcgtgcacacagcccagcttggagcgaacgacctacaccgaactgagatacctacagcgtgagctatgagaaagcgccacgcttcccgaagggagaaaggcggacaggtatccggtaagcggcagggtcggaacaggagagcgcacgagggagcttccagggggaaacgcctggtatctttatagtcctgtcgggtttcgccacctctgacttgagcgtcgatttttgtgatgctcgtcaggggggcggagcctatggaaaaacgccagcaacgcggcctttttacggttcctggccttttgctggccttttgctcacatgttctttcctgcgttatcccctgattctgtggataaccgtattaccgctagccaggaagagtttgtagaaacgcaaaaaggccatccgtcaggatggccttctgcttagtttgatgcctggcagtttatggcgggcgtcctgcccgccaccctccgggccgttgcttcacaacgttcaaatccgctcccggcggatttgtcctactcaggagagcgttcaccgacaaacaacagataaaacgaaaggcccagtcttccgactgagcctttcgttttatttg

pENTR2-L3-SfoI-EGFP-PBL-R1

gagggcctatttcccatgattccttcatatttgcatatacgatacaaggctgttagagagataattggaattaatttgactgtaaacacaaagatattagtacaaaatacgtgacgtagaaagtaataatttcttgggtagtttgcagttttaaaattatgttttaaaatggactatcatatgcttaccgtaacttgaaagtatttcgatttcttggctttatatatcttGTGGAAAGGACGAAACACCggGTCTTCgaGAAGACctgttttagagctaGAAAtagcaagttaaaataaggctagtccgttatcaacttgaaaaagtggcaccgagtcggtgcTTTTTTgttttagagctagaaatagcaagttaaaataaggctagtccgtTTTTagcgcgtgcgccaattctgcagacaaatggctctagaggtacccgttacataacttacggtaaatggcccgcctggctgaccgcccaacgacccccgcccattgacgtcaatagtaacgccaatagggactttccattgacgtcaatgggtggagtatttacggtaaactgcccacttggcagtacatcaagtgtatcatatgccaagtacgccccctattgacgtcaatgacggtaaatggcccgcctggcattGtgcccagtacatgaccttatgggactttcctacttggcagtacatctacgtattagtcatcgctattaccatggtcgaggtgagccccacgttctgcttcactctccccatctcccccccctccccacccccaattttgtatttatttattttttaattattttgtgcagcgatgggggcggggggggggggggggcgcgcgccaggcggggcggggcggggcgaggggcggggcggggcgaggcggagaggtgcggcggcagccaatcagagcggcgcgctccgaaagtttccttttatggcgaggcggcggcggcggcggccctataaaaagcgaagcgcgcggcgggcgggagtcgctgcgacgctgccttcgccccgtgccccgctccgccgccgcctcgcgccgcccgccccggctctgactgaccgcgttactcccacaggtgagcgggcgggacggcccttctcctccgggctgtaattagctgagcaagaggtaagggtttaagggatggttggttggtggggtattaatgtttaattacctggagcacctgcctgaaatcactttttttcaggttGGaccggtgtaaaacgacggccagtcttaagctcgggccccaaataatgattttatttCgactgatagtgacctgttcgttgcaacaaattgatgagcaatgcttttttataatgccaactttgtataataaagttgttgccaccatgggcGCCACGAACTTCTCTCTGTTAAAGCAAGCAGGAGACGTGGAAGAAAACCCCGGTCCTgttAGCAAGGGCGAGGAGCTGTTCACCGGGGTGGTGCCCATCCTGGTCGAGCTGGACGGCGACGTAAACGGCCACAAGTTCAGCGTGTCCGGCGAGGGCGAGGGCGATGCCACCTACGGCAAGCTGACCCTGAAGTTCATCTGCACCACCGGCAAGCTGCCCGTGCCCTGGCCCACCCTCGTGACCACCCTGACCTACGGCGTGCAGTGCTTCAGCCGCTACCCCGACCACATGAAGCAGCACGACTTCTTCAAGTCCGCCATGCCCGAAGGCTACGTCCAGGAGCGCACCATCTTCTTCAAGGACGACGGCAACTACAAGACCCGCGCCGAGGTGAAGTTCGAGGGCGACACCCTGGTGAACCGCATCGAGCTGAAGGGCATCGACTTCAAGGAGGACGGCAACATCCTGGGGCACAAGCTGGAGTACAACTACAACAGCCACAACGTCTATATCATGGCCGACAAGCAGAAGAACGGCATCAAGGTGAACTTCAAGATCCGCCACAACATCGAGGACGGCAGCGTGCAGCTCGCCGACCACTACCAGCAGAACACCCCCATCGGCGACGGCCCCGTGCTGCTGCCCGACAACCACTACCTGAGCACCCAGTCCGCCCTGAGCAAAGACCCCAACGAGAAGCGCGATCACATGGTCCTGCTGGAGTTCGTGACCGCCGCCGGGATCACTCTCGGCATGGACGAGCTGTACAAGgaattttaaccctagaaagataatcatattgtgacgtacgttaaagataatcatgcgtaaaattgacgcatgtgttttatcggtctgtatatcgaggtcccgtgaggcgtttgaatagatattaagttttattatatttacacttacatactaataataaattcaacaaacaattcttttacgtgacttttaagatttaactcaaacaaaaactcaaaatttcttctataaagtaacaaaactcaagtttgtacaaaaaagttgaacgagaaacgtaaaatgatataaatatcaatatattaaattagattttgcataaaaaacagactacataatactgtaaaacacaacatatgcagtcactatgaatcaactacttagatggtattagtgacctgtagaattctaaCTAGAGCTCGCTGATCAGCCTCGACTGTGCCTTCTAGTTGCCAGCCATCTGTTGTTTGCCCCTCCCCCGTGCCTTCCTTGACCCTGGAAGGTGCCACTCCCACTGTCCTTTCCTAATAAAATGAGGAAATTGCATCGCATTGTCTGAGTAGGTGTCATTCTATTCTGGGGGGTGGGGTGGGGCAGGACAGCAAGGGGGAGGATTGGGAAGAgAATAGCAGGCATGCTGGGGAgcggccgcgatatcccctatagtgagtcgtattacatggtcatagctgtttcctggcagctctggcccgtgtctcaaaatctctgatgttacattgcacaagataaaaatatatcatcatgaacaataaaactgtctgcttacataaacagtaatacaaggggtgttatgagccatattcaacgggaaacgtcgaggccgcgattaaattccaacatggatgctgatttatatgggtataaatgggctcgcgataatgtcgggcaatcaggtgcgacaatctatcgcttgtatgggaagcccgatgcgccagagttgtttctgaaacatggcaaaggtagcgttgccaatgatgttacagatgagatggtcagactaaactggctgacggaatttatgcctcttccgaccatcaagcattttatccgtactcctgatgatgcatggttactcaccactgcgatccccggaaaaacagcattccaggtattagaagaatatcctgattcaggtgaaaatattgttgatgcgctggcagtgttcctgcgccggttgcattcgattcctgtttgtaattgtccttttaacagcgatcgcgtatttcgtctcgctcaggcgcaatcacgaatgaataacggtttggttgatgcgagtgattttgatgacgagcgtaatggctggcctgttgaacaagtctggaaagaaatgcataaacttttgccattctcaccggattcagtcgtcactcatggtgatttctcacttgataaccttatttttgacgaggggaaattaataggttgtattgatgttggacgagtcggaatcgcagaccgataccaggatcttgccatcctatggaactgcctcggtgagttttctccttcattacagaaacggctttttcaaaaatatggtattgataatcctgatatgaataaattgcagtttcatttgatgctcgatgagtttttctaatcagaattggttaattggttgtaacactggcagagcattacgctgacttgacgggacggcgcaagctcatgaccaaaatcccttaacgtgagttacgcgtcgttccactgagcgtcagaccccgtagaaaagatcaaaggatcttcttgagatcctttttttctgcgcgtaatctgctgcttgcaaacaaaaaaaccaccgctaccagcggtggtttgtttgccggatcaagagctaccaactctttttccgaaggtaactggcttcagcagagcgcagataccaaatactgtccttctagtgtagccgtagttaggccaccacttcaagaactctgtagcaccgcctacatacctcgctctgctaatcctgttaccagtggctgctgccagtggcgataagtcgtgtcttaccgggttggactcaagacgatagttaccggataaggcgcagcggtcgggctgaacggggggttcgtgcacacagcccagcttggagcgaacgacctacaccgaactgagatacctacagcgtgagctatgagaaagcgccacgcttcccgaagggagaaaggcggacaggtatccggtaagcggcagggtcggaacaggagagcgcacgagggagcttccagggggaaacgcctggtatctttatagtcctgtcgggtttcgccacctctgacttgagcgtcgatttttgtgatgctcgtcaggggggcggagcctatggaaaaacgccagcaacgcggcctttttacggttcctggccttttgctggccttttgctcacatgt

pENTR2-L3-SfoI-Venus-PBL-R1

gagggcctatttcccatgattccttcatatttgcatatacgatacaaggctgttagagagataattggaattaatttgactgtaaacacaaagatattagtacaaaatacgtgacgtagaaagtaataatttcttgggtagtttgcagttttaaaattatgttttaaaatggactatcatatgcttaccgtaacttgaaagtatttcgatttcttggctttatatatcttGTGGAAAGGACGAAACACCggGTCTTCgaGAAGACctgttttagagctaGAAAtagcaagttaaaataaggctagtccgttatcaacttgaaaaagtggcaccgagtcggtgcTTTTTTgttttagagctagaaatagcaagttaaaataaggctagtccgtTTTTagcgcgtgcgccaattctgcagacaaatggctctagaggtacccgttacataacttacggtaaatggcccgcctggctgaccgcccaacgacccccgcccattgacgtcaatagtaacgccaatagggactttccattgacgtcaatgggtggagtatttacggtaaactgcccacttggcagtacatcaagtgtatcatatgccaagtacgccccctattgacgtcaatgacggtaaatggcccgcctggcattGtgcccagtacatgaccttatgggactttcctacttggcagtacatctacgtattagtcatcgctattaccatggtcgaggtgagccccacgttctgcttcactctccccatctcccccccctccccacccccaattttgtatttatttattttttaattattttgtgcagcgatgggggcggggggggggggggggcgcgcgccaggcggggcggggcggggcgaggggcggggcggggcgaggcggagaggtgcggcggcagccaatcagagcggcgcgctccgaaagtttccttttatggcgaggcggcggcggcggcggccctataaaaagcgaagcgcgcggcgggcgggagtcgctgcgacgctgccttcgccccgtgccccgctccgccgccgcctcgcgccgcccgccccggctctgactgaccgcgttactcccacaggtgagcgggcgggacggcccttctcctccgggctgtaattagctgagcaagaggtaagggtttaagggatggttggttggtggggtattaatgtttaattacctggagcacctgcctgaaatcactttttttcaggttGGaccggtgtaaaacgacggccagtcttaagctcgggccccaaataatgattttatttCgactgatagtgacctgttcgttgcaacaaattgatgagcaatgcttttttataatgccaactttgtataataaagttgttgccaccatgggcGCCACGAACTTCTCTCTGTTAAAGCAAGCAGGAGACGTGGAAGAAAACCCCGGTCCTgttAGCAAGGGCGAGGAGCTGTTCACCGGGGTGGTGCCCATCCTGGTCGAGCTGGACGGCGACGTAAACGGCCACAAGTTCAGCGTGTCCGGCGAGGGCGAGGGCGATGCCACCTACGGCAAGCTGACCCTGAAGCTGATCTGCACCACCGGCAAGCTGCCCGTGCCCTGGCCCACCCTCGTGACCACCCTGGGCTACGGCCTGCAGTGCTTCGCCCGCTACCCCGACCACATGAAGCAGCACGACTTCTTCAAGTCCGCCATGCCCGAAGGCTACGTCCAGGAGCGCACCATCTTCTTCAAGGACGACGGCAACTACAAGACCCGCGCCGAGGTGAAGTTCGAGGGCGACACCCTGGTGAACCGCATCGAGCTGAAGGGCATCGACTTCAAGGAGGACGGCAACATCCTGGGGCACAAGCTGGAGTACAACTACAACAGCCACAACGTCTATATCACCGCCGACAAGCAGAAGAACGGCATCAAGGCCAACTTCAAGATCCGCCACAACATCGAGGACGGCGGCGTGCAGCTCGCCGACCACTACCAGCAGAACACCCCCATCGGCGACGGCCCCGTGCTGCTGCCCGACAACCACTACCTGAGCTACCAGTCCAAGCTGAGCAAAGACCCCAACGAGAAGCGCGATCACATGGTCCTGCTGGAGTTCGTGACCGCCGCCGGGATCACTCTCGGCATGGACGAGCTGTACAAGgaattttaaccctagaaagataatcatattgtgacgtacgttaaagataatcatgcgtaaaattgacgcatgtgttttatcggtctgtatatcgaggtcccgtgaggcgtttgaatagatattaagttttattatatttacacttacatactaataataaattcaacaaacaattcttttacgtgacttttaagatttaactcaaacaaaaactcaaaatttcttctataaagtaacaaaactcaagtttgtacaaaaaagttgaacgagaaacgtaaaatgatataaatatcaatatattaaattagattttgcataaaaaacagactacataatactgtaaaacacaacatatgcagtcactatgaatcaactacttagatggtattagtgacctgtagaattctaaCTAGAGCTCGCTGATCAGCCTCGACTGTGCCTTCTAGTTGCCAGCCATCTGTTGTTTGCCCCTCCCCCGTGCCTTCCTTGACCCTGGAAGGTGCCACTCCCACTGTCCTTTCCTAATAAAATGAGGAAATTGCATCGCATTGTCTGAGTAGGTGTCATTCTATTCTGGGGGGTGGGGTGGGGCAGGACAGCAAGGGGGAGGATTGGGAAGAgAATAGCAGGCATGCTGGGGAgcggccgcgatatcccctatagtgagtcgtattacatggtcatagctgtttcctggcagctctggcccgtgtctcaaaatctctgatgttacattgcacaagataaaaatatatcatcatgaacaataaaactgtctgcttacataaacagtaatacaaggggtgttatgagccatattcaacgggaaacgtcgaggccgcgattaaattccaacatggatgctgatttatatgggtataaatgggctcgcgataatgtcgggcaatcaggtgcgacaatctatcgcttgtatgggaagcccgatgcgccagagttgtttctgaaacatggcaaaggtagcgttgccaatgatgttacagatgagatggtcagactaaactggctgacggaatttatgcctcttccgaccatcaagcattttatccgtactcctgatgatgcatggttactcaccactgcgatccccggaaaaacagcattccaggtattagaagaatatcctgattcaggtgaaaatattgttgatgcgctggcagtgttcctgcgccggttgcattcgattcctgtttgtaattgtccttttaacagcgatcgcgtatttcgtctcgctcaggcgcaatcacgaatgaataacggtttggttgatgcgagtgattttgatgacgagcgtaatggctggcctgttgaacaagtctggaaagaaatgcataaacttttgccattctcaccggattcagtcgtcactcatggtgatttctcacttgataaccttatttttgacgaggggaaattaataggttgtattgatgttggacgagtcggaatcgcagaccgataccaggatcttgccatcctatggaactgcctcggtgagttttctccttcattacagaaacggctttttcaaaaatatggtattgataatcctgatatgaataaattgcagtttcatttgatgctcgatgagtttttctaatcagaattggttaattggttgtaacactggcagagcattacgctgacttgacgggacggcgcaagctcatgaccaaaatcccttaacgtgagttacgcgtcgttccactgagcgtcagaccccgtagaaaagatcaaaggatcttcttgagatcctttttttctgcgcgtaatctgctgcttgcaaacaaaaaaaccaccgctaccagcggtggtttgtttgccggatcaagagctaccaactctttttccgaaggtaactggcttcagcagagcgcagataccaaatactgtccttctagtgtagccgtagttaggccaccacttcaagaactctgtagcaccgcctacatacctcgctctgctaatcctgttaccagtggctgctgccagtggcgataagtcgtgtcttaccgggttggactcaagacgatagttaccggataaggcgcagcggtcgggctgaacggggggttcgtgcacacagcccagcttggagcgaacgacctacaccgaactgagatacctacagcgtgagctatgagaaagcgccacgcttcccgaagggagaaaggcggacaggtatccggtaagcggcagggtcggaacaggagagcgcacgagggagcttccagggggaaacgcctggtatctttatagtcctgtcgggtttcgccacctctgacttgagcgtcgatttttgtgatgctcgtcaggggggcggagcctatggaaaaacgccagcaacgcggcctttttacggttcctggccttttgctggccttttgctcacatgt

pENTR2-L3-SfoI-Cerulean-PBL-R1

gagggcctatttcccatgattccttcatatttgcatatacgatacaaggctgttagagagataattggaattaatttgactgtaaacacaaagatattagtacaaaatacgtgacgtagaaagtaataatttcttgggtagtttgcagttttaaaattatgttttaaaatggactatcatatgcttaccgtaacttgaaagtatttcgatttcttggctttatatatcttGTGGAAAGGACGAAACACCggGTCTTCgaGAAGACctgttttagagctaGAAAtagcaagttaaaataaggctagtccgttatcaacttgaaaaagtggcaccgagtcggtgcTTTTTTgttttagagctagaaatagcaagttaaaataaggctagtccgtTTTTagcgcgtgcgccaattctgcagacaaatggctctagaggtacccgttacataacttacggtaaatggcccgcctggctgaccgcccaacgacccccgcccattgacgtcaatagtaacgccaatagggactttccattgacgtcaatgggtggagtatttacggtaaactgcccacttggcagtacatcaagtgtatcatatgccaagtacgccccctattgacgtcaatgacggtaaatggcccgcctggcattGtgcccagtacatgaccttatgggactttcctacttggcagtacatctacgtattagtcatcgctattaccatggtcgaggtgagccccacgttctgcttcactctccccatctcccccccctccccacccccaattttgtatttatttattttttaattattttgtgcagcgatgggggcggggggggggggggggcgcgcgccaggcggggcggggcggggcgaggggcggggcggggcgaggcggagaggtgcggcggcagccaatcagagcggcgcgctccgaaagtttccttttatggcgaggcggcggcggcggcggccctataaaaagcgaagcgcgcggcgggcgggagtcgctgcgacgctgccttcgccccgtgccccgctccgccgccgcctcgcgccgcccgccccggctctgactgaccgcgttactcccacaggtgagcgggcgggacggcccttctcctccgggctgtaattagctgagcaagaggtaagggtttaagggatggttggttggtggggtattaatgtttaattacctggagcacctgcctgaaatcactttttttcaggttGGaccggtgtaaaacgacggccagtcttaagctcgggccccaaataatgattttatttCgactgatagtgacctgttcgttgcaacaaattgatgagcaatgcttttttataatgccaactttgtataataaagttgttgccaccatgggcGCCACGAACTTCTCTCTGTTAAAGCAAGCAGGAGACGTGGAAGAAAACCCCGGTCCTgttAGCAAGGGCGAGGAGCTGTTCACCGGGGTGGTGCCCATCCTGGTCGAGCTGGACGGCGACGTAAACGGCCACAAGTTCAGCGTGTCCGGCGAGGGCGAGGGCGATGCCACCTACGGCAAGCTGACCCTGAAGTTCATCTGCACCACCGGCAAGCTGCCCGTGCCCTGGCCCACCCTCGTGACCACCCTGACCTGGGGCGTGCAGTGCTTCGCCCGCTACCCCGACCACATGAAGCAGCACGACTTCTTCAAGTCCGCCATGCCCGAAGGCTACGTCCAGGAGCGCACCATCTTCTTCAAGGACGACGGCAACTACAAGACCCGCGCCGAGGTGAAGTTCGAGGGCGACACCCTGGTGAACCGCATCGAGCTGAAGGGCATCGACTTCAAGGAGGACGGCAACATCCTGGGGCACAAGCTGGAGTACAACGCCATCAGCGACAACGTCTATATCACCGCCGACAAGCAGAAGAACGGCATCAAGGCCAACTTCAAGATCCGCCACAACATCGAGGACGGCAGCGTGCAGCTCGCCGACCACTACCAGCAGAACACCCCCATCGGCGACGGCCCCGTGCTGCTGCCCGACAACCACTACCTGAGCACCCAGTCCAAGCTGAGCAAAGACCCCAACGAGAAGCGCGATCACATGGTCCTGCTGGAGTTCGTGACCGCCGCCGGGATCACTCTCGGCATgGACGAGCTGTACAAGgaattttaaccctagaaagataatcatattgtgacgtacgttaaagataatcatgcgtaaaattgacgcatgtgttttatcggtctgtatatcgaggtcccgtgaggcgtttgaatagatattaagttttattatatttacacttacatactaataataaattcaacaaacaattcttttacgtgacttttaagatttaactcaaacaaaaactcaaaatttcttctataaagtaacaaaactcaagtttgtacaaaaaagttgaacgagaaacgtaaaatgatataaatatcaatatattaaattagattttgcataaaaaacagactacataatactgtaaaacacaacatatgcagtcactatgaatcaactacttagatggtattagtgacctgtagaattctaaCTAGAGCTCGCTGATCAGCCTCGACTGTGCCTTCTAGTTGCCAGCCATCTGTTGTTTGCCCCTCCCCCGTGCCTTCCTTGACCCTGGAAGGTGCCACTCCCACTGTCCTTTCCTAATAAAATGAGGAAATTGCATCGCATTGTCTGAGTAGGTGTCATTCTATTCTGGGGGGTGGGGTGGGGCAGGACAGCAAGGGGGAGGATTGGGAAGAgAATAGCAGGCATGCTGGGGAgcggccgcgatatcccctatagtgagtcgtattacatggtcatagctgtttcctggcagctctggcccgtgtctcaaaatctctgatgttacattgcacaagataaaaatatatcatcatgaacaataaaactgtctgcttacataaacagtaatacaaggggtgttatgagccatattcaacgggaaacgtcgaggccgcgattaaattccaacatggatgctgatttatatgggtataaatgggctcgcgataatgtcgggcaatcaggtgcgacaatctatcgcttgtatgggaagcccgatgcgccagagttgtttctgaaacatggcaaaggtagcgttgccaatgatgttacagatgagatggtcagactaaactggctgacggaatttatgcctcttccgaccatcaagcattttatccgtactcctgatgatgcatggttactcaccactgcgatccccggaaaaacagcattccaggtattagaagaatatcctgattcaggtgaaaatattgttgatgcgctggcagtgttcctgcgccggttgcattcgattcctgtttgtaattgtccttttaacagcgatcgcgtatttcgtctcgctcaggcgcaatcacgaatgaataacggtttggttgatgcgagtgattttgatgacgagcgtaatggctggcctgttgaacaagtctggaaagaaatgcataaacttttgccattctcaccggattcagtcgtcactcatggtgatttctcacttgataaccttatttttgacgaggggaaattaataggttgtattgatgttggacgagtcggaatcgcagaccgataccaggatcttgccatcctatggaactgcctcggtgagttttctccttcattacagaaacggctttttcaaaaatatggtattgataatcctgatatgaataaattgcagtttcatttgatgctcgatgagtttttctaatcagaattggttaattggttgtaacactggcagagcattacgctgacttgacgggacggcgcaagctcatgaccaaaatcccttaacgtgagttacgcgtcgttccactgagcgtcagaccccgtagaaaagatcaaaggatcttcttgagatcctttttttctgcgcgtaatctgctgcttgcaaacaaaaaaaccaccgctaccagcggtggtttgtttgccggatcaagagctaccaactctttttccgaaggtaactggcttcagcagagcgcagataccaaatactgtccttctagtgtagccgtagttaggccaccacttcaagaactctgtagcaccgcctacatacctcgctctgctaatcctgttaccagtggctgctgccagtggcgataagtcgtgtcttaccgggttggactcaagacgatagttaccggataaggcgcagcggtcgggctgaacggggggttcgtgcacacagcccagcttggagcgaacgacctacaccgaactgagatacctacagcgtgagctatgagaaagcgccacgcttcccgaagggagaaaggcggacaggtatccggtaagcggcagggtcggaacaggagagcgcacgagggagcttccagggggaaacgcctggtatctttatagtcctgtcgggtttcgccacctctgacttgagcgtcgatttttgtgatgctcgtcaggggggcggagcctatggaaaaacgccagcaacgcggcctttttacggttcctggccttttgctggccttttgctcacatgt

pENTR2-L3-SfoI-tdTomato-PBL-R1

gagggcctatttcccatgattccttcatatttgcatatacgatacaaggctgttagagagataattggaattaatttgactgtaaacacaaagatattagtacaaaatacgtgacgtagaaagtaataatttcttgggtagtttgcagttttaaaattatgttttaaaatggactatcatatgcttaccgtaacttgaaagtatttcgatttcttggctttatatatcttGTGGAAAGGACGAAACACCggGTCTTCgaGAAGACctgttttagagctaGAAAtagcaagttaaaataaggctagtccgttatcaacttgaaaaagtggcaccgagtcggtgcTTTTTTgttttagagctagaaatagcaagttaaaataaggctagtccgtTTTTagcgcgtgcgccaattctgcagacaaatggctctagaggtacccgttacataacttacggtaaatggcccgcctggctgaccgcccaacgacccccgcccattgacgtcaatagtaacgccaatagggactttccattgacgtcaatgggtggagtatttacggtaaactgcccacttggcagtacatcaagtgtatcatatgccaagtacgccccctattgacgtcaatgacggtaaatggcccgcctggcattGtgcccagtacatgaccttatgggactttcctacttggcagtacatctacgtattagtcatcgctattaccatggtcgaggtgagccccacgttctgcttcactctccccatctcccccccctccccacccccaattttgtatttatttattttttaattattttgtgcagcgatgggggcggggggggggggggggcgcgcgccaggcggggcggggcggggcgaggggcggggcggggcgaggcggagaggtgcggcggcagccaatcagagcggcgcgctccgaaagtttccttttatggcgaggcggcggcggcggcggccctataaaaagcgaagcgcgcggcgggcgggagtcgctgcgacgctgccttcgccccgtgccccgctccgccgccgcctcgcgccgcccgccccggctctgactgaccgcgttactcccacaggtgagcgggcgggacggcccttctcctccgggctgtaattagctgagcaagaggtaagggtttaagggatggttggttggtggggtattaatgtttaattacctggagcacctgcctgaaatcactttttttcaggttGGaccggtgtaaaacgacggccagtcttaagctcgggccccaaataatgattttatttCgactgatagtgacctgttcgttgcaacaaattgatgagcaatgcttttttataatgccaactttgtataataaagttgttgccaccatgggcGCCACGAACTTCTCTCTGTTAAAGCAAGCAGGAGACGTGGAAGAAAACCCCGGTCCTgttAGCAAGGGCGAGGAGGTCATCAAAGAGTTCATGCGCTTCAAGGTGCGCATGGAGGGCTCCATGAACGGCCACGAGTTCGAGATCGAGGGCGAGGGCGAGGGCCGCCCCTACGAGGGCACCCAGACCGCCAAGCTGAAGGTGACCAAGGGCGGCCCCCTGCCCTTCGCCTGGGACATCCTGTCCCCCCAGTTCATGTACGGCTCCAAGGCGTACGTGAAGCACCCCGCCGACATCCCCGATTACAAGAAGCTGTCCTTCCCCGAGGGCTTCAAGTGGGAGCGCGTGATGAACTTCGAGGACGGCGGTCTGGTGACCGTGACCCAGGACTCCTCCCTGCAGGACGGCACGCTGATCTACAAGGTGAAGATGCGCGGCACCAACTTCCCCCCCGACGGCCCCGTAATGCAGAAaAAGACCATGGGCTGGGAGGCCTCCACCGAGCGCCTGTACCCCCGCGACGGCGTGCTGAAGGGCGAGATCCACCAGGCCCTGAAGCTGAAGGACGGCGGCCACTACCTGGTGGAGTTCAAGACCATCTACATGGCCAAGAAGCCCGTGCAACTGCCCGGCTACTACTACGTGGACACCAAGCTGGACATCACCTCCCACAACGAGGACTACACCATCGTGGAACAGTACGAGCGCTCCGAGGGCCGCCACCACCTGTTCCTGGGGCATGGCACCGGCAGCACCGGCAGCGGCAGCTCCGGCACCGCCTCCTCCGAGGACAACAACATGGCCGTCATCAAAGAGTTCATGCGCTTCAAGGTGCGCATGGAGGGCTCCATGAACGGCCACGAGTTCGAGATCGAGGGCGAGGGCGAGGGCCGCCCCTACGAGGGCACCCAGACCGCCAAGCTGAAGGTGACCAAGGGCGGCCCCCTGCCCTTCGCCTGGGACATCCTGTCCCCCCAGTTCATGTACGGCTCCAAGGCGTACGTGAAGCACCCCGCCGACATCCCCGATTACAAGAAGCTGTCCTTCCCCGAGGGCTTCAAGTGGGAGCGCGTGATGAACTTCGAGGACGGCGGTCTGGTGACCGTGACCCAGGACTCCTCCCTGCAGGACGGCACGCTGATCTACAAGGTGAAGATGCGCGGCACCAACTTCCCCCCCGACGGCCCCGTAATGCAGAAaAAGACCATGGGCTGGGAGGCCTCCACCGAGCGCCTGTACCCCCGCGACGGCGTGCTGAAGGGCGAGATCCACCAGGCCCTGAAGCTGAAGGACGGCGGCCACTACCTGGTGGAGTTCAAGACCATCTACATGGCCAAGAAGCCCGTGCAACTGCCCGGCTACTACTACGTGGACACCAAGCTGGACATCACCTCCCACAACGAGGACTACACCATCGTGGAACAGTACGAGCGCTCCGAGGGCCGCCACCACCTGTTCCTGTACGGCATgGACGAGCTGTACAAGgaattttaaccctagaaagataatcatattgtgacgtacgttaaagataatcatgcgtaaaattgacgcatgtgttttatcggtctgtatatcgaggtcccgtgaggcgtttgaatagatattaagttttattatatttacacttacatactaataataaattcaacaaacaattcttttacgtgacttttaagatttaactcaaacaaaaactcaaaatttcttctataaagtaacaaaactcaagtttgtacaaaaaagttgaacgagaaacgtaaaatgatataaatatcaatatattaaattagattttgcataaaaaacagactacataatactgtaaaacacaacatatgcagtcactatgaatcaactacttagatggtattagtgacctgtagaattctaaCTAGAGCTCGCTGATCAGCCTCGACTGTGCCTTCTAGTTGCCAGCCATCTGTTGTTTGCCCCTCCCCCGTGCCTTCCTTGACCCTGGAAGGTGCCACTCCCACTGTCCTTTCCTAATAAAATGAGGAAATTGCATCGCATTGTCTGAGTAGGTGTCATTCTATTCTGGGGGGTGGGGTGGGGCAGGACAGCAAGGGGGAGGATTGGGAAGAgAATAGCAGGCATGCTGGGGAgcggccgcgatatcccctatagtgagtcgtattacatggtcatagctgtttcctggcagctctggcccgtgtctcaaaatctctgatgttacattgcacaagataaaaatatatcatcatgaacaataaaactgtctgcttacataaacagtaatacaaggggtgttatgagccatattcaacgggaaacgtcgaggccgcgattaaattccaacatggatgctgatttatatgggtataaatgggctcgcgataatgtcgggcaatcaggtgcgacaatctatcgcttgtatgggaagcccgatgcgccagagttgtttctgaaacatggcaaaggtagcgttgccaatgatgttacagatgagatggtcagactaaactggctgacggaatttatgcctcttccgaccatcaagcattttatccgtactcctgatgatgcatggttactcaccactgcgatccccggaaaaacagcattccaggtattagaagaatatcctgattcaggtgaaaatattgttgatgcgctggcagtgttcctgcgccggttgcattcgattcctgtttgtaattgtccttttaacagcgatcgcgtatttcgtctcgctcaggcgcaatcacgaatgaataacggtttggttgatgcgagtgattttgatgacgagcgtaatggctggcctgttgaacaagtctggaaagaaatgcataaacttttgccattctcaccggattcagtcgtcactcatggtgatttctcacttgataaccttatttttgacgaggggaaattaataggttgtattgatgttggacgagtcggaatcgcagaccgataccaggatcttgccatcctatggaactgcctcggtgagttttctccttcattacagaaacggctttttcaaaaatatggtattgataatcctgatatgaataaattgcagtttcatttgatgctcgatgagtttttctaatcagaattggttaattggttgtaacactggcagagcattacgctgacttgacgggacggcgcaagctcatgaccaaaatcccttaacgtgagttacgcgtcgttccactgagcgtcagaccccgtagaaaagatcaaaggatcttcttgagatcctttttttctgcgcgtaatctgctgcttgcaaacaaaaaaaccaccgctaccagcggtggtttgtttgccggatcaagagctaccaactctttttccgaaggtaactggcttcagcagagcgcagataccaaatactgtccttctagtgtagccgtagttaggccaccacttcaagaactctgtagcaccgcctacatacctcgctctgctaatcctgttaccagtggctgctgccagtggcgataagtcgtgtcttaccgggttggactcaagacgatagttaccggataaggcgcagcggtcgggctgaacggggggttcgtgcacacagcccagcttggagcgaacgacctacaccgaactgagatacctacagcgtgagctatgagaaagcgccacgcttcccgaagggagaaaggcggacaggtatccggtaagcggcagggtcggaacaggagagcgcacgagggagcttccagggggaaacgcctggtatctttatagtcctgtcgggtttcgccacctctgacttgagcgtcgatttttgtgatgctcgtcaggggggcggagcctatggaaaaacgccagcaacgcggcctttttacggttcctggccttttgctggccttttgctcacatgt

pENTR2-L3-SfoI-mCherry-PBL-R1

gagggcctatttcccatgattccttcatatttgcatatacgatacaaggctgttagagagataattggaattaatttgactgtaaacacaaagatattagtacaaaatacgtgacgtagaaagtaataatttcttgggtagtttgcagttttaaaattatgttttaaaatggactatcatatgcttaccgtaacttgaaagtatttcgatttcttggctttatatatcttGTGGAAAGGACGAAACACCggGTCTTCgaGAAGACctgttttagagctaGAAAtagcaagttaaaataaggctagtccgttatcaacttgaaaaagtggcaccgagtcggtgcTTTTTTgttttagagctagaaatagcaagttaaaataaggctagtccgtTTTTagcgcgtgcgccaattctgcagacaaatggctctagaggtacccgttacataacttacggtaaatggcccgcctggctgaccgcccaacgacccccgcccattgacgtcaatagtaacgccaatagggactttccattgacgtcaatgggtggagtatttacggtaaactgcccacttggcagtacatcaagtgtatcatatgccaagtacgccccctattgacgtcaatgacggtaaatggcccgcctggcattGtgcccagtacatgaccttatgggactttcctacttggcagtacatctacgtattagtcatcgctattaccatggtcgaggtgagccccacgttctgcttcactctccccatctcccccccctccccacccccaattttgtatttatttattttttaattattttgtgcagcgatgggggcggggggggggggggggcgcgcgccaggcggggcggggcggggcgaggggcggggcggggcgaggcggagaggtgcggcggcagccaatcagagcggcgcgctccgaaagtttccttttatggcgaggcggcggcggcggcggccctataaaaagcgaagcgcgcggcgggcgggagtcgctgcgacgctgccttcgccccgtgccccgctccgccgccgcctcgcgccgcccgccccggctctgactgaccgcgttactcccacaggtgagcgggcgggacggcccttctcctccgggctgtaattagctgagcaagaggtaagggtttaagggatggttggttggtggggtattaatgtttaattacctggagcacctgcctgaaatcactttttttcaggttGGaccggtgtaaaacgacggccagtcttaagctcgggccccaaataatgattttatttCgactgatagtgacctgttcgttgcaacaaattgatgagcaatgcttttttataatgccaactttgtataataaagttgttgccaccatgggcGCCACGAACTTCTCTCTGTTAAAGCAAGCAGGAGACGTGGAAGAAAACCCCGGTCCTgttAGCAAGGGCGAGGAGgataacatggccatcatcaaggagttcatgcgcttcaaggtgcacatggagggctccgtgaacggccacgagttcgagatcgagggcgagggcgagggccgcccctacgagggcacccagaccgccaagctgaaggtgaccaagggtggccccctgcccttcgcctgggacatcctgtcccctcagttcatgtacggctccaaggcctacgtgaagcaccccgccgacatccccgactacttgaagctgtccttccccgagggcttcaagtgggagcgcgtgatgaacttcgaggacggcggcgtggtgaccgtgacccaggactcctccctgcaggacggcgagttcatctacaaggtgaagctgcgcggcaccaacttcccctccgacggccccgtaatgcagaagaagaccatgggctgggaggcctcctccgagcggatgtaccccgaggacggAgccctgaagggcgagatcaagcagaggctgaagctgaaggacggcggccactacgacgctgaggtcaagaccacctacaaggccaagaagcccgtgcagctgcccggAgcctacaacgtcaacatcaagttggacatcacctcccacaacgaggactacaccatcgtggaacagtacgaacgcgccgagggccgccactccaccggcggcatgGACGAGCTGTACAAGgaattttaaccctagaaagataatcatattgtgacgtacgttaaagataatcatgcgtaaaattgacgcatgtgttttatcggtctgtatatcgaggtcccgtgaggcgtttgaatagatattaagttttattatatttacacttacatactaataataaattcaacaaacaattcttttacgtgacttttaagatttaactcaaacaaaaactcaaaatttcttctataaagtaacaaaactcaagtttgtacaaaaaagttgaacgagaaacgtaaaatgatataaatatcaatatattaaattagattttgcataaaaaacagactacataatactgtaaaacacaacatatgcagtcactatgaatcaactacttagatggtattagtgacctgtagaattctaaCTAGAGCTCGCTGATCAGCCTCGACTGTGCCTTCTAGTTGCCAGCCATCTGTTGTTTGCCCCTCCCCCGTGCCTTCCTTGACCCTGGAAGGTGCCACTCCCACTGTCCTTTCCTAATAAAATGAGGAAATTGCATCGCATTGTCTGAGTAGGTGTCATTCTATTCTGGGGGGTGGGGTGGGGCAGGACAGCAAGGGGGAGGATTGGGAAGAgAATAGCAGGCATGCTGGGGAgcggccgcgatatcccctatagtgagtcgtattacatggtcatagctgtttcctggcagctctggcccgtgtctcaaaatctctgatgttacattgcacaagataaaaatatatcatcatgaacaataaaactgtctgcttacataaacagtaatacaaggggtgttatgagccatattcaacgggaaacgtcgaggccgcgattaaattccaacatggatgctgatttatatgggtataaatgggctcgcgataatgtcgggcaatcaggtgcgacaatctatcgcttgtatgggaagcccgatgcgccagagttgtttctgaaacatggcaaaggtagcgttgccaatgatgttacagatgagatggtcagactaaactggctgacggaatttatgcctcttccgaccatcaagcattttatccgtactcctgatgatgcatggttactcaccactgcgatccccggaaaaacagcattccaggtattagaagaatatcctgattcaggtgaaaatattgttgatgcgctggcagtgttcctgcgccggttgcattcgattcctgtttgtaattgtccttttaacagcgatcgcgtatttcgtctcgctcaggcgcaatcacgaatgaataacggtttggttgatgcgagtgattttgatgacgagcgtaatggctggcctgttgaacaagtctggaaagaaatgcataaacttttgccattctcaccggattcagtcgtcactcatggtgatttctcacttgataaccttatttttgacgaggggaaattaataggttgtattgatgttggacgagtcggaatcgcagaccgataccaggatcttgccatcctatggaactgcctcggtgagttttctccttcattacagaaacggctttttcaaaaatatggtattgataatcctgatatgaataaattgcagtttcatttgatgctcgatgagtttttctaatcagaattggttaattggttgtaacactggcagagcattacgctgacttgacgggacggcgcaagctcatgaccaaaatcccttaacgtgagttacgcgtcgttccactgagcgtcagaccccgtagaaaagatcaaaggatcttcttgagatcctttttttctgcgcgtaatctgctgcttgcaaacaaaaaaaccaccgctaccagcggtggtttgtttgccggatcaagagctaccaactctttttccgaaggtaactggcttcagcagagcgcagataccaaatactgtccttctagtgtagccgtagttaggccaccacttcaagaactctgtagcaccgcctacatacctcgctctgctaatcctgttaccagtggctgctgccagtggcgataagtcgtgtcttaccgggttggactcaagacgatagttaccggataaggcgcagcggtcgggctgaacggggggttcgtgcacacagcccagcttggagcgaacgacctacaccgaactgagatacctacagcgtgagctatgagaaagcgccacgcttcccgaagggagaaaggcggacaggtatccggtaagcggcagggtcggaacaggagagcgcacgagggagcttccagggggaaacgcctggtatctttatagtcctgtcgggtttcgccacctctgacttgagcgtcgatttttgtgatgctcgtcaggggggcggagcctatggaaaaacgccagcaacgcggcctttttacggttcctggccttttgctggccttttgctcacatgt

pENTR2-L3-SfoI-iRFP713-PBL-R1

gagggcctatttcccatgattccttcatatttgcatatacgatacaaggctgttagagagataattggaattaatttgactgtaaacacaaagatattagtacaaaatacgtgacgtagaaagtaataatttcttgggtagtttgcagttttaaaattatgttttaaaatggactatcatatgcttaccgtaacttgaaagtatttcgatttcttggctttatatatcttGTGGAAAGGACGAAACACCggGTCTTCgaGAAGACctgttttagagctaGAAAtagcaagttaaaataaggctagtccgttatcaacttgaaaaagtggcaccgagtcggtgcTTTTTTgttttagagctagaaatagcaagttaaaataaggctagtccgtTTTTagcgcgtgcgccaattctgcagacaaatggctctagaggtacccgttacataacttacggtaaatggcccgcctggctgaccgcccaacgacccccgcccattgacgtcaatagtaacgccaatagggactttccattgacgtcaatgggtggagtatttacggtaaactgcccacttggcagtacatcaagtgtatcatatgccaagtacgccccctattgacgtcaatgacggtaaatggcccgcctggcattGtgcccagtacatgaccttatgggactttcctacttggcagtacatctacgtattagtcatcgctattaccatggtcgaggtgagccccacgttctgcttcactctccccatctcccccccctccccacccccaattttgtatttatttattttttaattattttgtgcagcgatgggggcggggggggggggggggcgcgcgccaggcggggcggggcggggcgaggggcggggcggggcgaggcggagaggtgcggcggcagccaatcagagcggcgcgctccgaaagtttccttttatggcgaggcggcggcggcggcggccctataaaaagcgaagcgcgcggcgggcgggagtcgctgcgacgctgccttcgccccgtgccccgctccgccgccgcctcgcgccgcccgccccggctctgactgaccgcgttactcccacaggtgagcgggcgggacggcccttctcctccgggctgtaattagctgagcaagaggtaagggtttaagggatggttggttggtggggtattaatgtttaattacctggagcacctgcctgaaatcactttttttcaggttGGaccggtgtaaaacgacggccagtcttaagctcgggccccaaataatgattttatttCgactgatagtgacctgttcgttgcaacaaattgatgagcaatgcttttttataatgccaactttgtataataaagttgttgccaccatgggcGCCACGAACTTCTCTCTGTTAAAGCAAGCAGGAGACGTGGAAGAAAACCCCGGTCCTgttATGGCCGAAGGGAGCGTGGCAAGACAGCCCGATCTGCTGACATGCGACGACGAACCAATCCACATTCCAGGAGCTATCCAGCCTCATGGACTGCTGCTGGCTCTGGCCGCTGACATGACTATCGTGGCAGGCTCTGATAACCTGCCAGAGCTGACCGGGCTGGCTATCGGAGCACTGATTGGCCGGTCCGCAGCCGACGTGTTCGATTCTGAGACTCACAATAGACTGACCATCGCCCTGGCTGAACCTGGGGCTGCAGTGGGAGCCCCAATTACCGTCGGCTTCACAATGCGAAAGGACGCTGGCTTTATCGGGTCCTGGCACCGCCATGATCAGCTGATTTTTCTGGAGCTGGAACCACCTCAGAGAGACGTGGCAGAGCCACAGGCTTTCTTTCGGAGAACAAACAGCGCCATCAGGCGgCTGCAGGCCGCTGAGACTCTGGAATCCGCTTGCGCAGCCGCTGCACAGGAAGTGAGGAAGATCACCGGGTTCGATCGCGTCATGATCTACAGATTCGCCTCAGACTTTAGCGGAGAAGTGATCGCAGAAGATCGCTGTGCCGAGGTCGAAAGTAAACTGGGCCTGCATTACCCAGCCTCAACCGTGCCTGCACAGGCACGACGGCTGTATACAATCAACCCCGTGCGGATCATTCCCGACATTAATTACAGACCAGTGCCCGTCACACCTGACCTGAACCCAGTGACTGGGAGGCCCATCGATCTGAGCTTCGCCATTCTGCGCAGCGTGTCCCCTGTCCACCTGGAGTTTATGAGGAATATCGGAATGCATGGCACCATGTCTATCAGTATTCTGAGAGGCGAAAGGCTGTGGGGGCTGATCGTGTGCCACCATCGGACACCTTACTATGTGGACCTGGACGGCAGGCAGGCTTGTGAACTGGTGGCTCAGGTGCTGGCTTGGCAGATTGGCGTGATGGAAGAACTttaaccctagaaagataatcatattgtgacgtacgttaaagataatcatgcgtaaaattgacgcatgtgttttatcggtctgtatatcgaggtcccgtgaggcgtttgaatagatattaagttttattatatttacacttacatactaataataaattcaacaaacaattcttttacgtgacttttaagatttaactcaaacaaaaactcaaaatttcttctataaagtaacaaaactcaagtttgtacaaaaaagttgaacgagaaacgtaaaatgatataaatatcaatatattaaattagattttgcataaaaaacagactacataatactgtaaaacacaacatatgcagtcactatgaatcaactacttagatggtattagtgacctgtagaattctaaCTAGAGCTCGCTGATCAGCCTCGACTGTGCCTTCTAGTTGCCAGCCATCTGTTGTTTGCCCCTCCCCCGTGCCTTCCTTGACCCTGGAAGGTGCCACTCCCACTGTCCTTTCCTAATAAAATGAGGAAATTGCATCGCATTGTCTGAGTAGGTGTCATTCTATTCTGGGGGGTGGGGTGGGGCAGGACAGCAAGGGGGAGGATTGGGAAGAgAATAGCAGGCATGCTGGGGAgcggccgcgatatcccctatagtgagtcgtattacatggtcatagctgtttcctggcagctctggcccgtgtctcaaaatctctgatgttacattgcacaagataaaaatatatcatcatgaacaataaaactgtctgcttacataaacagtaatacaaggggtgttatgagccatattcaacgggaaacgtcgaggccgcgattaaattccaacatggatgctgatttatatgggtataaatgggctcgcgataatgtcgggcaatcaggtgcgacaatctatcgcttgtatgggaagcccgatgcgccagagttgtttctgaaacatggcaaaggtagcgttgccaatgatgttacagatgagatggtcagactaaactggctgacggaatttatgcctcttccgaccatcaagcattttatccgtactcctgatgatgcatggttactcaccactgcgatccccggaaaaacagcattccaggtattagaagaatatcctgattcaggtgaaaatattgttgatgcgctggcagtgttcctgcgccggttgcattcgattcctgtttgtaattgtccttttaacagcgatcgcgtatttcgtctcgctcaggcgcaatcacgaatgaataacggtttggttgatgcgagtgattttgatgacgagcgtaatggctggcctgttgaacaagtctggaaagaaatgcataaacttttgccattctcaccggattcagtcgtcactcatggtgatttctcacttgataaccttatttttgacgaggggaaattaataggttgtattgatgttggacgagtcggaatcgcagaccgataccaggatcttgccatcctatggaactgcctcggtgagttttctccttcattacagaaacggctttttcaaaaatatggtattgataatcctgatatgaataaattgcagtttcatttgatgctcgatgagtttttctaatcagaattggttaattggttgtaacactggcagagcattacgctgacttgacgggacggcgcaagctcatgaccaaaatcccttaacgtgagttacgcgtcgttccactgagcgtcagaccccgtagaaaagatcaaaggatcttcttgagatcctttttttctgcgcgtaatctgctgcttgcaaacaaaaaaaccaccgctaccagcggtggtttgtttgccggatcaagagctaccaactctttttccgaaggtaactggcttcagcagagcgcagataccaaatactgtccttctagtgtagccgtagttaggccaccacttcaagaactctgtagcaccgcctacatacctcgctctgctaatcctgttaccagtggctgctgccagtggcgataagtcgtgtcttaccgggttggactcaagacgatagttaccggataaggcgcagcggtcgggctgaacggggggttcgtgcacacagcccagcttggagcgaacgacctacaccgaactgagatacctacagcgtgagctatgagaaagcgccacgcttcccgaagggagaaaggcggacaggtatccggtaagcggcagggtcggaacaggagagcgcacgagggagcttccagggggaaacgcctggtatctttatagtcctgtcgggtttcgccacctctgacttgagcgtcgatttttgtgatgctcgtcaggggggcggagcctatggaaaaacgccagcaacgcggcctttttacggttcctggccttttgctggccttttgctcacatgt

pENTR2-L3-SfoI-IRES-EGFP-PBL-R1

gagggcctatttcccatgattccttcatatttgcatatacgatacaaggctgttagagagataattggaattaatttgactgtaaacacaaagatattagtacaaaatacgtgacgtagaaagtaataatttcttgggtagtttgcagttttaaaattatgttttaaaatggactatcatatgcttaccgtaacttgaaagtatttcgatttcttggctttatatatcttGTGGAAAGGACGAAACACCggGTCTTCgaGAAGACctgttttagagctaGAAAtagcaagttaaaataaggctagtccgttatcaacttgaaaaagtggcaccgagtcggtgcTTTTTTgttttagagctagaaatagcaagttaaaataaggctagtccgtTTTTagcgcgtgcgccaattctgcagacaaatggctctagaggtacccgttacataacttacggtaaatggcccgcctggctgaccgcccaacgacccccgcccattgacgtcaatagtaacgccaatagggactttccattgacgtcaatgggtggagtatttacggtaaactgcccacttggcagtacatcaagtgtatcatatgccaagtacgccccctattgacgtcaatgacggtaaatggcccgcctggcattGtgcccagtacatgaccttatgggactttcctacttggcagtacatctacgtattagtcatcgctattaccatggtcgaggtgagccccacgttctgcttcactctccccatctcccccccctccccacccccaattttgtatttatttattttttaattattttgtgcagcgatgggggcggggggggggggggggcgcgcgccaggcggggcggggcggggcgaggggcggggcggggcgaggcggagaggtgcggcggcagccaatcagagcggcgcgctccgaaagtttccttttatggcgaggcggcggcggcggcggccctataaaaagcgaagcgcgcggcgggcgggagtcgctgcgacgctgccttcgccccgtgccccgctccgccgccgcctcgcgccgcccgccccggctctgactgaccgcgttactcccacaggtgagcgggcgggacggcccttctcctccgggctgtaattagctgagcaagaggtaagggtttaagggatggttggttggtggggtattaatgtttaattacctggagcacctgcctgaaatcactttttttcaggttGGaccggtgtaaaacgacggccagtcttaagctcgggccccaaataatgattttatttCgactgatagtgacctgttcgttgcaacaaattgatgagcaatgcttttttataatgccaactttgtataataaagttgttgccaccatgggcGCCACGAACTTCTCTCTGTTAGATTACAAGGACGACGATGACAAGtaaagatcttcccttttttttccacAGTCCGCCCCTCTCCCTCCCCCCCCCCTAACGTTACTGGCCGAAGCCGCTTGGAATAAGGCCGGTGTGCGTTTGTCTATATGTTATTTTCCACCATATTGCCGTCTTTTGGCAATGTGAGGGCCCGGAAACCTGGCCCTGTCTTCTTGACGAGCATTCCTAGGGGTCTTTCCCCTCTCGCCAAAGGAATGCAAGGTCTGTTGAATGTCGTGAAGGAAGCAGTTCCTCTGGAAGCTTCTTGAAGACAAACAACGTCTGTAGCGACCCTTTGCAGGCAGCGGAACCCCCCACCTGGCGACAGGTGCCTCTGCGGCCAAAAGCCACGTGTATAAGATACACCTGCAAAGGCGGCACAACCCCAGTGCCACGTTGTGAGTTGGATAGTTGTGGAAAGAGTCAAATGGCTCTCCTCAAGCGTATTCAACAAGGGGCTGAAGGATGCCCAGAAGGTACCCCATTGTATGGGATCTGATCTGGGGCCTCGGTaCACATGCTTTACATGTGTTTAGTCGAGGTTAAAAAAACGTCTAGGCCCCCCGAACCACGGGGACGTGGTTTTCCTTTGAAAAACACGATGATAAggatctgctagccaccATGGTGAGCAAGGGCGAGGAGCTGTTCACCGGGGTGGTGCCCATCCTGGTCGAGCTGGACGGCGACGTAAACGGCCACAAGTTCAGCGTGTCCGGCGAGGGCGAGGGCGATGCCACCTACGGCAAGCTGACCCTGAAGTTCATCTGCACCACCGGCAAGCTGCCCGTGCCCTGGCCCACCCTCGTGACCACCCTGACCTACGGCGTGCAGTGCTTCAGCCGCTACCCCGACCACATGAAGCAGCACGACTTCTTCAAGTCCGCCATGCCCGAAGGCTACGTCCAGGAGCGCACCATCTTCTTCAAGGACGACGGCAACTACAAGACCCGCGCCGAGGTGAAGTTCGAGGGCGACACCCTGGTGAACCGCATCGAGCTGAAGGGCATCGACTTCAAGGAGGACGGCAACATCCTGGGGCACAAGCTGGAGTACAACTACAACAGCCACAACGTCTATATCATGGCCGACAAGCAGAAGAACGGCATCAAGGTGAACTTCAAGATCCGCCACAACATCGAGGACGGCAGCGTGCAGCTCGCCGACCACTACCAGCAGAACACCCCCATCGGCGACGGCCCCGTGCTGCTGCCCGACAACCACTACCTGAGCACCCAGTCCGCCCTGAGCAAAGACCCCAACGAGAAGCGCGATCACATGGTCCTGCTGGAGTTCGTGACCGCCGCCGGGATCACTCTCGGCATGGACGAGCTGTACAAGgaattttaaccctagaaagataatcatattgtgacgtacgttaaagataatcatgcgtaaaattgacgcatgtgttttatcggtctgtatatcgaggtcccgtgaggcgtttgaatagatattaagttttattatatttacacttacatactaataataaattcaacaaacaattcttttacgtgacttttaagatttaactcaaacaaaaactcaaaatttcttctataaagtaacaaaactcaagtttgtacaaaaaagttgaacgagaaacgtaaaatgatataaatatcaatatattaaattagattttgcataaaaaacagactacataatactgtaaaacacaacatatgcagtcactatgaaCcaactacttagatggtattagtgacctgtagaattctaaCTAGAGCTCGCTGATCAGCCTCGACTGTGCCTTCTAGTTGCCAGCCATCTGTTGTTTGCCCCTCCCCCGTGCCTTCCTTGACCCTGGAAGGTGCCACTCCCACTGTCCTTTCCTAATAAAATGAGGAAATTGCATCGCATTGTCTGAGTAGGTGTCATTCTATTCTGGGGGGTGGGGTGGGGCAGGACAGCAAGGGGGAGGATTGGGAAGAgAATAGCAGGCATGCTGGGGAgcggccgcgatatcccctatagtgagtcgtattacatggtcatagctgtttcctggcagctctggcccgtgtctcaaaatctctgatgttacattgcacaagataaaaatatatcatcatgaacaataaaactgtctgcttacataaacagtaatacaaggggtgttatgagccatattcaacgggaaacgtcgaggccgcgattaaattccaacatggatgctgatttatatgggtataaatgggctcgcgataatgtcgggcaatcaggtgcgacaatctatcgcttgtatgggaagcccgatgcgccagagttgtttctgaaacatggcaaaggtagcgttgccaatgatgttacagatgagatggtcagactaaactggctgacggaatttatgcctcttccgaccatcaagcattttatccgtactcctgatgatgcatggttactcaccactgcgatccccggaaaaacagcattccaggtattagaagaatatcctgattcaggtgaaaatattgttgatgcgctggcagtgttcctgcgccggttgcattcgattcctgtttgtaattgtccttttaacagcgatcgcgtatttcgtctcgctcaggcgcaatcacgaatgaataacggtttggttgatgcgagtgattttgatgacgagcgtaatggctggcctgttgaacaagtctggaaagaaatgcataaacttttgccattctcaccggattcagtcgtcactcatggtgatttctcacttgataaccttatttttgacgaggggaaattaataggttgtattgatgttggacgagtcggaatcgcagaccgataccaggatcttgccatcctatggaactgcctcggtgagttttctccttcattacagaaacggctttttcaaaaatatggtattgataatcctgatatgaataaattgcagtttcatttgatgctcgatgagtttttctaatcagaattggttaattggttgtaacactggcagagcattacgctgacttgacgggacggcgcaagctcatgaccaaaatcccttaacgtgagttacgcgtcgttccactgagcgtcagaccccgtagaaaagatcaaaggatcttcttgagatcctttttttctgcgcgtaatctgctgcttgcaaacaaaaaaaccaccgctaccagcggtggtttgtttgccggatcaagagctaccaactctttttccgaaggtaactggcttcagcagagcgcagataccaaatactgtccttctagtgtagccgtagttaggccaccacttcaagaactctgtagcaccgcctacatacctcgctctgctaatcctgttaccagtggctgctgccagtggcgataagtcgtgtcttaccgggttggactcaagacgatagttaccggataaggcgcagcggtcgggctgaacggggggttcgtgcacacagcccagcttggagcgaacgacctacaccgaactgagatacctacagcgtgagctatgagaaagcgccacgcttcccgaagggagaaaggcggacaggtatccggtaagcggcagggtcggaacaggagagcgcacgagggagcttccagggggaaacgcctggtatctttatagtcctgtcgggtttcgccacctctgacttgagcgtcgatttttgtgatgctcgtcaggggggcggagcctatggaaaaacgccagcaacgcggcctttttacggttcctggccttttgctggccttttgctcacatgt

pENTR2-L3-SfoI-EGFP-secNanoLuc-PBL-R1

gagggcctatttcccatgattccttcatatttgcatatacgatacaaggctgttagagagataattggaattaatttgactgtaaacacaaagatattagtacaaaatacgtgacgtagaaagtaataatttcttgggtagtttgcagttttaaaattatgttttaaaatggactatcatatgcttaccgtaacttgaaagtatttcgatttcttggctttatatatcttGTGGAAAGGACGAAACACCggGTCTTCgaGAAGACctgttttagagctaGAAAtagcaagttaaaataaggctagtccgttatcaacttgaaaaagtggcaccgagtcggtgcTTTTTTgttttagagctagaaatagcaagttaaaataaggctagtccgtTTTTagcgcgtgcgccaattctgcagacaaatggctctagaggtacccgttacataacttacggtaaatggcccgcctggctgaccgcccaacgacccccgcccattgacgtcaatagtaacgccaatagggactttccattgacgtcaatgggtggagtatttacggtaaactgcccacttggcagtacatcaagtgtatcatatgccaagtacgccccctattgacgtcaatgacggtaaatggcccgcctggcattGtgcccagtacatgaccttatgggactttcctacttggcagtacatctacgtattagtcatcgctattaccatggtcgaggtgagccccacgttctgcttcactctccccatctcccccccctccccacccccaattttgtatttatttattttttaattattttgtgcagcgatgggggcggggggggggggggggcgcgcgccaggcggggcggggcggggcgaggggcggggcggggcgaggcggagaggtgcggcggcagccaatcagagcggcgcgctccgaaagtttccttttatggcgaggcggcggcggcggcggccctataaaaagcgaagcgcgcggcgggcgggagtcgctgcgacgctgccttcgccccgtgccccgctccgccgccgcctcgcgccgcccgccccggctctgactgaccgcgttactcccacaggtgagcgggcgggacggcccttctcctccgggctgtaattagctgagcaagaggtaagggtttaagggatggttggttggtggggtattaatgtttaattacctggagcacctgcctgaaatcactttttttcaggttGGaccggtgtaaaacgacggccagtcttaagctcgggccccaaataatgattttatttCgactgatagtgacctgttcgttgcaacaaattgatgagcaatgcttttttataatgccaactttgtataataaagttgttgccaccatgggcGCCACGAACTTCTCTCTGTTAAAGCAAGCAGGAGACGTGGAAGAAAACCCCGGTCCTgttAGCAAGGGCGAGGAGCTGTTCACCGGGGTGGTGCCCATCCTGGTCGAGCTGGACGGCGACGTAAACGGCCACAAGTTCAGCGTGTCCGGCGAGGGCGAGGGCGATGCCACCTACGGCAAGCTGACCCTGAAGTTCATCTGCACCACCGGCAAGCTGCCCGTGCCCTGGCCCACCCTCGTGACCACCCTGACCTACGGCGTGCAGTGCTTCAGCCGCTACCCCGACCACATGAAGCAGCACGACTTCTTCAAGTCCGCCATGCCCGAAGGCTACGTCCAGGAGCGCACCATCTTCTTCAAGGACGACGGCAACTACAAGACCCGCGCCGAGGTGAAGTTCGAGGGCGACACCCTGGTGAACCGCATCGAGCTGAAGGGCATCGACTTCAAGGAGGACGGCAACATCCTGGGGCACAAGCTGGAGTACAACTACAACAGCCACAACGTCTATATCATGGCCGACAAGCAGAAGAACGGCATCAAGGTGAACTTCAAGATCCGCCACAACATCGAGGACGGCAGCGTGCAGCTCGCCGACCACTACCAGCAGAACACCCCCATCGGCGACGGCCCCGTGCTGCTGCCCGACAACCACTACCTGAGCACCCAGTCCGCCCTGAGCAAAGACCCCAACGAGAAGCGCGATCACATGGTCCTGCTGGAGTTCGTGACCGCCGCCGGGATCACTCTCGGCATGGACGAGCTGTACAAGGAGGGCAGGGGAAGTCTTCTAACATGCGGGGACGTGGAGGAAAATCCCGGCCCCATGAACTCCTTCTCCACAAGCGCCTTCGGTCCAGTTGCCTTCTCCCTGGGCCTGCTCCTGGTGTTGCCTGCTGCCTTCCCTGCCCCAGTCTTCACACTCGAAGATTTCGTTGGGGACTGGCGACAGACAGCCGGCTACAACCTGGACCAAGTCCTTGAACAGGGAGGTGTGTCCAGTTTGTTTCAGAATCTCGGGGTGTCCGTAACTCCGATCCAAAGGATTGTCCTGAGCGGTGAAAATGGGCTGAAGATCGACATCCATGTCATCATCCCGTATGAAGGTCTGAGCGGCGACCAAATGGGCCAGATCGAAAAAATTTTTAAGGTGGTGTACCCTGTGGATGATCATCACTTTAAGGTGATCCTGCACTATGGCACACTGGTAATCGACGGGGTTACGCCGAACATGATCGACTATTTCGGACGGCCGTATGAAGGCATCGCCGTGTTCGACGGCAAAAAGATCACTGTAACAGGGACCCTGTGGAACGGCAACAAAATTATCGACGAGCGCCTGATCAACCCCGACGGCTCCCTGCTGTTCCGAGTAACCATCAACGGAGTGACCGGCTGGCGGCTGTGCGAACGCATTCTGGCTTAAccctagaaagataatcatattgtgacgtacgttaaagataatcatgcgtaaaattgacgcatgtgttttatcggtctgtatatcgaggtcccgtgaggcgtttgaatagatattaagttttattatatttacacttacatactaataataaattcaacaaacaattcttttacgtgacttttaagatttaactcaaacaaaaactcaaaatttcttctataaagtaacaaaactcaagtttgtacaaaaaagttgaacgagaaacgtaaaatgatataaatatcaatatattaaattagattttgcataaaaaacagactacataatactgtaaaacacaacatatgcagtcactatgaatcaactacttagatggtattagtgacctgtagaattctaaCTAGAGCTCGCTGATCAGCCTCGACTGTGCCTTCTAGTTGCCAGCCATCTGTTGTTTGCCCCTCCCCCGTGCCTTCCTTGACCCTGGAAGGTGCCACTCCCACTGTCCTTTCCTAATAAAATGAGGAAATTGCATCGCATTGTCTGAGTAGGTGTCATTCTATTCTGGGGGGTGGGGTGGGGCAGGACAGCAAGGGGGAGGATTGGGAAGAgAATAGCAGGCATGCTGGGGAgcggccgcgatatcccctatagtgagtcgtattacatggtcatagctgtttcctggcagctctggcccgtgtctcaaaatctctgatgttacattgcacaagataaaaatatatcatcatgaacaataaaactgtctgcttacataaacagtaatacaaggggtgttatgagccatattcaacgggaaacgtcgaggccgcgattaaattccaacatggatgctgatttatatgggtataaatgggctcgcgataatgtcgggcaatcaggtgcgacaatctatcgcttgtatgggaagcccgatgcgccagagttgtttctgaaacatggcaaaggtagcgttgccaatgatgttacagatgagatggtcagactaaactggctgacggaatttatgcctcttccgaccatcaagcattttatccgtactcctgatgatgcatggttactcaccactgcgatccccggaaaaacagcattccaggtattagaagaatatcctgattcaggtgaaaatattgttgatgcgctggcagtgttcctgcgccggttgcattcgattcctgtttgtaattgtccttttaacagcgatcgcgtatttcgtctcgctcaggcgcaatcacgaatgaataacggtttggttgatgcgagtgattttgatgacgagcgtaatggctggcctgttgaacaagtctggaaagaaatgcataaacttttgccattctcaccggattcagtcgtcactcatggtgatttctcacttgataaccttatttttgacgaggggaaattaataggttgtattgatgttggacgagtcggaatcgcagaccgataccaggatcttgccatcctatggaactgcctcggtgagttttctccttcattacagaaacggctttttcaaaaatatggtattgataatcctgatatgaataaattgcagtttcatttgatgctcgatgagtttttctaatcagaattggttaattggttgtaacactggcagagcattacgctgacttgacgggacggcgcaagctcatgaccaaaatcccttaacgtgagttacgcgtcgttccactgagcgtcagaccccgtagaaaagatcaaaggatcttcttgagatcctttttttctgcgcgtaatctgctgcttgcaaacaaaaaaaccaccgctaccagcggtggtttgtttgccggatcaagagctaccaactctttttccgaaggtaactggcttcagcagagcgcagataccaaatactgtccttctagtgtagccgtagttaggccaccacttcaagaactctgtagcaccgcctacatacctcgctctgctaatcctgttaccagtggctgctgccagtggcgataagtcgtgtcttaccgggttggactcaagacgatagttaccggataaggcgcagcggtcgggctgaacggggggttcgtgcacacagcccagcttggagcgaacgacctacaccgaactgagatacctacagcgtgagctatgagaaagcgccacgcttcccgaagggagaaaggcggacaggtatccggtaagcggcagggtcggaacaggagagcgcacgagggagcttccagggggaaacgcctggtatctttatagtcctgtcgggtttcgccacctctgacttgagcgtcgatttttgtgatgctcgtcaggggggcggagcctatggaaaaacgccagcaacgcggcctttttacggttcctggccttttgctggccttttgctcacatgt

pUC-DEST-hSOX2-EGFP-PuroTK

cccagtcacgacgttgtaaaacgacggccagtgccaagcttgcatgcctgcaggtcgggatatctgaattatcaactttgtataataaagttgttgccaccatgggcAACTTTTGTCGGAGACGGAGAAGCGGCCGTTCATCGACGAGGCTAAGCGGCTGCGAGCGCTGCACATGAAGGAGCACCCGGATTATAAATACCGGCCCCGGCGGAAAACCAAGACGCTCATGAAGAAGGATAAGTACACGCTGCCCGGCGGGCTGCTGGCCCCCGGCGGCAATAGCATGGCGAGCGGGGTCGGGGTGGGCGCCGGCCTGGGCGCGGGCGTGAACCAGCGCATGGACAGTTACGCGCACATGAACGGCTGGAGCAACGGCAGCTACAGCATGATGCAGGACCAGCTGGGCTACCCGCAGCACCCGGGCCTCAATGCGCACGGCGCAGCGCAGATGCAGCCCATGCACCGCTACGACGTGAGCGCCCTGCAGTACAACTCCATGACCAGCTCGCAGACCTACATGAACGGCTCGCCCACCTACAGCATGTCCTACTCGCAGCAGGGCACCCCTGGCATGGCTCTTGGCTCCATGGGTTCGGTGGTCAAGTCCGAGGCCAGCTCCAGCCCCCCTGTGGTTACCTCTTCCTCCCACTCCAGGGCGCCCTGCCAGGCCGGGGACCTCCGGGACATGATCAGCATGTATCTCCCCGGCGCCGAGGTGCCGGAACCCGCCGCCCCCAGCAGACTTCACATGTCCCAGCACTACCAGAGCGGCCCGGTGCCCGGCACGGCCATTAACGGCACACTGCCCCTCTCACACATGGCCACGAACTTCTCTCTGTTAAAGCAAGCAGGAGACGTGGAAGAAAACCCCGGTCCTgttAGCAAGGGCGAGGAGCTGTTCACCGGGGTGGTGCCCATCCTGGTCGAGCTGGACGGCGACGTAAACGGCCACAAGTTCAGCGTGTCCGGCGAGGGCGAGGGCGATGCCACCTACGGCAAGCTGACCCTGAAGTTCATCTGCACCACCGGCAAGCTGCCCGTGCCCTGGCCCACCCTCGTGACCACCCTGACCTACGGCGTGCAGTGCTTCAGCCGCTACCCCGACCACATGAAGCAGCACGACTTCTTCAAGTCCGCCATGCCCGAAGGCTACGTCCAGGAGCGCACCATCTTCTTCAAGGACGACGGCAACTACAAGACCCGCGCCGAGGTGAAGTTCGAGGGCGACACCCTGGTGAACCGCATCGAGCTGAAGGGCATCGACTTCAAGGAGGACGGCAACATCCTGGGGCACAAGCTGGAGTACAACTACAACAGCCACAACGTCTATATCATGGCCGACAAGCAGAAGAACGGCATCAAGGTGAACTTCAAGATCCGCCACAACATCGAGGACGGCAGCGTGCAGCTCGCCGACCACTACCAGCAGAACACCCCCATCGGCGACGGCCCCGTGCTGCTGCCCGACAACCACTACCTGAGCACCCAGTCCGCCCTGAGCAAAGACCCCAACGAGAAGCGCGATCACATGGTCCTGCTGGAGTTCGTGACCGCCGCCGGGATCACTCTCGGCATGGACGAGCTGTACAAGgaattttaaccctagaaagataatcatattgtgacgtacgttaaagataatcatgcgtaaaattgacgcatgtgttttatcggtctgtatatcgaggtcccgtgaggcgtttgaatagatattaagttttattatatttacacttacatactaataataaattcaacaaacaattcttttacgtgacttttaagatttaactcaaacaaaaactcaaaatttcttctataaagtaacaaaactcaagtttgtacaaaaaagcaggaattctaccgggtaggggaggcgcttttcccaaggcagtctggagcatgcgctttagcagccccgctgggcacttggcgctacacaagtggcctctggcctcgcacacattccacatccaccggtaggcgccaaccggctccgttctttggtggccccttcgcgccaccttctactcctcccctagtcaggaagttcccccccgccccgcagctcgcgtcgtgcaggacgtgacaaatggaagtagcacgtctcactagtctcgtgcagatggacagcaccgctgagcaatggaagcgggtaggcctttggggcagcggccaatagcagctttgctccttcgctttctgggctcagaggctgggaaggggtgggtccgggggcgggctcaggggcgggctcaggggcggggcgggcgcccgaaggtcctccggaggcccggcattctgcacgcttcaaaagcgcacgtctgccgcgctgttctcctcttcctcatctccgggcctttcgaccgatcatcaagcttgatcctcatgaccgagtacaagcccacggtgcgcctcgccacccgcgacgacgtccccagggccgtacgcaccctcgccgccgcgttcgccgactaccccgccacgcgccacaccgtcgatccggaccgccacatcgagcgggtcaccgagctgcaagaactcttcctcacgcgcgtcgggctcgacatcggcaaggtgtgggtcgcggacgacggcgccgcggtggcggtctggaccacgccggagagcgtcgaagcgggggcggtgttcgccgagatcggcccgcgcatggccgagttgagcggttcccggctggccgcgcagcaacagatggaaggcctcctggcgccgcaccggcccaaggagcccgcgtggttcctggccaccgtcggcgtctcgcccgaccaccagggcaagggtctgggcagcgccgtcgtgctccccggagtggaggcggccgagcgcgccggggtgcccgccttcctggagacctccgcgccccgcaacctccccttctacgagcggctcggcttcaccgtcaccgccgacgtcgaggtgcccgaaggaccgcgcacctggtgcatgacccgcaagcccggtgccggatccatgcccacgctactgcgggtttatatagacggtccccacgggatggggaaaaccaccaccacgcaactgctggtggccctgggttcgcgcgacgatatcgtctacgtacccgagccgatgacttactggcgggtgctgggggcttccgagacaatcgcgaacatctacaccacacaacaccgcctcgaccagggtgagatatcggccggggacgcggcggtggtaatgacaagcgcccagataacaatgggcatgccttatgccgtgaccgacgccgttctggctcctcatatcgggggggaggctgggagctcacatgccccgcccccggccctcaccctcatcttcgaccgccatcccatcgccgccctcctgtgctacccggccgcgcggtaccttatgggcagcatgaccccccaggccgtgctggcgttcgtggccctcatcccgccgaccttgcccggcaccaacatcgtgcttggggcccttccggaggacagacacatcgaccgcctggccaaacgccagcgccccggcgagcggctggacctggctatgctggctgcgattcgccgcgtttacgggctacttgccaatacggtgcggtatctgcagtgcggcgggtcgtggcgggaggactggggacagctttcggggacggccgtgccgccccagggtgccgagccccagagcaacgcgggcccacgaccccatatcggggacacgttatttaccctgtttcgggcccccgagttgctggcccccaacggcgacctgtataacgtgtttgcctgggccttggacgtcttggccaaacgcctccgttccatgcacgtctttatcctggattacgaccaatcgcccgccggctgccgggacgccctgctgcaacttacctccgggatggtccagacccacgtcaccacccccggctccataccgacgatatgcgacctggcgcgcacgtttgcccgggagatgggggaggctaactgagctctagcttatcgactcgccgatagtggaaaccgacgccccagcactcgtccgagggcaaaggaatagtcgagaaattgatgatctattaaacaataaagatgtccacatggaagtttttcctgtcatactttgttaagaagggtgagaacagagtacctacattttgaatggaaggattggagctacgggggtgggggtggggtgggattagataaatgcctgctctttactgaaggctctttactattgctttatgataatgtttcatagttggatatcataatttaaacaagcaaaaccaaattaagggccagctcattcctcccactcatgatctatagatcctctagagtcgagggctgcagcagctttcttgtacaaagtgggatatctataacaagaaaatatatatataataagttatcacgtaagtagaacatgaaataacaatataattatcgtatgagttaaatcttaaaagtcacgtaaaagataatcatgcgtcattttgactcacgcggtcgttatagttcaaaatcagtgacacttaccgcattgacaagcacgcctcacgggagctccaagcggcgactgagatgtcctaaatgcacagcgacggattcgcgctatttagaaagagagagcaatatttcaagaatgcatgcgtcaattttacgcagactatctttctagggttaaTGAGGGttGGACAGCGAACTGGAGGGGGGAGAAATTTTCAAAGAAAAACGAGGGAAATGGGAGGGGTGCAAAAGAGGAGAGTAAGAAACAGCATGGAGAAAACCCGGTACGCTCAAAAAGAAAAAGGAAAAAAAAAAATCCCATCACCCACAGCAAATGACAGCTGCAAAAGAGAACACCAATCCCATCCACACTCACGCAAAAACCGCGATGCCGACAAGAAAACTTTTATGAGAGAGATCCTGGACTTCTTTTTGGGGGACTATTTTTGTACAGAGAAAACCTGGGGAGGGTGGGGAGGGCGGGGGAATGGACCTTGTATAGATCTGGAGGAAAGAAAGCTACGAAAAACTTTTTAAAAGTTCTAGTGGTACGGTAGGAGCTTTGCAGGAAGTTTGCAAAAGTCTTTACCAATAATATTTAGAGCTAGTCTCCAAGCGACGAAAAAAATGTTTTAATATTTGCAAGCAACTTTTGTACAGTATTTATCGAGATAAACATGGCAATCAAAATGTCCATTGTTTATAAGCTGAGAATTTGCCAATATTTTTCAAGGAGAGGCTTCTTGCTGAATTTTGATTCTGCAGCTGAAATTTAGGACAGTTGCAAACGTGAAAAGaacaacttttctatacaaagttgatagcttgatatcaatgactctagaggatccccgggtaccgagctcgaattcgtaatcatggtcatagctgtttcctgtgtgaaattgttatccgctcacaattccacacaacatacgagccggaagcataaagtgtaaagcctggggtgcctaatgagtgagctaactcacattaattgcgttgcgctcactgcccgctttccagtcgggaaacctgtcgtgccagctgcattaatgaatcggccaacgcgcggggagaggcggtttgcgtattgggcgctcttccgcttcctcgctcactgactcgctgcgctcggtcgttcggctgcggcgagcggtatcagctcactcaaaggcggtaatacggttatccacagaatcaggggataacgcaggaaagaacatgtgagcaaaaggccagcaaaaggccaggaaccgtaaaaaggccgcgttgctggcgtttttccataggctccgcccccctgacgagcatcacaaaaatcgacgctcaagtcagaggtggcgaaacccgacaggactataaagataccaggcgtttccccctggaagctccctcgtgcgctctcctgttccgaccctgccgcttaccggatacctgtccgcctttctcccttcgggaagcgtggcgctttctcatagctcacgctgtaggtatctcagttcggtgtaggtcgttcgctccaagctgggctgtgtgcacgaaccccccgttcagcccgaccgctgcgccttatccggtaactatcgtcttgagtccaacccggtaagacacgacttatcgccactggcagcagccactggtaacaggattagcagagcgaggtatgtaggcggtgctacagagttcttgaagtggtggcctaactacggctacactagaaggacagtatttggtatctgcgctctgctgaagccagttaccttcggaaaaagagttggtagctcttgatccggcaaacaaaccaccgctggtagcggtggtttttttgtttgcaagcagcagattacgcgcagaaaaaaaggatctcaagaagatcctttgatcttttctacggggtctgacgctcagtggaacgaaaactcacgttaagggattttggtcatgagattatcaaaaaggatcttcacctagatccttttaaattaaaaatgaagttttaaatcaatctaaagtatatatgagtaaacttggtctgacagttaccaatgcttaatcagtgaggcacctatctcagcgatctgtctatttcgttcatccatagttgcctgactccccgtcgtgtagataactacgatacgggagggcttaccatctggccccagtgctgcaatgataccgcgagacccacgctcaccggctccagatttatcagcaataaaccagccagccggaagggccgagcgcagaagtggtcctgcaactttatccgcctccatccagtctattaattgttgccgggaagctagagtaagtagttcgccagttaatagtttgcgcaacgttgttgccattgctacaggcatcgtggtgtcacgctcgtcgtttggtatggcttcattcagctccggttcccaacgatcaaggcgagttacatgatcccccatgttgtgcaaaaaagcggttagctccttcggtcctccgatcgttgtcagaagtaagttggccgcagtgttatcactcatggttatggcagcactgcataattctcttactgtcatgccatccgtaagatgcttttctgtgactggtgagtactcaaccaagtcattctgagaatagtgtatgcggcgaccgagttgctcttgcccggcgtcaatacgggataataccgcgccacatagcagaactttaaaagtgctcatcattggaaaacgttcttcggggcgaaaactctcaaggatcttaccgctgttgagatccagttcgatgtaacccactcgtgcacccaactgatcttcagcatcttttactttcaccagcgtttctgggtgagcaaaaacaggaaggcaaaatgccgcaaaaaagggaataagggcgacacggaaatgttgaatactcatactcttcctttttcaatattattgaagcatttatcagggttattgtctcatgagcggatacatatttgaatgtatttagaaaaataaacaaataggggttccgcgcacatttccccgaaaagtgccacctgacgtctaagaaaccattattatcatgacattaacctataaaaataggcgtatcacgaggccctttcgtctcgcgcgtttcggtgatgacggtgaaaacctctgacacatgcagctcccggagacggtcacagcttgtctgtaagcggatgccgggagcagacaagcccgtcagggcgcgtcagcgggtgttggcgggtgtcggggctggcttaactatgcggcatcagagcagattgtactgagagtgcaccatatgcggtgtgaaataccgcacagatgcgtaaggagaaaataccgcatcaggcgccattcgccattcaggctgcgcaactgttgggaagggcgatcggtgcgggcctcttcgctattacgccagctggcgaaagggggatgtgctgcaaggcgattaagttgggtaacgccagggtttt

pUC-DEST-hPROX1-tdTomato-PuroTK

cccagtcacgacgttgtaaaacgacggccagtgccaagcttgcatgcctgcaggtcgggatatctgaattatcaactttgtataataaagttgttgccaccatgggcGCATCTGAGTCAACAATCCTCTGAGACAAACAGATAACTGAGATTTTAGAAGATTTTCTTCATTTAAAGCTTGGGTTTAATTTATAAAGAAGCCCAACTATTTGTTATTCTATTTTGAGAACGTATTTTGTTTTCATCATGGCAATCAAAAAGAAATAGGATTCAAATTCTGAAAAAATAATTGGAGACTTTCTTCTGGATAGCACTTATTTAATAAAGTGAGGAATCCCAAAAGTCACATCCCATATTCCTATCCTAATATCCACAATGAAATCCCAGTTTTTCAATAGGTCTGCGTTGGATCTTTCATACACTCTTCTTAAAACAAAGCTGTCAACCCCACATCACAATGCTTCTATATATAATGACTTTACATTAAAAGAATAGAAGCCAGCTATTTTTAGAAAATGCAGGTGCCATGTAAGCCCCTTTCTGCAAGAATGATCTTAGCTCAGTTTCCTTGGAATAACTGTAGACTTGAAACTGAAAACTTTATTAATGCCATTGTCTCCTTGTATCAGCAGGTTCCAGAGAGATTCCTGGAAGTTGCTCAGATCACATTACGGGAGTTTTTCAATGCCATTATCGCAGGCAAAGATGTTGATCCTTCCTGGAAGAAGGCCATATACAAGGTCATCTGCAAGCTGGATAGTGAAGTCCCTGAGATTTTCAAATCCCCGAACTGCCTACAAGAGCTGCTTCATGAGGCCACGAACTTCTCTCTGTTAAAGCAAGCAGGAGACGTGGAAGAAAACCCCGGTCCTgttAGCAAGGGCGAGGAGGTCATCAAAGAGTTCATGCGCTTCAAGGTGCGCATGGAGGGCTCCATGAACGGCCACGAGTTCGAGATCGAGGGCGAGGGCGAGGGCCGCCCCTACGAGGGCACCCAGACCGCCAAGCTGAAGGTGACCAAGGGCGGCCCCCTGCCCTTCGCCTGGGACATCCTGTCCCCCCAGTTCATGTACGGCTCCAAGGCGTACGTGAAGCACCCCGCCGACATCCCCGATTACAAGAAGCTGTCCTTCCCCGAGGGCTTCAAGTGGGAGCGCGTGATGAACTTCGAGGACGGCGGTCTGGTGACCGTGACCCAGGACTCCTCCCTGCAGGACGGCACGCTGATCTACAAGGTGAAGATGCGCGGCACCAACTTCCCCCCCGACGGCCCCGTAATGCAGAAaAAGACCATGGGCTGGGAGGCCTCCACCGAGCGCCTGTACCCCCGCGACGGCGTGCTGAAGGGCGAGATCCACCAGGCCCTGAAGCTGAAGGACGGCGGCCACTACCTGGTGGAGTTCAAGACCATCTACATGGCCAAGAAGCCCGTGCAACTGCCCGGCTACTACTACGTGGACACCAAGCTGGACATCACCTCCCACAACGAGGACTACACCATCGTGGAACAGTACGAGCGCTCCGAGGGCCGCCACCACCTGTTCCTGGGGCATGGCACCGGCAGCACCGGCAGCGGCAGCTCCGGCACCGCCTCCTCCGAGGACAACAACATGGCCGTCATCAAAGAGTTCATGCGCTTCAAGGTGCGCATGGAGGGCTCCATGAACGGCCACGAGTTCGAGATCGAGGGCGAGGGCGAGGGCCGCCCCTACGAGGGCACCCAGACCGCCAAGCTGAAGGTGACCAAGGGCGGCCCCCTGCCCTTCGCCTGGGACATCCTGTCCCCCCAGTTCATGTACGGCTCCAAGGCGTACGTGAAGCACCCCGCCGACATCCCCGATTACAAGAAGCTGTCCTTCCCCGAGGGCTTCAAGTGGGAGCGCGTGATGAACTTCGAGGACGGCGGTCTGGTGACCGTGACCCAGGACTCCTCCCTGCAGGACGGCACGCTGATCTACAAGGTGAAGATGCGCGGCACCAACTTCCCCCCCGACGGCCCCGTAATGCAGAAaAAGACCATGGGCTGGGAGGCCTCCACCGAGCGCCTGTACCCCCGCGACGGCGTGCTGAAGGGCGAGATCCACCAGGCCCTGAAGCTGAAGGACGGCGGCCACTACCTGGTGGAGTTCAAGACCATCTACATGGCCAAGAAGCCCGTGCAACTGCCCGGCTACTACTACGTGGACACCAAGCTGGACATCACCTCCCACAACGAGGACTACACCATCGTGGAACAGTACGAGCGCTCCGAGGGCCGCCACCACCTGTTCCTGTACGGCATgGACGAGCTGTACAAGgaattttaaccctagaaagataatcatattgtgacgtacgttaaagataatcatgcgtaaaattgacgcatgtgttttatcggtctgtatatcgaggtcccgtgaggcgtttgaatagatattaagttttattatatttacacttacatactaataataaattcaacaaacaattcttttacgtgacttttaagatttaactcaaacaaaaactcaaaatttcttctataaagtaacaaaactcaagtttgtacaaaaaagcaggaaCGGCAATAAAAAGACAGAATAAAACGCACGGGTGTTGGGTCGTTTGTTCttctaccgggtaggggaggcgcttttcccaaggcagtctggagcatgcgctttagcagccccgctgggcacttggcgctacacaagtggcctctggcctcgcacacattccacatccaccggtaggcgccaaccggctccgttctttggtggccccttcgcgccaccttctactcctcccctagtcaggaagttcccccccgccccgcagctcgcgtcgtgcaggacgtgacaaatggaagtagcacgtctcactagtctcgtgcagatggacagcaccgctgagcaatggaagcgggtaggcctttggggcagcggccaatagcagctttgctccttcgctttctgggctcagaggctgggaaggggtgggtccgggggcgggctcaggggcgggctcaggggcggggcgggcgcccgaaggtcctccggaggcccggcattctgcacgcttcaaaagcgcacgtctgccgcgctgttctcctcttcctcatctccgggcctttcgaccgatcatcaagcttgatcctcatgaccgagtacaagcccacggtgcgcctcgccacccgcgacgacgtccccagggccgtacgcaccctcgccgccgcgttcgccgactaccccgccacgcgccacaccgtcgatccggaccgccacatcgagcgggtcaccgagctgcaagaactcttcctcacgcgcgtcgggctcgacatcggcaaggtgtgggtcgcggacgacggcgccgcggtggcggtctggaccacgccggagagcgtcgaagcgggggcggtgttcgccgagatcggcccgcgcatggccgagttgagcggttcccggctggccgcgcagcaacagatggaaggcctcctggcgccgcaccggcccaaggagcccgcgtggttcctggccaccgtcggcgtctcgcccgaccaccagggcaagggtctgggcagcgccgtcgtgctccccggagtggaggcggccgagcgcgccggggtgcccgccttcctggagacctccgcgccccgcaacctccccttctacgagcggctcggcttcaccgtcaccgccgacgtcgaggtgcccgaaggaccgcgcacctggtgcatgacccgcaagcccggtgccggatccatgcccacgctactgcgggtttatatagacggtccccacgggatggggaaaaccaccaccacgcaactgctggtggccctgggttcgcgcgacgatatcgtctacgtacccgagccgatgacttactggcgggtgctgggggcttccgagacaatcgcgaacatctacaccacacaacaccgcctcgaccagggtgagatatcggccggggacgcggcggtggtaatgacaagcgcccagataacaatgggcatgccttatgccgtgaccgacgccgttctggctcctcatatcgggggggaggctgggagctcacatgccccgcccccggccctcaccctcatcttcgaccgccatcccatcgccgccctcctgtgctacccggccgcgcggtaccttatgggcagcatgaccccccaggccgtgctggcgttcgtggccctcatcccgccgaccttgcccggcaccaacatcgtgcttggggcccttccggaggacagacacatcgaccgcctggccaaacgccagcgccccggcgagcggctggacctggctatgctggctgcgattcgccgcgtttacgggctacttgccaatacggtgcggtatctgcagtgcggcgggtcgtggcgggaggactggggacagctttcggggacggccgtgccgccccagggtgccgagccccagagcaacgcgggcccacgaccccatatcggggacacgttatttaccctgtttcgggcccccgagttgctggcccccaacggcgacctgtataacgtgtttgcctgggccttggacgtcttggccaaacgcctccgttccatgcacgtctttatcctggattacgaccaatcgcccgccggctgccgggacgccctgctgcaacttacctccgggatggtccagacccacgtcaccacccccggctccataccgacgatatgcgacctggcgcgcacgtttgcccgggagatgggggaggctaactgagctctagcttatcgactcgccgatagtggaaaccgacgccccagcactcgtccgagggcaaaggaatagtcgagaaattgatgatctattaaacaataaagatgtccacatggaagtttttcctgtcatactttgttaagaagggtgagaacagagtacctacattttgaatggaaggattggagctacgggggtgggggtggggtgggattagataaatgcctgctctttactgaaggctctttactattgctttatgataatgtttcatagttggatatcataatttaaacaagcaaaaccaaattaagggccagctcattcctcccactcatgatctatagatcctctagagtcgagggctgcagcagctttcttgtacaaagtgggatatctataacaagaaaatatatatataataagttatcacgtaagtagaacatgaaataacaatataattatcgtatgagttaaatcttaaaagtcacgtaaaagataatcatgcgtcattttgactcacgcggtcgttatagttcaaaatcagtgacacttaccgcattgacaagcacgcctcacgggagctccaagcggcgactgagatgtcctaaatgcacagcgacggattcgcgctatttagaaagagagagcaatatttcaagaatgcatgcgtcaattttacgcagactatctttctagggttaaTAGAAATTTCAACAACTCTTTTTGAATGTATGAAGAGTAGCAGTCCCCTTTaaATGTCCAAGTTATATGTGTCTAGATTTTGATTTCATATATATGTGTATGGGAGGCATGGATATGTTATGAAATCAGCTGGTAATTCCTCCTCATCACGTTTCTCTCATTTTCTTTTGTTTTCCATTGCAAGGGGATGGTTGTTTTCTTTCTGCCTTTAGTTTGCTTTTGCCCAAGGCCCTTAACATTTGGACACTTAAAATAGGGTTAATTTTCAGGGAAAAAGAATGTTGGCGTGTGTAAAGTCTCTATTAGCAATGAAGGGAATTTGTTAACGATGCATCCACTTGATTGATGACTTATTGCAAATGGCGGTTGGCTGAGGAAAACCCATGACACAGCACAACTCTACAGACAGTGATGTGTCTCTTGTTTCTACTGCTAAGAAGGTCTGAAAATTTAATGAAACCACTTCATACATTTAAGTATTTTGTTTGGTTTGAACTCAATCAGTAGCTTTTCCTTACATGTTTAAAAATAATTCCAATGACAGATGAGCAGCTCACTTTTCCAAAGTACCCCAAAAGGCCAAATTAAAAAAGAAAAATAATCACTCTCAAGCCTTGTCTAAGAAAAGAGGCAAACTCTGAAAGTCGTACCAGTTTCTTCTGGAGGCAAAGCAATTTTGCACAAAACCAGCTCTCTCAAGATGAGACTAGAAATTCATACCTGGTCTTGTAGCCACCaacaacttttctatacaaagttgatagcttgatatcaatgactctagaggatccccgggtaccgagctcgaattcgtaatcatggtcatagctgtttcctgtgtgaaattgttatccgctcacaattccacacaacatacgagccggaagcataaagtgtaaagcctggggtgcctaatgagtgagctaactcacattaattgcgttgcgctcactgcccgctttccagtcgggaaacctgtcgtgccagctgcattaatgaatcggccaacgcgcggggagaggcggtttgcgtattgggcgctcttccgcttcctcgctcactgactcgctgcgctcggtcgttcggctgcggcgagcggtatcagctcactcaaaggcggtaatacggttatccacagaatcaggggataacgcaggaaagaacatgtgagcaaaaggccagcaaaaggccaggaaccgtaaaaaggccgcgttgctggcgtttttccataggctccgcccccctgacgagcatcacaaaaatcgacgctcaagtcagaggtggcgaaacccgacaggactataaagataccaggcgtttccccctggaagctccctcgtgcgctctcctgttccgaccctgccgcttaccggatacctgtccgcctttctcccttcgggaagcgtggcgctttctcatagctcacgctgtaggtatctcagttcggtgtaggtcgttcgctccaagctgggctgtgtgcacgaaccccccgttcagcccgaccgctgcgccttatccggtaactatcgtcttgagtccaacccggtaagacacgacttatcgccactggcagcagccactggtaacaggattagcagagcgaggtatgtaggcggtgctacagagttcttgaagtggtggcctaactacggctacactagaaggacagtatttggtatctgcgctctgctgaagccagttaccttcggaaaaagagttggtagctcttgatccggcaaacaaaccaccgctggtagcggtggtttttttgtttgcaagcagcagattacgcgcagaaaaaaaggatctcaagaagatcctttgatcttttctacggggtctgacgctcagtggaacgaaaactcacgttaagggattttggtcatgagattatcaaaaaggatcttcacctagatccttttaaattaaaaatgaagttttaaatcaatctaaagtatatatgagtaaacttggtctgacagttaccaatgcttaatcagtgaggcacctatctcagcgatctgtctatttcgttcatccatagttgcctgactccccgtcgtgtagataactacgatacgggagggcttaccatctggccccagtgctgcaatgataccgcgagacccacgctcaccggctccagatttatcagcaataaaccagccagccggaagggccgagcgcagaagtggtcctgcaactttatccgcctccatccagtctattaattgttgccgggaagctagagtaagtagttcgccagttaatagtttgcgcaacgttgttgccattgctacaggcatcgtggtgtcacgctcgtcgtttggtatggcttcattcagctccggttcccaacgatcaaggcgagttacatgatcccccatgttgtgcaaaaaagcggttagctccttcggtcctccgatcgttgtcagaagtaagttggccgcagtgttatcactcatggttatggcagcactgcataattctcttactgtcatgccatccgtaagatgcttttctgtgactggtgagtactcaaccaagtcattctgagaatagtgtatgcggcgaccgagttgctcttgcccggcgtcaatacgggataataccgcgccacatagcagaactttaaaagtgctcatcattggaaaacgttcttcggggcgaaaactctcaaggatcttaccgctgttgagatccagttcgatgtaacccactcgtgcacccaactgatcttcagcatcttttactttcaccagcgtttctgggtgagcaaaaacaggaaggcaaaatgccgcaaaaaagggaataagggcgacacggaaatgttgaatactcatactcttcctttttcaatattattgaagcatttatcagggttattgtctcatgagcggatacatatttgaatgtatttagaaaaataaacaaataggggttccgcgcacatttccccgaaaagtgccacctgacgtctaagaaaccattattatcatgacattaacctataaaaataggcgtatcacgaggccctttcgtctcgcgcgtttcggtgatgacggtgaaaacctctgacacatgcagctcccggagacggtcacagcttgtctgtaagcggatgccgggagcagacaagcccgtcagggcgcgtcagcgggtgttggcgggtgtcggggctggcttaactatgcggcatcagagcagattgtactgagagtgcaccatatgcggtgtgaaataccgcacagatgcgtaaggagaaaataccgcatcaggcgccattcgccattcaggctgcgcaactgttgggaagggcgatcggtgcgggcctcttcgctattacgccagctggcgaaagggggatgtgctgcaaggcgattaagttgggtaacgccagggtttt

pUC-DEST-cjBLIMP1-Venus-HygTK

cccagtcacgacgttgtaaaacgacggccagtgccaagcttgcatgcctgcaggtcgggatatctgaattatcaactttgtataataaagttgttgccaccatgggcgtctcccttccctgctgtctctctgctctgcactgtaggtctgccacaagcgatttagtagcaccagcaatctcaagacccacctgcgactccattctggagagaaaccgtaccagtgcaaggtgtgccctgccaagttcacccagttcgtgcacctgaaactgcacaagcgcctgcacacccgggagcggccccacaagtgcgcccagtgccacaagaactacatccatctctgtagcctcaaggttcacctgaaggggaactgccctgtagccccagcccctgggctgcccttggaagacctgacccgaatcaatgaagaaattgagaagtttgacatcagcgacaatgccgaccggcttgaggacgtggaggatgacatcagtgtggtctctgtcgtggagaaggaaattctggccatggtcagaaaagagaaagaagaaactggcctgaaagtgtctttgcaaagaaacatggggaatggactcctctcctcagggtgtggcctttacgagtcatcagatctgcccctcatgaagttgcctcccagcaacccgctacctctggtacctgtaaaggtcaaacaagaaacagtcgaaccaatggatcctGCCACGAACTTCTCTCTGTTAAAGCAAGCAGGAGACGTGGAAGAAAACCCCGGTCCTgttAGCAAGGGCGAGGAGCTGTTCACCGGGGTGGTGCCCATCCTGGTCGAGCTGGACGGCGACGTAAACGGCCACAAGTTCAGCGTGTCCGGCGAGGGCGAGGGCGATGCCACCTACGGCAAGCTGACCCTGAAGCTGATCTGCACCACCGGCAAGCTGCCCGTGCCCTGGCCCACCCTCGTGACCACCCTGGGCTACGGCCTGCAGTGCTTCGCCCGCTACCCCGACCACATGAAGCAGCACGACTTCTTCAAGTCCGCCATGCCCGAAGGCTACGTCCAGGAGCGCACCATCTTCTTCAAGGACGACGGCAACTACAAGACCCGCGCCGAGGTGAAGTTCGAGGGCGACACCCTGGTGAACCGCATCGAGCTGAAGGGCATCGACTTCAAGGAGGACGGCAACATCCTGGGGCACAAGCTGGAGTACAACTACAACAGCCACAACGTCTATATCACCGCCGACAAGCAGAAGAACGGCATCAAGGCCAACTTCAAGATCCGCCACAACATCGAGGACGGCGGCGTGCAGCTCGCCGACCACTACCAGCAGAACACCCCCATCGGCGACGGCCCCGTGCTGCTGCCCGACAACCACTACCTGAGCTACCAGTCCAAGCTGAGCAAAGACCCCAACGAGAAGCGCGATCACATGGTCCTGCTGGAGTTCGTGACCGCCGCCGGGATCACTCTCGGCATGGACGAGCTGTACAAGgaattttaaccctagaaagataatcatattgtgacgtacgttaaagataatcatgcgtaaaattgacgcatgtgttttatcggtctgtatatcgaggtcccgtgaggcgtttgaatagatattaagttttattatatttacacttacatactaataataaattcaacaaacaattcttttacgtgacttttaagatttaactcaaacaaaaactcaaaatttcttctataaagtaacaaaactcaagtttgtacaaaaaagcagaattctaccgggtaggggaggcgcttttcccaaggcagtctggagcatgcgctttagcagccccgctgggcacttggcgctacacaagtggcctctggcctcgcacacattccacatccaccggtaggcgccaaccggctccgttctttggtggccccttcgcgccaccttctactcctcccctagtcaggaagttcccccccgccccgcagctcgcgtcgtgcaggacgtgacaaatggaagtagcacgtctcactagtctcgtgcagatggacagcaccgctgagcaatggaagcgggtaggcctttggggcagcggccaatagcagctttgctccttcgctttctgggctcagaggctgggaaggggtgggtccgggggcgggctcaggggcgggctcaggggcggggcgggcgcccgaaggtcctccggaggcccggcattctgcacgcttcaaaagcgcacgtctgccgcgctgttctcctcttcctcatctccgggcctttcgaccgatcatcaagcttgatcctcatgaaaaagcctgaactcaccgcgacgtctgtcgagaagtttctgatcgaaaagttcgacagcgtctccgacctgatgcagctctcggagggcgaagaatctcgtgctttcagcttcgatgtaggagggcGtggatatgtcctgcgggtaaatagctgcgccgatggtttctacaaagatcgttatgtttatcggcactttgcatcggccgcgctcccgattccggaagtgcttgacattggggaattcagcgagagcctgacctattgcatctcccgccgtgcacagggtgtcacgttgcaagacctgcctgaaaccgaactgcccgctgttctgcagccggtcgcggaggccatggatgcgatcgctgcggccgatcttagccagacgagcgggttcggcccattcggaccgcaaggaatcggtcaatacactacatggcgtgatttcatatgcgcgattgctgatccccatgtgtatcactggcaaactgtgatggacgacaccgtcagtgcgtccgtcgcgcaggctctcgatgagctgatgctttgggccgaggactgccccgaagtccggcacctcgtgcacgcggatttcggctccaacaatgtcctgacggacaatggccgcataacagcggtcattgactggagcgaggcgatgttcggggattcccaatacgaggtcgccaacatcttcttctggaggccgtggttggcttgtatggagcagcagacgcgctacttcgagcggaggcatccggagcttgcaggatcgccgcggctccgggcgtatatgctccccattggtcttgaccaactctatcagagcttggttgacggcaatttcgatgatgcagcttgggcgcagggtcgatgcgacgcaatcgtccgatccggagccgggactgtcgggcgtacacaaatcgcccgcagaagcgcggccgtctggaccgatggctgtgtagaagtcgcgtctgcgttcgaccaggctgcgcgttctcgcggccatagcaaccgacgtacggcgttgcgccctcgccggcagcaagaagccacggaagtccgcccggagcagaaaatgcccacgctactgcgggtttatatagacggtccccacgggatggggaaaaccaccaccacgcaactgctggtggccctgggttcgcgcgacgatatcgtctacgtacccgagccgatgacttactggcgggtgctgggggcttccgagacaatcgcgaacatctacaccacacaacaccgcctcgaccagggtgagatatcggccggggacgcggcggtggtaatgacaagcgcccagataacaatgggcatgccttatgccgtgaccgacgccgttctggctcctcatatcgggggggaggctgggagctcacatgccccgcccccggccctcaccctcatcttcgaccgccatcccatcgccgccctcctgtgctacccggccgcgcggtaccttatgggcagcatgaccccccaggccgtgctggcgttcgtggccctcatcccgccgaccttgcccggcaccaacatcgtgcttggggcccttccggaggacagacacatcgaccgcctggccaaacgccagcgccccggcgagcggctggacctggctatgctggctgcgattcgccgcgtttacgggctacttgccaatacggtgcggtatctgcagtgcggcgggtcgtggcgggaggactggggacagctttcggggacggccgtgccgccccagggtgccgagccccagagcaacgcgggcccacgaccccatatcggggacacgttatttaccctgtttcgggcccccgagttgctggcccccaacggcgacctgtataacgtgtttgcctgggccttggacgtcttggccaaacgcctccgttccatgcacgtctttatcctggattacgaccaatcgcccgccggctgccgggacgccctgctgcaacttacctccgggatggtccagacccacgtcaccacccccggctccataccgacgatatgcgacctggcgcgcacgtttgcccgggagatgggggaggctaactgagctctagcttatcgactcgccgatagtggaaaccgacgccccagcactcgtccgagggcaaaggaatagtcgagaaattgatgatctattaaacaataaagatgtccacatggaagtttttcctgtcatactttgttaagaagggtgagaacagagtacctacattttgaatggaaggattggagctacgggggtgggggtggggtgggattagataaatgcctgctctttactgaaggctctttactattgctttatgataatgtttcatagttggatatcataatttaaacaagcaaaaccaaattaagggccagctcattcctcccactcatgatctatagatcctctagagtcgagggctgcagcagctttcttgtacaaagtgggatatctataacaagaaaatatatatataataagttatcacgtaagtagaacatgaaataacaatataattatcgtatgagttaaatcttaaaagtcacgtaaaagataatcatgcgtcattttgactcacgcggtcgttatagttcaaaatcagtgacacttaccgcattgacaagcacgcctcacgggagctccaagcggcgactgagatgtcctaaatgcacagcgacggattcgcgctatttagaaagagagagcaatatttcaagaatgcatgcgtcaattttacgcagactatctttctagggttaataagattttccgaaaacacttgttttgtttcttaagttatgacttggtgagtcagggtgcctgtaggaagttgcttgtacataattccagctctgcaaagctctcttgatagcaaatggtttcccctcacctctggaattaaagaaggaactccaaagttactgaaatctcagggcatgaacaaggcaaaggccatatatatatatacttatttactcccatgtctatatatttgcccctgtgtattttgaatatttgtgtggatatgtttgcatagccttcccattactgagactattgcctagtcataattattttttcaatgataatccttcataatttattatacaatttatcattcagaaagcaataattaaaaaagtttacaataactggaaagattccttgtaatttgagtatcaatgtatttttgtcttgtggccattctttgtagataatttctgcacatctctataagtacctaagatttagttaaacaaatctatgacttcagtcaacctctctctctctaataaacaacttttctatacaaagttgatagcttgatatcaatgactctagaggatccccgggtaccgagctcgaattcgtaatcatggtcatagctgtttcctgtgtgaaattgttatccgctcacaattccacacaacatacgagccggaagcataaagtgtaaagcctggggtgcctaatgagtgagctaactcacattaattgcgttgcgctcactgcccgctttccagtcgggaaacctgtcgtgccagctgcattaatgaatcggccaacgcgcggggagaggcggtttgcgtattgggcgctcttccgcttcctcgctcactgactcgctgcgctcggtcgttcggctgcggcgagcggtatcagctcactcaaaggcggtaatacggttatccacagaatcaggggataacgcaggaaagaacatgtgagcaaaaggccagcaaaaggccaggaaccgtaaaaaggccgcgttgctggcgtttttccataggctccgcccccctgacgagcatcacaaaaatcgacgctcaagtcagaggtggcgaaacccgacaggactataaagataccaggcgtttccccctggaagctccctcgtgcgctctcctgttccgaccctgccgcttaccggatacctgtccgcctttctcccttcgggaagcgtggcgctttctcatagctcacgctgtaggtatctcagttcggtgtaggtcgttcgctccaagctgggctgtgtgcacgaaccccccgttcagcccgaccgctgcgccttatccggtaactatcgtcttgagtccaacccggtaagacacgacttatcgccactggcagcagccactggtaacaggattagcagagcgaggtatgtaggcggtgctacagagttcttgaagtggtggcctaactacggctacactagaaggacagtatttggtatctgcgctctgctgaagccagttaccttcggaaaaagagttggtagctcttgatccggcaaacaaaccaccgctggtagcggtggtttttttgtttgcaagcagcagattacgcgcagaaaaaaaggatctcaagaagatcctttgatcttttctacggggtctgacgctcagtggaacgaaaactcacgttaagggattttggtcatgagattatcaaaaaggatcttcacctagatccttttaaattaaaaatgaagttttaaatcaatctaaagtatatatgagtaaacttggtctgacagttaccaatgcttaatcagtgaggcacctatctcagcgatctgtctatttcgttcatccatagttgcctgactccccgtcgtgtagataactacgatacgggagggcttaccatctggccccagtgctgcaatgataccgcgagacccacgctcaccggctccagatttatcagcaataaaccagccagccggaagggccgagcgcagaagtggtcctgcaactttatccgcctccatccagtctattaattgttgccgggaagctagagtaagtagttcgccagttaatagtttgcgcaacgttgttgccattgctacaggcatcgtggtgtcacgctcgtcgtttggtatggcttcattcagctccggttcccaacgatcaaggcgagttacatgatcccccatgttgtgcaaaaaagcggttagctccttcggtcctccgatcgttgtcagaagtaagttggccgcagtgttatcactcatggttatggcagcactgcataattctcttactgtcatgccatccgtaagatgcttttctgtgactggtgagtactcaaccaagtcattctgagaatagtgtatgcggcgaccgagttgctcttgcccggcgtcaatacgggataataccgcgccacatagcagaactttaaaagtgctcatcattggaaaacgttcttcggggcgaaaactctcaaggatcttaccgctgttgagatccagttcgatgtaacccactcgtgcacccaactgatcttcagcatcttttactttcaccagcgtttctgggtgagcaaaaacaggaaggcaaaatgccgcaaaaaagggaataagggcgacacggaaatgttgaatactcatactcttcctttttcaatattattgaagcatttatcagggttattgtctcatgagcggatacatatttgaatgtatttagaaaaataaacaaataggggttccgcgcacatttccccgaaaagtgccacctgacgtctaagaaaccattattatcatgacattaacctataaaaataggcgtatcacgaggccctttcgtctcgcgcgtttcggtgatgacggtgaaaacctctgacacatgcagctcccggagacggtcacagcttgtctgtaagcggatgccgggagcagacaagcccgtcagggcgcgtcagcgggtgttggcgggtgtcggggctggcttaactatgcggcatcagagcagattgtactgagagtgcaccatatgcggtgtgaaataccgcacagatgcgtaaggagaaaataccgcatcaggcgccattcgccattcaggctgcgcaactgttgggaagggcgatcggtgcgggcctcttcgctattacgccagctggcgaaagggggatgtgctgcaaggcgattaagttgggtaacgccagggtttt

pUC-DEST-cjSTELLA-Cerulean-PuroTK

cccagtcacgacgttgtaaaacgacggccagtgccaagcttgcatgcctgcaggtcgggatatctgaattatcaactttgtataataaagttgttgccaccatgggcttaccctaggggtcactcctaggtgttaggttgatactttttcacatgctgttacaaacatcttcactgtaacctgtgtagtaagagaacaaacacttcagcagcagaatgagttgcatttaaggcattcagggaactgagactagaacgttttattgacaagctgcacttggtaccactacaaagttactctcaggctgccattctgtagtacccatatccccatcctccttttttttcctcctaaaatgtttttcttttggccccatgcccttttttgtgtgttccctgttccaacatttacctaaacaaacattatatttgagggttaattacatttctcttgggtacaatattccaattaattcatttcatcattttttcagaatgcatcaagaagaggaatgttcaaatgtccctgcagtttctgcaagtctagcggatgggatccttctgagaatgccagaatggggaattacgacaccaagccacttcagccaGCCACGAACTTCTCTCTGTTAAAGCAAGCAGGAGACGTGGAAGAAAACCCCGGTCCTgttAGCAAGGGCGAGGAGCTGTTCACCGGGGTGGTGCCCATCCTGGTCGAGCTGGACGGCGACGTAAACGGCCACAAGTTCAGCGTGTCCGGCGAGGGCGAGGGCGATGCCACCTACGGCAAGCTGACCCTGAAGTTCATCTGCACCACCGGCAAGCTGCCCGTGCCCTGGCCCACCCTCGTGACCACCCTGACCTGGGGCGTGCAGTGCTTCGCCCGCTACCCCGACCACATGAAGCAGCACGACTTCTTCAAGTCCGCCATGCCCGAAGGCTACGTCCAGGAGCGCACCATCTTCTTCAAGGACGACGGCAACTACAAGACCCGCGCCGAGGTGAAGTTCGAGGGCGACACCCTGGTGAACCGCATCGAGCTGAAGGGCATCGACTTCAAGGAGGACGGCAACATCCTGGGGCACAAGCTGGAGTACAACGCCATCAGCGACAACGTCTATATCACCGCCGACAAGCAGAAGAACGGCATCAAGGCCAACTTCAAGATCCGCCACAACATCGAGGACGGCAGCGTGCAGCTCGCCGACCACTACCAGCAGAACACCCCCATCGGCGACGGCCCCGTGCTGCTGCCCGACAACCACTACCTGAGCACCCAGTCCAAGCTGAGCAAAGACCCCAACGAGAAGCGCGATCACATGGTCCTGCTGGAGTTCGTGACCGCCGCCGGGATCACTCTCGGCATgGACGAGCTGTACAAGgaattttaaccctagaaagataatcatattgtgacgtacgttaaagataatcatgcgtaaaattgacgcatgtgttttatcggtctgtatatcgaggtcccgtgaggcgtttgaatagatattaagttttattatatttacacttacatactaataataaattcaacaaacaattcttttacgtgacttttaagatttaactcaaacaaaaactcaaaatttcttctataaagtaacaaaactcaagtttgtacaaaaaagcaggaattctaccgggtaggggaggcgcttttcccaaggcagtctggagcatgcgctttagcagccccgctgggcacttggcgctacacaagtggcctctggcctcgcacacattccacatccaccggtaggcgccaaccggctccgttctttggtggccccttcgcgccaccttctactcctcccctagtcaggaagttcccccccgccccgcagctcgcgtcgtgcaggacgtgacaaatggaagtagcacgtctcactagtctcgtgcagatggacagcaccgctgagcaatggaagcgggtaggcctttggggcagcggccaatagcagctttgctccttcgctttctgggctcagaggctgggaaggggtgggtccgggggcgggctcaggggcgggctcaggggcggggcgggcgcccgaaggtcctccggaggcccggcattctgcacgcttcaaaagcgcacgtctgccgcgctgttctcctcttcctcatctccgggcctttcgaccgatcatcaagcttgatcctcatgaccgagtacaagcccacggtgcgcctcgccacccgcgacgacgtccccagggccgtacgcaccctcgccgccgcgttcgccgactaccccgccacgcgccacaccgtcgatccggaccgccacatcgagcgggtcaccgagctgcaagaactcttcctcacgcgcgtcgggctcgacatcggcaaggtgtgggtcgcggacgacggcgccgcggtggcggtctggaccacgccggagagcgtcgaagcgggggcggtgttcgccgagatcggcccgcgcatggccgagttgagcggttcccggctggccgcgcagcaacagatggaaggcctcctggcgccgcaccggcccaaggagcccgcgtggttcctggccaccgtcggcgtctcgcccgaccaccagggcaagggtctgggcagcgccgtcgtgctccccggagtggaggcggccgagcgcgccggggtgcccgccttcctggagacctccgcgccccgcaacctccccttctacgagcggctcggcttcaccgtcaccgccgacgtcgaggtgcccgaaggaccgcgcacctggtgcatgacccgcaagcccggtgccggatccatgcccacgctactgcgggtttatatagacggtccccacgggatggggaaaaccaccaccacgcaactgctggtggccctgggttcgcgcgacgatatcgtctacgtacccgagccgatgacttactggcgggtgctgggggcttccgagacaatcgcgaacatctacaccacacaacaccgcctcgaccagggtgagatatcggccggggacgcggcggtggtaatgacaagcgcccagataacaatgggcatgccttatgccgtgaccgacgccgttctggctcctcatatcgggggggaggctgggagctcacatgccccgcccccggccctcaccctcatcttcgaccgccatcccatcgccgccctcctgtgctacccggccgcgcggtaccttatgggcagcatgaccccccaggccgtgctggcgttcgtggccctcatcccgccgaccttgcccggcaccaacatcgtgcttggggcccttccggaggacagacacatcgaccgcctggccaaacgccagcgccccggcgagcggctggacctggctatgctggctgcgattcgccgcgtttacgggctacttgccaatacggtgcggtatctgcagtgcggcgggtcgtggcgggaggactggggacagctttcggggacggccgtgccgccccagggtgccgagccccagagcaacgcgggcccacgaccccatatcggggacacgttatttaccctgtttcgggcccccgagttgctggcccccaacggcgacctgtataacgtgtttgcctgggccttggacgtcttggccaaacgcctccgttccatgcacgtctttatcctggattacgaccaatcgcccgccggctgccgggacgccctgctgcaacttacctccgggatggtccagacccacgtcaccacccccggctccataccgacgatatgcgacctggcgcgcacgtttgcccgggagatgggggaggctaactgagctctagcttatcgactcgccgatagtggaaaccgacgccccagcactcgtccgagggcaaaggaatagtcgagaaattgatgatctattaaacaataaagatgtccacatggaagtttttcctgtcatactttgttaagaagggtgagaacagagtacctacattttgaatggaaggattggagctacgggggtgggggtggggtgggattagataaatgcctgctctttactgaaggctctttactattgctttatgataatgtttcatagttggatatcataatttaaacaagcaaaaccaaattaagggccagctcattcctcccactcatgatctatagatcctctagagtcgagggctgcagcagctttcttgtacaaagtgggatatctataacaagaaaatatatatataataagttatcacgtaagtagaacatgaaataacaatataattatcgtatgagttaaatcttaaaagtcacgtaaaagataatcatgcgtcattttgactcacgcggtcgttatagttcaaaatcagtgacacttaccgcattgacaagcacgcctcacgggagctccaagcggcgactgagatgtcctaaatgcacagcgacggattcgcgctatttagaaagagagagcaatatttcaagaatgcatgcgtcaattttacgcagactatctttctagggttaataaaccttattcttgccccttttttttcttgctggtaattttttatagcagaccgagaaagctactctctgctcatataaattatatatcaataattttgataaacgagttcaaggttgtatttttcttgtatctttttccctactatgatgctagtaatttataagggatctgtgtagtttgaatatatttggataactttagctgtactgtttggtttgtcccaaagaagccaagagcatgtaaatattcccatgtgtttcagaagcccaaagtcagtgaggtgaaacccagcaacaagacactgaagcaaagatacttgtgaataaagaaagcattagctagtttggttagagcataataattagattttctggctttcaaaaatttgggttgcggtaagagcaaagtttgtgctatttttacagttttcagtacaaaaaggtgttaatatagaaacaataaagttgacattagattaccttttaaaaatagtttttgattttatagttgttcttgctaacaattaggtgaacaatagtaacaacttttctatacaaagttgatagcttgatatcaatgactctagaggatccccgggtaccgagctcgaattcgtaatcatggtcatagctgtttcctgtgtgaaattgttatccgctcacaattccacacaacatacgagccggaagcataaagtgtaaagcctggggtgcctaatgagtgagctaactcacattaattgcgttgcgctcactgcccgctttccagtcgggaaacctgtcgtgccagctgcattaatgaatcggccaacgcgcggggagaggcggtttgcgtattgggcgctcttccgcttcctcgctcactgactcgctgcgctcggtcgttcggctgcggcgagcggtatcagctcactcaaaggcggtaatacggttatccacagaatcaggggataacgcaggaaagaacatgtgagcaaaaggccagcaaaaggccaggaaccgtaaaaaggccgcgttgctggcgtttttccataggctccgcccccctgacgagcatcacaaaaatcgacgctcaagtcagaggtggcgaaacccgacaggactataaagataccaggcgtttccccctggaagctccctcgtgcgctctcctgttccgaccctgccgcttaccggatacctgtccgcctttctcccttcgggaagcgtggcgctttctcatagctcacgctgtaggtatctcagttcggtgtaggtcgttcgctccaagctgggctgtgtgcacgaaccccccgttcagcccgaccgctgcgccttatccggtaactatcgtcttgagtccaacccggtaagacacgacttatcgccactggcagcagccactggtaacaggattagcagagcgaggtatgtaggcggtgctacagagttcttgaagtggtggcctaactacggctacactagaaggacagtatttggtatctgcgctctgctgaagccagttaccttcggaaaaagagttggtagctcttgatccggcaaacaaaccaccgctggtagcggtggtttttttgtttgcaagcagcagattacgcgcagaaaaaaaggatctcaagaagatcctttgatcttttctacggggtctgacgctcagtggaacgaaaactcacgttaagggattttggtcatgagattatcaaaaaggatcttcacctagatccttttaaattaaaaatgaagttttaaatcaatctaaagtatatatgagtaaacttggtctgacagttaccaatgcttaatcagtgaggcacctatctcagcgatctgtctatttcgttcatccatagttgcctgactccccgtcgtgtagataactacgatacgggagggcttaccatctggccccagtgctgcaatgataccgcgagacccacgctcaccggctccagatttatcagcaataaaccagccagccggaagggccgagcgcagaagtggtcctgcaactttatccgcctccatccagtctattaattgttgccgggaagctagagtaagtagttcgccagttaatagtttgcgcaacgttgttgccattgctacaggcatcgtggtgtcacgctcgtcgtttggtatggcttcattcagctccggttcccaacgatcaaggcgagttacatgatcccccatgttgtgcaaaaaagcggttagctccttcggtcctccgatcgttgtcagaagtaagttggccgcagtgttatcactcatggttatggcagcactgcataattctcttactgtcatgccatccgtaagatgcttttctgtgactggtgagtactcaaccaagtcattctgagaatagtgtatgcggcgaccgagttgctcttgcccggcgtcaatacgggataataccgcgccacatagcagaactttaaaagtgctcatcattggaaaacgttcttcggggcgaaaactctcaaggatcttaccgctgttgagatccagttcgatgtaacccactcgtgcacccaactgatcttcagcatcttttactttcaccagcgtttctgggtgagcaaaaacaggaaggcaaaatgccgcaaaaaagggaataagggcgacacggaaatgttgaatactcatactcttcctttttcaatattattgaagcatttatcagggttattgtctcatgagcggatacatatttgaatgtatttagaaaaataaacaaataggggttccgcgcacatttccccgaaaagtgccacctgacgtctaagaaaccattattatcatgacattaacctataaaaataggcgtatcacgaggccctttcgtctcgcgcgtttcggtgatgacggtgaaaacctctgacacatgcagctcccggagacggtcacagcttgtctgtaagcggatgccgggagcagacaagcccgtcagggcgcgtcagcgggtgttggcgggtgtcggggctggcttaactatgcggcatcagagcagattgtactgagagtgcaccatatgcggtgtgaaataccgcacagatgcgtaaggagaaaataccgcatcaggcgccattcgccattcaggctgcgcaactgttgggaagggcgatcggtgcgggcctcttcgctattacgccagctggcgaaagggggatgtgctgcaaggcgattaagttgggtaacgccagggtttt

pPB-VASA-tdTomato-pNeo

ctaaattgtaagcgttaatattttgttaaaattcgcgttaaatttttgttaaatcagctcattttttaaccaataggccgaaatcggcaaaatcccttataaatcaaaagaatagaccgagatagggttgagtgttgttccagtttggaacaagagtccactattaaagaacgtggactccaacgtcaaagggcgaaaaaccgtctatcagggcgatggcccactacgtgaaccatcaccctaatcaagttttttggggtcgaggtgccgtaaagcactaaatcggaaccctaaagggagcccccgatttagagcttgacggggaaagccggcgaacgtggcgagaaaggaagggaagaaagcgaaaggagcgggcgctagggcgctggcaagtgtagcggtcacgctgcgcgtaaccaccacacccgccgcgcttaatgcgccgctacagggcgcgtcccattcgccattcaggctgcgcaactgttgggaagggcgatcggtgcgggcctcttcgctattacgccagctggcgaaagggggatgtgctgcaaggcgattaagttgggtaacgccagggttttcccagtcacgacgttgtaaaacgacggccagtgaattgtaatacgactcactatagggcgaattgggtaccttaaccctagaaagatagtctgcgtaaaattgacgcatgcattcttgaaatattgctctctctttctaaatagcgcgaatccgtcgctgtgcatttaggacatctcagtcgccgcttggagctcccgtgaggcgtgcttgtcaatgcggtaagtgtcactgattttgaactataacgaccgcgtgagtcaaaatgacgcatgattatcttttacgtgacttttaagatttaactcatacgataattatattgttatttcatgttctacttacgtgataacttattatatatatattttcttgttatagatgatccactagtaacggccgccagtgtgctggaattcgcggccgcCttgaggccagaaattggcgaccagcctgggcaacacagggagactcttgggaggctgaggcaggaggctcacttgaggcccggaattggagaccagcctggcaacacagggagactctttctctacaaaattttttttaaaaattagctgggcatggtggcttgtgcctgtagtcccagctattcgggaggctgaggtggcaggatcacttgagcacagaaggttgaggctgcagtaagctatgatcattagtgcactcccctctgggtgacagagtgagaccttgtctcaaaaataaataaataaataaataaaaaatatattcattttgatcatcttgagaatagtctgacaaagtttattgctaactagatgaatcctgaaattcttggtggtgatggttctagagataaaaagtatgaagcaagcctgagcctattaagtgctttatcctttatgtaccagtatcaaactgtgttgctatttatgttcctgaatgagataggaatttcttcagattcagagaagggaaaatgacagaaacgacaacaaaaatgagaactacactttttttttttttttttttttttgaggcggagtctcggtctgaggcccaggctggagtgcagtcgcgcgatctcggctcactgcaagctccgtctcccgggttcacgccattctcctgcctcaacctctggagtagctgggactacaggcgcccgccaccacgcccggctaattttttgtatttttagtagagacggggtttcgccgtgttagccaggatggttgcatctcctgacctcgtgatccgcccgcttcggcctccccaagtgctgggattacagacagacgtaagccaccgcgcccggcctctgagaactacacttttaaaacagcccatggtcacgcgttttgaagggctggggaaatactgagggattttacctgcttttggggtggggggtgggggcagaacaagaaaatctgattgaagacagcatgaagtatcaaaataacccttggccactttggctagtgcccgtaggaatataaacaggtgggccacaaatccaagccttgatgaatgacctatgccagcaccaatttcctccctccttctggatcagagcctgtttccttccaaagtggtggacccccgaaatcttacaggcttttttttttttttttttttcctctgtctcactctgtcgcccaggtagagatcaatggcatgatctcggttcattgcaacctctgcctcccgggttcaagcggttctcttgcctcagcctcctgagtagctgggactacaggcagccgccaccatacccggctaatttttttaattttagtagagacggggtttcaccatgttggctaggctggtctcgaactcctagcctcatgtgatccgcccgcctcggcctcccaaagtgctgggattacaggcgtgagtcaccatgcccagccgagtctaactttctttacgatttcctggggtcccagctggacggagtctcatccctgccccccttactgggaggaaaagggaatgcacctcctccatgagcctcagctgcactttgaggcacctggaatccccggatgtggagcgagtctctgaaatgcagaacccgaggggccaacccatggactgtgatgggtaaaaggggagagagcactaaagaggccaagtagggaggacattatccctttccccttttattattattttttgttagaaaaaggcaggtggacatatttagcttgcgaagcttgcacaaggaaaatccagagcgtctccagaataaggatcccacgagagaacgtggacacaagtaggccctcagccagccttgtacccacaggcccaacaggccacttggctatgaggccagagcgtcgccataggggcccgaacgctagcgtttagggaatccgcaggctagaagtggaggcgggacgccactggtcgtctgagcgctgattggctggtggcgctagtcaccagccaatcgtcaacagacgccatttgttgttggagccacgcacctgacgcagtgggcgtcttgcacgtgcagccgtttaagtcgcgtgggcgcctgttatggcttgggagagcaagccgcggagaggtgagtgggccgcttttctacgggaactggcctgaactcggccgtacgggctccccagtaaccgacctttggccaaagtgtggatggagtaggaggagttttgaagccgtccttcgaagcctttctgcctcggggtctcagagtcccacgggtgcccaaagtctgggcttgaggcctgcagaggaggcgaaggggccttattcctggcttcttcactgcgttttctctgaacagacatggtccgagtaccatttgtgtacaaatccttcttgatgaagctggttgtcctttgtcggcaggatttgggcaaccatacccttcttcccttccccactaaaatccaaggcctccctcttcaccgagtcgccgcccccctacccagtcgactcccgtacccctgtccccgcccccacgcacgccacaaacaccccaaagcaaacatcctatccaccttggatgttaatcatgcgaagggagaaatcttaagtagtttctgaaaaaagttgcctctcccttaatattcagtaagtcgtttggggaccctttgagggaggagataaagtctatgaacagatgtttgggaaaagcctaagtcttgctggggacatggcgattgctagaatgactaggtaagaggttcaactagtttctaaagctaagaaaggatggagataaatgccacctttagataagaagagacctaattccttctttggaaaaaacttttggggtatcagcacctagttgggtagtttcaagatgtatgtgaaacaatgagtttatgcttttaaagttatgattctgatctaattgagtccaaatgtgcatttttttttttttttatgaatagaacttgaagcACCCTCGAGaccATGGTGAGCAAGGGCGAGGAGGTCATCAAAGAGTTCATGCGCTTCAAGGTGCGCATGGAGGGCTCCATGAACGGCCACGAGTTCGAGATCGAGGGCGAGGGCGAGGGCCGCCCCTACGAGGGCACCCAGACCGCCAAGCTGAAGGTGACCAAGGGCGGCCCCCTGCCCTTCGCCTGGGACATCCTGTCCCCCCAGTTCATGTACGGCTCCAAGGCGTACGTGAAGCACCCCGCCGACATCCCCGATTACAAGAAGCTGTCCTTCCCCGAGGGCTTCAAGTGGGAGCGCGTGATGAACTTCGAGGACGGCGGTCTGGTGACCGTGACCCAGGACTCCTCCCTGCAGGACGGCACGCTGATCTACAAGGTGAAGATGCGCGGCACCAACTTCCCCCCCGACGGCCCCGTAATGCAGAAGAAGACCATGGGCTGGGAGGCCTCCACCGAGCGCCTGTACCCCCGCGACGGCGTGCTGAAGGGCGAGATCCACCAGGCCCTGAAGCTGAAGGACGGCGGCCACTACCTGGTGGAGTTCAAGACCATCTACATGGCCAAGAAGCCCGTGCAACTGCCCGGCTACTACTACGTGGACACCAAGCTGGACATCACCTCCCACAACGAGGACTACACCATCGTGGAACAGTACGAGCGCTCCGAGGGCCGCCACCACCTGTTCCTGGGGCATGGCACCGGCAGCACCGGCAGCGGCAGCTCCGGCACCGCCTCCTCCGAGGACAACAACATGGCCGTCATCAAAGAGTTCATGCGCTTCAAGGTGCGCATGGAGGGCTCCATGAACGGCCACGAGTTCGAGATCGAGGGCGAGGGCGAGGGCCGCCCCTACGAGGGCACCCAGACCGCCAAGCTGAAGGTGACCAAGGGCGGCCCCCTGCCCTTCGCCTGGGACATCCTGTCCCCCCAGTTCATGTACGGCTCCAAGGCGTACGTGAAGCACCCCGCCGACATCCCCGATTACAAGAAGCTGTCCTTCCCCGAGGGCTTCAAGTGGGAGCGCGTGATGAACTTCGAGGACGGCGGTCTGGTGACCGTGACCCAGGACTCCTCCCTGCAGGACGGCACGCTGATCTACAAGGTGAAGATGCGCGGCACCAACTTCCCCCCCGACGGCCCCGTAATGCAGAAGAAGACCATGGGCTGGGAGGCCTCCACCGAGCGCCTGTACCCCCGCGACGGCGTGCTGAAGGGCGAGATCCACCAGGCCCTGAAGCTGAAGGACGGCGGCCACTACCTGGTGGAGTTCAAGACCATCTACATGGCCAAGAAGCCCGTGCAACTGCCCGGCTACTACTACGTGGACACCAAGCTGGACATCACCTCCCACAACGAGGACTACACCATCGTGGAACAGTACGAGCGCTCCGAGGGCCGCCACCACCTGTTCCTGTACGGCATGGACGAGCTGTACAAGTAGgtcgacctgtgccttctagttgccagccatctgttgtttgcccctcccccgtgccttccttgaccctggaaggtgccactcccactgtcctttcctaataaaatgaggaaattgcatcgcattgtctgagtaggtgtcattctattctggggggtggggtggggcaggacagcaagggggaggattgggaagacaatagcaggcatgctggggatgcggtgggctctatggaagctttaatgcggtagtttatcacagttaaattgctaacgcagtcaggcaccgtgtatgaaatctaacaatgcgctcatcgtcatcctcggcaccgtcaccctggatgctgtaggcataggcttggttatgccggtactgccgggcctcttgcggggaattctaccgggtaggggaggcgcttttcccaaggcagtctggagcatgcgctttagcagccccgctgggcacttggcgctacacaagtggcctctggcctcgcacacattccacatccaccggtaggcgccaaccggctccgttctttggtggccccttcgcgccaccttctactcctcccctagtcaggaagttcccccccgccccgcagctcgcgtcgtgcaggacgtgacaaatggaagtagcacgtctcactagtctcgtgcagatggacagcaccgctgagcaatggaagcgggtaggcctttggggcagcggccaatagcagctttgctccttcgctttctgggctcagaggctgggaaggggtgggtccgggggcgggctcaggggcgggctcaggggcggggcgggcgcccgaaggtcctccggaggcccggcattctgcacgcttcaaaagcgcacgtctgccgcgctgttctcctcttcctcatctccgggcctttcgacctgcagccaatatgggatcggccattgaacaagatggattgcacgcaggttctccggccgcttgggtggagaggctattcggctatgactgggcacaacagacaatcggctgctctgatgccgccgtgttccggctgtcagcgcaggggcgcccggttctttttgtcaagaccgacctgtccggtgccctgaatgaactgcaggacgaggcagcgcggctatcgtggctggccacgacgggcgttccttgcgcagctgtgctcgacgttgtcactgaagcgggaagggactggctgctattgggcgaagtgccggggcaggatctcctgtcatctcaccttgctcctgccgagaaagtatccatcatggctgatgcaatgcggcggctgcatacgcttgatccggctacctgcccattcgaccaccaagcgaaacatcgcatcgagcgagcacgtactcggatggaagccggtcttgtcgatcaggatgatctggacgaagagcatcaggggctcgcgccagccgaactgttcgccaggctcaaggcgcgcatgcccgacggcgatgatctcgtcgtgacccatggcgatgcctgcttgccgaatatcatggtggaaaatggccgcttttctggattcatcgactgtggccggctgggtgtggcggaccgctatcaggacatagcgttggctacccgtgatattgctgaagagcttggcggcgaatgggctgaccgcttcctcgtgctttacggtatcgccgctcccgattcgcagcgcatcgccttctatcgccttcttgacgagttcttctgaggggatcgatccgctgtaagtctgcagaaattgatgatctattaaacaataaagatgtccactaaaatggaagtttttcctgtcatactttgttaagaagggtgagaacagagtacctacattttgaatggaaggattggagctacgggggtgggggtggggtgggattagataaatgcctgctctttactgaaggctctttactattgctttatgataatgtttcatagttggatatcagatcctttgttactttatagaagaaattttgagtttttgtttttttttaataaataaataaacataaataaattgtttgttgaatttattattagtatgtaagtgtaaatataataaaacttaatatctattcaaattaataaataaacctcgatatacagaccgataaaacacatgcgtcaattttacgcatgattatctttaacgtacgtcacaatatgattatctttctagggttaaagagctccagcttttgttccctttagtgagggttaatttcgagcttggcgtaatcatggtcatagctgtttcctgtgtgaaattgttatccgctcacaattccacacaacatacgagccggaagcataaagtgtaaagcctggggtgcctaatgagtgagctaactcacattaattgcgttgcgctcactgcccgctttccagtcgggaaacctgtcgtgccagctgcattaatgaatcggccaacgcgcggggagaggcggtttgcgtattgggcgctcttccgcttcctcgctcactgactcgctgcgctcggtcgttcggctgcggcgagcggtatcagctcactcaaaggcggtaatacggttatccacagaatcaggggataacgcaggaaagaacatgtgagcaaaaggccagcaaaaggccaggaaccgtaaaaaggccgcgttgctggcgtttttccataggctccgcccccctgacgagcatcacaaaaatcgacgctcaagtcagaggtggcgaaacccgacaggactataaagataccaggcgtttccccctggaagctccctcgtgcgctctcctgttccgaccctgccgcttaccggatacctgtccgcctttctcccttcgggaagcgtggcgctttctcatagctcacgctgtaggtatctcagttcggtgtaggtcgttcgctccaagctgggctgtgtgcacgaaccccccgttcagcccgaccgctgcgccttatccggtaactatcgtcttgagtccaacccggtaagacacgacttatcgccactggcagcagccactggtaacaggattagcagagcgaggtatgtaggcggtgctacagagttcttgaagtggtggcctaactacggctacactagaaggacagtatttggtatctgcgctctgctgaagccagttaccttcggaaaaagagttggtagctcttgatccggcaaacaaaccaccgctggtagcggtggtttttttgtttgcaagcagcagattacgcgcagaaaaaaaggatctcaagaagatcctttgatcttttctacggggtctgacgctcagtggaacgaaaactcacgttaagggattttggtcatgagattatcaaaaaggatcttcacctagatccttttaaattaaaaatgaagttttaaatcaatctaaagtatatatgagtaaacttggtctgacagttaccaatgcttaatcagtgaggcacctatctcagcgatctgtctatttcgttcatccatagttgcctgactccccgtcgtgtagataactacgatacgggagggcttaccatctggccccagtgctgcaatgataccgcgagacccacgctcaccggctccagatttatcagcaataaaccagccagccggaagggccgagcgcagaagtggtcctgcaactttatccgcctccatccagtctattaattgttgccgggaagctagagtaagtagttcgccagttaatagtttgcgcaacgttgttgccattgctacaggcatcgtggtgtcacgctcgtcgtttggtatggcttcattcagctccggttcccaacgatcaaggcgagttacatgatcccccatgttgtgcaaaaaagcggttagctccttcggtcctccgatcgttgtcagaagtaagttggccgcagtgttatcactcatggttatggcagcactgcataattctcttactgtcatgccatccgtaagatgcttttctgtgactggtgagtactcaaccaagtcattctgagaatagtgtatgcggcgaccgagttgctcttgcccggcgtcaatacgggataataccgcgccacatagcagaactttaaaagtgctcatcattggaaaacgttcttcggggcgaaaactctcaaggatcttaccgctgttgagatccagttcgatgtaacccactcgtgcacccaactgatcttcagcatcttttactttcaccagcgtttctgggtgagcaaaaacaggaaggcaaaatgccgcaaaaaagggaataagggcgacacggaaatgttgaatactcatactcttcctttttcaatattattgaagcatttatcagggttattgtctcatgagcggatacatatttgaatgtatttagaaaaataaacaaataggggttccgcgcacatttccccgaaaagtgccac

pENTR-L1-U6-BbsI-R5

tatacaaaagttgaacgagaaacgtaaaatgatataaatatcaatatattaaattagattttgcataaaaaacagactacataatactgtaaaacacaacatatccagtcactatgaatcaactacttagatggtattagtgacctgtactgcagctctggcccgtgtctcaaaatctctgatgttacattgcacaagataaaaatatatcatcatgaacaataaaactgtctgcttacataaacagtaatacaaggggtgttatgagccatattcaacgggaaacgtcgaggccgcgattaaattccaacatggatgctgatttatatgggtataaatgggctcgcgataatgtcgggcaatcaggtgcgacaatctatcgcttgtatgggaagcccgatgcgccagagttgtttctgaaacatggcaaaggtagcgttgccaatgatgttacagatgagatggtcagactaaactggctgacggaatttatgcctcttccgaccatcaagcattttatccgtactcctgatgatgcatggttactcaccactgcgatccccggaaaaacagcattccaggtattagaagaatatcctgattcaggtgaaaatattgttgatgcgctggcagtgttcctgcgccggttgcattcgattcctgtttgtaattgtccttttaacagcgatcgcgtatttcgtctcgctcaggcgcaatcacgaatgaataacggtttggttgatgcgagtgattttgatgacgagcgtaatggctggcctgttgaacaagtctggaaagaaatgcataaacttttgccattctcaccggattcagtcgtcactcatggtgatttctcacttgataaccttatttttgacgaggggaaattaataggttgtattgatgttggacgagtcggaatcgcagaccgataccaggatcttgccatcctatggaactgcctcggtgagttttctccttcattacagaaacggctttttcaaaaatatggtattgataatcctgatatgaataaattgcagtttcatttgatgctcgatgagtttttctaatcagaattggttaattggttgtaacactggcagagcattacgctgacttgacgggacggcgcaagctcatgaccaaaatcccttaacgtgagttttcgttccactgagcgtcagaccccgtagaaaagatcaaaggatcttcttgagatcctttttttctgcgcgtaatctgctgcttgcaaacaaaaaaaccaccgctaccagcggtggtttgtttgccggatcaagagctaccaactctttttccgaaggtaactggcttcagcagagcgcagataccaaatactgtccttctagtgtagccgtagttaggccaccacttcaagaactctgtagcaccgcctacatacctcgctctgctaatcctgttaccagtggctgctgccagtggcgataagtcgtgtcttaccgggttggactcaagacgatagttaccggataaggcgcagcggtcgggctgaacggggggttcgtgcacacagcccagcttggagcgaacgacctacaccgaactgagatacctacagcgtgagctatgagaaagcgccacgcttcccgaagggagaaaggcggacaggtatccggtaagcggcagggtcggaacaggagagcgcacgagggagcttccagggggaaacgcctggtatctttatagtcctgtcgggtttcgccacctctgacttgagcgtcgatttttgtgatgctcgtcaggggggcggagcctatggaaaaacgccagcaacgcggcctttttacggttcctggccttttgctggccttttgctcacatgttctttcctgcgttatcccctgattctgtggataaccgtattaccgctagcatggatctcgggccccaaataatgattttattttgactgatagtgacctgttcgttgcaacaaattgatgagcaatgcttttttataatgccaactttgtacaaaaaagcagagggcctatttcccatgattccttcatatttgcatatacgatacaaggctgttagagagataattggaattaatttgactgtaaacacaaagatattagtacaaaatacgtgacgtagaaagtaataatttcttgggtagtttgcagttttaaaattatgttttaaaatggactatcatatgcttaccgtaacttgaaagtatttcgatttcttggctttatatatcttgtggaaaggacgaaacaccgggtcttcgagaagacctgttttagagctagaaatagcaagttaaaataaggctagtccgttatcaacttgaaaaagtggcaccgagtcggtgcttttttgttttagagctagaaatagcaagttaaaataaggctagtccgtttttagcgcgtgcgccaattctgcagacaaatggctctagaggtacccaactttg

pENTR-L1-U6-BsmBI-R5

ctagcatggatctcgggccccaaataatgattttattttgactgatagtgacctgttcgttgcaacaaattgatgagcaatgcttttttataatgccaactttgtacaaaaaagcagtcgactggatccggtaccaaggtcgggcaggaagagggcctatttcccatgattccttcatatttgcatatacgatacaaggctgttagagagataattagaattaatttgactgtaaacacaaagatattagtacaaaatacgtgacgtagaaagtaataatttcttgggtagtttgcagttttaaaattatgttttaaaatggactatcatatgcttaccgtaacttgaaagtatttcgatttcttggctttatatatcttgtggaaaggacgaaacaccgttcactgccgtataggcagtgagacgattaatgcgtctcagttttagagctagaaatagcaagttaaaataaggctagtccgttatcaacttgaaaaagtggcaccgagtcggtgcgttcactgccgtataggcagtttttttaagcttgggccgctcgaggtaccaactttgtatacaaaagttgaacgagaaacgtaaaatgatataaatatcaatatattaaattagattttgcataaaaaacagactacataatactgtaaaacacaacatatccagtcactatgaatcaactacttagatggtattagtgacctgtactgcagctctggcccgtgtctcaaaatctctgatgttacattgcacaagataaaaatatatcatcatgaacaataaaactgtctgcttacataaacagtaatacaaggggtgttatgagccatattcaacgggaaacgtcgaggccgcgattaaattccaacatggatgctgatttatatgggtataaatgggctcgcgataatgtcgggcaatcaggtgcgacaatctatcgcttgtatgggaagcccgatgcgccagagttgtttctgaaacatggcaaaggtagcgttgccaatgatgttacagatgagatggtcagactaaactggctgacggaatttatgcctcttccgaccatcaagcattttatccgtactcctgatgatgcatggttactcaccactgcgatccccggaaaaacagcattccaggtattagaagaatatcctgattcaggtgaaaatattgttgatgcgctggcagtgttcctgcgccggttgcattcgattcctgtttgtaattgtccttttaacagcgatcgcgtatttcgtcttgctcaggcgcaatcacgaatgaataacggtttggttgatgcgagtgattttgatgacgagcgtaatggctggcctgttgaacaagtctggaaagaaatgcataaacttttgccattctcaccggattcagtcgtcactcatggtgatttctcacttgataaccttatttttgacgaggggaaattaataggttgtattgatgttggacgagtcggaatcgcagaccgataccaggatcttgccatcctatggaactgcctcggtgagttttctccttcattacagaaacggctttttcaaaaatatggtattgataatcctgatatgaataaattgcagtttcatttgatgctcgatgagtttttctaatcagaattggttaattggttgtaacactggcagagcattacgctgacttgacgggacggcgcaagctcatgaccaaaatcccttaacgtgagttttcgttccactgagcgtcagaccccgtagaaaagatcaaaggatcttcttgagatcctttttttctgcgcgtaatctgctgcttgcaaacaaaaaaaccaccgctaccagcggtggtttgtttgccggatcaagagctaccaactctttttccgaaggtaactggcttcagcagagcgcagataccaaatactgtccttctagtgtagccgtagttaggccaccacttcaagaactctgtagcaccgcctacatacctcgctctgctaatcctgttaccagtggctgctgccagtggcgataagtcgtgtcttaccgggttggactcaagacgatagttaccggataaggcgcagcggtcgggctgaacggggggttcgtgcacacagcccagcttggagcgaacgacctacaccgaactgagatacctacagcgtgagctatgagaaagcgccacgcttcccgaagggagaaaggcggacaggtatccggtaagcggcagggtcggaacaggagagcgcacgagggagcttccagggggaaacgcctggtatctttatagtcctgtcgggtttcgccacctctgacttgagcgtcgatttttgtgatgctcgtcaggggggcggagcctatggaaaaacgccagcaacgcggcctttttacggttcctggccttttgctggccttttgctcacatgttctttcctgcgttatcccctgattctgtggataaccgtattaccg

pENTR-R4-H1-BbsI-L2

ctttcctgcgttatcccctgattctgtggataaccgtattaccgtatacacagccagtctgcaggtcgaccatagtgactggatatgttgtgttttacagtattatgtagtctgttttttatgcaaaatctaatttaatatattgatatttatatcattttacgtttctcgttcaacttttctatacaaagttgatagcttgatatcaatactctgaattcgaacgctgacgtcatcaacccgctccaaggaatcgcgggcccagtgtcactaggcgggaacacccagcgcgcgtgcgccctggcaggaagatggctgtgagggacaggggagtggcgccctgcaatatttgcatgtcgctatgtgttctgggaaatcaccataaacgtgaaatgtctttggatttgggaatcttataagttctgtatgaggaccacagatcaccgggtcttcgagaagacctgttttagagctagaaatagcaagttaaaataaggctagtccgttatcaacttgaaaaagtggcaccgagtcggtgcttttttgatatctagacccagctttcttgtacaaagttggcattataagaaagcattgcttatcaatttgttgcaacgaacaggtcactatcagtcaaaataaaatcattatttgccatccagctgcagctctggcccgtgtctcaaaatctctgatgttacattgcacaagataaaaatatatcatcatgaacaataaaactgtctgcttacataaacagtaatacaaggggtgttatgagccatattcaacgggaaacgtcgaggccgcgattaaattccaacatggatgctgatttatatgggtataaatgggctcgcgataatgtcgggcaatcaggtgcgacaatctatcgcttgtatgggaagcccgatgcgccagagttgtttctgaaacatggcaaaggtagcgttgccaatgatgttacagatgagatggtcagactaaactggctgacggaatttatgcctcttccgaccatcaagcattttatccgtactcctggtgatgcatggttactcaccactgcgatccccggaaaaacagcattccaggtattagaagaatatcctgattcaggtgaaaatattgttgatgcgctggcagtgttcctgcgccggttgcattcgattcctgtttgtaattgtccttttaacagcgatcgcgtatttcgtctcgctcaggcgcaatcacgaatgaataacggtttggttgatgcgagtgattttgatgacgagcgtaatggctggcctgttgaacaagtctggaaagaaatgcataaacttttgccattctcaccggattcagtcgtcactcatggtgatttctcacttgataaccttatttttgacgaggggaaattaataggttgtattgatgttggacgagtcggaatcgcagaccgataccaggatcttgccatcctatggaactgcctcggtgagttttctccttcattacagaaacggctttttcaaaaatatggtattgataatcctgatatgaataaattgcagtttcatttgatgctcgatgagtttttctaatcagaattggttaattggttgtaacattattcagattgggccccgttccactgagcgtcagaccccgtagaaaagatcaaaggatcttcttgagatcctttttttctgcgcgtaatctgctgcttgcaaacaaaaaaaccaccgctaccagcggtggtttgtttgccggatcaagagctaccaactctttttccgaaggtaactggcttcagcagagcgcagataccaaatactgttcttctagtgtagccgtagttaggccaccacttcaagaactctgtagcaccgcctacatacctcgctctgctaatcctgttaccagtggctgctgccagtggcgataagtcgtgtcttaccgggttggactcaagacgatagttaccggataaggcgcagcggtcgggctgaacggggggttcgtgcacacagcccagcttggagcgaacgacctacaccgaactgagatacctacagcgtgagctatgagaaagcgccacgcttcccgaagggagaaaggcggacaggtatccggtaagcggcagggtcggaacaggagagcgcacgagggagcttccagggggaaacgcctggtatctttatagtcctgtcgggtttcgccacctctgacttgagcgtcgatttttgtgatgctcgtcaggggggcggagcctatggaaaaacgccagcaacgcggcctttttacggttcctggccttttgctggccttttgctcacatgtt

pENTR-R4-H1-BsmBI-L2

tgcacacagcccagcttggagcgaacgacctacaccgaactgagatacctacagcgtgagctatgagaaagcgccacgcttcccgaagggagaaaggcggacaggtatccggtaagcggcagggtcggaacaggagagcgcacgagggagcttccagggggaaacgcctggtatctttatagtcctgtcgggtttcgccacctctgacttgagcgtcgatttttgtgatgctcgtcaggggggcggagcctatggaaaaacgccagcaacgcggcctttttacggttcctggccttttgctggccttttgctcacatgttctttcctgcgttatcccctgattctgtggataaccgtattaccgtatacacagccagtctgcaggtcgaccatagtgactggatatgttgtgttttacagtattatgtagtctgttttttatgcaaaatctaatttaatatattgatatttatatcattttacgtttctcgttcaacttttctatacaaagttgatagcttgatatcaatactctgaattcgaacgctgacgtcatcaacccgctccaaggaatcgcgggcccagtgtcactaggcgggaacacccagcgcgcgtgcgccctggcaggaagatggctgtgagggacaggggagtggcgccctgcaatatttgcatgtcgctatgtgttctgggaaatcaccataaacgtgaaatgtctttggatttgggaatcttataagttctgtatgaggaccacagatcaccgttcactgccgtataggcagtgagacgattaatgcgtctcagttttagagctagaaatagcaagttaaaataaggctagtccgttatcaacttgaaaaagtggcaccgagtcggtgcgttcactgccgtataggcagtttttttaagcttctagacccagctttcttgtacaaagttggcattataagaaagcattgcttatcaatttgttgcaacgaacaggtcactatcagtcaaaataaaatcattatttgccatccagctgcagctctggcccgtgtctcaaaatctctgatgttacattgcacaagataaaaatatatcatcatgaacaataaaactgtctgcttacataaacagtaatacaaggggtgttatgagccatattcaacgggaaacgtcgaggccgcgattaaattccaacatggatgctgatttatatgggtataaatgggctcgcgataatgtcgggcaatcaggtgcgacaatctatcgcttgtatgggaagcccgatgcgccagagttgtttctgaaacatggcaaaggtagcgttgccaatgatgttacagatgagatggtcagactaaactggctgacggaatttatgcctcttccgaccatcaagcattttatccgtactcctgatgatgcatggttactcaccactgcgatccccggaaaaacagcattccaggtattagaagaatatcctgattcaggtgaaaatattgttgatgcgctggcagtgttcctgcgccggttgcattcgattcctgtttgtaattgtccttttaacagcgatcgcgtatttcgtcttgctcaggcgcaatcacgaatgaataacggtttggttgatgcgagtgattttgatgacgagcgtaatggctggcctgttgaacaagtctggaaagaaatgcataaacttttgccattctcaccggattcagtcgtcactcatggtgatttctcacttgataaccttatttttgacgaggggaaattaataggttgtattgatgttggacgagtcggaatcgcagaccgataccaggatcttgccatcctatggaactgcctcggtgagttttctccttcattacagaaacggctttttcaaaaatatggtattgataatcctgatatgaataaattgcagtttcatttgatgctcgatgagtttttctaatcagaattggttaattggttgtaacactggcagagcattacgctgacttgacgggacggcgcaagctcatgaccaaaatcccttaacgtgagttttcgttccactgagcgtcagaccccgtagaaaagatcaaaggatcttcttgagatcctttttttctgcgcgtaatctgctgcttgcaaacaaaaaaaccaccgctaccagcggtggtttgtttgccggatcaagagctaccaactctttttccgaaggtaactggcttcagcagagcgcagataccaaatactgtccttctagtgtagccgtagttaggccaccacttcaagaactctgtagcaccgcctacatacctcgctctgctaatcctgttaccagtggctgctgccagtggcgataagtcgtgtcttaccgggttggactcaagacgatagttaccggataaggcgcagcggtcgggctgaacggggggttcg

pENTR-L5-CMV-R3

agcttcgttacataacttacggtaaatggcccgcctggctgaccgcccaacgacccccgcccattgacgtcaataatgacgtatgttcccatagtaacgccaatagggactttccattgacgtcaatgggtggagtatttacggtaaactgcccacttggcagtacatcaagtgtatcatatgccaagtacgccccctattgacgtcaatgacggtaaatggcccgcctggcattatgcccagtacatgaccttatgggactttcctacttggcagtacatctacgtattagtcatcgctattaccatggtgatgcggttttggcagtacatcaatgggcgtggatagcggtttgactcacggggatttccaagtctccaccccattgacgtcaatgggagtttgttttggcaccaaaatcaacgggactttccaaaatgtcgtaacaactccgccccattgacgcaaatgggcggtaggcgtgtacggtgggaggtctatataagcagagctcgtttagtgaaccgtcagatcgcctggagacgccatccacgctgttttgacctccatagaagacaccgggaccgatccagcctccgaagcttcaactttgtataataaagttgaacgagaaacgtaaaatgatataaatatcaatatattaaattagattttgcataaaaaacagactacataatactgtaaaacacaacatatccagtcactatgaatcaactacttagatggtattagtgacctgtactgcagctctggcccgtgtctcaaaatctctgatgttacattgcacaagataaaaatatatcatcatgaacaataaaactgtctgcttacataaacagtaatacaaggggtgttatgagccatattcaacgggaaacgtcgaggccgcgattaaattccaacatggatgctgatttatatgggtataaatgggctcgcgataatgtcgggcaatcaggtgcgacaatctatcgcttgtatgggaagcccgatgcgccagagttgtttctgaaacatggcaaaggtagcgttgccaatgatgttacagatgagatggtcagactaaactggctgacggaatttatgcctcttccgaccatcaagcattttatccgtactcctgatgatgcatggttactcaccactgcgatccccggaaaaacagcattccaggtattagaagaatatcctgattcaggtgaaaatattgttgatgcgctggcagtgttcctgcgccggttgcattcgattcctgtttgtaattgtccttttaacagcgatcgcgtatttcgtctcgctcaggcgcaatcacgaatgaataacggtttggttgatgcgagtgattttgatgacgagcgtaatggctggcctgttgaacaagtctggaaagaaatgcataaacttttgccattctcaccggattcagtcgtcactcatggtgatttctcacttgataaccttatttttgacgaggggaaattaataggttgtattgatgttggacgagtcggaatcgcagaccgataccaggatcttgccatcctatggaactgcctcggtgagttttctccttcattacagaaacggctttttcaaaaatatggtattgataatcctgatatgaataaattgcagtttcatttgatgctcgatgagtttttctaatcagaattggttaattggttgtaacactggcagagcattacgctgacttgacgggacggcgcaagctcatgaccaaaatcccttaacgtgagttttcgttccactgagcgtcagaccccgtagaaaagatcaaaggatcttcttgagatcctttttttctgcgcgtaatctgctgcttgcaaacaaaaaaaccaccgctaccagcggtggtttgtttgccggatcaagagctaccaactctttttccgaaggtaactggcttcagcagagcgcagataccaaatactgtccttctagtgtagccgtagttaggccaccacttcaagaactctgtagcaccgcctacatacctcgctctgctaatcctgttaccagtggctgctgccagtggcgataagtcgtgtcttaccgggttggactcaagacgatagttaccggataaggcgcagcggtcgggctgaacggggggttcgtgcacacagcccagcttggagcgaacgacctacaccgaactgagatacctacagcgtgagctatgagaaagcgccacgcttcccgaagggagaaaggcggacaggtatccggtaagcggcagggtcggaacaggagagcgcacgagggagcttccagggggaaacgcctggtatctttatagtcctgtcgggtttcgccacctctgacttgagcgtcgatttttgtgatgctcgtcaggggggcggagcctatggaaaaacgccagcaacgcggcctttttacggttcctggccttttgctggccttttgctcacatgttctttcctgcgttatcccctgattctgtggataaccgtattaccgctagccaggaagagtttgtagaaacgcaaaaaggccatccgtcaggatggccttctgcttagtttgatgcctggcagtttatggcgggcgtcctgcccgccaccctccgggccgttgcttcacaacgttcaaatccgctcccggcggatttgtcctactcaggagagcgttcaccgacaaacaacagataaaacgaaaggcccagtcttccgactgagcctttcgttttatttgatgcctggcagttccctactctcgcgttaacgctagcatggatctcgggccccaaataatgattttattttgactgatagtgacctgttcgttgcaacaaattgatgagcaatgcttttttataatgccaactttgtatacaaaagttga

pENTR-L5-CAG-R3

tcgacattgattattgactagttattaatagtaatcaattacggggtcattagttcatagcccatatatggagttccgcgttacataacttacggtaaatggcccgcctggctgaccgcccaacgacccccgcccattgacgtcaataatgacgtatgttcccatagtaacgccaatagggactttccattgacgtcaatgggtggagtatttacggtaaactgcccacttggcagtacatcaagtgtatcatatgccaagtacgccccctattgacgtcaatgacggtaaatggcccgcctggcattatgcccagtacatgaccttatgggactttcctacttggcagtacatctacgtattagtcatcgctattaccatggtcgaggtgagccccacgttctgcttcactctccccatctcccccccctccccacccccaattttgtatttatttattttttaattattttgtgcagcgatgggggcggggggggggggggggcgcgcgccaggcggggcggggcggggcgaggggcggggcggggcgaggcggagaggtgcggcggcagccaatcagagcggcgcgctccgaaagtttccttttatggcgaggcggcggcggcggcggccctataaaaagcgaagcgcgcggcgggcggggagtcgctgcgacgctgccttcgccccgtgccccgctccgccgccgcctcgcgccgcccgccccggctctgactgaccgcgttactcccacaggtgagcgggcgggacggcccttctcctccgggctgtaattagcgcttggtttaatgacggcttgtttcttttctgtggctgcgtgaaagccttgaggggctccgggagggccctttgtgcggggggagcggctcggggggtgcgtgcgtgtgtgtgtgcgtggggagcgccgcgtgcggctccgcgctgcccggcggctgtgagcgctgcgggcgcggcgcggggctttgtgcgctccgcagtgtgcgcgaggggagcgcggccgggggcggtgccccgcggtgcggggggggctgcgaggggaacaaaggctgcgtgcggggtgtgtgcgtgggggggtgagcagggggtgtgggcgcgtcggtcgggctgcaaccccccctgcacccccctccccgagttgctgagcacggcccggcttcgggtgcggggctccgtacggggcgtggcgcggggctcgccgtgccgggcggggggtggcggcaggtgggggtgccgggcggggcggggccgcctcgggccggggagggctcgggggaggggcgcggcggcccccggagcgccggcggctgtcgaggcgcggcgagccgcagccattgccttttatggtaatcgtgcgagagggcgcagggacttcctttgtcccaaatctgtgcggagccgaaatctgggaggcgccgccgcaccccctctagcgggcgcggggcgaagcggtgcggcgccggcaggaaggaaatgggcggggagggccttcgtgcgtcgccgcgccgccgtccccttctccctctccagcctcggggctgtccgcggggggacggctgccttcgggggggacggggcagggcggggttcggcttctggcgtgtgaccggcggctctagagcctctgctaaccatgttcatgccttcttctttttcctacagctcctgggcaacgtgctggttattgtgctgtctcatcattttggcaaagaattcaagcttcaactttgtataataaagttgaacgagaaacgtaaaatgatataaatatcaatatattaaattagattttgcataaaaaacagactacataatactgtaaaacacaacatatccagtcactatgaatcaactacttagatggtattagtgacctgtactgcagctctggcccgtgtctcaaaatctctgatgttacattgcacaagataaaaatatatcatcatgaacaataaaactgtctgcttacataaacagtaatacaaggggtgttatgagccatattcaacgggaaacgtcgaggccgcgattaaattccaacatggatgctgatttatatgggtataaatgggctcgcgataatgtcgggcaatcaggtgcgacaatctatcgcttgtatgggaagcccgatgcgccagagttgtttctgaaacatggcaaaggtagcgttgccaatgatgttacagatgagatggtcagactaaactggctgacggaatttatgcctcttccgaccatcaagcattttatccgtactcctgatgatgcatggttactcaccactgcgatccccggaaaaacagcattccaggtattagaagaatatcctgattcaggtgaaaatattgttgatgcgctggcagtgttcctgcgccggttgcattcgattcctgtttgtaattgtccttttaacagcgatcgcgtatttcgtctcgctcaggcgcaatcacgaatgaataacggtttggttgatgcgagtgattttgatgacgagcgtaatggctggcctgttgaacaagtctggaaagaaatgcataaacttttgccattctcaccggattcagtcgtcactcatggtgatttctcacttgataaccttatttttgacgaggggaaattaataggttgtattgatgttggacgagtcggaatcgcagaccgataccaggatcttgccatcctatggaactgcctcggtgagttttctccttcattacagaaacggctttttcaaaaatatggtattgataatcctgatatgaataaattgcagtttcatttgatgctcgatgagtttttctaatcagaattggttaattggttgtaacactggcagagcattacgctgacttgacgggacggcgcaagctcatgaccaaaatcccttaacgtgagttttcgttccactgagcgtcagaccccgtagaaaagatcaaaggatcttcttgagatcctttttttctgcgcgtaatctgctgcttgcaaacaaaaaaaccaccgctaccagcggtggtttgtttgccggatcaagagctaccaactctttttccgaaggtaactggcttcagcagagcgcagataccaaatactgtccttctagtgtagccgtagttaggccaccacttcaagaactctgtagcaccgcctacatacctcgctctgctaatcctgttaccagtggctgctgccagtggcgataagtcgtgtcttaccgggttggactcaagacgatagttaccggataaggcgcagcggtcgggctgaacggggggttcgtgcacacagcccagcttggagcgaacgacctacaccgaactgagatacctacagcgtgagctatgagaaagcgccacgcttcccgaagggagaaaggcggacaggtatccggtaagcggcagggtcggaacaggagagcgcacgagggagcttccagggggaaacgcctggtatctttatagtcctgtcgggtttcgccacctctgacttgagcgtcgatttttgtgatgctcgtcaggggggcggagcctatggaaaaacgccagcaacgcggcctttttacggttcctggccttttgctggccttttgctcacatgttctttcctgcgttatcccctgattctgtggataaccgtattaccgctagccaggaagagtttgtagaaacgcaaaaaggccatccgtcaggatggccttctgcttagtttgatgcctggcagtttatggcgggcgtcctgcccgccaccctccgggccgttgcttcacaacgttcaaatccgctcccggcggatttgtcctactcaggagagcgttcaccgacaaacaacagataaaacgaaaggcccagtcttccgactgagcctttcgttttatttgatgcctggcagttccctactctcgcgttaacgctagcatggatctcgggccccaaataatgattttattttgactgatagtgacctgttcgttgcaacaaattgatgagcaatgcttttttataatgccaactttgtatacaaaagttgaagcttg

pENTR-L5-EF1α-R3

agctttgcaaagatggataaagttttaaacagagaggaatctttgcagctaatggaccttctaggtcttgaaaggagtgggaattggctccggtgcccgtcagtgggcagagcgcacatcgcccacagtccccgagaagttggggggaggggtcggcaattgaaccggtgcctagagaaggtggcgcggggtaaactgggaaagtgatgtcgtgtactggctccgcctttttcccgagggtgggggagaaccgtatataagtgcagtagtcgccgtgaacgttctttttcgcaacgggtttgccgccagaacacaggtaagtgccgtgtgtggttcccgcgggcctggcctctttacgggttatggcccttgcgtgccttgaattacttccacctggctgcagtacgtgattcttgatcccgagcttcgggttggaagtgggtgggagagttcgaggccttgcgcttaaggagccccttcgcctcgtgcttgagttgaggcctggcctgggcgctggggccgccgcgtgcgaatctggtggcaccttcgcgcctgtctcgctgctttcgataagtctctagccatttaaaatttttgatgacctgctgcgacgctttttttctggcaagatagtcttgtaaatgcgggccaagatctgcacactggtatttcggtttttggggccgcgggcggcgacggggcccgtgcgtcccagcgcacatgttcggcgaggcggggcctgcgagcgcggccaccgagaatcggacgggggtagtctcaagctggccggcctgctctggtgcctggcctcgcgccgccgtgtatcgccccgccctgggcggcaaggctggcccggtcggcaccagttgcgtgagcggaaagatggccgcttcccggccctgctgcagggagctcaaaatggaggacgcggcgctcgggagagcgggcgggtgagtcacccacacaaaggaaaagggcctttccgtcctcagccgtcgcttcatgtgactccacggagtaccgggcgccgtccaggcacctcgattagttctcgagcttttggagtacgtcgtctttaggttggggggaggggttttatgcgatggagtttccccacactgagtgggtggagactgaagttaggccagcttggcacttgatgtaattctccttggaatttgccctttttgagtttggatcttggttcattctcaagcctcagacagtggttcaaagtttttttcttccatttcaggtgtcgtgaggaattagcttggtactaatacgactcactatagggagacccaagctggctaggtaagcttcaactttgtataataaagttgaacgagaaacgtaaaatgatataaatatcaatatattaaattagattttgcataaaaaacagactacataatactgtaaaacacaacatatccagtcactatgaatcaactacttagatggtattagtgacctgtactgcagctctggcccgtgtctcaaaatctctgatgttacattgcacaagataaaaatatatcatcatgaacaataaaactgtctgcttacataaacagtaatacaaggggtgttatgagccatattcaacgggaaacgtcgaggccgcgattaaattccaacatggatgctgatttatatgggtataaatgggctcgcgataatgtcgggcaatcaggtgcgacaatctatcgcttgtatgggaagcccgatgcgccagagttgtttctgaaacatggcaaaggtagcgttgccaatgatgttacagatgagatggtcagactaaactggctgacggaatttatgcctcttccgaccatcaagcattttatccgtactcctgatgatgcatggttactcaccactgcgatccccggaaaaacagcattccaggtattagaagaatatcctgattcaggtgaaaatattgttgatgcgctggcagtgttcctgcgccggttgcattcgattcctgtttgtaattgtccttttaacagcgatcgcgtatttcgtctcgctcaggcgcaatcacgaatgaataacggtttggttgatgcgagtgattttgatgacgagcgtaatggctggcctgttgaacaagtctggaaagaaatgcataaacttttgccattctcaccggattcagtcgtcactcatggtgatttctcacttgataaccttatttttgacgaggggaaattaataggttgtattgatgttggacgagtcggaatcgcagaccgataccaggatcttgccatcctatggaactgcctcggtgagttttctccttcattacagaaacggctttttcaaaaatatggtattgataatcctgatatgaataaattgcagtttcatttgatgctcgatgagtttttctaatcagaattggttaattggttgtaacactggcagagcattacgctgacttgacgggacggcgcaagctcatgaccaaaatcccttaacgtgagttttcgttccactgagcgtcagaccccgtagaaaagatcaaaggatcttcttgagatcctttttttctgcgcgtaatctgctgcttgcaaacaaaaaaaccaccgctaccagcggtggtttgtttgccggatcaagagctaccaactctttttccgaaggtaactggcttcagcagagcgcagataccaaatactgtccttctagtgtagccgtagttaggccaccacttcaagaactctgtagcaccgcctacatacctcgctctgctaatcctgttaccagtggctgctgccagtggcgataagtcgtgtcttaccgggttggactcaagacgatagttaccggataaggcgcagcggtcgggctgaacggggggttcgtgcacacagcccagcttggagcgaacgacctacaccgaactgagatacctacagcgtgagctatgagaaagcgccacgcttcccgaagggagaaaggcggacaggtatccggtaagcggcagggtcggaacaggagagcgcacgagggagcttccagggggaaacgcctggtatctttatagtcctgtcgggtttcgccacctctgacttgagcgtcgatttttgtgatgctcgtcaggggggcggagcctatggaaaaacgccagcaacgcggcctttttacggttcctggccttttgctggccttttgctcacatgttctttcctgcgttatcccctgattctgtggataaccgtattaccgctagccaggaagagtttgtagaaacgcaaaaaggccatccgtcaggatggccttctgcttagtttgatgcctggcagtttatggcgggcgtcctgcccgccaccctccgggccgttgcttcacaacgttcaaatccgctcccggcggatttgtcctactcaggagagcgttcaccgacaaacaacagataaaacgaaaggcccagtcttccgactgagcctttcgttttatttgatgcctggcagttccctactctcgcgttaacgctagcatggatctcgggccccaaataatgattttattttgactgatagtgacctgttcgttgcaacaaattgatgagcaatgcttttttataatgccaactttgtatacaaaagttga

pENTR-L3-Cas9-L2

gtacaaagttggcattataagaaagcattgcttatcaatttgttgcaacgaacaggtcactatcagtcaaaataaaatcattatttgccatccagctgcagctctggcccgtgtctcaaaatctctgatgttacattgcacaagataaaaatatatcatcatgaacaataaaactgtctgcttacataaacagtaatacaaggggtgttatgagccatattcaacgggaaacgtcgaggccgcgattaaattccaacatggatgctgatttatatgggtataaatgggctcgcgataatgtcgggcaatcaggtgcgacaatctatcgcttgtatgggaagcccgatgcgccagagttgtttctgaaacatggcaaaggtagcgttgccaatgatgttacagatgagatggtcagactaaactggctgacggaatttatgcctcttccgaccatcaagcattttatccgtactcctgatgatgcatggttactcaccactgcgatccccggaaaaacagcattccaggtattagaagaatatcctgattcaggtgaaaatattgttgatgcgctggcagtgttcctgcgccggttgcattcgattcctgtttgtaattgtccttttaacagcgatcgcgtatttcgtctcgctcaggcgcaatcacgaatgaataacggtttggttgatgcgagtgattttgatgacgagcgtaatggctggcctgttgaacaagtctggaaagaaatgcataaacttttgccattctcaccggattcagtcgtcactcatggtgatttctcacttgataaccttatttttgacgaggggaaattaataggttgtattgatgttggacgagtcggaatcgcagaccgataccaggatcttgccatcctatggaactgcctcggtgagttttctccttcattacagaaacggctttttcaaaaatatggtattgataatcctgatatgaataaattgcagtttcatttgatgctcgatgagtttttctaatcagaattggttaattggttgtaacactggcagagcattacgctgacttgacgggacggcgcaagctcatgaccaaaatcccttaacgtgagttttcgttccactgagcgtcagaccccgtagaaaagatcaaaggatcttcttgagatcctttttttctgcgcgtaatctgctgcttgcaaacaaaaaaaccaccgctaccagcggtggtttgtttgccggatcaagagctaccaactctttttccgaaggtaactggcttcagcagagcgcagataccaaatactgtccttctagtgtagccgtagttaggccaccacttcaagaactctgtagcaccgcctacatacctcgctctgctaatcctgttaccagtggctgctgccagtggcgataagtcgtgtcttaccgggttggactcaagacgatagttaccggataaggcgcagcggtcgggctgaacggggggttcgtgcacacagcccagcttggagcgaacgacctacaccgaactgagatacctacagcgtgagctatgagaaagcgccacgcttcccgaagggagaaaggcggacaggtatccggtaagcggcagggtcggaacaggagagcgcacgagggagcttccagggggaaacgcctggtatctttatagtcctgtcgggtttcgccacctctgacttgagcgtcgatttttgtgatgctcgtcaggggggcggagcctatggaaaaacgccagcaacgcggcctttttacggttcctggccttttgctggccttttgctcacatgttctttcctgcgttatcccctgattctgtggataaccgtattaccgctagccaggaagagtttgtagaaacgcaaaaaggccatccgtcaggatggccttctgcttagtttgatgcctggcagtttatggcgggcgtcctgcccgccaccctccgggccgttgcttcacaacgttcaaatccgctcccggcggatttgtcctactcaggagagcgttcaccgacaaacaacagataaaacgaaaggcccagtcttccgactgagcctttcgttttatttgatgcctggcagttccctactctcgcgttaacgctagcatggatctcgggccccaaataatgattttattttgactgatagtgacctgttcgttgcaacaaattgatgagcaatgcttttttataatgccaactttgtataataaagttggcttcgaaggagatagaaccatggactataaggaccacgacggagactacaaggatcatgatattgattacaaagacgatgacgataagatggccccaaagaagaagcggaaggtcggtatccacggagtcccagcagccgacaagaagtacagcatcggcctggacatcggcaccaactctgtgggctgggccgtgatcaccgacgagtacaaggtgcccagcaagaaattcaaggtgctgggcaacaccgaccggcacagcatcaagaagaacctgatcggagccctgctgttcgacagcggcgaaacagccgaggccacccggctgaagagaaccgccagaagaagatacaccagacggaagaaccggatctgctatctgcaagagatcttcagcaacgagatggccaaggtggacgacagcttcttccacagactggaagagtccttcctggtggaagaggataagaagcacgagcggcaccccatcttcggcaacatcgtggacgaggtggcctaccacgagaagtaccccaccatctaccacctgagaaagaaactggtggacagcaccgacaaggccgacctgcggctgatctatctggccctggcccacatgatcaagttccggggccacttcctgatcgagggcgacctgaaccccgacaacagcgacgtggacaagctgttcatccagctggtgcagacctacaaccagctgttcgaggaaaaccccatcaacgccagcggcgtggacgccaaggccatcctgtctgccagactgagcaagagcagacggctggaaaatctgatcgcccagctgcccggcgagaagaagaatggcctgttcggaaacctgattgccctgagcctgggcctgacccccaacttcaagagcaacttcgacctggccgaggatgccaaactgcagctgagcaaggacacctacgacgacgacctggacaacctgctggcccagatcggcgaccagtacgccgacctgtttctggccgccaagaacctgtccgacgccatcctgctgagcgacatcctgagagtgaacaccgagatcaccaaggcccccctgagcgcctctatgatcaagagatacgacgagcaccaccaggacctgaccctgctgaaagctctcgtgcggcagcagctgcctgagaagtacaaagagattttcttcgaccagagcaagaacggctacgccggctacattgacggcggagccagccaggaagagttctacaagttcatcaagcccatcctggaaaagatggacggcaccgaggaactgctcgtgaagctgaacagagaggacctgctgcggaagcagcggaccttcgacaacggcagcatcccccaccagatccacctgggagagctgcacgccattctgcggcggcaggaagatttttacccattcctgaaggacaaccgggaaaagatcgagaagatcctgaccttccgcatcccctactacgtgggccctctggccaggggaaacagcagattcgcctggatgaccagaaagagcgaggaaaccatcaccccctggaacttcgaggaagtggtggacaagggcgcttccgcccagagcttcatcgagcggatgaccaacttcgataagaacctgcccaacgagaaggtgctgcccaagcacagcctgctgtacgagtacttcaccgtgtataacgagctgaccaaagtgaaatacgtgaccgagggaatgagaaagcccgccttcctgagcggcgagcagaaaaaggccatcgtggacctgctgttcaagaccaaccggaaagtgaccgtgaagcagctgaaagaggactacttcaagaaaatcgagtgcttcgactccgtggaaatctccggcgtggaagatcggttcaacgcctccctgggcacataccacgatctgctgaaaattatcaaggacaaggacttcctggacaatgaggaaaacgaggacattctggaagatatcgtgctgaccctgacactgtttgaggacagagagatgatcgaggaacggctgaaaacctatgcccacctgttcgacgacaaagtgatgaagcagctgaagcggcggagatacaccggctggggcaggctgagccggaagctgatcaacggcatccgggacaagcagtccggcaagacaatcctggatttcctgaagtccgacggcttcgccaacagaaacttcatgcagctgatccacgacgacagcctgacctttaaagaggacatccagaaagcccaggtgtccggccagggcgatagcctgcacgagcacattgccaatctggccggcagccccgccattaagaagggcatcctgcagacagtgaaggtggtggacgagctcgtgaaagtgatgggccggcacaagcccgagaacatcgtgatcgaaatggccagagagaaccagaccacccagaagggacagaagaacagccgcgagagaatgaagcggatcgaagagggcatcaaagagctgggcagccagatcctgaaagaacaccccgtggaaaacacccagctgcagaacgagaagctgtacctgtactacctgcagaatgggcgggatatgtacgtggaccaggaactggacatcaaccggctgtccgactacgatgtggaccatatcgtgcctcagagctttctgaaggacgactccatcgacaacaaggtgctgaccagaagcgacaagaaccggggcaagagcgacaacgtgccctccgaagaggtcgtgaagaagatgaagaactactggcggcagctgctgaacgccaagctgattacccagagaaagttcgacaatctgaccaaggccgagagaggcggcctgagcgaactggataaggccggcttcatcaagagacagctggtggaaacccggcagatcacaaagcacgtggcacagatcctggactcccggatgaacactaagtacgacgagaatgacaagctgatccgggaagtgaaagtgatcaccctgaagtccaagctggtgtccgatttccggaaggatttccagttttacaaagtgcgcgagatcaacaactaccaccacgcccacgacgcctacctgaacgccgtcgtgggaaccgccctgatcaaaaagtaccctaagctggaaagcgagttcgtgtacggcgactacaaggtgtacgacgtgcggaagatgatcgccaagagcgagcaggaaatcggcaaggctaccgccaagtacttcttctacagcaacatcatgaactttttcaagaccgagattaccctggccaacggcgagatccggaagcggcctctgatcgagacaaacggcgaaaccggggagatcgtgtgggataagggccgggattttgccaccgtgcggaaagtgctgagcatgccccaagtgaatatcgtgaaaaagaccgaggtgcagacaggcggcttcagcaaagagtctatcctgcccaagaggaacagcgataagctgatcgccagaaagaaggactgggaccctaagaagtacggcggcttcgacagccccaccgtggcctattctgtgctggtggtggccaaagtggaaaagggcaagtccaagaaactgaagagtgtgaaagagctgctggggatcaccatcatggaaagaagcagcttcgagaagaatcccatcgactttctggaagccaagggctacaaagaagtgaaaaaggacctgatcatcaagctgcctaagtactccctgttcgagctggaaaacggccggaagagaatgctggcctctgccggcgaactgcagaagggaaacgaactggccctgccctccaaatatgtgaacttcctgtacctggccagccactatgagaagctgaagggctcccccgaggataatgagcagaaacagctgtttgtggaacagcacaagcactacctggacgagatcatcgagcagatcagcgagttctccaagagagtgatcctggccgacgctaatctggacaaagtgctgtccgcctacaacaagcaccgggataagcccatcagagagcaggccgagaatatcatccacctgtttaccctgaccaatctgggagcccctgccgccttcaagtactttgacaccaccatcgaccggaagaggtacaccagcaccaaagaggtgctggacgccaccctgatccaccagagcatcaccggcctgtacgagacacggatcgacctgtctcagctgggaggcgacaaaaggccggcggccacgaaaaaggccggccaggcaaaaaagaaaaaggaattctaatgacagctttctt

pENTR-L3-Cas9n-L2

gtacaaagttggcattataagaaagcattgcttatcaatttgttgcaacgaacaggtcactatcagtcaaaataaaatcattatttgccatccagctgcagctctggcccgtgtctcaaaatctctgatgttacattgcacaagataaaaatatatcatcatgaacaataaaactgtctgcttacataaacagtaatacaaggggtgttatgagccatattcaacgggaaacgtcgaggccgcgattaaattccaacatggatgctgatttatatgggtataaatgggctcgcgataatgtcgggcaatcaggtgcgacaatctatcgcttgtatgggaagcccgatgcgccagagttgtttctgaaacatggcaaaggtagcgttgccaatgatgttacagatgagatggtcagactaaactggctgacggaatttatgcctcttccgaccatcaagcattttatccgtactcctgatgatgcatggttactcaccactgcgatccccggaaaaacagcattccaggtattagaagaatatcctgattcaggtgaaaatattgttgatgcgctggcagtgttcctgcgccggttgcattcgattcctgtttgtaattgtccttttaacagcgatcgcgtatttcgtctcgctcaggcgcaatcacgaatgaataacggtttggttgatgcgagtgattttgatgacgagcgtaatggctggcctgttgaacaagtctggaaagaaatgcataaacttttgccattctcaccggattcagtcgtcactcatggtgatttctcacttgataaccttatttttgacgaggggaaattaataggttgtattgatgttggacgagtcggaatcgcagaccgataccaggatcttgccatcctatggaactgcctcggtgagttttctccttcattacagaaacggctttttcaaaaatatggtattgataatcctgatatgaataaattgcagtttcatttgatgctcgatgagtttttctaatcagaattggttaattggttgtaacactggcagagcattacgctgacttgacgggacggcgcaagctcatgaccaaaatcccttaacgtgagttttcgttccactgagcgtcagaccccgtagaaaagatcaaaggatcttcttgagatcctttttttctgcgcgtaatctgctgcttgcaaacaaaaaaaccaccgctaccagcggtggtttgtttgccggatcaagagctaccaactctttttccgaaggtaactggcttcagcagagcgcagataccaaatactgtccttctagtgtagccgtagttaggccaccacttcaagaactctgtagcaccgcctacatacctcgctctgctaatcctgttaccagtggctgctgccagtggcgataagtcgtgtcttaccgggttggactcaagacgatagttaccggataaggcgcagcggtcgggctgaacggggggttcgtgcacacagcccagcttggagcgaacgacctacaccgaactgagatacctacagcgtgagctatgagaaagcgccacgcttcccgaagggagaaaggcggacaggtatccggtaagcggcagggtcggaacaggagagcgcacgagggagcttccagggggaaacgcctggtatctttatagtcctgtcgggtttcgccacctctgacttgagcgtcgatttttgtgatgctcgtcaggggggcggagcctatggaaaaacgccagcaacgcggcctttttacggttcctggccttttgctggccttttgctcacatgttctttcctgcgttatcccctgattctgtggataaccgtattaccgctagccaggaagagtttgtagaaacgcaaaaaggccatccgtcaggatggccttctgcttagtttgatgcctggcagtttatggcgggcgtcctgcccgccaccctccgggccgttgcttcacaacgttcaaatccgctcccggcggatttgtcctactcaggagagcgttcaccgacaaacaacagataaaacgaaaggcccagtcttccgactgagcctttcgttttatttgatgcctggcagttccctactctcgcgttaacgctagcatggatctcgggccccaaataatgattttattttgactgatagtgacctgttcgttgcaacaaattgatgagcaatgcttttttataatgccaactttgtataataaagttggcttcgaaggagatagaaccatggactataaggaccacgacggagactacaaggatcatgatattgattacaaagacgatgacgataagatggccccaaagaagaagcggaaggtcggtatccacggagtcccagcagccgacaagaagtacagcatcggcctggCcatcggcaccaactctgtgggctgggccgtgatcaccgacgagtacaaggtgcccagcaagaaattcaaggtgctgggcaacaccgaccggcacagcatcaagaagaacctgatcggagccctgctgttcgacagcggcgaaacagccgaggccacccggctgaagagaaccgccagaagaagatacaccagacggaagaaccggatctgctatctgcaagagatcttcagcaacgagatggccaaggtggacgacagcttcttccacagactggaagagtccttcctggtggaagaggataagaagcacgagcggcaccccatcttcggcaacatcgtggacgaggtggcctaccacgagaagtaccccaccatctaccacctgagaaagaaactggtggacagcaccgacaaggccgacctgcggctgatctatctggccctggcccacatgatcaagttccggggccacttcctgatcgagggcgacctgaaccccgacaacagcgacgtggacaagctgttcatccagctggtgcagacctacaaccagctgttcgaggaaaaccccatcaacgccagcggcgtggacgccaaggccatcctgtctgccagactgagcaagagcagacggctggaaaatctgatcgcccagctgcccggcgagaagaagaatggcctgttcggaaacctgattgccctgagcctgggcctgacccccaacttcaagagcaacttcgacctggccgaggatgccaaactgcagctgagcaaggacacctacgacgacgacctggacaacctgctggcccagatcggcgaccagtacgccgacctgtttctggccgccaagaacctgtccgacgccatcctgctgagcgacatcctgagagtgaacaccgagatcaccaaggcccccctgagcgcctctatgatcaagagatacgacgagcaccaccaggacctgaccctgctgaaagctctcgtgcggcagcagctgcctgagaagtacaaagagattttcttcgaccagagcaagaacggctacgccggctacattgacggcggagccagccaggaagagttctacaagttcatcaagcccatcctggaaaagatggacggcaccgaggaactgctcgtgaagctgaacagagaggacctgctgcggaagcagcggaccttcgacaacggcagcatcccccaccagatccacctgggagagctgcacgccattctgcggcggcaggaagatttttacccattcctgaaggacaaccgggaaaagatcgagaagatcctgaccttccgcatcccctactacgtgggccctctggccaggggaaacagcagattcgcctggatgaccagaaagagcgaggaaaccatcaccccctggaacttcgaggaagtggtggacaagggcgcttccgcccagagcttcatcgagcggatgaccaacttcgataagaacctgcccaacgagaaggtgctgcccaagcacagcctgctgtacgagtacttcaccgtgtataacgagctgaccaaagtgaaatacgtgaccgagggaatgagaaagcccgccttcctgagcggcgagcagaaaaaggccatcgtggacctgctgttcaagaccaaccggaaagtgaccgtgaagcagctgaaagaggactacttcaagaaaatcgagtgcttcgactccgtggaaatctccggcgtggaagatcggttcaacgcctccctgggcacataccacgatctgctgaaaattatcaaggacaaggacttcctggacaatgaggaaaacgaggacattctggaagatatcgtgctgaccctgacactgtttgaggacagagagatgatcgaggaacggctgaaaacctatgcccacctgttcgacgacaaagtgatgaagcagctgaagcggcggagatacaccggctggggcaggctgagccggaagctgatcaacggcatccgggacaagcagtccggcaagacaatcctggatttcctgaagtccgacggcttcgccaacagaaacttcatgcagctgatccacgacgacagcctgacctttaaagaggacatccagaaagcccaggtgtccggccagggcgatagcctgcacgagcacattgccaatctggccggcagccccgccattaagaagggcatcctgcagacagtgaaggtggtggacgagctcgtgaaagtgatgggccggcacaagcccgagaacatcgtgatcgaaatggccagagagaaccagaccacccagaagggacagaagaacagccgcgagagaatgaagcggatcgaagagggcatcaaagagctgggcagccagatcctgaaagaacaccccgtggaaaacacccagctgcagaacgagaagctgtacctgtactacctgcagaatgggcgggatatgtacgtggaccaggaactggacatcaaccggctgtccgactacgatgtggaccatatcgtgcctcagagctttctgaaggacgactccatcgacaacaaggtgctgaccagaagcgacaagaaccggggcaagagcgacaacgtgccctccgaagaggtcgtgaagaagatgaagaactactggcggcagctgctgaacgccaagctgattacccagagaaagttcgacaatctgaccaaggccgagagaggcggcctgagcgaactggataaggccggcttcatcaagagacagctggtggaaacccggcagatcacaaagcacgtggcacagatcctggactcccggatgaacactaagtacgacgagaatgacaagctgatccgggaagtgaaagtgatcaccctgaagtccaagctggtgtccgatttccggaaggatttccagttttacaaagtgcgcgagatcaacaactaccaccacgcccacgacgcctacctgaacgccgtcgtgggaaccgccctgatcaaaaagtaccctaagctggaaagcgagttcgtgtacggcgactacaaggtgtacgacgtgcggaagatgatcgccaagagcgagcaggaaatcggcaaggctaccgccaagtacttcttctacagcaacatcatgaactttttcaagaccgagattaccctggccaacggcgagatccggaagcggcctctgatcgagacaaacggcgaaaccggggagatcgtgtgggataagggccgggattttgccaccgtgcggaaagtgctgagcatgccccaagtgaatatcgtgaaaaagaccgaggtgcagacaggcggcttcagcaaagagtctatcctgcccaagaggaacagcgataagctgatcgccagaaagaaggactgggaccctaagaagtacggcggcttcgacagccccaccgtggcctattctgtgctggtggtggccaaagtggaaaagggcaagtccaagaaactgaagagtgtgaaagagctgctggggatcaccatcatggaaagaagcagcttcgagaagaatcccatcgactttctggaagccaagggctacaaagaagtgaaaaaggacctgatcatcaagctgcctaagtactccctgttcgagctggaaaacggccggaagagaatgctggcctctgccggcgaactgcagaagggaaacgaactggccctgccctccaaatatgtgaacttcctgtacctggccagccactatgagaagctgaagggctcccccgaggataatgagcagaaacagctgtttgtggaacagcacaagcactacctggacgagatcatcgagcagatcagcgagttctccaagagagtgatcctggccgacgctaatctggacaaagtgctgtccgcctacaacaagcaccgggataagcccatcagagagcaggccgagaatatcatccacctgtttaccctgaccaatctgggagcccctgccgccttcaagtactttgacaccaccatcgaccggaagaggtacaccagcaccaaagaggtgctggacgccaccctgatccaccagagcatcaccggcctgtacgagacacggatcgacctgtctcagctgggaggcgacaaaaggccggcggccacgaaaaaggccggccaggcaaaaaagaaaaaggaattctaatgacagctttctt

pENTR-L3-Csy4-Cas9-L2

ttgtacaaagttggcattataagaaagcattgcttatcaatttgttgcaacgaacaggtcactatcagtcaaaataaaatcattatttgccatccagctgcagctctggcccgtgtctcaaaatctctgatgttacattgcacaagataaaaatatatcatcatgaacaataaaactgtctgcttacataaacagtaatacaaggggtgttatgagccatattcaacgggaaacgtcgaggccgcgattaaattccaacatggatgctgatttatatgggtataaatgggctcgcgataatgtcgggcaatcaggtgcgacaatctatcgcttgtatgggaagcccgatgcgccagagttgtttctgaaacatggcaaaggtagcgttgccaatgatgttacagatgagatggtcagactaaactggctgacggaatttatgcctcttccgaccatcaagcattttatccgtactcctgatgatgcatggttactcaccactgcgatccccggaaaaacagcattccaggtattagaagaatatcctgattcaggtgaaaatattgttgatgcgctggcagtgttcctgcgccggttgcattcgattcctgtttgtaattgtccttttaacagcgatcgcgtatttcgtctcgctcaggcgcaatcacgaatgaataacggtttggttgatgcgagtgattttgatgacgagcgtaatggctggcctgttgaacaagtctggaaagaaatgcataaacttttgccattctcaccggattcagtcgtcactcatggtgatttctcacttgataaccttatttttgacgaggggaaattaataggttgtattgatgttggacgagtcggaatcgcagaccgataccaggatcttgccatcctatggaactgcctcggtgagttttctccttcattacagaaacggctttttcaaaaatatggtattgataatcctgatatgaataaattgcagtttcatttgatgctcgatgagtttttctaatcagaattggttaattggttgtaacactggcagagcattacgctgacttgacgggacggcgcaagctcatgaccaaaatcccttaacgtgagttttcgttccactgagcgtcagaccccgtagaaaagatcaaaggatcttcttgagatcctttttttctgcgcgtaatctgctgcttgcaaacaaaaaaaccaccgctaccagcggtggtttgtttgccggatcaagagctaccaactctttttccgaaggtaactggcttcagcagagcgcagataccaaatactgtccttctagtgtagccgtagttaggccaccacttcaagaactctgtagcaccgcctacatacctcgctctgctaatcctgttaccagtggctgctgccagtggcgataagtcgtgtcttaccgggttggactcaagacgatagttaccggataaggcgcagcggtcgggctgaacggggggttcgtgcacacagcccagcttggagcgaacgacctacaccgaactgagatacctacagcgtgagctatgagaaagcgccacgcttcccgaagggagaaaggcggacaggtatccggtaagcggcagggtcggaacaggagagcgcacgagggagcttccagggggaaacgcctggtatctttatagtcctgtcgggtttcgccacctctgacttgagcgtcgatttttgtgatgctcgtcaggggggcggagcctatggaaaaacgccagcaacgcggcctttttacggttcctggccttttgctggccttttgctcacatgttctttcctgcgttatcccctgattctgtggataaccgtattaccgctagccaggaagagtttgtagaaacgcaaaaaggccatccgtcaggatggccttctgcttagtttgatgcctggcagtttatggcgggcgtcctgcccgccaccctccgggccgttgcttcacaacgttcaaatccgctcccggcggatttgtcctactcaggagagcgttcaccgacaaacaacagataaaacgaaaggcccagtcttccgactgagcctttcgttttatttgatgcctggcagttccctactctcgcgttaacgctagcatggatctcgggccccaaataatgattttattttgactgatagtgacctgttcgttgcaacaaattgatgagcaatgcttttttataatgccaactttgtataataaagttgggtaccgcgggcccgggatccaccggtcgccaccatggaccactacctcgacattcgcttgcgaccggacccggaatttcccccggcgcaactcatgagcgtgctcttcggcaagctccaccaggccctggtggcacagggcggggacaggatcggcgtgagcttccccgacctcgacgaaagccgctcccggctgggcgagcgcctgcgcattcatgcctcggcggacgaccttcgtgccctgctcgcccggccctggctggaagggttgcgggaccatctgcaattcggagaaccggcagtcgtgcctcaccccacaccgtaccgtcaggtcagtcgggttcaggcgaaaagcaatccggaacgcctgcggcggcggctcatgcgccggcacgatctgagtgaggaggaggctcggaaacgcattcccgatacggtcgcgagagccttggacctgcccttcgtcacgctacgcagccagagcaccggacagcacttccgtctcttcatccgccacgggccgttgcaggtgacggcagaggaaggaggattcacctgttacgggttgagcaaaggaggtttcgttccctggttcgagggcagaggaagtctgctaacatgcggtgacgtcgaggagaatcctggcccaatggataaaaagtattctattggtttagacatcggcactaattccgttggatgggctgtcataaccgatgaatacaaagtaccttcaaagaaatttaaggtgttggggaacacagaccgtcattcgattaaaaagaatcttatcggtgccctcctattcgatagtggcgaaacggcagaggcgactcgcctgaaacgaaccgctcggagaaggtatacacgtcgcaagaaccgaatatgttacttacaagaaatttttagcaatgagatggccaaagttgacgattctttctttcaccgtttggaagagtccttccttgtcgaagaggacaagaaacatgaacggcaccccatctttggaaacatagtagatgaggtggcatatcatgaaaagtacccaacgatttatcacctcagaaaaaagctagttgactcaactgataaagcggacctgaggttaatctacttggctcttgcccatatgataaagttccgtgggcactttctcattgagggtgatctaaatccggacaactcggatgtcgacaaactgttcatccagttagtacaaacctataatcagttgtttgaagagaaccctataaatgcaagtggcgtggatgcgaaggctattcttagcgcccgcctctctaaatcccgacggctagaaaacctgatcgcacaattacccggagagaagaaaaatgggttgttcggtaaccttatagcgctctcactaggcctgacaccaaattttaagtcgaacttcgacttagctgaagatgccaaattgcagcttagtaaggacacgtacgatgacgatctcgacaatctactggcacaaattggagatcagtatgcggacttatttttggctgccaaaaaccttagcgatgcaatcctcctatctgacatactgagagttaatactgagattaccaaggcgccgttatccgcttcaatgatcaaaaggtacgatgaacatcaccaagacttgacacttctcaaggccctagtccgtcagcaactgcctgagaaatataaggaaatattctttgatcagtcgaaaaacgggtacgcaggttatattgacggcggagcgagtcaagaggaattctacaagtttatcaaacccatattagagaagatggatgggacggaagagttgcttgtaaaactcaatcgcgaagatctactgcgaaagcagcggactttcgacaacggtagcattccacatcaaatccacttaggcgaattgcatgctatacttagaaggcaggaggatttttatccgttcctcaaagacaatcgtgaaaagattgagaaaatcctaacctttcgcataccttactatgtgggacccctggcccgagggaactctcggttcgcatggatgacaagaaagtccgaagaaacgattactccatggaattttgaggaagttgtcgataaaggtgcgtcagctcaatcgttcatcgagaggatgaccaactttgacaagaatttaccgaacgaaaaagtattgcctaagcacagtttactttacgagtatttcacagtgtacaatgaactcacgaaagttaagtatgtcactgagggcatgcgtaaacccgcctttctaagcggagaacagaagaaagcaatagtagatctgttattcaagaccaaccgcaaagtgacagttaagcaattgaaagaggactactttaagaaaattgaatgcttcgattctgtcgagatctccggggtagaagatcgatttaatgcgtcacttggtacgtatcatgacctcctaaagataattaaagataaggacttcctggataacgaagagaatgaagatatcttagaagatatagtgttgactcttaccctctttgaagatcgggaaatgattgaggaaagactaaaaacatacgctcacctgttcgacgataaggttatgaaacagttaaagaggcgtcgctatacgggctggggacgattgtcgcggaaacttatcaacgggataagagacaagcaaagtggtaaaactattctcgattttctaaagagcgacggcttcgccaataggaactttatgcagctgatccatgatgactctttaaccttcaaagaggatatacaaaaggcacaggtttccggacaaggggactcattgcacgaacatattgcgaatcttgctggttcgccagccatcaaaaagggcatactccagacagtcaaagtagtggatgagctagttaaggtcatgggacgtcacaaaccggaaaacattgtaatcgagatggcacgcgaaaatcaaacgactcagaaggggcaaaaaaacagtcgagagcggatgaagagaatagaagagggtattaaagaactgggcagccagatcttaaaggagcatcctgtggaaaatacccaattgcagaacgagaaactttacctctattacctacaaaatggaagggacatgtatgttgatcaggaactggacataaaccgtttatctgattacgacgtcgatcacattgtaccccaatcctttttgaaggacgattcaatcgacaataaagtgcttacacgctcggataagaaccgagggaaaagtgacaatgttccaagcgaggaagtcgtaaagaaaatgaagaactattggcggcagctcctaaatgcgaaactgataacgcaaagaaagttcgataacttaactaaagctgagaggggtggcttgtctgaacttgacaaggccggatttattaaacgtcagctcgtggaaacccgccaaatcacaaagcatgttgcacagatactagattcccgaatgaatacgaaatacgacgagaacgataagctgattcgggaagtcaaagtaatcactttaaagtcaaaattggtgtcggacttcagaaaggattttcaattctataaagttagggagataaataactaccaccatgcgcacgacgcttatcttaatgccgtcgtagggaccgcactcattaagaaatacccgaagctagaaagtgagtttgtgtatggtgattacaaagtttatgacgtccgtaagatgatcgcgaaaagcgaacaggagataggcaaggctacagccaaatacttcttttattctaacattatgaatttctttaagacggaaatcactctggcaaacggagagatacgcaaacgacctttaattgaaaccaatggggagacaggtgaaatcgtatgggataagggccgggacttcgcgacggtgagaaaagttttgtccatgccccaagtcaacatagtaaagaaaactgaggtgcagaccggagggttttcaaaggaatcgattcttccaaaaaggaatagtgataagctcatcgctcgtaaaaaggactgggacccgaaaaagtacggtggcttcgatagccctacagttgcctattctgtcctagtagtggcaaaagttgagaagggaaaatccaagaaactgaagtcagtcaaagaattattggggataacgattatggagcgctcgtcttttgaaaagaaccccatcgacttccttgaggcgaaaggttacaaggaagtaaaaaaggatctcataattaaactaccaaagtatagtctgtttgagttagaaaatggccgaaaacggatgttggctagcgccggagagcttcaaaaggggaacgaactcgcactaccgtctaaatacgtgaatttcctgtatttagcgtcccattacgagaagttgaaaggttcacctgaagataacgaacagaagcaactttttgttgagcagcacaaacattatctcgacgaaatcatagagcaaatttcggaattcagtaagagagtcatcctagctgatgccaatctggacaaagtattaagcgcatacaacaagcacagggataaacccatacgtgagcaggcggaaaatattatccatttgtttactcttaccaacctcggcgctccagccgcattcaagtattttgacacaacgatagatcgcaaacgatacacttctaccaaggaggtgctagacgcgacactgattcaccaatccatcacgggattatatgaaactcggatagatttgtcacagcttgggggtgacggatcccccaagaagaagaggaaagtctcgagcgactacaaagaccatgacggtgattataaagatcatgacatcgattacaaggatgacgatgacaagtgaagcggccgccagctttc

pENTR-L3-Csy4-Cas9n-L2

ttgtacaaagttggcattataagaaagcattgcttatcaatttgttgcaacgaacaggtcactatcagtcaaaataaaatcattatttgccatccagctgcagctctggcccgtgtctcaaaatctctgatgttacattgcacaagataaaaatatatcatcatgaacaataaaactgtctgcttacataaacagtaatacaaggggtgttatgagccatattcaacgggaaacgtcgaggccgcgattaaattccaacatggatgctgatttatatgggtataaatgggctcgcgataatgtcgggcaatcaggtgcgacaatctatcgcttgtatgggaagcccgatgcgccagagttgtttctgaaacatggcaaaggtagcgttgccaatgatgttacagatgagatggtcagactaaactggctgacggaatttatgcctcttccgaccatcaagcattttatccgtactcctgatgatgcatggttactcaccactgcgatccccggaaaaacagcattccaggtattagaagaatatcctgattcaggtgaaaatattgttgatgcgctggcagtgttcctgcgccggttgcattcgattcctgtttgtaattgtccttttaacagcgatcgcgtatttcgtctcgctcaggcgcaatcacgaatgaataacggtttggttgatgcgagtgattttgatgacgagcgtaatggctggcctgttgaacaagtctggaaagaaatgcataaacttttgccattctcaccggattcagtcgtcactcatggtgatttctcacttgataaccttatttttgacgaggggaaattaataggttgtattgatgttggacgagtcggaatcgcagaccgataccaggatcttgccatcctatggaactgcctcggtgagttttctccttcattacagaaacggctttttcaaaaatatggtattgataatcctgatatgaataaattgcagtttcatttgatgctcgatgagtttttctaatcagaattggttaattggttgtaacactggcagagcattacgctgacttgacgggacggcgcaagctcatgaccaaaatcccttaacgtgagttttcgttccactgagcgtcagaccccgtagaaaagatcaaaggatcttcttgagatcctttttttctgcgcgtaatctgctgcttgcaaacaaaaaaaccaccgctaccagcggtggtttgtttgccggatcaagagctaccaactctttttccgaaggtaactggcttcagcagagcgcagataccaaatactgtccttctagtgtagccgtagttaggccaccacttcaagaactctgtagcaccgcctacatacctcgctctgctaatcctgttaccagtggctgctgccagtggcgataagtcgtgtcttaccgggttggactcaagacgatagttaccggataaggcgcagcggtcgggctgaacggggggttcgtgcacacagcccagcttggagcgaacgacctacaccgaactgagatacctacagcgtgagctatgagaaagcgccacgcttcccgaagggagaaaggcggacaggtatccggtaagcggcagggtcggaacaggagagcgcacgagggagcttccagggggaaacgcctggtatctttatagtcctgtcgggtttcgccacctctgacttgagcgtcgatttttgtgatgctcgtcaggggggcggagcctatggaaaaacgccagcaacgcggcctttttacggttcctggccttttgctggccttttgctcacatgttctttcctgcgttatcccctgattctgtggataaccgtattaccgctagccaggaagagtttgtagaaacgcaaaaaggccatccgtcaggatggccttctgcttagtttgatgcctggcagtttatggcgggcgtcctgcccgccaccctccgggccgttgcttcacaacgttcaaatccgctcccggcggatttgtcctactcaggagagcgttcaccgacaaacaacagataaaacgaaaggcccagtcttccgactgagcctttcgttttatttgatgcctggcagttccctactctcgcgttaacgctagcatggatctcgggccccaaataatgattttattttgactgatagtgacctgttcgttgcaacaaattgatgagcaatgcttttttataatgccaactttgtataataaagttgggtaccgcgggcccgggatccaccggtcgccaccatggaccactacctcgacattcgcttgcgaccggacccggaatttcccccggcgcaactcatgagcgtgctcttcggcaagctccaccaggccctggtggcacagggcggggacaggatcggcgtgagcttccccgacctcgacgaaagccgctcccggctgggcgagcgcctgcgcattcatgcctcggcggacgaccttcgtgccctgctcgcccggccctggctggaagggttgcgggaccatctgcaattcggagaaccggcagtcgtgcctcaccccacaccgtaccgtcaggtcagtcgggttcaggcgaaaagcaatccggaacgcctgcggcggcggctcatgcgccggcacgatctgagtgaggaggaggctcggaaacgcattcccgatacggtcgcgagagccttggacctgcccttcgtcacgctacgcagccagagcaccggacagcacttccgtctcttcatccgccacgggccgttgcaggtgacggcagaggaaggaggattcacctgttacgggttgagcaaaggaggtttcgttccctggttcgagggcagaggaagtctgctaacatgcggtgacgtcgaggagaatcctggcccaatggataaaaagtattctattggtttagCcatcggcactaattccgttggatgggctgtcataaccgatgaatacaaagtaccttcaaagaaatttaaggtgttggggaacacagaccgtcattcgattaaaaagaatcttatcggtgccctcctattcgatagtggcgaaacggcagaggcgactcgcctgaaacgaaccgctcggagaaggtatacacgtcgcaagaaccgaatatgttacttacaagaaatttttagcaatgagatggccaaagttgacgattctttctttcaccgtttggaagagtccttccttgtcgaagaggacaagaaacatgaacggcaccccatctttggaaacatagtagatgaggtggcatatcatgaaaagtacccaacgatttatcacctcagaaaaaagctagttgactcaactgataaagcggacctgaggttaatctacttggctcttgcccatatgataaagttccgtgggcactttctcattgagggtgatctaaatccggacaactcggatgtcgacaaactgttcatccagttagtacaaacctataatcagttgtttgaagagaaccctataaatgcaagtggcgtggatgcgaaggctattcttagcgcccgcctctctaaatcccgacggctagaaaacctgatcgcacaattacccggagagaagaaaaatgggttgttcggtaaccttatagcgctctcactaggcctgacaccaaattttaagtcgaacttcgacttagctgaagatgccaaattgcagcttagtaaggacacgtacgatgacgatctcgacaatctactggcacaaattggagatcagtatgcggacttatttttggctgccaaaaaccttagcgatgcaatcctcctatctgacatactgagagttaatactgagattaccaaggcgccgttatccgcttcaatgatcaaaaggtacgatgaacatcaccaagacttgacacttctcaaggccctagtccgtcagcaactgcctgagaaatataaggaaatattctttgatcagtcgaaaaacgggtacgcaggttatattgacggcggagcgagtcaagaggaattctacaagtttatcaaacccatattagagaagatggatgggacggaagagttgcttgtaaaactcaatcgcgaagatctactgcgaaagcagcggactttcgacaacggtagcattccacatcaaatccacttaggcgaattgcatgctatacttagaaggcaggaggatttttatccgttcctcaaagacaatcgtgaaaagattgagaaaatcctaacctttcgcataccttactatgtgggacccctggcccgagggaactctcggttcgcatggatgacaagaaagtccgaagaaacgattactccatggaattttgaggaagttgtcgataaaggtgcgtcagctcaatcgttcatcgagaggatgaccaactttgacaagaatttaccgaacgaaaaagtattgcctaagcacagtttactttacgagtatttcacagtgtacaatgaactcacgaaagttaagtatgtcactgagggcatgcgtaaacccgcctttctaagcggagaacagaagaaagcaatagtagatctgttattcaagaccaaccgcaaagtgacagttaagcaattgaaagaggactactttaagaaaattgaatgcttcgattctgtcgagatctccggggtagaagatcgatttaatgcgtcacttggtacgtatcatgacctcctaaagataattaaagataaggacttcctggataacgaagagaatgaagatatcttagaagatatagtgttgactcttaccctctttgaagatcgggaaatgattgaggaaagactaaaaacatacgctcacctgttcgacgataaggttatgaaacagttaaagaggcgtcgctatacgggctggggacgattgtcgcggaaacttatcaacgggataagagacaagcaaagtggtaaaactattctcgattttctaaagagcgacggcttcgccaataggaactttatgcagctgatccatgatgactctttaaccttcaaagaggatatacaaaaggcacaggtttccggacaaggggactcattgcacgaacatattgcgaatcttgctggttcgccagccatcaaaaagggcatactccagacagtcaaagtagtggatgagctagttaaggtcatgggacgtcacaaaccggaaaacattgtaatcgagatggcacgcgaaaatcaaacgactcagaaggggcaaaaaaacagtcgagagcggatgaagagaatagaagagggtattaaagaactgggcagccagatcttaaaggagcatcctgtggaaaatacccaattgcagaacgagaaactttacctctattacctacaaaatggaagggacatgtatgttgatcaggaactggacataaaccgtttatctgattacgacgtcgatcacattgtaccccaatcctttttgaaggacgattcaatcgacaataaagtgcttacacgctcggataagaaccgagggaaaagtgacaatgttccaagcgaggaagtcgtaaagaaaatgaagaactattggcggcagctcctaaatgcgaaactgataacgcaaagaaagttcgataacttaactaaagctgagaggggtggcttgtctgaacttgacaaggccggatttattaaacgtcagctcgtggaaacccgccaaatcacaaagcatgttgcacagatactagattcccgaatgaatacgaaatacgacgagaacgataagctgattcgggaagtcaaagtaatcactttaaagtcaaaattggtgtcggacttcagaaaggattttcaattctataaagttagggagataaataactaccaccatgcgcacgacgcttatcttaatgccgtcgtagggaccgcactcattaagaaatacccgaagctagaaagtgagtttgtgtatggtgattacaaagtttatgacgtccgtaagatgatcgcgaaaagcgaacaggagataggcaaggctacagccaaatacttcttttattctaacattatgaatttctttaagacggaaatcactctggcaaacggagagatacgcaaacgacctttaattgaaaccaatggggagacaggtgaaatcgtatgggataagggccgggacttcgcgacggtgagaaaagttttgtccatgccccaagtcaacatagtaaagaaaactgaggtgcagaccggagggttttcaaaggaatcgattcttccaaaaaggaatagtgataagctcatcgctcgtaaaaaggactgggacccgaaaaagtacggtggcttcgatagccctacagttgcctattctgtcctagtagtggcaaaagttgagaagggaaaatccaagaaactgaagtcagtcaaagaattattggggataacgattatggagcgctcgtcttttgaaaagaaccccatcgacttccttgaggcgaaaggttacaaggaagtaaaaaaggatctcataattaaactaccaaagtatagtctgtttgagttagaaaatggccgaaaacggatgttggctagcgccggagagcttcaaaaggggaacgaactcgcactaccgtctaaatacgtgaatttcctgtatttagcgtcccattacgagaagttgaaaggttcacctgaagataacgaacagaagcaactttttgttgagcagcacaaacattatctcgacgaaatcatagagcaaatttcggaattcagtaagagagtcatcctagctgatgccaatctggacaaagtattaagcgcatacaacaagcacagggataaacccatacgtgagcaggcggaaaatattatccatttgtttactcttaccaacctcggcgctccagccgcattcaagtattttgacacaacgatagatcgcaaacgatacacttctaccaaggaggtgctagacgcgacactgattcaccaatccatcacgggattatatgaaactcggatagatttgtcacagcttgggggtgacggatcccccaagaagaagaggaaagtctcgagcgactacaaagaccatgacggtgattataaagatcatgacatcgattacaaggatgacgatgacaagtgaagcggccgccagctttc

pENTR-L3-Csy4-FokI-dCas9-L2

ttgtacaaagttggcattataagaaagcattgcttatcaatttgttgcaacgaacaggtcactatcagtcaaaataaaatcattatttgccatccagctgcagctctggcccgtgtctcaaaatctctgatgttacattgcacaagataaaaatatatcatcatgaacaataaaactgtctgcttacataaacagtaatacaaggggtgttatgagccatattcaacgggaaacgtcgaggccgcgattaaattccaacatggatgctgatttatatgggtataaatgggctcgcgataatgtcgggcaatcaggtgcgacaatctatcgcttgtatgggaagcccgatgcgccagagttgtttctgaaacatggcaaaggtagcgttgccaatgatgttacagatgagatggtcagactaaactggctgacggaatttatgcctcttccgaccatcaagcattttatccgtactcctgatgatgcatggttactcaccactgcgatccccggaaaaacagcattccaggtattagaagaatatcctgattcaggtgaaaatattgttgatgcgctggcagtgttcctgcgccggttgcattcgattcctgtttgtaattgtccttttaacagcgatcgcgtatttcgtctcgctcaggcgcaatcacgaatgaataacggtttggttgatgcgagtgattttgatgacgagcgtaatggctggcctgttgaacaagtctggaaagaaatgcataaacttttgccattctcaccggattcagtcgtcactcatggtgatttctcacttgataaccttatttttgacgaggggaaattaataggttgtattgatgttggacgagtcggaatcgcagaccgataccaggatcttgccatcctatggaactgcctcggtgagttttctccttcattacagaaacggctttttcaaaaatatggtattgataatcctgatatgaataaattgcagtttcatttgatgctcgatgagtttttctaatcagaattggttaattggttgtaacactggcagagcattacgctgacttgacgggacggcgcaagctcatgaccaaaatcccttaacgtgagttttcgttccactgagcgtcagaccccgtagaaaagatcaaaggatcttcttgagatcctttttttctgcgcgtaatctgctgcttgcaaacaaaaaaaccaccgctaccagcggtggtttgtttgccggatcaagagctaccaactctttttccgaaggtaactggcttcagcagagcgcagataccaaatactgtccttctagtgtagccgtagttaggccaccacttcaagaactctgtagcaccgcctacatacctcgctctgctaatcctgttaccagtggctgctgccagtggcgataagtcgtgtcttaccgggttggactcaagacgatagttaccggataaggcgcagcggtcgggctgaacggggggttcgtgcacacagcccagcttggagcgaacgacctacaccgaactgagatacctacagcgtgagctatgagaaagcgccacgcttcccgaagggagaaaggcggacaggtatccggtaagcggcagggtcggaacaggagagcgcacgagggagcttccagggggaaacgcctggtatctttatagtcctgtcgggtttcgccacctctgacttgagcgtcgatttttgtgatgctcgtcaggggggcggagcctatggaaaaacgccagcaacgcggcctttttacggttcctggccttttgctggccttttgctcacatgttctttcctgcgttatcccctgattctgtggataaccgtattaccgctagccaggaagagtttgtagaaacgcaaaaaggccatccgtcaggatggccttctgcttagtttgatgcctggcagtttatggcgggcgtcctgcccgccaccctccgggccgttgcttcacaacgttcaaatccgctcccggcggatttgtcctactcaggagagcgttcaccgacaaacaacagataaaacgaaaggcccagtcttccgactgagcctttcgttttatttgatgcctggcagttccctactctcgcgttaacgctagcatggatctcgggccccaaataatgattttattttgactgatagtgacctgttcgttgcaacaaattgatgagcaatgcttttttataatgccaactttgtataataaagttgggtaccgcgggcccgggatccaccggtcgccaccatgggtgatcattatctggatattcggctgaggcctgatccagagttcccacctgcgcagctgatgtctgtcctttttggcaaacttcatcaggccctggttgcccagggcggagatcggataggggtaagctttccagacctcgacgaaagccggagccgcctgggagaacgcctgcggatccacgcttctgccgacgatctgagagccttgctggcaaggccatggcttgaggggctccgggatcacctgcagtttggcgaacccgccgttgttccccacccaaccccttatcggcaggtgtctagagtgcaggccaaatctaatccagaacggctgcgacggcgactcatgcggcgacatgatcttagcgaggaagaggcccgaaaaagaatccctgataccgtggcccgcgcccttgacttgccttttgtcacactgcggtcccagagtacggggcagcatttcagacttttcattcgacacgggccactgcaagttaccgccgaagaaggaggctttacttgttatggactctccaagggaggtttcgtgccctggtttgagggcagaggaagtctgttaacatgcggtgacgtcgaggagaatcctggcccaatgcctaagaagaagcggaaggtgagcagccaacttgtgaagtctgaactcgaggagaaaaaatcagagttgagacacaagttgaagtacgtgccacacgaatacatcgagcttatcgagatcgccagaaacagtacccaggataggatccttgagatgaaagtcatggagttctttatgaaggtctacggttatagaggaaagcaccttggcggtagcagaaagcccgatggcgccatctatactgtcggatctcctatcgattatggggtgatcgtggataccaaagcttactcaggcgggtacaacttgcccataggacaagccgacgagatgcagcggtatgtcgaagagaaccagacgcgcaacaagcacatcaaccccaatgaatggtggaaagtgtacccaagtagtgtgactgagttcaagttcctgtttgtctccggccactttaagggcaattataaagctcagctcactagactcaatcacatcacaaactgcaacggagctgtgttgtcagtggaggagctcctgattggaggcgagatgatcaaagccggcacccttacactggaggaggtgcggcggaagttcaacaatggagagatcaacttcggtggcggtggatccgataaaaagtattctattggtttagccatcggcactaattccgttggatgggctgtcataaccgatgaatacaaagtaccttcaaagaaatttaaggtgttggggaacacagaccgtcattcgattaaaaagaatcttatcggtgccctcctattcgatagtggcgaaacggcagaggcgactcgcctgaaacgaaccgctcggagaaggtatacacgtcgcaagaaccgaatatgttacttacaagaaatttttagcaatgagatggccaaagttgacgattctttctttcaccgtttggaagagtccttccttgtcgaagaggacaagaaacatgaacggcaccccatctttggaaacatagtagatgaggtggcatatcatgaaaagtacccaacgatttatcacctcagaaaaaagctagttgactcaactgataaagcggacctgaggttaatctacttggctcttgcccatatgataaagttccgtgggcactttctcattgagggtgatctaaatccggacaactcggatgtcgacaaactgttcatccagttagtacaaacctataatcagttgtttgaagagaaccctataaatgcaagtggcgtggatgcgaaggctattcttagcgcccgcctctctaaatcccgacggctagaaaacctgatcgcacaattacccggagagaagaaaaatgggttgttcggtaaccttatagcgctctcactaggcctgacaccaaattttaagtcgaacttcgacttagctgaagatgccaaattgcagcttagtaaggacacgtacgatgacgatctcgacaatctactggcacaaattggagatcagtatgcggacttatttttggctgccaaaaaccttagcgatgcaatcctcctatctgacatactgagagttaatactgagattaccaaggcgccgttatccgcttcaatgatcaaaaggtacgatgaacatcaccaagacttgacacttctcaaggccctagtccgtcagcaactgcctgagaaatataaggaaatattctttgatcagtcgaaaaacgggtacgcaggttatattgacggcggagcgagtcaagaggaattctacaagtttatcaaacccatattagagaagatggatgggacggaagagttgcttgtaaaactcaatcgcgaagatctactgcgaaagcagcggactttcgacaacggtagcattccacatcaaatccacttaggcgaattgcatgctatacttagaaggcaggaggatttttatccgttcctcaaagacaatcgtgaaaagattgagaaaatcctaacctttcgcataccttactatgtgggacccctggcccgagggaactctcggttcgcatggatgacaagaaagtccgaagaaacgattactccatggaattttgaggaagttgtcgataaaggtgcgtcagctcaatcgttcatcgagaggatgaccaactttgacaagaatttaccgaacgaaaaagtattgcctaagcacagtttactttacgagtatttcacagtgtacaatgaactcacgaaagttaagtatgtcactgagggcatgcgtaaacccgcctttctaagcggagaacagaagaaagcaatagtagatctgttattcaagaccaaccgcaaagtgacagttaagcaattgaaagaggactactttaagaaaattgaatgcttcgattctgtcgagatctccggggtagaagatcgatttaatgcgtcacttggtacgtatcatgacctcctaaagataattaaagataaggacttcctggataacgaagagaatgaagatatcttagaagatatagtgttgactcttaccctctttgaagatcgggaaatgattgaggaaagactaaaaacatacgctcacctgttcgacgataaggttatgaaacagttaaagaggcgtcgctatacgggctggggacgattgtcgcggaaacttatcaacgggataagagacaagcaaagtggtaaaactattctcgattttctaaagagcgacggcttcgccaataggaactttatgcagctgatccatgatgactctttaaccttcaaagaggatatacaaaaggcacaggtttccggacaaggggactcattgcacgaacatattgcgaatcttgctggttcgccagccatcaaaaagggcatactccagacagtcaaagtagtggatgagctagttaaggtcatgggacgtcacaaaccggaaaacattgtaatcgagatggcacgcgaaaatcaaacgactcagaaggggcaaaaaaacagtcgagagcggatgaagagaatagaagagggtattaaagaactgggcagccagatcttaaaggagcatcctgtggaaaatacccaattgcagaacgagaaactttacctctattacctacaaaatggaagggacatgtatgttgatcaggaactggacataaaccgtttatctgattacgacgtcgatgccattgtaccccaatcctttttgaaggacgattcaatcgacaataaagtgcttacacgctcggataagaaccgagggaaaagtgacaatgttccaagcgaggaagtcgtaaagaaaatgaagaactattggcggcagctcctaaatgcgaaactgataacgcaaagaaagttcgataacttaactaaagctgagaggggtggcttgtctgaacttgacaaggccggatttattaaacgtcagctcgtggaaacccgccaaatcacaaagcatgttgcacagatactagattcccgaatgaatacgaaatacgacgagaacgataagctgattcgggaagtcaaagtaatcactttaaagtcaaaattggtgtcggacttcagaaaggattttcaattctataaagttagggagataaataactaccaccatgcgcacgacgcttatcttaatgccgtcgtagggaccgcactcattaagaaatacccgaagctagaaagtgagtttgtgtatggtgattacaaagtttatgacgtccgtaagatgatcgcgaaaagcgaacaggagataggcaaggctacagccaaatacttcttttattctaacattatgaatttctttaagacggaaatcactctggcaaacggagagatacgcaaacgacctttaattgaaaccaatggggagacaggtgaaatcgtatgggataagggccgggacttcgcgacggtgagaaaagttttgtccatgccccaagtcaacatagtaaagaaaactgaggtgcagaccggagggttttcaaaggaatcgattcttccaaaaaggaatagtgataagctcatcgctcgtaaaaaggactgggacccgaaaaagtacggtggcttcgatagccctacagttgcctattctgtcctagtagtggcaaaagttgagaagggaaaatccaagaaactgaagtcagtcaaagaattattggggataacgattatggagcgctcgtcttttgaaaagaaccccatcgacttccttgaggcgaaaggttacaaggaagtaaaaaaggatctcataattaaactaccaaagtatagtctgtttgagttagaaaatggccgaaaacggatgttggctagcgccggagagcttcaaaaggggaacgaactcgcactaccgtctaaatacgtgaatttcctgtatttagcgtcccattacgagaagttgaaaggttcacctgaagataacgaacagaagcaactttttgttgagcagcacaaacattatctcgacgaaatcatagagcaaatttcggaattcagtaagagagtcatcctagctgatgccaatctggacaaagtattaagcgcatacaacaagcacagggataaacccatacgtgagcaggcggaaaatattatccatttgtttactcttaccaacctcggcgctccagccgcattcaagtattttgacacaacgatagatcgcaaacgatacacttctaccaaggaggtgctagacgcgacactgattcaccaatccatcacgggattatatgaaactcggatagatttgtcacagcttgggggtgacggatcccccaagaagaagaggaaagtctcgagcgactacaaagaccatgacggtgattataaagatcatgacatcgattacaaggatgacgatgacaagtgaagcggccgccagctttc

pENTR-L3-Cas9-EGFP-L2

catggactataaggaccacgacggagactacaaggatcatgatattgattacaaagacgatgacgataagatggccccaaagaagaagcggaaggtcggtatccacggagtcccagcagccgacaagaagtacagcatcggcctggacatcggcaccaactctgtgggctgggccgtgatcaccgacgagtacaaggtgcccagcaagaaattcaaggtgctgggcaacaccgaccggcacagcatcaagaagaacctgatcggagccctgctgttcgacagcggcgaaacagccgaggccacccggctgaagagaaccgccagaagaagatacaccagacggaagaaccggatctgctatctgcaagagatcttcagcaacgagatggccaaggtggacgacagcttcttccacagactggaagagtccttcctggtggaagaggataagaagcacgagcggcaccccatcttcggcaacatcgtggacgaggtggcctaccacgagaagtaccccaccatctaccacctgagaaagaaactggtggacagcaccgacaaggccgacctgcggctgatctatctggccctggcccacatgatcaagttccggggccacttcctgatcgagggcgacctgaaccccgacaacagcgacgtggacaagctgttcatccagctggtgcagacctacaaccagctgttcgaggaaaaccccatcaacgccagcggcgtggacgccaaggccatcctgtctgccagactgagcaagagcagacggctggaaaatctgatcgcccagctgcccggcgagaagaagaatggcctgttcggaaacctgattgccctgagcctgggcctgacccccaacttcaagagcaacttcgacctggccgaggatgccaaactgcagctgagcaaggacacctacgacgacgacctggacaacctgctggcccagatcggcgaccagtacgccgacctgtttctggccgccaagaacctgtccgacgccatcctgctgagcgacatcctgagagtgaacaccgagatcaccaaggcccccctgagcgcctctatgatcaagagatacgacgagcaccaccaggacctgaccctgctgaaagctctcgtgcggcagcagctgcctgagaagtacaaagagattttcttcgaccagagcaagaacggctacgccggctacattgacggcggagccagccaggaagagttctacaagttcatcaagcccatcctggaaaagatggacggcaccgaggaactgctcgtgaagctgaacagagaggacctgctgcggaagcagcggaccttcgacaacggcagcatcccccaccagatccacctgggagagctgcacgccattctgcggcggcaggaagatttttacccattcctgaaggacaaccgggaaaagatcgagaagatcctgaccttccgcatcccctactacgtgggccctctggccaggggaaacagcagattcgcctggatgaccagaaagagcgaggaaaccatcaccccctggaacttcgaggaagtggtggacaagggcgcttccgcccagagcttcatcgagcggatgaccaacttcgataagaacctgcccaacgagaaggtgctgcccaagcacagcctgctgtacgagtacttcaccgtgtataacgagctgaccaaagtgaaatacgtgaccgagggaatgagaaagcccgccttcctgagcggcgagcagaaaaaggccatcgtggacctgctgttcaagaccaaccggaaagtgaccgtgaagcagctgaaagaggactacttcaagaaaatcgagtgcttcgactccgtggaaatctccggcgtggaagatcggttcaacgcctccctgggcacataccacgatctgctgaaaattatcaaggacaaggacttcctggacaatgaggaaaacgaggacattctggaagatatcgtgctgaccctgacactgtttgaggacagagagatgatcgaggaacggctgaaaacctatgcccacctgttcgacgacaaagtgatgaagcagctgaagcggcggagatacaccggctggggcaggctgagccggaagctgatcaacggcatccgggacaagcagtccggcaagacaatcctggatttcctgaagtccgacggcttcgccaacagaaacttcatgcagctgatccacgacgacagcctgacctttaaagaggacatccagaaagcccaggtgtccggccagggcgatagcctgcacgagcacattgccaatctggccggcagccccgccattaagaagggcatcctgcagacagtgaaggtggtggacgagctcgtgaaagtgatgggccggcacaagcccgagaacatcgtgatcgaaatggccagagagaaccagaccacccagaagggacagaagaacagccgcgagagaatgaagcggatcgaagagggcatcaaagagctgggcagccagatcctgaaagaacaccccgtggaaaacacccagctgcagaacgagaagctgtacctgtactacctgcagaatgggcgggatatgtacgtggaccaggaactggacatcaaccggctgtccgactacgatgtggaccatatcgtgcctcagagctttctgaaggacgactccatcgacaacaaggtgctgaccagaagcgacaagaaccggggcaagagcgacaacgtgccctccgaagaggtcgtgaagaagatgaagaactactggcggcagctgctgaacgccaagctgattacccagagaaagttcgacaatctgaccaaggccgagagaggcggcctgagcgaactggataaggccggcttcatcaagagacagctggtggaaacccggcagatcacaaagcacgtggcacagatcctggactcccggatgaacactaagtacgacgagaatgacaagctgatccgggaagtgaaagtgatcaccctgaagtccaagctggtgtccgatttccggaaggatttccagttttacaaagtgcgcgagatcaacaactaccaccacgcccacgacgcctacctgaacgccgtcgtgggaaccgccctgatcaaaaagtaccctaagctggaaagcgagttcgtgtacggcgactacaaggtgtacgacgtgcggaagatgatcgccaagagcgagcaggaaatcggcaaggctaccgccaagtacttcttctacagcaacatcatgaactttttcaagaccgagattaccctggccaacggcgagatccggaagcggcctctgatcgagacaaacggcgaaaccggggagatcgtgtgggataagggccgggattttgccaccgtgcggaaagtgctgagcatgccccaagtgaatatcgtgaaaaagaccgaggtgcagacaggcggcttcagcaaagagtctatcctgcccaagaggaacagcgataagctgatcgccagaaagaaggactgggaccctaagaagtacggcggcttcgacagccccaccgtggcctattctgtgctggtggtggccaaagtggaaaagggcaagtccaagaaactgaagagtgtgaaagagctgctggggatcaccatcatggaaagaagcagcttcgagaagaatcccatcgactttctggaagccaagggctacaaagaagtgaaaaaggacctgatcatcaagctgcctaagtactccctgttcgagctggaaaacggccggaagagaatgctggcctctgccggcgaactgcagaagggaaacgaactggccctgccctccaaatatgtgaacttcctgtacctggccagccactatgagaagctgaagggctcccccgaggataatgagcagaaacagctgtttgtggaacagcacaagcactacctggacgagatcatcgagcagatcagcgagttctccaagagagtgatcctggccgacgctaatctggacaaagtgctgtccgcctacaacaagcaccgggataagcccatcagagagcaggccgagaatatcatccacctgtttaccctgaccaatctgggagcccctgccgccttcaagtactttgacaccaccatcgaccggaagaggtacaccagcaccaaagaggtgctggacgccaccctgatccaccagagcatcaccggcctgtacgagacacggatcgacctgtctcagctgggaggcgacaaaaggccggcggccacgaaaaaggccggccaggcaaaaaagaaaaaggaattcggcagtagccgggcaccggtgaaacagactttgaattttgaccttctcaagttggcgggagacgtggagtccaacccagggccggatatcatggtgagcaagggcgaggagctgttcaccggggtggtgcccatcctggtcgagctggacggcgacgtaaacggccacaagttcagcgtgtccggcgagggcgagggcgatgccacctacggcaagctgaccctgaagttcatctgcaccaccggcaagctgcccgtgccctggcccaccctcgtgaccaccctgacctacggcgtgcagtgcttcagccgctaccccgaccacatgaagcagcacgacttcttcaagtccgccatgcccgaaggctacgtccaggagcgcaccatcttcttcaaggacgacggcaactacaagacccgcgccgaggtgaagttcgagggcgacaccctggtgaaccgcatcgagctgaagggcatcgacttcaaggaggacggcaacatcctggggcacaagctggagtacaactacaacagccacaacgtctatatcatggccgacaagcagaagaacggcatcaaggtgaacttcaagatccgccacaacatcgaggacggcagcgtgcagctcgccgaccactaccagcagaacacccccatcggcgacggccccgtgctgctgcccgacaaccactacctgagcacccagtccgccctgagcaaagaccccaacgagaagcgcgatcacatggtcctgctggagttcgtgaccgccgccgggatcactctcggcatggacgagctgtacaagtaacagctttcttgtacaaagttggcattataagaaagcattgcttatcaatttgttgcaacgaacaggtcactatcagtcaaaataaaatcattatttgccatccagctgcagctctggcccgtgtctcaaaatctctgatgttacattgcacaagataaaaatatatcatcatgaacaataaaactgtctgcttacataaacagtaatacaaggggtgttatgagccatattcaacgggaaacgtcgaggccgcgattaaattccaacatggatgctgatttatatgggtataaatgggctcgcgataatgtcgggcaatcaggtgcgacaatctatcgcttgtatgggaagcccgatgcgccagagttgtttctgaaacatggcaaaggtagcgttgccaatgatgttacagatgagatggtcagactaaactggctgacggaatttatgcctcttccgaccatcaagcattttatccgtactcctgatgatgcatggttactcaccactgcgatccccggaaaaacagcattccaggtattagaagaatatcctgattcaggtgaaaatattgttgatgcgctggcagtgttcctgcgccggttgcattcgattcctgtttgtaattgtccttttaacagcgatcgcgtatttcgtctcgctcaggcgcaatcacgaatgaataacggtttggttgatgcgagtgattttgatgacgagcgtaatggctggcctgttgaacaagtctggaaagaaatgcataaacttttgccattctcaccggattcagtcgtcactcatggtgatttctcacttgataaccttatttttgacgaggggaaattaataggttgtattgatgttggacgagtcggaatcgcagaccgataccaggatcttgccatcctatggaactgcctcggtgagttttctccttcattacagaaacggctttttcaaaaatatggtattgataatcctgatatgaataaattgcagtttcatttgatgctcgatgagtttttctaatcagaattggttaattggttgtaacactggcagagcattacgctgacttgacgggacggcgcaagctcatgaccaaaatcccttaacgtgagttttcgttccactgagcgtcagaccccgtagaaaagatcaaaggatcttcttgagatcctttttttctgcgcgtaatctgctgcttgcaaacaaaaaaaccaccgctaccagcggtggtttgtttgccggatcaagagctaccaactctttttccgaaggtaactggcttcagcagagcgcagataccaaatactgtccttctagtgtagccgtagttaggccaccacttcaagaactctgtagcaccgcctacatacctcgctctgctaatcctgttaccagtggctgctgccagtggcgataagtcgtgtcttaccgggttggactcaagacgatagttaccggataaggcgcagcggtcgggctgaacggggggttcgtgcacacagcccagcttggagcgaacgacctacaccgaactgagatacctacagcgtgagctatgagaaagcgccacgcttcccgaagggagaaaggcggacaggtatccggtaagcggcagggtcggaacaggagagcgcacgagggagcttccagggggaaacgcctggtatctttatagtcctgtcgggtttcgccacctctgacttgagcgtcgatttttgtgatgctcgtcaggggggcggagcctatggaaaaacgccagcaacgcggcctttttacggttcctggccttttgctggccttttgctcacatgttctttcctgcgttatcccctgattctgtggataaccgtattaccgctagccaggaagagtttgtagaaacgcaaaaaggccatccgtcaggatggccttctgcttagtttgatgcctggcagtttatggcgggcgtcctgcccgccaccctccgggccgttgcttcacaacgttcaaatccgctcccggcggatttgtcctactcaggagagcgttcaccgacaaacaacagataaaacgaaaggcccagtcttccgactgagcctttcgttttatttgatgcctggcagttccctactctcgcgttaacgctagcatggatctcgggccccaaataatgattttattttgactgatagtgacctgttcgttgcaacaaattgatgagcaatgcttttttataatgccaactttgtataataaagttggcttcgaaggagatagaac

pENTR-L3-Cas9n-EGFP-L2

catggactataaggaccacgacggagactacaaggatcatgatattgattacaaagacgatgacgataagatggccccaaagaagaagcggaaggtcggtatccacggagtcccagcagccgacaagaagtacagcatcggcctggccatcggcaccaactctgtgggctgggccgtgatcaccgacgagtacaaggtgcccagcaagaaattcaaggtgctgggcaacaccgaccggcacagcatcaagaagaacctgatcggagccctgctgttcgacagcggcgaaacagccgaggccacccggctgaagagaaccgccagaagaagatacaccagacggaagaaccggatctgctatctgcaagagatcttcagcaacgagatggccaaggtggacgacagcttcttccacagactggaagagtccttcctggtggaagaggataagaagcacgagcggcaccccatcttcggcaacatcgtggacgaggtggcctaccacgagaagtaccccaccatctaccacctgagaaagaaactggtggacagcaccgacaaggccgacctgcggctgatctatctggccctggcccacatgatcaagttccggggccacttcctgatcgagggcgacctgaaccccgacaacagcgacgtggacaagctgttcatccagctggtgcagacctacaaccagctgttcgaggaaaaccccatcaacgccagcggcgtggacgccaaggccatcctgtctgccagactgagcaagagcagacggctggaaaatctgatcgcccagctgcccggcgagaagaagaatggcctgttcggcaacctgattgccctgagcctgggcctgacccccaacttcaagagcaacttcgacctggccgaggatgccaaactgcagctgagcaaggacacctacgacgacgacctggacaacctgctggcccagatcggcgaccagtacgccgacctgtttctggccgccaagaacctgtccgacgccatcctgctgagcgacatcctgagagtgaacaccgagatcaccaaggcccccctgagcgcctctatgatcaagagatacgacgagcaccaccaggacctgaccctgctgaaagctctcgtgcggcagcagctgcctgagaagtacaaagagattttcttcgaccagagcaagaacggctacgccggctacattgacggcggagccagccaggaagagttctacaagttcatcaagcccatcctggaaaagatggacggcaccgaggaactgctcgtgaagctgaacagagaggacctgctgcggaagcagcggaccttcgacaacggcagcatcccccaccagatccacctgggagagctgcacgccattctgcggcggcaggaagatttttacccattcctgaaggacaaccgggaaaagatcgagaagatcctgaccttccgcatcccctactacgtgggccctctggccaggggaaacagcagattcgcctggatgaccagaaagagcgaggaaaccatcaccccctggaacttcgaggaagtggtggacaagggcgcttccgcccagagcttcatcgagcggatgaccaacttcgataagaacctgcccaacgagaaggtgctgcccaagcacagcctgctgtacgagtacttcaccgtgtataacgagctgaccaaagtgaaatacgtgaccgagggaatgagaaagcccgccttcctgagcggcgagcagaaaaaggccatcgtggacctgctgttcaagaccaaccggaaagtgaccgtgaagcagctgaaagaggactacttcaagaaaatcgagtgcttcgactccgtggaaatctccggcgtggaagatcggttcaacgcctccctgggcacataccacgatctgctgaaaattatcaaggacaaggacttcctggacaatgaggaaaacgaggacattctggaagatatcgtgctgaccctgacactgtttgaggacagagagatgatcgaggaacggctgaaaacctatgcccacctgttcgacgacaaagtgatgaagcagctgaagcggcggagatacaccggctggggcaggctgagccggaagctgatcaacggcatccgggacaagcagtccggcaagacaatcctggatttcctgaagtccgacggcttcgccaacagaaacttcatgcagctgatccacgacgacagcctgacctttaaagaggacatccagaaagcccaggtgtccggccagggcgatagcctgcacgagcacattgccaatctggccggcagccccgccattaagaagggcatcctgcagacagtgaaggtggtggacgagctcgtgaaagtgatgggccggcacaagcccgagaacatcgtgatcgaaatggccagagagaaccagaccacccagaagggacagaagaacagccgcgagagaatgaagcggatcgaagagggcatcaaagagctgggcagccagatcctgaaagaacaccccgtggaaaacacccagctgcagaacgagaagctgtacctgtactacctgcagaatgggcgggatatgtacgtggaccaggaactggacatcaaccggctgtccgactacgatgtggaccatatcgtgcctcagagctttctgaaggacgactccatcgacaacaaggtgctgaccagaagcgacaagaaccggggcaagagcgacaacgtgccctccgaagaggtcgtgaagaagatgaagaactactggcggcagctgctgaacgccaagctgattacccagagaaagttcgacaatctgaccaaggccgagagaggcggcctgagcgaactggataaggccggcttcatcaagagacagctggtggaaacccggcagatcacaaagcacgtggcacagatcctggactcccggatgaacactaagtacgacgagaatgacaagctgatccgggaagtgaaagtgatcaccctgaagtccaagctggtgtccgatttccggaaggatttccagttttacaaagtgcgcgagatcaacaactaccaccacgcccacgacgcctacctgaacgccgtcgtgggaaccgccctgatcaaaaagtaccctaagctggaaagcgagttcgtgtacggcgactacaaggtgtacgacgtgcggaagatgatcgccaagagcgagcaggaaatcggcaaggctaccgccaagtacttcttctacagcaacatcatgaactttttcaagaccgagattaccctggccaacggcgagatccggaagcggcctctgatcgagacaaacggcgaaaccggggagatcgtgtgggataagggccgggattttgccaccgtgcggaaagtgctgagcatgccccaagtgaatatcgtgaaaaagaccgaggtgcagacaggcggcttcagcaaagagtctatcctgcccaagaggaacagcgataagctgatcgccagaaagaaggactgggaccctaagaagtacggcggcttcgacagccccaccgtggcctattctgtgctggtggtggccaaagtggaaaagggcaagtccaagaaactgaagagtgtgaaagagctgctggggatcaccatcatggaaagaagcagcttcgagaagaatcccatcgactttctggaagccaagggctacaaagaagtgaaaaaggacctgatcatcaagctgcctaagtactccctgttcgagctggaaaacggccggaagagaatgctggcctctgccggcgaactgcagaagggaaacgaactggccctgccctccaaatatgtgaacttcctgtacctggccagccactatgagaagctgaagggctcccccgaggataatgagcagaaacagctgtttgtggaacagcacaagcactacctggacgagatcatcgagcagatcagcgagttctccaagagagtgatcctggccgacgctaatctggacaaagtgctgtccgcctacaacaagcaccgggataagcccatcagagagcaggccgagaatatcatccacctgtttaccctgaccaatctgggagcccctgccgccttcaagtactttgacaccaccatcgaccggaagaggtacaccagcaccaaagaggtgctggacgccaccctgatccaccagagcatcaccggcctgtacgagacacggatcgacctgtctcagctgggaggcgacaaaaggccggcggccacgaaaaaggccggccaggcaaaaaagaaaaaggaattcggcagtagccgggcaccggtgaaacagactttgaattttgaccttctcaagttggcgggagacgtggagtccaacccagggccggatatcatggtgagcaagggcgaggagctgttcaccggggtggtgcccatcctggtcgagctggacggcgacgtaaacggccacaagttcagcgtgtccggcgagggcgagggcgatgccacctacggcaagctgaccctgaagttcatctgcaccaccggcaagctgcccgtgccctggcccaccctcgtgaccaccctgacctacggcgtgcagtgcttcagccgctaccccgaccacatgaagcagcacgacttcttcaagtccgccatgcccgaaggctacgtccaggagcgcaccatcttcttcaaggacgacggcaactacaagacccgcgccgaggtgaagttcgagggcgacaccctggtgaaccgcatcgagctgaagggcatcgacttcaaggaggacggcaacatcctggggcacaagctggagtacaactacaacagccacaacgtctatatcatggccgacaagcagaagaacggcatcaaggtgaacttcaagatccgccacaacatcgaggacggcagcgtgcagctcgccgaccactaccagcagaacacccccatcggcgacggccccgtgctgctgcccgacaaccactacctgagcacccagtccgccctgagcaaagaccccaacgagaagcgcgatcacatggtcctgctggagttcgtgaccgccgccgggatcactctcggcatggacgagctgtacaagtaacagctttcttgtacaaagttggcattataagaaagcattgcttatcaatttgttgcaacgaacaggtcactatcagtcaaaataaaatcattatttgccatccagctgcagctctggcccgtgtctcaaaatctctgatgttacattgcacaagataaaaatatatcatcatgaacaataaaactgtctgcttacataaacagtaatacaaggggtgttatgagccatattcaacgggaaacgtcgaggccgcgattaaattccaacatggatgctgatttatatgggtataaatgggctcgcgataatgtcgggcaatcaggtgcgacaatctatcgcttgtatgggaagcccgatgcgccagagttgtttctgaaacatggcaaaggtagcgttgccaatgatgttacagatgagatggtcagactaaactggctgacggaatttatgcctcttccgaccatcaagcattttatccgtactcctgatgatgcatggttactcaccactgcgatccccggaaaaacagcattccaggtattagaagaatatcctgattcaggtgaaaatattgttgatgcgctggcagtgttcctgcgccggttgcattcgattcctgtttgtaattgtccttttaacagcgatcgcgtatttcgtctcgctcaggcgcaatcacgaatgaataacggtttggttgatgcgagtgattttgatgacgagcgtaatggctggcctgttgaacaagtctggaaagaaatgcataaacttttgccattctcaccggattcagtcgtcactcatggtgatttctcacttgataaccttatttttgacgaggggaaattaataggttgtattgatgttggacgagtcggaatcgcagaccgataccaggatcttgccatcctatggaactgcctcggtgagttttctccttcattacagaaacggctttttcaaaaatatggtattgataatcctgatatgaataaattgcagtttcatttgatgctcgatgagtttttctaatcagaattggttaattggttgtaacactggcagagcattacgctgacttgacgggacggcgcaagctcatgaccaaaatcccttaacgtgagttttcgttccactgagcgtcagaccccgtagaaaagatcaaaggatcttcttgagatcctttttttctgcgcgtaatctgctgcttgcaaacaaaaaaaccaccgctaccagcggtggtttgtttgccggatcaagagctaccaactctttttccgaaggtaactggcttcagcagagcgcagataccaaatactgtccttctagtgtagccgtagttaggccaccacttcaagaactctgtagcaccgcctacatacctcgctctgctaatcctgttaccagtggctgctgccagtggcgataagtcgtgtcttaccgggttggactcaagacgatagttaccggataaggcgcagcggtcgggctgaacggggggttcgtgcacacagcccagcttggagcgaacgacctacaccgaactgagatacctacagcgtgagctatgagaaagcgccacgcttcccgaagggagaaaggcggacaggtatccggtaagcggcagggtcggaacaggagagcgcacgagggagcttccagggggaaacgcctggtatctttatagtcctgtcgggtttcgccacctctgacttgagcgtcgatttttgtgatgctcgtcaggggggcggagcctatggaaaaacgccagcaacgcggcctttttacggttcctggccttttgctggccttttgctcacatgttctttcctgcgttatcccctgattctgtggataaccgtattaccgctagccaggaagagtttgtagaaacgcaaaaaggccatccgtcaggatggccttctgcttagtttgatgcctggcagtttatggcgggcgtcctgcccgccaccctccgggccgttgcttcacaacgttcaaatccgctcccggcggatttgtcctactcaggagagcgttcaccgacaaacaacagataaaacgaaaggcccagtcttccgactgagcctttcgttttatttgatgcctggcagttccctactctcgcgttaacgctagcatggatctcgggccccaaataatgattttattttgactgatagtgacctgttcgttgcaacaaattgatgagcaatgcttttttataatgccaactttgtataataaagttggcttcgaaggagatagaac

pENTR-L3-Csy4-Cas9-EGFP-L2

tgcacacagcccagcttggagcgaacgacctacaccgaactgagatacctacagcgtgagctatgagaaagcgccacgcttcccgaagggagaaaggcggacaggtatccggtaagcggcagggtcggaacaggagagcgcacgagggagcttccagggggaaacgcctggtatctttatagtcctgtcgggtttcgccacctctgacttgagcgtcgatttttgtgatgctcgtcaggggggcggagcctatggaaaaacgccagcaacgcggcctttttacggttcctggccttttgctggccttttgctcacatgttctttcctgcgttatcccctgattctgtggataaccgtattaccgctagccaggaagagtttgtagaaacgcaaaaaggccatccgtcaggatggccttctgcttagtttgatgcctggcagtttatggcgggcgtcctgcccgccaccctccgggccgttgcttcacaacgttcaaatccgctcccggcggatttgtcctactcaggagagcgttcaccgacaaacaacagataaaacgaaaggcccagtcttccgactgagcctttcgttttatttgatgcctggcagttccctactctcgcgttaacgctagcatggatctcgggccccaaataatgattttattttgactgatagtgacctgttcgttgcaacaaattgatgagcaatgcttttttataatgccaactttgtataataaagttgggtaccgcgggcccgggatccaccggtcgccaccatggaccactacctcgacattcgcttgcgaccggacccggaatttcccccggcgcaactcatgagcgtgctcttcggcaagctccaccaggccctggtggcacagggcggggacaggatcggcgtgagcttccccgacctcgacgaaagccgctcccggctgggcgagcgcctgcgcattcatgcctcggcggacgaccttcgtgccctgctcgcccggccctggctggaagggttgcgggaccatctgcaattcggagaaccggcagtcgtgcctcaccccacaccgtaccgtcaggtcagtcgggttcaggcgaaaagcaatccggaacgcctgcggcggcggctcatgcgccggcacgatctgagtgaggaggaggctcggaaacgcattcccgatacggtcgcgagagccttggacctgcccttcgtcacgctacgcagccagagcaccggacagcacttccgtctcttcatccgccacgggccgttgcaggtgacggcagaggaaggaggattcacctgttacgggttgagcaaaggaggtttcgttccctggttcgagggcagaggaagtctgctaacatgcggtgacgtcgaggagaatcctggcccaatggataaaaagtattctattggtttagacatcggcactaattccgttggatgggctgtcataaccgatgaatacaaagtaccttcaaagaaatttaaggtgttggggaacacagaccgtcattcgattaaaaagaatcttatcggtgccctcctattcgatagtggcgaaacggcagaggcgactcgcctgaaacgaaccgctcggagaaggtatacacgtcgcaagaaccgaatatgttacttacaagaaatttttagcaatgagatggccaaagttgacgattctttctttcaccgtttggaagagtccttccttgtcgaagaggacaagaaacatgaacggcaccccatctttggaaacatagtagatgaggtggcatatcatgaaaagtacccaacgatttatcacctcagaaaaaagctagttgactcaactgataaagcggacctgaggttaatctacttggctcttgcccatatgataaagttccgtgggcactttctcattgagggtgatctaaatccggacaactcggatgtcgacaaactgttcatccagttagtacaaacctataatcagttgtttgaagagaaccctataaatgcaagtggcgtggatgcgaaggctattcttagcgcccgcctctctaaatcccgacggctagaaaacctgatcgcacaattacccggagagaagaaaaatgggttgttcggtaaccttatagcgctctcactaggcctgacaccaaattttaagtcgaacttcgacttagctgaagatgccaaattgcagcttagtaaggacacgtacgatgacgatctcgacaatctactggcacaaattggagatcagtatgcggacttatttttggctgccaaaaaccttagcgatgcaatcctcctatctgacatactgagagttaatactgagattaccaaggcgccgttatccgcttcaatgatcaaaaggtacgatgaacatcaccaagacttgacacttctcaaggccctagtccgtcagcaactgcctgagaaatataaggaaatattctttgatcagtcgaaaaacgggtacgcaggttatattgacggcggagcgagtcaagaggaattctacaagtttatcaaacccatattagagaagatggatgggacggaagagttgcttgtaaaactcaatcgcgaagatctactgcgaaagcagcggactttcgacaacggtagcattccacatcaaatccacttaggcgaattgcatgctatacttagaaggcaggaggatttttatccgttcctcaaagacaatcgtgaaaagattgagaaaatcctaacctttcgcataccttactatgtgggacccctggcccgagggaactctcggttcgcatggatgacaagaaagtccgaagaaacgattactccatggaattttgaggaagttgtcgataaaggtgcgtcagctcaatcgttcatcgagaggatgaccaactttgacaagaatttaccgaacgaaaaagtattgcctaagcacagtttactttacgagtatttcacagtgtacaatgaactcacgaaagttaagtatgtcactgagggcatgcgtaaacccgcctttctaagcggagaacagaagaaagcaatagtagatctgttattcaagaccaaccgcaaagtgacagttaagcaattgaaagaggactactttaagaaaattgaatgcttcgattctgtcgagatctccggggtagaagatcgatttaatgcgtcacttggtacgtatcatgacctcctaaagataattaaagataaggacttcctggataacgaagagaatgaagatatcttagaagatatagtgttgactcttaccctctttgaagatcgggaaatgattgaggaaagactaaaaacatacgctcacctgttcgacgataaggttatgaaacagttaaagaggcgtcgctatacgggctggggacgattgtcgcggaaacttatcaacgggataagagacaagcaaagtggtaaaactattctcgattttctaaagagcgacggcttcgccaataggaactttatgcagctgatccatgatgactctttaaccttcaaagaggatatacaaaaggcacaggtttccggacaaggggactcattgcacgaacatattgcgaatcttgctggttcgccagccatcaaaaagggcatactccagacagtcaaagtagtggatgagctagttaaggtcatgggacgtcacaaaccggaaaacattgtaatcgagatggcacgcgaaaatcaaacgactcagaaggggcaaaaaaacagtcgagagcggatgaagagaatagaagagggtattaaagaactgggcagccagatcttaaaggagcatcctgtggaaaatacccaattgcagaacgagaaactttacctctattacctacaaaatggaagggacatgtatgttgatcaggaactggacataaaccgtttatctgattacgacgtcgatcacattgtaccccaatcctttttgaaggacgattcaatcgacaataaagtgcttacacgctcggataagaaccgagggaaaagtgacaatgttccaagcgaggaagtcgtaaagaaaatgaagaactattggcggcagctcctaaatgcgaaactgataacgcaaagaaagttcgataacttaactaaagctgagaggggtggcttgtctgaacttgacaaggccggatttattaaacgtcagctcgtggaaacccgccaaatcacaaagcatgttgcacagatactagattcccgaatgaatacgaaatacgacgagaacgataagctgattcgggaagtcaaagtaatcactttaaagtcaaaattggtgtcggacttcagaaaggattttcaattctataaagttagggagataaataactaccaccatgcgcacgacgcttatcttaatgccgtcgtagggaccgcactcattaagaaatacccgaagctagaaagtgagtttgtgtatggtgattacaaagtttatgacgtccgtaagatgatcgcgaaaagcgaacaggagataggcaaggctacagccaaatacttcttttattctaacattatgaatttctttaagacggaaatcactctggcaaacggagagatacgcaaacgacctttaattgaaaccaatggggagacaggtgaaatcgtatgggataagggccgggacttcgcgacggtgagaaaagttttgtccatgccccaagtcaacatagtaaagaaaactgaggtgcagaccggagggttttcaaaggaatcgattcttccaaaaaggaatagtgataagctcatcgctcgtaaaaaggactgggacccgaaaaagtacggtggcttcgatagccctacagttgcctattctgtcctagtagtggcaaaagttgagaagggaaaatccaagaaactgaagtcagtcaaagaattattggggataacgattatggagcgctcgtcttttgaaaagaaccccatcgacttccttgaggcgaaaggttacaaggaagtaaaaaaggatctcataattaaactaccaaagtatagtctgtttgagttagaaaatggccgaaaacggatgttggctagcgccggagagcttcaaaaggggaacgaactcgcactaccgtctaaatacgtgaatttcctgtatttagcgtcccattacgagaagttgaaaggttcacctgaagataacgaacagaagcaactttttgttgagcagcacaaacattatctcgacgaaatcatagagcaaatttcggaattcagtaagagagtcatcctagctgatgccaatctggacaaagtattaagcgcatacaacaagcacagggataaacccatacgtgagcaggcggaaaatattatccatttgtttactcttaccaacctcggcgctccagccgcattcaagtattttgacacaacgatagatcgcaaacgatacacttctaccaaggaggtgctagacgcgacactgattcaccaatccatcacgggattatatgaaactcggatagatttgtcacagcttgggggtgacggatcccccaagaagaagaggaaagtctcgagtagccgcgctaagcgggcaccggtgaaacagactttgaattttgaccttctcaagttggcgggagacgtggagtccaacccagggccggatatcatggtgagcaagggcgaggagctgttcaccggggtggtgcccatcctggtcgagctggacggcgacgtaaacggccacaagttcagcgtgtccggcgagggcgagggcgatgccacctacggcaagctgaccctgaagttcatctgcaccaccggcaagctgcccgtgccctggcccaccctcgtgaccaccctgacctacggcgtgcagtgcttcagccgctaccccgaccacatgaagcagcacgacttcttcaagtccgccatgcccgaaggctacgtccaggagcgcaccatcttcttcaaggacgacggcaactacaagacccgcgccgaggtgaagttcgagggcgacaccctggtgaaccgcatcgagctgaagggcatcgacttcaaggaggacggcaacatcctggggcacaagctggagtacaactacaacagccacaacgtctatatcatggccgacaagcagaagaacggcatcaaggtgaacttcaagatccgccacaacatcgaggacggcagcgtgcagctcgccgaccactaccagcagaacacccccatcggcgacggccccgtgctgctgcccgacaaccactacctgagcacccagtccgccctgagcaaagaccccaacgagaagcgcgatcacatggtcctgctggagttcgtgaccgccgccgggatcactctcggcatggacgagctgtacaagtaacagctttcttgtacaaagttggcattataagaaagcattgcttatcaatttgttgcaacgaacaggtcactatcagtcaaaataaaatcattatttgccatccagctgcagctctggcccgtgtctcaaaatctctgatgttacattgcacaagataaaaatatatcatcatgaacaataaaactgtctgcttacataaacagtaatacaaggggtgttatgagccatattcaacgggaaacgtcgaggccgcgattaaattccaacatggatgctgatttatatgggtataaatgggctcgcgataatgtcgggcaatcaggtgcgacaatctatcgcttgtatgggaagcccgatgcgccagagttgtttctgaaacatggcaaaggtagcgttgccaatgatgttacagatgagatggtcagactaaactggctgacggaatttatgcctcttccgaccatcaagcattttatccgtactcctgatgatgcatggttactcaccactgcgatccccggaaaaacagcattccaggtattagaagaatatcctgattcaggtgaaaatattgttgatgcgctggcagtgttcctgcgccggttgcattcgattcctgtttgtaattgtccttttaacagcgatcgcgtatttcgtctcgctcaggcgcaatcacgaatgaataacggtttggttgatgcgagtgattttgatgacgagcgtaatggctggcctgttgaacaagtctggaaagaaatgcataaacttttgccattctcaccggattcagtcgtcactcatggtgatttctcacttgataaccttatttttgacgaggggaaattaataggttgtattgatgttggacgagtcggaatcgcagaccgataccaggatcttgccatcctatggaactgcctcggtgagttttctccttcattacagaaacggctttttcaaaaatatggtattgataatcctgatatgaataaattgcagtttcatttgatgctcgatgagtttttctaatcagaattggttaattggttgtaacactggcagagcattacgctgacttgacgggacggcgcaagctcatgaccaaaatcccttaacgtgagttttcgttccactgagcgtcagaccccgtagaaaagatcaaaggatcttcttgagatcctttttttctgcgcgtaatctgctgcttgcaaacaaaaaaaccaccgctaccagcggtggtttgtttgccggatcaagagctaccaactctttttccgaaggtaactggcttcagcagagcgcagataccaaatactgtccttctagtgtagccgtagttaggccaccacttcaagaactctgtagcaccgcctacatacctcgctctgctaatcctgttaccagtggctgctgccagtggcgataagtcgtgtcttaccgggttggactcaagacgatagttaccggataaggcgcagcggtcgggctgaacggggggttcg

pENTR-L3-Csy4-Cas9n-EGFP-L2

tgcacacagcccagcttggagcgaacgacctacaccgaactgagatacctacagcgtgagctatgagaaagcgccacgcttcccgaagggagaaaggcggacaggtatccggtaagcggcagggtcggaacaggagagcgcacgagggagcttccagggggaaacgcctggtatctttatagtcctgtcgggtttcgccacctctgacttgagcgtcgatttttgtgatgctcgtcaggggggcggagcctatggaaaaacgccagcaacgcggcctttttacggttcctggccttttgctggccttttgctcacatgttctttcctgcgttatcccctgattctgtggataaccgtattaccgctagccaggaagagtttgtagaaacgcaaaaaggccatccgtcaggatggccttctgcttagtttgatgcctggcagtttatggcgggcgtcctgcccgccaccctccgggccgttgcttcacaacgttcaaatccgctcccggcggatttgtcctactcaggagagcgttcaccgacaaacaacagataaaacgaaaggcccagtcttccgactgagcctttcgttttatttgatgcctggcagttccctactctcgcgttaacgctagcatggatctcgggccccaaataatgattttattttgactgatagtgacctgttcgttgcaacaaattgatgagcaatgcttttttataatgccaactttgtataataaagttgggtaccgcgggcccgggatccaccggtcgccaccatggaccactacctcgacattcgcttgcgaccggacccggaatttcccccggcgcaactcatgagcgtgctcttcggcaagctccaccaggccctggtggcacagggcggggacaggatcggcgtgagcttccccgacctcgacgaaagccgctcccggctgggcgagcgcctgcgcattcatgcctcggcggacgaccttcgtgccctgctcgcccggccctggctggaagggttgcgggaccatctgcaattcggagaaccggcagtcgtgcctcaccccacaccgtaccgtcaggtcagtcgggttcaggcgaaaagcaatccggaacgcctgcggcggcggctcatgcgccggcacgatctgagtgaggaggaggctcggaaacgcattcccgatacggtcgcgagagccttggacctgcccttcgtcacgctacgcagccagagcaccggacagcacttccgtctcttcatccgccacgggccgttgcaggtgacggcagaggaaggaggattcacctgttacgggttgagcaaaggaggtttcgttccctggttcgagggcagaggaagtctgttaacatgcggtgacgtcgaggagaatcctggcccaatggataaaaagtattctattggtttagccatcggcactaattccgttggatgggctgtcataaccgatgaatacaaagtaccttcaaagaaatttaaggtgttggggaacacagaccgtcattcgattaaaaagaatcttatcggtgccctcctattcgatagtggcgaaacggcagaggcgactcgcctgaaacgaaccgctcggagaaggtatacacgtcgcaagaaccgaatatgttacttacaagaaatttttagcaatgagatggccaaagttgacgattctttctttcaccgtttggaagagtccttccttgtcgaagaggacaagaaacatgaacggcaccccatctttggaaacatagtagatgaggtggcatatcatgaaaagtacccaacgatttatcacctcagaaaaaagctagttgactcaactgataaagcggacctgaggttaatctacttggctcttgcccatatgataaagttccgtgggcactttctcattgagggtgatctaaatccggacaactcggatgtcgacaaactgttcatccagttagtacaaacctataatcagttgtttgaagagaaccctataaatgcaagtggcgtggatgcgaaggctattcttagcgcccgcctctctaaatcccgacggctagaaaacctgatcgcacaattacccggagagaagaaaaatgggttgttcggtaaccttatagcgctctcactaggcctgacaccaaattttaagtcgaacttcgacttagctgaagatgccaaattgcagcttagtaaggacacgtacgatgacgatctcgacaatctactggcacaaattggagatcagtatgcggacttatttttggctgccaaaaaccttagcgatgcaatcctcctatctgacatactgagagttaatactgagattaccaaggcgccgttatccgcttcaatgatcaaaaggtacgatgaacatcaccaagacttgacacttctcaaggccctagtccgtcagcaactgcctgagaaatataaggaaatattctttgatcagtcgaaaaacgggtacgcaggttatattgacggcggagcgagtcaagaggaattctacaagtttatcaaacccatattagagaagatggatgggacggaagagttgcttgtaaaactcaatcgcgaagatctactgcgaaagcagcggactttcgacaacggtagcattccacatcaaatccacttaggcgaattgcatgctatacttagaaggcaggaggatttttatccgttcctcaaagacaatcgtgaaaagattgagaaaatcctaacctttcgcataccttactatgtgggacccctggcccgagggaactctcggttcgcatggatgacaagaaagtccgaagaaacgattactccatggaattttgaggaagttgtcgataaaggtgcgtcagctcaatcgttcatcgagaggatgaccaactttgacaagaatttaccgaacgaaaaagtattgcctaagcacagtttactttacgagtatttcacagtgtacaatgaactcacgaaagttaagtatgtcactgagggcatgcgtaaacccgcctttctaagcggagaacagaagaaagcaatagtagatctgttattcaagaccaaccgcaaagtgacagttaagcaattgaaagaggactactttaagaaaattgaatgcttcgattctgtcgagatctccggggtagaagatcgatttaatgcgtcacttggtacgtatcatgacctcctaaagataattaaagataaggacttcctggataacgaagagaatgaagatatcttagaagatatagtgttgactcttaccctctttgaagatcgggaaatgattgaggaaagactaaaaacatacgctcacctgttcgacgataaggttatgaaacagttaaagaggcgtcgctatacgggctggggacgattgtcgcggaaacttatcaacgggataagagacaagcaaagtggtaaaactattctcgattttctaaagagcgacggcttcgccaataggaactttatgcagctgatccatgatgactctttaaccttcaaagaggatatacaaaaggcacaggtttccggacaaggggactcattgcacgaacatattgcgaatcttgctggttcgccagccatcaaaaagggcatactccagacagtcaaagtagtggatgagctagttaaggtcatgggacgtcacaaaccggaaaacattgtaatcgagatggcacgcgaaaatcaaacgactcagaaggggcaaaaaaacagtcgagagcggatgaagagaatagaagagggtattaaagaactgggcagccagatcttaaaggagcatcctgtggaaaatacccaattgcagaacgagaaactttacctctattacctacaaaatggaagggacatgtatgttgatcaggaactggacataaaccgtttatctgattacgacgtcgatcacattgtaccccaatcctttttgaaggacgattcaatcgacaataaagtgcttacacgctcggataagaaccgagggaaaagtgacaatgttccaagcgaggaagtcgtaaagaaaatgaagaactattggcggcagctcctaaatgcgaaactgataacgcaaagaaagttcgataacttaactaaagctgagaggggtggcttgtctgaacttgacaaggccggatttattaaacgtcagctcgtggaaacccgccaaatcacaaagcatgttgcacagatactagattcccgaatgaatacgaaatacgacgagaacgataagctgattcgggaagtcaaagtaatcactttaaagtcaaaattggtgtcggacttcagaaaggattttcaattctataaagttagggagataaataactaccaccatgcgcacgacgcttatcttaatgccgtcgtagggaccgcactcattaagaaatacccgaagctagaaagtgagtttgtgtatggtgattacaaagtttatgacgtccgtaagatgatcgcgaaaagcgaacaggagataggcaaggctacagccaaatacttcttttattctaacattatgaatttctttaagacggaaatcactctggcaaacggagagatacgcaaacgacctttaattgaaaccaatggggagacaggtgaaatcgtatgggataagggccgggacttcgcgacggtgagaaaagttttgtccatgccccaagtcaacatagtaaagaaaactgaggtgcagaccggagggttttcaaaggaatcgattcttccaaaaaggaatagtgataagctcatcgctcgtaaaaaggactgggacccgaaaaagtacggtggcttcgatagccctacagttgcctattctgtcctagtagtggcaaaagttgagaagggaaaatccaagaaactgaagtcagtcaaagaattattggggataacgattatggagcgctcgtcttttgaaaagaaccccatcgacttccttgaggcgaaaggttacaaggaagtaaaaaaggatctcataattaaactaccaaagtatagtctgtttgagttagaaaatggccgaaaacggatgttggctagcgccggagagcttcaaaaggggaacgaactcgcactaccgtctaaatacgtgaatttcctgtatttagcgtcccattacgagaagttgaaaggttcacctgaagataacgaacagaagcaactttttgttgagcagcacaaacattatctcgacgaaatcatagagcaaatttcggaattcagtaagagagtcatcctagctgatgccaatctggacaaagtattaagcgcatacaacaagcacagggataaacccatacgtgagcaggcggaaaatattatccatttgtttactcttaccaacctcggcgctccagccgcattcaagtattttgacacaacgatagatcgcaaacgatacacttctaccaaggaggtgctagacgcgacactgattcaccaatccatcacgggattatatgaaactcggatagatttgtcacagcttgggggtgacggatcccccaagaagaagaggaaagtctcgagtagccgcgctaagcgggcaccggtgaaacagactttgaattttgaccttctcaagttggcgggagacgtggagtccaacccagggccggatatcatggtgagcaagggcgaggagctgttcaccggggtggtgcccatcctggtcgagctggacggcgacgtaaacggccacaagttcagcgtgtccggcgagggcgagggcgatgccacctacggcaagctgaccctgaagttcatctgcaccaccggcaagctgcccgtgccctggcccaccctcgtgaccaccctgacctacggcgtgcagtgcttcagccgctaccccgaccacatgaagcagcacgacttcttcaagtccgccatgcccgaaggctacgtccaggagcgcaccatcttcttcaaggacgacggcaactacaagacccgcgccgaggtgaagttcgagggcgacaccctggtgaaccgcatcgagctgaagggcatcgacttcaaggaggacggcaacatcctggggcacaagctggagtacaactacaacagccacaacgtctatatcatggccgacaagcagaagaacggcatcaaggtgaacttcaagatccgccacaacatcgaggacggcagcgtgcagctcgccgaccactaccagcagaacacccccatcggcgacggccccgtgctgctgcccgacaaccactacctgagcacccagtccgccctgagcaaagaccccaacgagaagcgcgatcacatggtcctgctggagttcgtgaccgccgccgggatcactctcggcatggacgagctgtacaagtaacagctttcttgtacaaagttggcattataagaaagcattgcttatcaatttgttgcaacgaacaggtcactatcagtcaaaataaaatcattatttgccatccagctgcagctctggcccgtgtctcaaaatctctgatgttacattgcacaagataaaaatatatcatcatgaacaataaaactgtctgcttacataaacagtaatacaaggggtgttatgagccatattcaacgggaaacgtcgaggccgcgattaaattccaacatggatgctgatttatatgggtataaatgggctcgcgataatgtcgggcaatcaggtgcgacaatctatcgcttgtatgggaagcccgatgcgccagagttgtttctgaaacatggcaaaggtagcgttgccaatgatgttacagatgagatggtcagactaaactggctgacggaatttatgcctcttccgaccatcaagcattttatccgtactcctgatgatgcatggttactcaccactgcgatccccggaaaaacagcattccaggtattagaagaatatcctgattcaggtgaaaatattgttgatgcgctggcagtgttcctgcgccggttgcattcgattcctgtttgtaattgtccttttaacagcgatcgcgtatttcgtctcgctcaggcgcaatcacgaatgaataacggtttggttgatgcgagtgattttgatgacgagcgtaatggctggcctgttgaacaagtctggaaagaaatgcataaacttttgccattctcaccggattcagtcgtcactcatggtgatttctcacttgataaccttatttttgacgaggggaaattaataggttgtattgatgttggacgagtcggaatcgcagaccgataccaggatcttgccatcctatggaactgcctcggtgagttttctccttcattacagaaacggctttttcaaaaatatggtattgataatcctgatatgaataaattgcagtttcatttgatgctcgatgagtttttctaatcagaattggttaattggttgtaacactggcagagcattacgctgacttgacgggacggcgcaagctcatgaccaaaatcccttaacgtgagttttcgttccactgagcgtcagaccccgtagaaaagatcaaaggatcttcttgagatcctttttttctgcgcgtaatctgctgcttgcaaacaaaaaaaccaccgctaccagcggtggtttgtttgccggatcaagagctaccaactctttttccgaaggtaactggcttcagcagagcgcagataccaaatactgtccttctagtgtagccgtagttaggccaccacttcaagaactctgtagcaccgcctacatacctcgctctgctaatcctgttaccagtggctgctgccagtggcgataagtcgtgtcttaccgggttggactcaagacgatagttaccggataaggcgcagcggtcgggctgaacggggggttcg

pENTR-L3-Csy4-FokI-dCas9-EGFP-L2

tgcacacagcccagcttggagcgaacgacctacaccgaactgagatacctacagcgtgagctatgagaaagcgccacgcttcccgaagggagaaaggcggacaggtatccggtaagcggcagggtcggaacaggagagcgcacgagggagcttccagggggaaacgcctggtatctttatagtcctgtcgggtttcgccacctctgacttgagcgtcgatttttgtgatgctcgtcaggggggcggagcctatggaaaaacgccagcaacgcggcctttttacggttcctggccttttgctggccttttgctcacatgttctttcctgcgttatcccctgattctgtggataaccgtattaccgctagccaggaagagtttgtagaaacgcaaaaaggccatccgtcaggatggccttctgcttagtttgatgcctggcagtttatggcgggcgtcctgcccgccaccctccgggccgttgcttcacaacgttcaaatccgctcccggcggatttgtcctactcaggagagcgttcaccgacaaacaacagataaaacgaaaggcccagtcttccgactgagcctttcgttttatttgatgcctggcagttccctactctcgcgttaacgctagcatggatctcgggccccaaataatgattttattttgactgatagtgacctgttcgttgcaacaaattgatgagcaatgcttttttataatgccaactttgtataataaagttgggtaccgcgggcccgggatccaccggtcgccaccatgggtgatcattatctggatattcggctgaggcctgatccagagttcccacctgcgcagctgatgtctgtcctttttggcaaacttcatcaggccctggttgcccagggcggagatcggataggggtaagctttccagacctcgacgaaagccggagccgcctgggagaacgcctgcggatccacgcttctgccgacgatctgagagccttgctggcaaggccatggcttgaggggctccgggatcacctgcagtttggcgaacccgccgttgttccccacccaaccccttatcggcaggtgtctagagtgcaggccaaatctaatccagaacggctgcgacggcgactcatgcggcgacatgatcttagcgaggaagaggcccgaaaaagaatccctgataccgtggcccgcgcccttgacttgccttttgtcacactgcggtcccagagtacggggcagcatttcagacttttcattcgacacgggccactgcaagttaccgccgaagaaggaggctttacttgttatggactctccaagggaggtttcgtgccctggtttgagggcagaggaagtctgttaacatgcggtgacgtcgaggagaatcctggcccaatgcctaagaagaagcggaaggtgagcagccaacttgtgaagtctgaactcgaggagaaaaaatcagagttgagacacaagttgaagtacgtgccacacgaatacatcgagcttatcgagatcgccagaaacagtacccaggataggatccttgagatgaaagtcatggagttctttatgaaggtctacggttatagaggaaagcaccttggcggtagcagaaagcccgatggcgccatctatactgtcggatctcctatcgattatggggtgatcgtggataccaaagcttactcaggcgggtacaacttgcccataggacaagccgacgagatgcagcggtatgtcgaagagaaccagacgcgcaacaagcacatcaaccccaatgaatggtggaaagtgtacccaagtagtgtgactgagttcaagttcctgtttgtctccggccactttaagggcaattataaagctcagctcactagactcaatcacatcacaaactgcaacggagctgtgttgtcagtggaggagctcctgattggaggcgagatgatcaaagccggcacccttacactggaggaggtgcggcggaagttcaacaatggagagatcaacttcggtggcggtggatccgataaaaagtattctattggtttagccatcggcactaattccgttggatgggctgtcataaccgatgaatacaaagtaccttcaaagaaatttaaggtgttggggaacacagaccgtcattcgattaaaaagaatcttatcggtgccctcctattcgatagtggcgaaacggcagaggcgactcgcctgaaacgaaccgctcggagaaggtatacacgtcgcaagaaccgaatatgttacttacaagaaatttttagcaatgagatggccaaagttgacgattctttctttcaccgtttggaagagtccttccttgtcgaagaggacaagaaacatgaacggcaccccatctttggaaacatagtagatgaggtggcatatcatgaaaagtacccaacgatttatcacctcagaaaaaagctagttgactcaactgataaagcggacctgaggttaatctacttggctcttgcccatatgataaagttccgtgggcactttctcattgagggtgatctaaatccggacaactcggatgtcgacaaactgttcatccagttagtacaaacctataatcagttgtttgaagagaaccctataaatgcaagtggcgtggatgcgaaggctattcttagcgcccgcctctctaaatcccgacggctagaaaacctgatcgcacaattacccggagagaagaaaaatgggttgttcggtaaccttatagcgctctcactaggcctgacaccaaattttaagtcgaacttcgacttagctgaagatgccaaattgcagcttagtaaggacacgtacgatgacgatctcgacaatctactggcacaaattggagatcagtatgcggacttatttttggctgccaaaaaccttagcgatgcaatcctcctatctgacatactgagagttaatactgagattaccaaggcgccgttatccgcttcaatgatcaaaaggtacgatgaacatcaccaagacttgacacttctcaaggccctagtccgtcagcaactgcctgagaaatataaggaaatattctttgatcagtcgaaaaacgggtacgcaggttatattgacggcggagcgagtcaagaggaattctacaagtttatcaaacccatattagagaagatggatgggacggaagagttgcttgtaaaactcaatcgcgaagatctactgcgaaagcagcggactttcgacaacggtagcattccacatcaaatccacttaggcgaattgcatgctatacttagaaggcaggaggatttttatccgttcctcaaagacaatcgtgaaaagattgagaaaatcctaacctttcgcataccttactatgtgggacccctggcccgagggaactctcggttcgcatggatgacaagaaagtccgaagaaacgattactccatggaattttgaggaagttgtcgataaaggtgcgtcagctcaatcgttcatcgagaggatgaccaactttgacaagaatttaccgaacgaaaaagtattgcctaagcacagtttactttacgagtatttcacagtgtacaatgaactcacgaaagttaagtatgtcactgagggcatgcgtaaacccgcctttctaagcggagaacagaagaaagcaatagtagatctgttattcaagaccaaccgcaaagtgacagttaagcaattgaaagaggactactttaagaaaattgaatgcttcgattctgtcgagatctccggggtagaagatcgatttaatgcgtcacttggtacgtatcatgacctcctaaagataattaaagataaggacttcctggataacgaagagaatgaagatatcttagaagatatagtgttgactcttaccctctttgaagatcgggaaatgattgaggaaagactaaaaacatacgctcacctgttcgacgataaggttatgaaacagttaaagaggcgtcgctatacgggctggggacgattgtcgcggaaacttatcaacgggataagagacaagcaaagtggtaaaactattctcgattttctaaagagcgacggcttcgccaataggaactttatgcagctgatccatgatgactctttaaccttcaaagaggatatacaaaaggcacaggtttccggacaaggggactcattgcacgaacatattgcgaatcttgctggttcgccagccatcaaaaagggcatactccagacagtcaaagtagtggatgagctagttaaggtcatgggacgtcacaaaccggaaaacattgtaatcgagatggcacgcgaaaatcaaacgactcagaaggggcaaaaaaacagtcgagagcggatgaagagaatagaagagggtattaaagaactgggcagccagatcttaaaggagcatcctgtggaaaatacccaattgcagaacgagaaactttacctctattacctacaaaatggaagggacatgtatgttgatcaggaactggacataaaccgtttatctgattacgacgtcgatgccattgtaccccaatcctttttgaaggacgattcaatcgacaataaagtgcttacacgctcggataagaaccgagggaaaagtgacaatgttccaagcgaggaagtcgtaaagaaaatgaagaactattggcggcagctcctaaatgcgaaactgataacgcaaagaaagttcgataacttaactaaagctgagaggggtggcttgtctgaacttgacaaggccggatttattaaacgtcagctcgtggaaacccgccaaatcacaaagcatgttgcacagatactagattcccgaatgaatacgaaatacgacgagaacgataagctgattcgggaagtcaaagtaatcactttaaagtcaaaattggtgtcggacttcagaaaggattttcaattctataaagttagggagataaataactaccaccatgcgcacgacgcttatcttaatgccgtcgtagggaccgcactcattaagaaatacccgaagctagaaagtgagtttgtgtatggtgattacaaagtttatgacgtccgtaagatgatcgcgaaaagcgaacaggagataggcaaggctacagccaaatacttcttttattctaacattatgaatttctttaagacggaaatcactctggcaaacggagagatacgcaaacgacctttaattgaaaccaatggggagacaggtgaaatcgtatgggataagggccgggacttcgcgacggtgagaaaagttttgtccatgccccaagtcaacatagtaaagaaaactgaggtgcagaccggagggttttcaaaggaatcgattcttccaaaaaggaatagtgataagctcatcgctcgtaaaaaggactgggacccgaaaaagtacggtggcttcgatagccctacagttgcctattctgtcctagtagtggcaaaagttgagaagggaaaatccaagaaactgaagtcagtcaaagaattattggggataacgattatggagcgctcgtcttttgaaaagaaccccatcgacttccttgaggcgaaaggttacaaggaagtaaaaaaggatctcataattaaactaccaaagtatagtctgtttgagttagaaaatggccgaaaacggatgttggctagcgccggagagcttcaaaaggggaacgaactcgcactaccgtctaaatacgtgaatttcctgtatttagcgtcccattacgagaagttgaaaggttcacctgaagataacgaacagaagcaactttttgttgagcagcacaaacattatctcgacgaaatcatagagcaaatttcggaattcagtaagagagtcatcctagctgatgccaatctggacaaagtattaagcgcatacaacaagcacagggataaacccatacgtgagcaggcggaaaatattatccatttgtttactcttaccaacctcggcgctccagccgcattcaagtattttgacacaacgatagatcgcaaacgatacacttctaccaaggaggtgctagacgcgacactgattcaccaatccatcacgggattatatgaaactcggatagatttgtcacagcttgggggtgacggatcccccaagaagaagaggaaagtctcgagtagccgcgctaagcgggcaccggtgaaacagactttgaattttgaccttctcaagttggcgggagacgtggagtccaacccagggccggatatcatggtgagcaagggcgaggagctgttcaccggggtggtgcccatcctggtcgagctggacggcgacgtaaacggccacaagttcagcgtgtccggcgagggcgagggcgatgccacctacggcaagctgaccctgaagttcatctgcaccaccggcaagctgcccgtgccctggcccaccctcgtgaccaccctgacctacggcgtgcagtgcttcagccgctaccccgaccacatgaagcagcacgacttcttcaagtccgccatgcccgaaggctacgtccaggagcgcaccatcttcttcaaggacgacggcaactacaagacccgcgccgaggtgaagttcgagggcgacaccctggtgaaccgcatcgagctgaagggcatcgacttcaaggaggacggcaacatcctggggcacaagctggagtacaactacaacagccacaacgtctatatcatggccgacaagcagaagaacggcatcaaggtgaacttcaagatccgccacaacatcgaggacggcagcgtgcagctcgccgaccactaccagcagaacacccccatcggcgacggccccgtgctgctgcccgacaaccactacctgagcacccagtccgccctgagcaaagaccccaacgagaagcgcgatcacatggtcctgctggagttcgtgaccgccgccgggatcactctcggcatggacgagctgtacaagtaacagctttcttgtacaaagttggcattataagaaagcattgcttatcaatttgttgcaacgaacaggtcactatcagtcaaaataaaatcattatttgccatccagctgcagctctggcccgtgtctcaaaatctctgatgttacattgcacaagataaaaatatatcatcatgaacaataaaactgtctgcttacataaacagtaatacaaggggtgttatgagccatattcaacgggaaacgtcgaggccgcgattaaattccaacatggatgctgatttatatgggtataaatgggctcgcgataatgtcgggcaatcaggtgcgacaatctatcgcttgtatgggaagcccgatgcgccagagttgtttctgaaacatggcaaaggtagcgttgccaatgatgttacagatgagatggtcagactaaactggctgacggaatttatgcctcttccgaccatcaagcattttatccgtactcctgatgatgcatggttactcaccactgcgatccccggaaaaacagcattccaggtattagaagaatatcctgattcaggtgaaaatattgttgatgcgctggcagtgttcctgcgccggttgcattcgattcctgtttgtaattgtccttttaacagcgatcgcgtatttcgtctcgctcaggcgcaatcacgaatgaataacggtttggttgatgcgagtgattttgatgacgagcgtaatggctggcctgttgaacaagtctggaaagaaatgcataaacttttgccattctcaccggattcagtcgtcactcatggtgatttctcacttgataaccttatttttgacgaggggaaattaataggttgtattgatgttggacgagtcggaatcgcagaccgataccaggatcttgccatcctatggaactgcctcggtgagttttctccttcattacagaaacggctttttcaaaaatatggtattgataatcctgatatgaataaattgcagtttcatttgatgctcgatgagtttttctaatcagaattggttaattggttgtaacactggcagagcattacgctgacttgacgggacggcgcaagctcatgaccaaaatcccttaacgtgagttttcgttccactgagcgtcagaccccgtagaaaagatcaaaggatcttcttgagatcctttttttctgcgcgtaatctgctgcttgcaaacaaaaaaaccaccgctaccagcggtggtttgtttgccggatcaagagctaccaactctttttccgaaggtaactggcttcagcagagcgcagataccaaatactgtccttctagtgtagccgtagttaggccaccacttcaagaactctgtagcaccgcctacatacctcgctctgctaatcctgttaccagtggctgctgccagtggcgataagtcgtgtcttaccgggttggactcaagacgatagttaccggataaggcgcagcggtcgggctgaacggggggttcg

pENTR-L3-SpCas9-HF1-2A-EGFP-L2

catggataaaaagtattctattggtttagacatcggcactaattccgttggatgggctgtcataaccgatgaatacaaagtaccttcaaagaaatttaaggtgttggggaacacagaccgtcattcgattaaaaagaatcttatcggtgccctcctattcgatagtggcgaaacggcagaggcgactcgcctgaaacgaaccgctcggagaaggtatacacgtcgcaagaaccgaatatgttacttacaagaaatttttagcaatgagatggccaaagttgacgattctttctttcaccgtttggaagagtccttccttgtcgaagaggacaagaaacatgaacggcaccccatctttggaaacatagtagatgaggtggcatatcatgaaaagtacccaacgatttatcacctcagaaaaaagctagttgactcaactgataaagcggacctgaggttaatctacttggctcttgcccatatgataaagttccgtgggcactttctcattgagggtgatctaaatccggacaactcggatgtcgacaaactgttcatccagttagtacaaacctataatcagttgtttgaagagaaccctataaatgcaagtggcgtggatgcgaaggctattcttagcgcccgcctctctaaatcccgacggctagaaaacctgatcgcacaattacccggagagaagaaaaatgggttgttcggtaaccttatagcgctctcactaggcctgacaccaaattttaagtcgaacttcgacttagctgaagatgccaaattgcagcttagtaaggacacgtacgatgacgatctcgacaatctactggcacaaattggagatcagtatgcggacttatttttggctgccaaaaaccttagcgatgcaatcctcctatctgacatactgagagttaatactgagattaccaaggcgccgttatccgcttcaatgatcaaaaggtacgatgaacatcaccaagacttgacacttctcaaggccctagtccgtcagcaactgcctgagaaatataaggaaatattctttgatcagtcgaaaaacgggtacgcaggttatattgacggcggagcgagtcaagaggaattctacaagtttatcaaacccatattagagaagatggatgggacggaagagttgcttgtaaaactcaatcgcgaagatctactgcgaaagcagcggactttcgacaacggtagcattccacatcaaatccacttaggcgaattgcatgctatacttagaaggcaggaggatttttatccgttcctcaaagacaatcgtgaaaagattgagaaaatcctaacctttcgcataccttactatgtgggacccctggcccgagggaactctcggttcgcatggatgacaagaaagtccgaagaaacgattactccctggaattttgaggaagttgtcgataaaggtgcgtcagctcaatcgttcatcgagaggatgaccgcctttgacaagaatttaccgaacgaaaaagtattgcctaagcacagtttactttacgagtatttcacagtgtacaatgaactcacgaaagttaagtatgtcactgagggcatgcgtaaacccgcctttctaagcggagaacagaagaaagcaatagtagatctgttattcaagaccaaccgcaaagtgacagttaagcaattgaaagaggactactttaagaaaattgaatgcttcgattctgtcgagatctccggggtagaagatcgatttaatgcgtcacttggtacgtatcatgacctcctaaagataattaaagataaggacttcctggataacgaagagaatgaagatatcttagaagatatagtgttgactcttaccctctttgaagatcgggaaatgattgaggaaagactaaaaacatacgctcacctgttcgacgataaggttatgaaacagttaaagaggcgtcgctatacgggctggggagccttgtcgcggaaacttatcaacgggataagagacaagcaaagtggtaaaactattctcgattttctaaagagcgacggcttcgccaataggaactttatggccctgatccatgatgactctttaaccttcaaagaggatatacaaaaggcacaggtttccggacaaggggactcattgcacgaacatattgcgaatcttgctggttcgccagccatcaaaaagggcatactccagacagtcaaagtagtggatgagctagttaaggtcatgggacgtcacaaaccggaaaacattgtaatcgagatggcacgcgaaaatcaaacgactcagaaggggcaaaaaaacagtcgagagcggatgaagagaatagaagagggtattaaagaactgggcagccagatcttaaaggagcatcctgtggaaaatacccaattgcagaacgagaaactttacctctattacctacaaaatggaagggacatgtatgttgatcaggaactggacataaaccgtttatctgattacgacgtcgatcacattgtaccccaatcctttttgaaggacgattcaatcgacaataaagtgcttacacgctcggataagaaccgagggaaaagtgacaatgttccaagcgaggaagtcgtaaagaaaatgaagaactattggcggcagctcctaaatgcgaaactgataacgcaaagaaagttcgataacttaactaaagctgagaggggtggcttgtctgaacttgacaaggccggatttattaaacgtcagctcgtggaaacccgcgccatcacaaagcatgttgcccagatactagattcccgaatgaatacgaaatacgacgagaacgataagctgattcgggaagtcaaagtaatcactttaaagtcaaaattggtgtcggacttcagaaaggattttcaattctataaagttagggagataaataactaccaccatgcgcacgacgcttatcttaatgccgtcgtagggaccgcactcattaagaaatacccgaagctagaaagtgagtttgtgtatggtgattacaaagtttatgacgtccgtaagatgatcgcgaaaagcgaacaggagataggcaaggctacagccaaatacttcttttattctaacattatgaatttctttaagacggaaatcactctggcaaacggagagatacgcaaacgacctttaattgaaaccaatggggagacaggtgaaatcgtatgggataagggccgggacttcgcgacggtgagaaaagttttgtccatgccccaagtcaacatagtaaagaaaactgaggtgcagaccggagggttttcaaaggaatcgattcttccaaaaaggaatagtgataagctcatcgctcgtaaaaaggactgggacccgaaaaagtacggtggcttcgatagccctacagttgcctattctgtcctagtagtggcaaaagttgagaagggaaaatccaagaaactgaagtcagtcaaagaattattggggataacgattatggagcgctcgtcttttgaaaagaaccccatcgacttccttgaggcgaaaggttacaaggaagtaaaaaaggatctcataattaaactaccaaagtatagtctgtttgagttagaaaatggccgaaaacggatgttggctagcgccggagagcttcaaaaggggaacgaactcgcactaccgtctaaatacgtgaatttcctgtatttagcgtcccattacgagaagttgaaaggttcacctgaagataacgaacagaagcaactttttgttgagcagcacaaacattatctcgacgaaatcatagagcaaatttcggaattcagtaagagagtcatcctagctgatgccaatctggacaaagtattaagcgcatacaacaagcacagggataaacccatacgtgagcaggcggaaaatattatccatttgtttactcttaccaacctcggcgctccagccgcattcaagtattttgacacaacgatagatcgcaaacgatacacttctaccaaggaggtgctagacgcgacactgattcaccaatccatcacgggattatatgaaactcggatagatttgtcacagcttgggggtgacggatcccccaagaagaagaggaaagtctcgagtagccgcgctaagcgggcaccggtgaaacagactttgaattttgaccttctcaagttggcgggagacgtggagtccaacccagggccggatatcatggtgagcaagggcgaggagctgttcaccggggtggtgcccatcctggtcgagctggacggcgacgtaaacggccacaagttcagcgtgtccggcgagggcgagggcgatgccacctacggcaagctgaccctgaagttcatctgcaccaccggcaagctgcccgtgccctggcccaccctcgtgaccaccctgacctacggcgtgcagtgcttcagccgctaccccgaccacatgaagcagcacgacttcttcaagtccgccatgcccgaaggctacgtccaggagcgcaccatcttcttcaaggacgacggcaactacaagacccgcgccgaggtgaagttcgagggcgacaccctggtgaaccgcatcgagctgaagggcatcgacttcaaggaggacggcaacatcctggggcacaagctggagtacaactacaacagccacaacgtctatatcatggccgacaagcagaagaacggcatcaaggtgaacttcaagatccgccacaacatcgaggacggcagcgtgcagctcgccgaccactaccagcagaacacccccatcggcgacggccccgtgctgctgcccgacaaccactacctgagcacccagtccgccctgagcaaagaccccaacgagaagcgcgatcacatggtcctgctggagttcgtgaccgccgccgggatcactctcggcatggacgagctgtacaagtaacagctttcttgtacaaagttggcattataagaaagcattgcttatcaatttgttgcaacgaacaggtcactatcagtcaaaataaaatcattatttgccatccagctgcagctctggcccgtgtctcaaaatctctgatgttacattgcacaagataaaaatatatcatcatgaacaataaaactgtctgcttacataaacagtaatacaaggggtgttatgagccatattcaacgggaaacgtcgaggccgcgattaaattccaacatggatgctgatttatatgggtataaatgggctcgcgataatgtcgggcaatcaggtgcgacaatctatcgcttgtatgggaagcccgatgcgccagagttgtttctgaaacatggcaaaggtagcgttgccaatgatgttacagatgagatggtcagactaaactggctgacggaatttatgcctcttccgaccatcaagcattttatccgtactcctgatgatgcatggttactcaccactgcgatccccggaaaaacagcattccaggtattagaagaatatcctgattcaggtgaaaatattgttgatgcgctggcagtgttcctgcgccggttgcattcgattcctgtttgtaattgtccttttaacagcgatcgcgtatttcgtctcgctcaggcgcaatcacgaatgaataacggtttggttgatgcgagtgattttgatgacgagcgtaatggctggcctgttgaacaagtctggaaagaaatgcataaacttttgccattctcaccggattcagtcgtcactcatggtgatttctcacttgataaccttatttttgacgaggggaaattaataggttgtattgatgttggacgagtcggaatcgcagaccgataccaggatcttgccatcctatggaactgcctcggtgagttttctccttcattacagaaacggctttttcaaaaatatggtattgataatcctgatatgaataaattgcagtttcatttgatgctcgatgagtttttctaatcagaattggttaattggttgtaacactggcagagcattacgctgacttgacgggacggcgcaagctcatgaccaaaatcccttaacgtgagttttcgttccactgagcgtcagaccccgtagaaaagatcaaaggatcttcttgagatcctttttttctgcgcgtaatctgctgcttgcaaacaaaaaaaccaccgctaccagcggtggtttgtttgccggatcaagagctaccaactctttttccgaaggtaactggcttcagcagagcgcagataccaaatactgtccttctagtgtagccgtagttaggccaccacttcaagaactctgtagcaccgcctacatacctcgctctgctaatcctgttaccagtggctgctgccagtggcgataagtcgtgtcttaccgggttggactcaagacgatagttaccggataaggcgcagcggtcgggctgaacggggggttcgtgcacacagcccagcttggagcgaacgacctacaccgaactgagatacctacagcgtgagctatgagaaagcgccacgcttcccgaagggagaaaggcggacaggtatccggtaagcggcagggtcggaacaggagagcgcacgagggagcttccagggggaaacgcctggtatctttatagtcctgtcgggtttcgccacctctgacttgagcgtcgatttttgtgatgctcgtcaggggggcggagcctatggaaaaacgccagcaacgcggcctttttacggttcctggccttttgctggccttttgctcacatgttctttcctgcgttatcccctgattctgtggataaccgtattaccgctagccaggaagagtttgtagaaacgcaaaaaggccatccgtcaggatggccttctgcttagtttgatgcctggcagtttatggcgggcgtcctgcccgccaccctccgggccgttgcttcacaacgttcaaatccgctcccggcggatttgtcctactcaggagagcgttcaccgacaaacaacagataaaacgaaaggcccagtcttccgactgagcctttcgttttatttgatgcctggcagttccctactctcgcgttaacgctagcatggatctcgggccccaaataatgattttattttgactgatagtgacctgttcgttgcaacaaattgatgagcaatgcttttttataatgccaactttgtataataaagttggcttcgaaggagatagaac

pENTR-L3-SpCas9-HF4-2A-EGFP-L2

catggataaaaagtattctattggtttagacatcggcactaattccgttggatgggctgtcataaccgatgaatacaaagtaccttcaaagaaatttaaggtgttggggaacacagaccgtcattcgattaaaaagaatcttatcggtgccctcctattcgatagtggcgaaacggcagaggcgactcgcctgaaacgaaccgctcggagaaggtatacacgtcgcaagaaccgaatatgttacttacaagaaatttttagcaatgagatggccaaagttgacgattctttctttcaccgtttggaagagtccttccttgtcgaagaggacaagaaacatgaacggcaccccatctttggaaacatagtagatgaggtggcatatcatgaaaagtacccaacgatttatcacctcagaaaaaagctagttgactcaactgataaagcggacctgaggttaatctacttggctcttgcccatatgataaagttccgtgggcactttctcattgagggtgatctaaatccggacaactcggatgtcgacaaactgttcatccagttagtacaaacctataatcagttgtttgaagagaaccctataaatgcaagtggcgtggatgcgaaggctattcttagcgcccgcctctctaaatcccgacggctagaaaacctgatcgcacaattacccggagagaagaaaaatgggttgttcggtaaccttatagcgctctcactaggcctgacaccaaattttaagtcgaacttcgacttagctgaagatgccaaattgcagcttagtaaggacacgtacgatgacgatctcgacaatctactggcacaaattggagatcagtatgcggacttatttttggctgccaaaaaccttagcgatgcaatcctcctatctgacatactgagagttaatactgagattaccaaggcgccgttatccgcttcaatgatcaaaaggtacgatgaacatcaccaagacttgacacttctcaaggccctagtccgtcagcaactgcctgagaaatataaggaaatattctttgatcagtcgaaaaacgggtacgcaggttatattgacggcggagcgagtcaagaggaattctacaagtttatcaaacccatattagagaagatggatgggacggaagagttgcttgtaaaactcaatcgcgaagatctactgcgaaagcagcggactttcgacaacggtagcattccacatcaaatccacttaggcgaattgcatgctatacttagaaggcaggaggatttttatccgttcctcaaagacaatcgtgaaaagattgagaaaatcctaacctttcgcatacctgcctatgtgggacccctggcccgagggaactctcggttcgcatggatgacaagaaagtccgaagaaacgattactccctggaattttgaggaagttgtcgataaaggtgcgtcagctcaatcgttcatcgagaggatgaccgcctttgacaagaatttaccgaacgaaaaagtattgcctaagcacagtttactttacgagtatttcacagtgtacaatgaactcacgaaagttaagtatgtcactgagggcatgcgtaaacccgcctttctaagcggagaacagaagaaagcaatagtagatctgttattcaagaccaaccgcaaagtgacagttaagcaattgaaagaggactactttaagaaaattgaatgcttcgattctgtcgagatctccggggtagaagatcgatttaatgcgtcacttggtacgtatcatgacctcctaaagataattaaagataaggacttcctggataacgaagagaatgaagatatcttagaagatatagtgttgactcttaccctctttgaagatcgggaaatgattgaggaaagactaaaaacatacgctcacctgttcgacgataaggttatgaaacagttaaagaggcgtcgctatacgggctggggagccttgtcgcggaaacttatcaacgggataagagacaagcaaagtggtaaaactattctcgattttctaaagagcgacggcttcgccaataggaactttatggccctgatccatgatgactctttaaccttcaaagaggatatacaaaaggcacaggtttccggacaaggggactcattgcacgaacatattgcgaatcttgctggttcgccagccatcaaaaagggcatactccagacagtcaaagtagtggatgagctagttaaggtcatgggacgtcacaaaccggaaaacattgtaatcgagatggcacgcgaaaatcaaacgactcagaaggggcaaaaaaacagtcgagagcggatgaagagaatagaagagggtattaaagaactgggcagccagatcttaaaggagcatcctgtggaaaatacccaattgcagaacgagaaactttacctctattacctacaaaatggaagggacatgtatgttgatcaggaactggacataaaccgtttatctgattacgacgtcgatcacattgtaccccaatcctttttgaaggacgattcaatcgacaataaagtgcttacacgctcggataagaaccgagggaaaagtgacaatgttccaagcgaggaagtcgtaaagaaaatgaagaactattggcggcagctcctaaatgcgaaactgataacgcaaagaaagttcgataacttaactaaagctgagaggggtggcttgtctgaacttgacaaggccggatttattaaacgtcagctcgtggaaacccgcgccatcacaaagcatgttgcccagatactagattcccgaatgaatacgaaatacgacgagaacgataagctgattcgggaagtcaaagtaatcactttaaagtcaaaattggtgtcggacttcagaaaggattttcaattctataaagttagggagataaataactaccaccatgcgcacgacgcttatcttaatgccgtcgtagggaccgcactcattaagaaatacccgaagctagaaagtgagtttgtgtatggtgattacaaagtttatgacgtccgtaagatgatcgcgaaaagcgaacaggagataggcaaggctacagccaaatacttcttttattctaacattatgaatttctttaagacggaaatcactctggcaaacggagagatacgcaaacgacctttaattgaaaccaatggggagacaggtgaaatcgtatgggataagggccgggacttcgcgacggtgagaaaagttttgtccatgccccaagtcaacatagtaaagaaaactgaggtgcagaccggagggttttcaaaggaatcgattcttccaaaaaggaatagtgataagctcatcgctcgtaaaaaggactgggacccgaaaaagtacggtggcttcgatagccctacagttgcctattctgtcctagtagtggcaaaagttgagaagggaaaatccaagaaactgaagtcagtcaaagaattattggggataacgattatggagcgctcgtcttttgaaaagaaccccatcgacttccttgaggcgaaaggttacaaggaagtaaaaaaggatctcataattaaactaccaaagtatagtctgtttgagttagaaaatggccgaaaacggatgttggctagcgccggagagcttcaaaaggggaacgaactcgcactaccgtctaaatacgtgaatttcctgtatttagcgtcccattacgagaagttgaaaggttcacctgaagataacgaacagaagcaactttttgttgagcagcacaaacattatctcgacgaaatcatagagcaaatttcggaattcagtaagagagtcatcctagctgatgccaatctggacaaagtattaagcgcatacaacaagcacagggataaacccatacgtgagcaggcggaaaatattatccatttgtttactcttaccaacctcggcgctccagccgcattcaagtattttgacacaacgatagatcgcaaacgatacacttctaccaaggaggtgctagacgcgacactgattcaccaatccatcacgggattatatgaaactcggatagatttgtcacagcttgggggtgacggatcccccaagaagaagaggaaagtctcgagtagccgcgctaagcgggcaccggtgaaacagactttgaattttgaccttctcaagttggcgggagacgtggagtccaacccagggccggatatcatggtgagcaagggcgaggagctgttcaccggggtggtgcccatcctggtcgagctggacggcgacgtaaacggccacaagttcagcgtgtccggcgagggcgagggcgatgccacctacggcaagctgaccctgaagttcatctgcaccaccggcaagctgcccgtgccctggcccaccctcgtgaccaccctgacctacggcgtgcagtgcttcagccgctaccccgaccacatgaagcagcacgacttcttcaagtccgccatgcccgaaggctacgtccaggagcgcaccatcttcttcaaggacgacggcaactacaagacccgcgccgaggtgaagttcgagggcgacaccctggtgaaccgcatcgagctgaagggcatcgacttcaaggaggacggcaacatcctggggcacaagctggagtacaactacaacagccacaacgtctatatcatggccgacaagcagaagaacggcatcaaggtgaacttcaagatccgccacaacatcgaggacggcagcgtgcagctcgccgaccactaccagcagaacacccccatcggcgacggccccgtgctgctgcccgacaaccactacctgagcacccagtccgccctgagcaaagaccccaacgagaagcgcgatcacatggtcctgctggagttcgtgaccgccgccgggatcactctcggcatggacgagctgtacaagtaacagctttcttgtacaaagttggcattataagaaagcattgcttatcaatttgttgcaacgaacaggtcactatcagtcaaaataaaatcattatttgccatccagctgcagctctggcccgtgtctcaaaatctctgatgttacattgcacaagataaaaatatatcatcatgaacaataaaactgtctgcttacataaacagtaatacaaggggtgttatgagccatattcaacgggaaacgtcgaggccgcgattaaattccaacatggatgctgatttatatgggtataaatgggctcgcgataatgtcgggcaatcaggtgcgacaatctatcgcttgtatgggaagcccgatgcgccagagttgtttctgaaacatggcaaaggtagcgttgccaatgatgttacagatgagatggtcagactaaactggctgacggaatttatgcctcttccgaccatcaagcattttatccgtactcctgatgatgcatggttactcaccactgcgatccccggaaaaacagcattccaggtattagaagaatatcctgattcaggtgaaaatattgttgatgcgctggcagtgttcctgcgccggttgcattcgattcctgtttgtaattgtccttttaacagcgatcgcgtatttcgtctcgctcaggcgcaatcacgaatgaataacggtttggttgatgcgagtgattttgatgacgagcgtaatggctggcctgttgaacaagtctggaaagaaatgcataaacttttgccattctcaccggattcagtcgtcactcatggtgatttctcacttgataaccttatttttgacgaggggaaattaataggttgtattgatgttggacgagtcggaatcgcagaccgataccaggatcttgccatcctatggaactgcctcggtgagttttctccttcattacagaaacggctttttcaaaaatatggtattgataatcctgatatgaataaattgcagtttcatttgatgctcgatgagtttttctaatcagaattggttaattggttgtaacactggcagagcattacgctgacttgacgggacggcgcaagctcatgaccaaaatcccttaacgtgagttttcgttccactgagcgtcagaccccgtagaaaagatcaaaggatcttcttgagatcctttttttctgcgcgtaatctgctgcttgcaaacaaaaaaaccaccgctaccagcggtggtttgtttgccggatcaagagctaccaactctttttccgaaggtaactggcttcagcagagcgcagataccaaatactgtccttctagtgtagccgtagttaggccaccacttcaagaactctgtagcaccgcctacatacctcgctctgctaatcctgttaccagtggctgctgccagtggcgataagtcgtgtcttaccgggttggactcaagacgatagttaccggataaggcgcagcggtcgggctgaacggggggttcgtgcacacagcccagcttggagcgaacgacctacaccgaactgagatacctacagcgtgagctatgagaaagcgccacgcttcccgaagggagaaaggcggacaggtatccggtaagcggcagggtcggaacaggagagcgcacgagggagcttccagggggaaacgcctggtatctttatagtcctgtcgggtttcgccacctctgacttgagcgtcgatttttgtgatgctcgtcaggggggcggagcctatggaaaaacgccagcaacgcggcctttttacggttcctggccttttgctggccttttgctcacatgttctttcctgcgttatcccctgattctgtggataaccgtattaccgctagccaggaagagtttgtagaaacgcaaaaaggccatccgtcaggatggccttctgcttagtttgatgcctggcagtttatggcgggcgtcctgcccgccaccctccgggccgttgcttcacaacgttcaaatccgctcccggcggatttgtcctactcaggagagcgttcaccgacaaacaacagataaaacgaaaggcccagtcttccgactgagcctttcgttttatttgatgcctggcagttccctactctcgcgttaacgctagcatggatctcgggccccaaataatgattttattttgactgatagtgacctgttcgttgcaacaaattgatgagcaatgcttttttataatgccaactttgtataataaagttggcttcgaaggagatagaac

pENTR-L3-SpCas9-HF2-2A-EGFP-L2

catggataaaaagtattctattggtttagacatcggcactaattccgttggatgggctgtcataaccgatgaatacaaagtaccttcaaagaaatttaaggtgttggggaacacagaccgtcattcgattaaaaagaatcttatcggtgccctcctattcgatagtggcgaaacggcagaggcgactcgcctgaaacgaaccgctcggagaaggtatacacgtcgcaagaaccgaatatgttacttacaagaaatttttagcaatgagatggccaaagttgacgattctttctttcaccgtttggaagagtccttccttgtcgaagaggacaagaaacatgaacggcaccccatctttggaaacatagtagatgaggtggcatatcatgaaaagtacccaacgatttatcacctcagaaaaaagctagttgactcaactgataaagcggacctgaggttaatctacttggctcttgcccatatgataaagttccgtgggcactttctcattgagggtgatctaaatccggacaactcggatgtcgacaaactgttcatccagttagtacaaacctataatcagttgtttgaagagaaccctataaatgcaagtggcgtggatgcgaaggctattcttagcgcccgcctctctaaatcccgacggctagaaaacctgatcgcacaattacccggagagaagaaaaatgggttgttcggtaaccttatagcgctctcactaggcctgacaccaaattttaagtcgaacttcgacttagctgaagatgccaaattgcagcttagtaaggacacgtacgatgacgatctcgacaatctactggcacaaattggagatcagtatgcggacttatttttggctgccaaaaaccttagcgatgcaatcctcctatctgacatactgagagttaatactgagattaccaaggcgccgttatccgcttcaatgatcaaaaggtacgatgaacatcaccaagacttgacacttctcaaggccctagtccgtcagcaactgcctgagaaatataaggaaatattctttgatcagtcgaaaaacgggtacgcaggttatattgacggcggagcgagtcaagaggaattctacaagtttatcaaacccatattagagaagatggatgggacggaagagttgcttgtaaaactcaatcgcgaagatctactgcgaaagcagcggactttcgacaacggtagcattccacatcaaatccacttaggcgaattgcatgctatacttagaaggcaggaggatttttatccgttcctcaaagacaatcgtgaaaagattgagaaaatcctaacctttcgcataccttactatgtgggacccctggcccgagggaactctcggttcgcatggatgacaagaaagtccgaagaaacgattactccctggaattttgaggaagttgtcgataaaggtgcgtcagctcaatcgttcatcgagaggatgaccgcctttgacaagaatttaccgaacgaaaaagtattgcctaagcacagtttactttacgagtatttcacagtgtacaatgaactcacgaaagttaagtatgtcactgagggcatgcgtaaacccgcctttctaagcggagaacagaagaaagcaatagtagatctgttattcaagaccaaccgcaaagtgacagttaagcaattgaaagaggactactttaagaaaattgaatgcttcgattctgtcgagatctccggggtagaagatcgatttaatgcgtcacttggtacgtatcatgacctcctaaagataattaaagataaggacttcctggataacgaagagaatgaagatatcttagaagatatagtgttgactcttaccctctttgaagatcgggaaatgattgaggaaagactaaaaacatacgctcacctgttcgacgataaggttatgaaacagttaaagaggcgtcgctatacgggctggggagccttgtcgcggaaacttatcaacgggataagagacaagcaaagtggtaaaactattctcgattttctaaagagcgacggcttcgccaataggaactttatggccctgatccatgatgactctttaaccttcaaagaggatatacaaaaggcacaggtttccggacaaggggactcattgcacgaacatattgcgaatcttgctggttcgccagccatcaaaaagggcatactccagacagtcaaagtagtggatgagctagttaaggtcatgggacgtcacaaaccggaaaacattgtaatcgagatggcacgcgaaaatcaaacgactcagaaggggcaaaaaaacagtcgagagcggatgaagagaatagaagagggtattaaagaactgggcagccagatcttaaaggagcatcctgtggaaaatacccaattgcagaacgagaaactttacctctattacctacaaaatggaagggacatgtatgttgatcaggaactggacataaaccgtttatctgattacgacgtcgatcacattgtaccccaatcctttttgaaggacgattcaatcgacaataaagtgcttacacgctcggataagaaccgagggaaaagtgacaatgttccaagcgaggaagtcgtaaagaaaatgaagaactattggcggcagctcctaaatgcgaaactgataacgcaaagaaagttcgataacttaactaaagctgagaggggtggcttgtctgaacttgacaaggccggatttattaaacgtcagctcgtggaaacccgcgccatcacaaagcatgttgcccagatactagattcccgaatgaatacgaaatacgacgagaacgataagctgattcgggaagtcaaagtaatcactttaaagtcaaaattggtgtcggacttcagaaaggattttcaattctataaagttagggagataaataactaccaccatgcgcacgacgcttatcttaatgccgtcgtagggaccgcactcattaagaaatacccgaagctagaaagtgagtttgtgtatggtgattacaaagtttatgacgtccgtaagatgatcgcgaaaagcgaacaggagataggcaaggctacagccaaatacttcttttattctaacattatgaatttctttaagacggaaatcactctggcaaacggagagatacgcaaacgacctttaattgaaaccaatggggagacaggtgaaatcgtatgggataagggccgggacttcgcgacggtgagaaaagttttgtccatgccccaagtcaacatagtaaagaaaactgaggtgcagaccggagggttttcaaaggaatcgattcttccaaaaaggaatagtgataagctcatcgctcgtaaaaaggactgggacccgaaaaagtacggtggcttcgagagccctacagttgcctattctgtcctagtagtggcaaaagttgagaagggaaaatccaagaaactgaagtcagtcaaagaattattggggataacgattatggagcgctcgtcttttgaaaagaaccccatcgacttccttgaggcgaaaggttacaaggaagtaaaaaaggatctcataattaaactaccaaagtatagtctgtttgagttagaaaatggccgaaaacggatgttggctagcgccggagagcttcaaaaggggaacgaactcgcactaccgtctaaatacgtgaatttcctgtatttagcgtcccattacgagaagttgaaaggttcacctgaagataacgaacagaagcaactttttgttgagcagcacaaacattatctcgacgaaatcatagagcaaatttcggaattcagtaagagagtcatcctagctgatgccaatctggacaaagtattaagcgcatacaacaagcacagggataaacccatacgtgagcaggcggaaaatattatccatttgtttactcttaccaacctcggcgctccagccgcattcaagtattttgacacaacgatagatcgcaaacgatacacttctaccaaggaggtgctagacgcgacactgattcaccaatccatcacgggattatatgaaactcggatagatttgtcacagcttgggggtgacggatcccccaagaagaagaggaaagtctcgagtagccgcgctaagcgggcaccggtgaaacagactttgaattttgaccttctcaagttggcgggagacgtggagtccaacccagggccggatatcatggtgagcaagggcgaggagctgttcaccggggtggtgcccatcctggtcgagctggacggcgacgtaaacggccacaagttcagcgtgtccggcgagggcgagggcgatgccacctacggcaagctgaccctgaagttcatctgcaccaccggcaagctgcccgtgccctggcccaccctcgtgaccaccctgacctacggcgtgcagtgcttcagccgctaccccgaccacatgaagcagcacgacttcttcaagtccgccatgcccgaaggctacgtccaggagcgcaccatcttcttcaaggacgacggcaactacaagacccgcgccgaggtgaagttcgagggcgacaccctggtgaaccgcatcgagctgaagggcatcgacttcaaggaggacggcaacatcctggggcacaagctggagtacaactacaacagccacaacgtctatatcatggccgacaagcagaagaacggcatcaaggtgaacttcaagatccgccacaacatcgaggacggcagcgtgcagctcgccgaccactaccagcagaacacccccatcggcgacggccccgtgctgctgcccgacaaccactacctgagcacccagtccgccctgagcaaagaccccaacgagaagcgcgatcacatggtcctgctggagttcgtgaccgccgccgggatcactctcggcatggacgagctgtacaagtaacagctttcttgtacaaagttggcattataagaaagcattgcttatcaatttgttgcaacgaacaggtcactatcagtcaaaataaaatcattatttgccatccagctgcagctctggcccgtgtctcaaaatctctgatgttacattgcacaagataaaaatatatcatcatgaacaataaaactgtctgcttacataaacagtaatacaaggggtgttatgagccatattcaacgggaaacgtcgaggccgcgattaaattccaacatggatgctgatttatatgggtataaatgggctcgcgataatgtcgggcaatcaggtgcgacaatctatcgcttgtatgggaagcccgatgcgccagagttgtttctgaaacatggcaaaggtagcgttgccaatgatgttacagatgagatggtcagactaaactggctgacggaatttatgcctcttccgaccatcaagcattttatccgtactcctgatgatgcatggttactcaccactgcgatccccggaaaaacagcattccaggtattagaagaatatcctgattcaggtgaaaatattgttgatgcgctggcagtgttcctgcgccggttgcattcgattcctgtttgtaattgtccttttaacagcgatcgcgtatttcgtctcgctcaggcgcaatcacgaatgaataacggtttggttgatgcgagtgattttgatgacgagcgtaatggctggcctgttgaacaagtctggaaagaaatgcataaacttttgccattctcaccggattcagtcgtcactcatggtgatttctcacttgataaccttatttttgacgaggggaaattaataggttgtattgatgttggacgagtcggaatcgcagaccgataccaggatcttgccatcctatggaactgcctcggtgagttttctccttcattacagaaacggctttttcaaaaatatggtattgataatcctgatatgaataaattgcagtttcatttgatgctcgatgagtttttctaatcagaattggttaattggttgtaacactggcagagcattacgctgacttgacgggacggcgcaagctcatgaccaaaatcccttaacgtgagttttcgttccactgagcgtcagaccccgtagaaaagatcaaaggatcttcttgagatcctttttttctgcgcgtaatctgctgcttgcaaacaaaaaaaccaccgctaccagcggtggtttgtttgccggatcaagagctaccaactctttttccgaaggtaactggcttcagcagagcgcagataccaaatactgtccttctagtgtagccgtagttaggccaccacttcaagaactctgtagcaccgcctacatacctcgctctgctaatcctgttaccagtggctgctgccagtggcgataagtcgtgtcttaccgggttggactcaagacgatagttaccggataaggcgcagcggtcgggctgaacggggggttcgtgcacacagcccagcttggagcgaacgacctacaccgaactgagatacctacagcgtgagctatgagaaagcgccacgcttcccgaagggagaaaggcggacaggtatccggtaagcggcagggtcggaacaggagagcgcacgagggagcttccagggggaaacgcctggtatctttatagtcctgtcgggtttcgccacctctgacttgagcgtcgatttttgtgatgctcgtcaggggggcggagcctatggaaaaacgccagcaacgcggcctttttacggttcctggccttttgctggccttttgctcacatgttctttcctgcgttatcccctgattctgtggataaccgtattaccgctagccaggaagagtttgtagaaacgcaaaaaggccatccgtcaggatggccttctgcttagtttgatgcctggcagtttatggcgggcgtcctgcccgccaccctccgggccgttgcttcacaacgttcaaatccgctcccggcggatttgtcctactcaggagagcgttcaccgacaaacaacagataaaacgaaaggcccagtcttccgactgagcctttcgttttatttgatgcctggcagttccctactctcgcgttaacgctagcatggatctcgggccccaaataatgattttattttgactgatagtgacctgttcgttgcaacaaattgatgagcaatgcttttttataatgccaactttgtataataaagttggcttcgaaggagatagaac

pENTR-L3-eSpCas9(1.1)-2A-EGFP-L2

gatcttcagcaacgagatggccaaggtggacgacagcttcttccacagactggaagagtccttcctggtggaagaggataagaagcacgagcggcaccccatcttcggcaacatcgtggacgaggtggcctaccacgagaagtaccccaccatctaccacctgagaaagaaactggtggacagcaccgacaaggccgacctgcggctgatctatctggccctggcccacatgatcaagttccggggccacttcctgatcgagggcgacctgaaccccgacaacagcgacgtggacaagctgttcatccagctggtgcagacctacaaccagctgttcgaggaaaaccccatcaacgccagcggcgtggacgccaaggccatcctgtctgccagactgagcaagagcagacggctggaaaatctgatcgcccagctgcccggcgagaagaagaatggcctgttcggaaacctgattgccctgagcctgggcctgacccccaacttcaagagcaacttcgacctggccgaggatgccaaactgcagctgagcaaggacacctacgacgacgacctggacaacctgctggcccagatcggcgaccagtacgccgacctgtttctggccgccaagaacctgtccgacgccatcctgctgagcgacatcctgagagtgaacaccgagatcaccaaggcccccctgagcgcctctatgatcaagagatacgacgagcaccaccaggacctgaccctgctgaaagctctcgtgcggcagcagctgcctgagaagtacaaagagattttcttcgaccagagcaagaacggctacgccggctacattgacggcggagccagccaggaagagttctacaagttcatcaagcccatcctggaaaagatggacggcaccgaggaactgctcgtgaagctgaacagagaggacctgctgcggaagcagcggaccttcgacaacggcagcatcccccaccagatccacctgggagagctgcacgccattctgcggcggcaggaagatttttacccattcctgaaggacaaccgggaaaagatcgagaagatcctgaccttccgcatcccctactacgtgggccctctggccaggggaaacagcagattcgcctggatgaccagaaagagcgaggaaaccatcaccccctggaacttcgaggaagtggtggacaagggcgcttccgcccagagcttcatcgagcggatgaccaacttcgataagaacctgcccaacgagaaggtgctgcccaagcacagcctgctgtacgagtacttcaccgtgtataacgagctgaccaaagtgaaatacgtgaccgagggaatgagaaagcccgccttcctgagcggcgagcagaaaaaggccatcgtggacctgctgttcaagaccaaccggaaagtgaccgtgaagcagctgaaagaggactacttcaagaaaatcgagtgcttcgactccgtggaaatctccggcgtggaagatcggttcaacgcctccctgggcacataccacgatctgctgaaaattatcaaggacaaggacttcctggacaatgaggaaaacgaggacattctggaagatatcgtgctgaccctgacactgtttgaggacagagagatgatcgaggaacggctgaaaacctatgcccacctgttcgacgacaaagtgatgaagcagctgaagcggcggagatacaccggctggggcaggctgagccggaagctgatcaacggcatccgggacaagcagtccggcaagacaatcctggatttcctgaagtccgacggcttcgccaacagaaacttcatgcagctgatccacgacgacagcctgacctttaaagaggacatccagaaagcccaggtgtccggccagggcgatagcctgcacgagcacattgccaatctggccggcagccccgccattaagaagggcatcctgcagacagtgaaggtggtggacgagctcgtgaaagtgatgggccggcacaagcccgagaacatcgtgatcgaaatggccagagagaaccagaccacccagaagggacagaagaacagccgcgagagaatgaagcggatcgaagagggcatcaaagagctgggcagccagatcctgaaagaacaccccgtggaaaacacccagctgcagaacgagaagctgtacctgtactacctgcagaatgggcgggatatgtacgtggaccaggaactggacatcaaccggctgtccgactacgatgtggaccatatcgtgcctcagagctttctggcggacgactccatcgacaacaaggtgctgaccagaagcgacaagaaccggggcaagagcgacaacgtgccctccgaagaggtcgtgaagaagatgaagaactactggcggcagctgctgaacgccaagctgattacccagagaaagttcgacaatctgaccaaggccgagagaggcggcctgagcgaactggataaggccggcttcatcaagagacagctggtggaaacccggcagatcacaaagcacgtggcacagatcctggactcccggatgaacactaagtacgacgagaatgacaagctgatccgggaagtgaaagtgatcaccctgaagtccaagctggtgtccgatttccggaaggatttccagttttacaaagtgcgcgagatcaacaactaccaccacgcccacgacgcctacctgaacgccgtcgtgggaaccgccctgatcaaaaagtaccctgcgctggaaagcgagttcgtgtacggcgactacaaggtgtacgacgtgcggaagatgatcgccaagagcgagcaggaaatcggcaaggctaccgccaagtacttcttctacagcaacatcatgaactttttcaagaccgagattaccctggccaacggcgagatccggaaggcgcctctgatcgagacaaacggcgaaaccggggagatcgtgtgggataagggccgggattttgccaccgtgcggaaagtgctgagcatgccccaagtgaatatcgtgaaaaagaccgaggtgcagacaggcggcttcagcaaagagtctatcctgcccaagaggaacagcgataagctgatcgccagaaagaaggactgggaccctaagaagtacggcggcttcgacagccccaccgtggcctattctgtgctggtggtggccaaagtggaaaagggcaagtccaagaaactgaagagtgtgaaagagctgctggggatcaccatcatggaaagaagcagcttcgagaagaatcccatcgactttctggaagccaagggctacaaagaagtgaaaaaggacctgatcatcaagctgcctaagtactccctgttcgagctggaaaacggccggaagagaatgctggcctctgccggcgaactgcagaagggaaacgaactggccctgccctccaaatatgtgaacttcctgtacctggccagccactatgagaagctgaagggctcccccgaggataatgagcagaaacagctgtttgtggaacagcacaagcactacctggacgagatcatcgagcagatcagcgagttctccaagagagtgatcctggccgacgctaatctggacaaagtgctgtccgcctacaacaagcaccgggataagcccatcagagagcaggccgagaatatcatccacctgtttaccctgaccaatctgggagcccctgccgccttcaagtactttgacaccaccatcgaccggaagaggtacaccagcaccaaagaggtgctggacgccaccctgatccaccagagcatcaccggcctgtacgagacacggatcgacctgtctcagctgggaggcgacaaaaggccggcggccacgaaaaaggccggccaggcaaaaaagaaaaaggaattcggcagtagccgggcaccggtgaaacagactttgaattttgaccttctcaagttggcgggagacgtggagtccaacccagggccggatatcatggtgagcaagggcgaggagctgttcaccggggtggtgcccatcctggtcgagctggacggcgacgtaaacggccacaagttcagcgtgtccggcgagggcgagggcgatgccacctacggcaagctgaccctgaagttcatctgcaccaccggcaagctgcccgtgccctggcccaccctcgtgaccaccctgacctacggcgtgcagtgcttcagccgctaccccgaccacatgaagcagcacgacttcttcaagtccgccatgcccgaaggctacgtccaggagcgcaccatcttcttcaaggacgacggcaactacaagacccgcgccgaggtgaagttcgagggcgacaccctggtgaaccgcatcgagctgaagggcatcgacttcaaggaggacggcaacatcctggggcacaagctggagtacaactacaacagccacaacgtctatatcatggccgacaagcagaagaacggcatcaaggtgaacttcaagatccgccacaacatcgaggacggcagcgtgcagctcgccgaccactaccagcagaacacccccatcggcgacggccccgtgctgctgcccgacaaccactacctgagcacccagtccgccctgagcaaagaccccaacgagaagcgcgatcacatggtcctgctggagttcgtgaccgccgccgggatcactctcggcatggacgagctgtacaagtaacagctttcttgtacaaagttggcattataagaaagcattgcttatcaatttgttgcaacgaacaggtcactatcagtcaaaataaaatcattatttgccatccagctgcagctctggcccgtgtctcaaaatctctgatgttacattgcacaagataaaaatatatcatcatgaacaataaaactgtctgcttacataaacagtaatacaaggggtgttatgagccatattcaacgggaaacgtcgaggccgcgattaaattccaacatggatgctgatttatatgggtataaatgggctcgcgataatgtcgggcaatcaggtgcgacaatctatcgcttgtatgggaagcccgatgcgccagagttgtttctgaaacatggcaaaggtagcgttgccaatgatgttacagatgagatggtcagactaaactggctgacggaatttatgcctcttccgaccatcaagcattttatccgtactcctgatgatgcatggttactcaccactgcgatccccggaaaaacagcattccaggtattagaagaatatcctgattcaggtgaaaatattgttgatgcgctggcagtgttcctgcgccggttgcattcgattcctgtttgtaattgtccttttaacagcgatcgcgtatttcgtctcgctcaggcgcaatcacgaatgaataacggtttggttgatgcgagtgattttgatgacgagcgtaatggctggcctgttgaacaagtctggaaagaaatgcataaacttttgccattctcaccggattcagtcgtcactcatggtgatttctcacttgataaccttatttttgacgaggggaaattaataggttgtattgatgttggacgagtcggaatcgcagaccgataccaggatcttgccatcctatggaactgcctcggtgagttttctccttcattacagaaacggctttttcaaaaatatggtattgataatcctgatatgaataaattgcagtttcatttgatgctcgatgagtttttctaatcagaattggttaattggttgtaacactggcagagcattacgctgacttgacgggacggcgcaagctcatgaccaaaatcccttaacgtgagttttcgttccactgagcgtcagaccccgtagaaaagatcaaaggatcttcttgagatcctttttttctgcgcgtaatctgctgcttgcaaacaaaaaaaccaccgctaccagcggtggtttgtttgccggatcaagagctaccaactctttttccgaaggtaactggcttcagcagagcgcagataccaaatactgtccttctagtgtagccgtagttaggccaccacttcaagaactctgtagcaccgcctacatacctcgctctgctaatcctgttaccagtggctgctgccagtggcgataagtcgtgtcttaccgggttggactcaagacgatagttaccggataaggcgcagcggtcgggctgaacggggggttcgtgcacacagcccagcttggagcgaacgacctacaccgaactgagatacctacagcgtgagctatgagaaagcgccacgcttcccgaagggagaaaggcggacaggtatccggtaagcggcagggtcggaacaggagagcgcacgagggagcttccagggggaaacgcctggtatctttatagtcctgtcgggtttcgccacctctgacttgagcgtcgatttttgtgatgctcgtcaggggggcggagcctatggaaaaacgccagcaacgcggcctttttacggttcctggccttttgctggccttttgctcacatgttctttcctgcgttatcccctgattctgtggataaccgtattaccgctagccaggaagagtttgtagaaacgcaaaaaggccatccgtcaggatggccttctgcttagtttgatgcctggcagtttatggcgggcgtcctgcccgccaccctccgggccgttgcttcacaacgttcaaatccgctcccggcggatttgtcctactcaggagagcgttcaccgacaaacaacagataaaacgaaaggcccagtcttccgactgagcctttcgttttatttgatgcctggcagttccctactctcgcgttaacgctagcatggatctcgggccccaaataatgattttattttgactgatagtgacctgttcgttgcaacaaattgatgagcaatgcttttttataatgccaactttgtataataaagttggcttcgaaggagatagaaccatggactataaggaccacgacggagactacaaggatcatgatattgattacaaagacgatgacgataagatggccccaaagaagaagcggaaggtcggtatccacggagtcccagcagccgacaagaagtacagcatcggcctggacatcggcaccaactctgtgggctgggccgtgatcaccgacgagtacaaggtgcccagcaagaaattcaaggtgctgggcaacaccgaccggcacagcatcaagaagaacctgatcggagccctgctgttcgacagcggcgaaacagccgaggccacccggctgaagagaaccgccagaagaagatacaccagacggaagaaccggatctgctatctgcaaga

pENTR-L3-eSpCas9n(1.1)-2A-EGFP-L2

gatcttcagcaacgagatggccaaggtggacgacagcttcttccacagactggaagagtccttcctggtggaagaggataagaagcacgagcggcaccccatcttcggcaacatcgtggacgaggtggcctaccacgagaagtaccccaccatctaccacctgagaaagaaactggtggacagcaccgacaaggccgacctgcggctgatctatctggccctggcccacatgatcaagttccggggccacttcctgatcgagggcgacctgaaccccgacaacagcgacgtggacaagctgttcatccagctggtgcagacctacaaccagctgttcgaggaaaaccccatcaacgccagcggcgtggacgccaaggccatcctgtctgccagactgagcaagagcagacggctggaaaatctgatcgcccagctgcccggcgagaagaagaatggcctgttcggaaacctgattgccctgagcctgggcctgacccccaacttcaagagcaacttcgacctggccgaggatgccaaactgcagctgagcaaggacacctacgacgacgacctggacaacctgctggcccagatcggcgaccagtacgccgacctgtttctggccgccaagaacctgtccgacgccatcctgctgagcgacatcctgagagtgaacaccgagatcaccaaggcccccctgagcgcctctatgatcaagagatacgacgagcaccaccaggacctgaccctgctgaaagctctcgtgcggcagcagctgcctgagaagtacaaagagattttcttcgaccagagcaagaacggctacgccggctacattgacggcggagccagccaggaagagttctacaagttcatcaagcccatcctggaaaagatggacggcaccgaggaactgctcgtgaagctgaacagagaggacctgctgcggaagcagcggaccttcgacaacggcagcatcccccaccagatccacctgggagagctgcacgccattctgcggcggcaggaagatttttacccattcctgaaggacaaccgggaaaagatcgagaagatcctgaccttccgcatcccctactacgtgggccctctggccaggggaaacagcagattcgcctggatgaccagaaagagcgaggaaaccatcaccccctggaacttcgaggaagtggtggacaagggcgcttccgcccagagcttcatcgagcggatgaccaacttcgataagaacctgcccaacgagaaggtgctgcccaagcacagcctgctgtacgagtacttcaccgtgtataacgagctgaccaaagtgaaatacgtgaccgagggaatgagaaagcccgccttcctgagcggcgagcagaaaaaggccatcgtggacctgctgttcaagaccaaccggaaagtgaccgtgaagcagctgaaagaggactacttcaagaaaatcgagtgcttcgactccgtggaaatctccggcgtggaagatcggttcaacgcctccctgggcacataccacgatctgctgaaaattatcaaggacaaggacttcctggacaatgaggaaaacgaggacattctggaagatatcgtgctgaccctgacactgtttgaggacagagagatgatcgaggaacggctgaaaacctatgcccacctgttcgacgacaaagtgatgaagcagctgaagcggcggagatacaccggctggggcaggctgagccggaagctgatcaacggcatccgggacaagcagtccggcaagacaatcctggatttcctgaagtccgacggcttcgccaacagaaacttcatgcagctgatccacgacgacagcctgacctttaaagaggacatccagaaagcccaggtgtccggccagggcgatagcctgcacgagcacattgccaatctggccggcagccccgccattaagaagggcatcctgcagacagtgaaggtggtggacgagctcgtgaaagtgatgggccggcacaagcccgagaacatcgtgatcgaaatggccagagagaaccagaccacccagaagggacagaagaacagccgcgagagaatgaagcggatcgaagagggcatcaaagagctgggcagccagatcctgaaagaacaccccgtggaaaacacccagctgcagaacgagaagctgtacctgtactacctgcagaatgggcgggatatgtacgtggaccaggaactggacatcaaccggctgtccgactacgatgtggaccatatcgtgcctcagagctttctggcggacgactccatcgacaacaaggtgctgaccagaagcgacaagaaccggggcaagagcgacaacgtgccctccgaagaggtcgtgaagaagatgaagaactactggcggcagctgctgaacgccaagctgattacccagagaaagttcgacaatctgaccaaggccgagagaggcggcctgagcgaactggataaggccggcttcatcaagagacagctggtggaaacccggcagatcacaaagcacgtggcacagatcctggactcccggatgaacactaagtacgacgagaatgacaagctgatccgggaagtgaaagtgatcaccctgaagtccaagctggtgtccgatttccggaaggatttccagttttacaaagtgcgcgagatcaacaactaccaccacgcccacgacgcctacctgaacgccgtcgtgggaaccgccctgatcaaaaagtaccctgcgctggaaagcgagttcgtgtacggcgactacaaggtgtacgacgtgcggaagatgatcgccaagagcgagcaggaaatcggcaaggctaccgccaagtacttcttctacagcaacatcatgaactttttcaagaccgagattaccctggccaacggcgagatccggaaggcgcctctgatcgagacaaacggcgaaaccggggagatcgtgtgggataagggccgggattttgccaccgtgcggaaagtgctgagcatgccccaagtgaatatcgtgaaaaagaccgaggtgcagacaggcggcttcagcaaagagtctatcctgcccaagaggaacagcgataagctgatcgccagaaagaaggactgggaccctaagaagtacggcggcttcgacagccccaccgtggcctattctgtgctggtggtggccaaagtggaaaagggcaagtccaagaaactgaagagtgtgaaagagctgctggggatcaccatcatggaaagaagcagcttcgagaagaatcccatcgactttctggaagccaagggctacaaagaagtgaaaaaggacctgatcatcaagctgcctaagtactccctgttcgagctggaaaacggccggaagagaatgctggcctctgccggcgaactgcagaagggaaacgaactggccctgccctccaaatatgtgaacttcctgtacctggccagccactatgagaagctgaagggctcccccgaggataatgagcagaaacagctgtttgtggaacagcacaagcactacctggacgagatcatcgagcagatcagcgagttctccaagagagtgatcctggccgacgctaatctggacaaagtgctgtccgcctacaacaagcaccgggataagcccatcagagagcaggccgagaatatcatccacctgtttaccctgaccaatctgggagcccctgccgccttcaagtactttgacaccaccatcgaccggaagaggtacaccagcaccaaagaggtgctggacgccaccctgatccaccagagcatcaccggcctgtacgagacacggatcgacctgtctcagctgggaggcgacaaaaggccggcggccacgaaaaaggccggccaggcaaaaaagaaaaaggaattcggcagtagccgggcaccggtgaaacagactttgaattttgaccttctcaagttggcgggagacgtggagtccaacccagggccggatatcatggtgagcaagggcgaggagctgttcaccggggtggtgcccatcctggtcgagctggacggcgacgtaaacggccacaagttcagcgtgtccggcgagggcgagggcgatgccacctacggcaagctgaccctgaagttcatctgcaccaccggcaagctgcccgtgccctggcccaccctcgtgaccaccctgacctacggcgtgcagtgcttcagccgctaccccgaccacatgaagcagcacgacttcttcaagtccgccatgcccgaaggctacgtccaggagcgcaccatcttcttcaaggacgacggcaactacaagacccgcgccgaggtgaagttcgagggcgacaccctggtgaaccgcatcgagctgaagggcatcgacttcaaggaggacggcaacatcctggggcacaagctggagtacaactacaacagccacaacgtctatatcatggccgacaagcagaagaacggcatcaaggtgaacttcaagatccgccacaacatcgaggacggcagcgtgcagctcgccgaccactaccagcagaacacccccatcggcgacggccccgtgctgctgcccgacaaccactacctgagcacccagtccgccctgagcaaagaccccaacgagaagcgcgatcacatggtcctgctggagttcgtgaccgccgccgggatcactctcggcatggacgagctgtacaagtaacagctttcttgtacaaagttggcattataagaaagcattgcttatcaatttgttgcaacgaacaggtcactatcagtcaaaataaaatcattatttgccatccagctgcagctctggcccgtgtctcaaaatctctgatgttacattgcacaagataaaaatatatcatcatgaacaataaaactgtctgcttacataaacagtaatacaaggggtgttatgagccatattcaacgggaaacgtcgaggccgcgattaaattccaacatggatgctgatttatatgggtataaatgggctcgcgataatgtcgggcaatcaggtgcgacaatctatcgcttgtatgggaagcccgatgcgccagagttgtttctgaaacatggcaaaggtagcgttgccaatgatgttacagatgagatggtcagactaaactggctgacggaatttatgcctcttccgaccatcaagcattttatccgtactcctgatgatgcatggttactcaccactgcgatccccggaaaaacagcattccaggtattagaagaatatcctgattcaggtgaaaatattgttgatgcgctggcagtgttcctgcgccggttgcattcgattcctgtttgtaattgtccttttaacagcgatcgcgtatttcgtctcgctcaggcgcaatcacgaatgaataacggtttggttgatgcgagtgattttgatgacgagcgtaatggctggcctgttgaacaagtctggaaagaaatgcataaacttttgccattctcaccggattcagtcgtcactcatggtgatttctcacttgataaccttatttttgacgaggggaaattaataggttgtattgatgttggacgagtcggaatcgcagaccgataccaggatcttgccatcctatggaactgcctcggtgagttttctccttcattacagaaacggctttttcaaaaatatggtattgataatcctgatatgaataaattgcagtttcatttgatgctcgatgagtttttctaatcagaattggttaattggttgtaacactggcagagcattacgctgacttgacgggacggcgcaagctcatgaccaaaatcccttaacgtgagttttcgttccactgagcgtcagaccccgtagaaaagatcaaaggatcttcttgagatcctttttttctgcgcgtaatctgctgcttgcaaacaaaaaaaccaccgctaccagcggtggtttgtttgccggatcaagagctaccaactctttttccgaaggtaactggcttcagcagagcgcagataccaaatactgtccttctagtgtagccgtagttaggccaccacttcaagaactctgtagcaccgcctacatacctcgctctgctaatcctgttaccagtggctgctgccagtggcgataagtcgtgtcttaccgggttggactcaagacgatagttaccggataaggcgcagcggtcgggctgaacggggggttcgtgcacacagcccagcttggagcgaacgacctacaccgaactgagatacctacagcgtgagctatgagaaagcgccacgcttcccgaagggagaaaggcggacaggtatccggtaagcggcagggtcggaacaggagagcgcacgagggagcttccagggggaaacgcctggtatctttatagtcctgtcgggtttcgccacctctgacttgagcgtcgatttttgtgatgctcgtcaggggggcggagcctatggaaaaacgccagcaacgcggcctttttacggttcctggccttttgctggccttttgctcacatgttctttcctgcgttatcccctgattctgtggataaccgtattaccgctagccaggaagagtttgtagaaacgcaaaaaggccatccgtcaggatggccttctgcttagtttgatgcctggcagtttatggcgggcgtcctgcccgccaccctccgggccgttgcttcacaacgttcaaatccgctcccggcggatttgtcctactcaggagagcgttcaccgacaaacaacagataaaacgaaaggcccagtcttccgactgagcctttcgttttatttgatgcctggcagttccctactctcgcgttaacgctagcatggatctcgggccccaaataatgattttattttgactgatagtgacctgttcgttgcaacaaattgatgagcaatgcttttttataatgccaactttgtataataaagttggcttcgaaggagatagaaccatggactataaggaccacgacggagactacaaggatcatgatattgattacaaagacgatgacgataagatggccccaaagaagaagcggaaggtcggtatccacggagtcccagcagccgacaagaagtacagcatcggcctggccatcggcaccaactctgtgggctgggccgtgatcaccgacgagtacaaggtgcccagcaagaaattcaaggtgctgggcaacaccgaccggcacagcatcaagaagaacctgatcggagccctgctgttcgacagcggcgaaacagccgaggccacccggctgaagagaaccgccagaagaagatacaccagacggaagaaccggatctgctatctgcaaga

CSIV-DEST

ctttaagaccaatgacttacaaggcagctgtagatcttagccactttttaaaagaaaaggggggactggaagggctaattcactcccaacgaagacaagatatcataacttcgtatagcatacattatacgaagttattccggactgtactgggtctctctggttagaccagatctgagcctgggagctctctggctaactagggaacccactgcttaagcctcaataaagcttgccttgagtgcttcaagtagtgtgtgcccgtctgttgtgtgactctggtaactagagatccctcagacccttttagtcagtgtggaaaatctctagcagggcccgtttaaacccgctgatcagcctcgactgtgccttctagttgccagccatctgttgtttgcccctcccccgtgccttccttgaccctggaaggtgccactcccactgtcctttcctaataaaatgaggaaattgcatcgcattgtctgagtaggtgtcattctattctggggggtggggtggggcaggacagcaagggggaggattgggaagacaatagcaggcatgctggggatgcggtgggctctatggcttctgaggcggaaagaaccagctggggctctagggggtatccccacgcgccctgtagcggcgcattaagcgcggcgggtgtggtggttacgcgcagcgtgaccgctacacttgccagcgccctagcgcccgctcctttcgctttcttcccttcctttctcgccacgttcgccggctttccccgtcaagctctaaatcggggcatccctttagggttccgatttagtgctttacggcacctcgaccccaaaaaacttgattagggtgatggttcacgtagtgggccatcgccctgatagacggtttttcgccctttgacgttggagtccacgttctttaatagtggactcttgttccaaactggaacaacactcaaccctatctcggtctattcttttgatttataagggattttggggatttcggcctattggttaaaaaatgagctgatttaacaaaaatttaacgcgaattaattctgtggaatgtgtgtcagttagggtgtggaaagtccccaggctccccaggcaggcagaagtatgcaaagcatgcatctcaattagtcagcaaccaggtgtggaaagtccccaggctccccagcaggcagaagtatgcaaagcatgcatctcaattagtcagcaaccatagtcccgcccctaactccgcccatcccgcccctaactccgcccagttccgcccattctccgccccatggctgactaattttttttatttatgcagaggccgaggccgcctctgcctctgagctattccagaagtagtgaggaggcttttttggaggcctaggcttttgcaaaaagctcccgggagcttgtatatccattttcggatctgatcagcacgtgttgacaattaatcatcggcatagtatatcggcatagtataatacgacaaggtgaggaactaaaccatggccaagttgaccagtgccgttccggtgctcaccgcgcgcgacgtcgccggagcggtcgagttctggaccgaccggctcgggttctcccgggacttcgtggaggacgacttcgccggtgtggtccgggacgacgtgaccctgttcatcagcgcggtccaggaccaggtggtgccggacaacaccctggcctgggtgtgggtgcgcggcctggacgagctgtacgccgagtggtcggaggtcgtgtccacgaacttccgggacgcctccgggccggccatgaccgagatcggcgagcagccgtgggggcgggagttcgccctgcgcgacccggccggcaactgcgtgcacttcgtggccgaggagcaggactgacacgtgctacgagatttcgattccaccgccgccttctatgaaaggttgggcttcggaatcgttttccgggacgccggctggatgatcctccagcgcggggatctcatgctggagttcttcgcccaccccaacttgtttattgcagcttataatggttacaaataaagcaatagcatcacaaatttcacaaataaagcatttttttcactgcattctagttgtggtttgtccaaactcatcaatgtatcttatcatgtctgtataccgtcgacctctagctagagcttggcgtaatcatggtcatagctgtttcctgtgtgaaattgttatccgctcacaattccacacaacatacgagccggaagcataaagtgtaaagcctggggtgcctaatgagtgagctaactcacattaattgcgttgcgctcactgcccgctttccagtcgggaaacctgtcgtgccagctgcattaatgaatcggccaacgcgcggggagaggcggtttgcgtattgggcgctcttccgcttcctcgctcactgactcgctgcgctcggtcgttcggctgcggcgagcggtatcagctcactcaaaggcggtaatacggttatccacagaatcaggggataacgcaggaaagaacatgtgagcaaaaggccagcaaaaggccaggaaccgtaaaaaggccgcgttgctggcgtttttccataggctccgcccccctgacgagcatcacaaaaatcgacgctcaagtcagaggtggcgaaacccgacaggactataaagataccaggcgtttccccctggaagctccctcgtgcgctctcctgttccgaccctgccgcttaccggatacctgtccgcctttctcccttcgggaagcgtggcgctttctcaatgctcacgctgtaggtatctcagttcggtgtaggtcgttcgctccaagctgggctgtgtgcacgaaccccccgttcagcccgaccgctgcgccttatccggtaactatcgtcttgagtccaacccggtaagacacgacttatcgccactggcagcagccactggtaacaggattagcagagcgaggtatgtaggcggtgctacagagttcttgaagtggtggcctaactacggctacactagaaggacagtatttggtatctgcgctctgctgaagccagttaccttcggaaaaagagttggtagctcttgatccggcaaacaaaccaccgctggtagcggtggtttttttgtttgcaagcagcagattacgcgcagaaaaaaaggatctcaagaagatcctttgatcttttctacggggtctgacgctcagtggaacgaaaactcacgttaagggattttggtcatgagattatcaaaaaggatcttcacctagatccttttaaattaaaaatgaagttttaaatcaatctaaagtatatatgagtaaacttggtctgacagttaccaatgcttaatcagtgaggcacctatctcagcgatctgtctatttcgttcatccatagttgcctgactccccgtcgtgtagataactacgatacgggagggcttaccatctggccccagtgctgcaatgataccgcgagacccacgctcaccggctccagatttatcagcaataaaccagccagccggaagggccgagcgcagaagtggtcctgcaactttatccgcctccatccagtctattaattgttgccgggaagctagagtaagtagttcgccagttaatagtttgcgcaacgttgttgccattgctacaggcatcgtggtgtcacgctcgtcgtttggtatggcttcattcagctccggttcccaacgatcaaggcgagttacatgatcccccatgttgtgcaaaaaagcggttagctccttcggtcctccgatcgttgtcagaagtaagttggccgcagtgttatcactcatggttatggcagcactgcataattctcttactgtcatgccatccgtaagatgcttttctgtgactggtgagtactcaaccaagtcattctgagaatagtgtatgcggcgaccgagttgctcttgcccggcgtcaatacgggataataccgcgccacatagcagaactttaaaagtgctcatcattggaaaacgttcttcggggcgaaaactctcaaggatcttaccgctgttgagatccagttcgatgtaacccactcgtgcacccaactgatcttcagcatcttttactttcaccagcgtttctgggtgagcaaaaacaggaaggcaaaatgccgcaaaaaagggaataagggcgacacggaaatgttgaatactcatactcttcctttttcaatattattgaagcatttatcagggttattgtctcatgagcggatacatatttgaatgtatttagaaaaataaacaaataggggttccgcgcacatttccccgaaaagtgccacctgacgtcgacggatcgggagatctcccgatcccctatggtcgactctcagtacaatctgctctgatgccgcatagttaagccagtatctgctccctgcttgtgtgttggaggtcgctgagtagtgcgcgagcaaaatttaagctacaacaaggcaaggcttgaccgacaattgcatgaagaatctgcttagggttaggcgttttgcgctgcttcgcgatgtacgggccagatatacgcgttgacattgattattgactagttattaatagtaatcaattacggggtcattagttcatagcccatatatggagttccgcgttacataacttacggtaaatggcccgcctggctgaccgcccaacgacccccgcccattgacgtcaataatgacgtatgttcccatagtaacgccaatagggactttccattgacgtcaatgggtggagtatttacggtaaactgcccacttggcagtacatcaagtgtatcatatgccaagtacgccccctattgacgtcaatgacggtaaatggcccgcctggcattatgcccagtacatgaccttatgggactttcctacttggcagtacatctacgtattagtcatcgctattaccatggtgatgcggttttggcagtacatcaatgggcgtggatagcggtttgactcacggggatttccaagtctccaccccattgacgtcaatgggagtttgttttggcaccaaaatcaacgggactttccaaaatgtcgtaacaactccgccccattgacgcaaatgggcggtaggcgtgtacggtgggaggtctatataagcagcgcgttttgcctgtactgggtctctctggttagaccagatctgagcctgggagctctctggctaactagggaacccactgcttaagcctcaataaagcttgccttgagtgcttcaagtagtgtgtgcccgtctgttgtgtgactctggtaactagagatccctcagacccttttagtcagtgtggaaaatctctagcagtggcgcccgaacagggacttgaaagcgaaagggaaaccagaggagctctctcgacgcaggactcggcttgctgaagcgcgcacggcaagaggcgaggggcggcgactggtgagtacgccaaaaattttgactagcggaggctagaaggagagagatgggtgcgagagcgtcagtattaagcgggggagaattagatcgcgatgggaaaaaattcggttaaggccagggggaaagaaaaaatataaattaaaacatatagtatgggcaagcagggagctagaacgattcgcagttaatcctggcctgttagaaacatcagaaggctgtagacaaatactgggacagctacaaccatcccttcagacaggatcagaagaacttagatcattatataatacagtagcaaccctctattgtgtgcatcaaaggatagagataaaagacaccaaggaagctttagacaagatagaggaagagcaaaacaaaagtaagaccaccgcacagcaagcggccgctgatcttcagacctggaggaggagatatgagggacaattggagaagtgaattatataaatataaagtagtaaaaattgaaccattaggagtagcacccaccaaggcaaagagaagagtggtgcagagagaaaaaagagcagtgggaataggagctttgttccttgggttcttgggagcagcaggaagcactatgggcgcagcgtcaatgacgctgacggtacaggccagacaattattgtctggtatagtgcagcagcagaacaatttgctgagggctattgaggcgcaacagcatctgttgcaactcacagtctggggcatcaagcagctccaggcaagaatcctggctgtggaaagatacctaaaggatcaacagctcctggggatttggggttgctctggaaaactcatttgcaccactgctgtgccttggaatgctagttggagtaataaatctctggaacagatttggaatcacacgacctggatggagtgggacagagaaattaacaattacacaagcttaatacactccttaattgaagaatcgcaaaaccagcaagaaaagaatgaacaagaattattggaattagataaatgggcaagtttgtggaattggtttaacataacaaattggctgtggtatataaaattattcataatgatagtaggaggcttggtaggtttaagaatagtttttgctgtactttctatagtgaatagagttaggcagggatattcaccattatcgtttcagacccacctcccaaccccgaggggacccgacaggcccgaaggaatagaagaagaaggtggagagagagacagagacagatccattcgattagtgaacggatctacaaatggcagtattcatccacaattttaaaagaaaaggggggattggggggtacagtgcaggggaaagaatagtagacataatagcaacagacatacaaactaaagaattacaaaaacaaattacaaaaattcaaaattttcgggtttattacagggacagcagaaattcactttgaattctagcatcacaagtttgtacaaaaaagctgaacgagaaacgtaaaatgatataaatatcaatatattaaattagattttgcataaaaaacagactacataatactgtaaaacacaacatatccagtcactatggcggccgcattaggcaccccaggctttacactttatgcttccggctcgtataatgtgtggattttgagttaggatccgtcgagattttcaggagctaaggaagctaaaatggagaaaaaaatcactggatataccaccgttgatatatcccaatggcatcgtaaagaacattttgaggcatttcagtcagttgctcaatgtacctataaccagaccgttcagctggatattacggcctttttaaagaccgtaaagaaaaataagcacaagttttatccggcctttattcacattcttgcccgcctgatgaatgctcatccggaattccgtatggcaatgaaagacggtgagctggtgatatgggatagtgttcacccttgttacaccgttttccatgagcaaactgaaacgttttcatcgctctggagtgaataccacgacgatttccggcagtttctacacatatattcgcaagatgtggcgtgttacggtgaaaacctggcctatttccctaaagggtttattgagaatatgtttttcgtctcagccaatccctgggtgagtttcaccagttttgatttaaacgtggccaatatggacaacttcttcgcccccgttttcaccatgggcaaatattatacgcaaggcgacaaggtgctgatgccgctggcgattcaggttcatcatgccgtttgtgatggcttccatgtcggcagaatgcttaatgaattacaacagtactgcgatgagtggcagggcggggcgtaaacgcgtggatccggcttactaaaagccagataacagtatgcgtatttgcgcgctgatttttgcggtataagaatatatactgatatgtatacccgaagtatgtcaaaaagaggtatgctatgaagcagcgtattacagtgacagttgacagcgacagctatcagttgctcaaggcatatatgatgtcaatatctccggtctggtaagcacaaccatgcagaatgaagcccgtcgtctgcgtgccgaacgctggaaagcggaaaatcaggaagggatggctgaggtcgcccggtttattgaaatgaacggctcttttgctgacgagaacaggggctggtgaaatgcagtttaaggtttacacctataaaagagagagccgttatcgtctgtttgtggatgtacagagtgatattattgacacgcccgggcgacggatggtgatccccctggccagtgcacgtctgctgtcagataaagtctcccgtgaactttacccggtggtgcatatcggggatgaaagctggcgcatgatgaccaccgatatggccagtgtgccggtctccgttatcggggaagaagtggctgatctcagccaccgcgaaaatgacatcaaaaacgccattaacctgatgttctggggaatataaatgtcaggctcccttatacacagccagtctgcaggtcgaccatagtgactggatatgttgtgttttacagtattatgtagtctgttttttatgcaaaatctaatttaatatattgatatttatatcattttacgtttctcgttcagctttcttgtacaaagtggtgatgctaccggtctcgaaacatcgagggatcaagcttatcgataatcaacctctggattacaaaatttgtgaaagattgactggtattcttaactatgttgctccttttacgctatgtggatacgctgctttaatgcctttgtatcatgctattgcttcccgtatggctttcattttctcctccttgtataaatcctggttgctgtctctttatgaggagttgtggcccgttgtcaggcaacgtggcgtggtgtgcactgtgtttgctgacgcaacccccactggttggggcattgccaccacctgtcagctcctttccgggactttcgctttccccctccctattgccacggcggaactcatcgccgcctgccttgcccgctgctggacaggggctcggctgttgggcactgacaattccgtggtgttgtcggggaagctgacgtcctttccatggctgctcgcctgtgttgccacctggattctgcgcgggacgtccttctgctacgtcccttcggccctcaatccagcggaccttccttcccgcggcctgctgccggctctgcggcctcttccgcgtcttcgccttcgccctcagacgagtcggatctccctttgggccgcctccccgcatcgataccgtcgagacctggaaaaacatggagcaatcacaagtagcaacacagcagctaccaatgctgcttgtgcctggctagaagcacaagaggaggaggaggtgggttttccagtcacacctcaggtac

pUC-DEST-R1R4

gacctgcaggcatgcaagcttggcactggccgtcgttttacaacgtcgtgactgggaaaaccctggcgttacccaacttaatcgccttgcagcacatccccctttcgccagctggcgtaatagcgaagaggcccgcaccgatcgcccttcccaacagttgcgcagcctgaatggcgaatggcgcctgatgcggtattttctccttacgcatctgtgcggtatttcacaccgcatatggtgcactctcagtacaatctgctctgatgccgcatagttaagccagccccgacacccgccaacacccgctgacgcgccctgacgggcttgtctgctcccggcatccgcttacagacaagctgtgaccgtctccgggagctgcatgtgtcagaggttttcaccgtcatcaccgaaacgcgcgagacgaaagggcctcgtgatacgcctatttttataggttaatgtcatgataataatggtttcttagacgtcaggtggcacttttcggggaaatgtgcgcggaacccctatttgtttatttttctaaatacattcaaatatgtatccgctcatgagacaataaccctgataaatgcttcaataatattgaaaaaggaagagtatgagtattcaacatttccgtgtcgcccttattcccttttttgcggcattttgccttcctgtttttgctcacccagaaacgctggtgaaagtaaaagatgctgaagatcagttgggtgcacgagtgggttacatcgaactggatctcaacagcggtaagatccttgagagttttcgccccgaagaacgttttccaatgatgagcacttttaaagttctgctatgtggcgcggtattatcccgtattgacgccgggcaagagcaactcggtcgccgcatacactattctcagaatgacttggttgagtactcaccagtcacagaaaagcatcttacggatggcatgacagtaagagaattatgcagtgctgccataaccatgagtgataacactgcggccaacttacttctgacaacgatcggaggaccgaaggagctaaccgcttttttgcacaacatgggggatcatgtaactcgccttgatcgttgggaaccggagctgaatgaagccataccaaacgacgagcgtgacaccacgatgcctgtagcaatggcaacaacgttgcgcaaactattaactggcgaactacttactctagcttcccggcaacaattaatagactggatggaggcggataaagttgcaggaccacttctgcgctcggcccttccggctggctggtttattgctgataaatctggagccggtgagcgtgggtctcgcggtatcattgcagcactggggccagatggtaagccctcccgtatcgtagttatctacacgacggggagtcaggcaactatggatgaacgaaatagacagatcgctgagataggtgcctcactgattaagcattggtaactgtcagaccaagtttactcatatatactttagattgatttaaaacttcatttttaatttaaaaggatctaggtgaagatcctttttgataatctcatgaccaaaatcccttaacgtgagttttcgttccactgagcgtcagaccccgtagaaaagatcaaaggatcttcttgagatcctttttttctgcgcgtaatctgctgcttgcaaacaaaaaaaccaccgctaccagcggtggtttgtttgccggatcaagagctaccaactctttttccgaaggtaactggcttcagcagagcgcagataccaaatactgtccttctagtgtagccgtagttaggccaccacttcaagaactctgtagcaccgcctacatacctcgctctgctaatcctgttaccagtggctgctgccagtggcgataagtcgtgtcttaccgggttggactcaagacgatagttaccggataaggcgcagcggtcgggctgaacggggggttcgtgcacacagcccagcttggagcgaacgacctacaccgaactgagatacctacagcgtgagctatgagaaagcgccacgcttcccgaagggagaaaggcggacaggtatccggtaagcggcagggtcggaacaggagagcgcacgagggagcttccagggggaaacgcctggtatctttatagtcctgtcgggtttcgccacctctgacttgagcgtcgatttttgtgatgctcgtcaggggggcggagcctatggaaaaacgccagcaacgcggcctttttacggttcctggccttttgctggccttttgctcacatgttctttcctgcgttatcccctgattctgtggataaccgtattaccgcctttgagtgagctgataccgctcgccgcagccgaacgaccgagcgcagcgagtcagtgagcgaggaagcggaagagcgcccaatacgcaaaccgcctctccccgcgcgttggccgattcattaatgcagctggcacgacaggtttcccgactggaaagcgggcagtgagcgcaacgcaattaatgtgagttagctcactcattaggcaccccaggctttacactttatgcttccggctcgtatgttgtgtggaattgtgagcggataacaatttcacacaggaaacagctatgaccatgattacgaattcgagctcggtacccggggatcctctagagtcggggcaagtttgtacaaaaaagctgaacgagaaacgtaaaatgatataaatatcaatatattaaattagattttgcataaaaaacagactacataatactgtaaaacacaacatatccagtcactatgaatcaactacttagatggtattagtgacctgtagtcgaccgacagccttccaaatgttcttcgggtgatgctgccaacttagtcgaccgacagccttccaaatgttcttctcaaacggaatcgtcgtatccagcctactcgctattgtcctcaatgccgtattaaatcataaaaagaaataagaaaaagaggtgcgagcctcttttttgtgtgacaaaataaaaacatctacctattcatatacgctagtgtcatagtcctgaaaatcatctgcatcaagaacaatttcacaactcttatacttttctcttacaagtcgttcggcttcatctggattttcagcctctatacttactaaacgtgataaagtttctgtaatttctactgtatcgacctgcagactggctgtgtataagggagcctgacatttatattccccagaacatcaggttaatggcgtttttgatgtcattttcgcggtggctgagatcagccacttcttccccgataacggagaccggcacactggccatatcggtggtcatcatgcgccagctttcatccccgatatgcaccaccgggtaaagttcacgggagactttatctgacagcagacgtgcactggccagggggatcaccatccgtcgcccgggcgtgtcaataatatcactctgtacatccacaaacagacgataacggctctctcttttataggtgtaaaccttaaactgcatttcaccagtccctgttctcgtcagcaaaagagccgttcatttcaataaaccgggcgacctcagccatcccttcctgattttccgctttccagcgttcggcacgcagacgacgggcttcattctgcatggttgtgcttaccagaccggagatattgacatcatatatgccttgagcaactgatagctgtcgctgtcaactgtcactgtaatacgctgcttcatagcacacctctttttgacatacttcgggtatacatatcagtatatattcttataccgcaaaaatcagcgcgcaaatacgcatactgttatctggcttttagtaagccggatccacgcgattacgccccgccctgccactcatcgcagtactgttgtaattcattaagcattctgccgacatggaagccatcacagacggcatgatgaacctgaatcgccagcggcatcagcaccttgtcgccttgcgtataatatttgcccatggtgaaaacgggggcgaagaagttgtccatattggccacgtttaaatcaaaactggtgaaactcacccagggattggctgagacgaaaaacatattctcaataaaccctttagggaaataggccaggttttcaccgtaacacgccacatcttgcgaatatatgtgtagaaactgccggaaatcgtcgtggtattcactccagagcgatgaaaacgtttcagtttgctcatggaaaacggtgtaacaagggtgaacactatcccatatcaccagctcaccgtctttcattgccatacggaattccggatgagcattcatcaggcgggcaagaatgtgaataaaggccggataaaacttgtgcttatttttctttacggtctttaaaaaggccgtaatatccagctgaacggtctggttataggtacattgagcaactgactgaaatgcctcaaaatgttctttacgatgccattgggatatatcaacggtggtatatccagtgatttttttctccattttagcttccttagctcctgaaaatctcgataactcaaaaaatacgcccggtagtgatcttatttcattatggtgaaagttggaacctcttacgtgccgatcaacgtctcattttcgccaaaagttggcccagggcttcccggtatcaacagggacaccaggatttatttattctgcgaagtgatcttccgtcacaggtatttattcggcgcaaagtgcgtcgggtgatgctgccaacttagtcgactacaggtcactaataccatctaagtagttgattcatagtgactggatatgttgtgttttacagtattatgtagtctgttttttatgcaaaatctaatttaatatattgatatttatatcattttacgtttctcgttcaacttttctatacaaagttgcccc

pUC-DEST-R5R4

ccggggatcctctagagtcggggcaactttgtatacaaaagttgaacgagaaacgtaaaatgatataaatatcaatatattaaattagattttgcataaaaaacagactacataatactgtaaaacacaacatatccagtcactatgaatcaactacttagatggtattagtgacctgtagtcgaccgacagccttccaaatgttcttcgggtgatgctgccaacttagtcgaccgacagccttccaaatgttcttctcaaacggaatcgtcgtatccagcctactcgctattgtcctcaatgccgtattaaatcataaaaagaaataagaaaaagaggtgcgagcctcttttttgtgtgacaaaataaaaacatctacctattcatatacgctagtgtcatagtcctgaaaatcatctgcatcaagaacaatttcacaactcttatacttttctcttacaagtcgttcggcttcatctggattttcagcctctatacttactaaacgtgataaagtttctgtaatttctactgtatcgacctgcagactggctgtgtataagggagcctgacatttatattccccagaacatcaggttaatggcgtttttgatgtcattttcgcggtggctgagatcagccacttcttccccgataacggagaccggcacactggccatatcggtggtcatcatgcgccagctttcatccccgatatgcaccaccgggtaaagttcacgggagactttatctgacagcagacgtgcactggccagggggatcaccatccgtcgcccgggcgtgtcaataatatcactctgtacatccacaaacagacgataacggctctctcttttataggtgtaaaccttaaactgcatttcaccagtccctgttctcgtcagcaaaagagccgttcatttcaataaaccgggcgacctcagccatcccttcctgattttccgctttccagcgttcggcacgcagacgacgggcttcattctgcatggttgtgcttaccagaccggagatattgacatcatatatgccttgagcaactgatagctgtcgctgtcaactgtcactgtaatacgctgcttcatagcacacctctttttgacatacttcgggtatacatatcagtatatattcttataccgcaaaaatcagcgcgcaaatacgcatactgttatctggcttttagtaagccggatccacgcgattacgccccgccctgccactcatcgcagtactgttgtaattcattaagcattctgccgacatggaagccatcacagacggcatgatgaacctgaatcgccagcggcatcagcaccttgtcgccttgcgtataatatttgcccatggtgaaaacgggggcgaagaagttgtccatattggccacgtttaaatcaaaactggtgaaactcacccagggattggctgagacgaaaaacatattctcaataaaccctttagggaaataggccaggttttcaccgtaacacgccacatcttgcgaatatatgtgtagaaactgccggaaatcgtcgtggtattcactccagagcgatgaaaacgtttcagtttgctcatggaaaacggtgtaacaagggtgaacactatcccatatcaccagctcaccgtctttcattgccatacggaattccggatgagcattcatcaggcgggcaagaatgtgaataaaggccggataaaacttgtgcttatttttctttacggtctttaaaaaggccgtaatatccagctgaacggtctggttataggtacattgagcaactgactgaaatgcctcaaaatgttctttacgatgccattgggatatatcaacggtggtatatccagtgatttttttctccattttagcttccttagctcctgaaaatctcgataactcaaaaaatacgcccggtagtgatcttatttcattatggtgaaagttggaacctcttacgtgccgatcaacgtctcattttcgccaaaagttggcccagggcttcccggtatcaacagggacaccaggatttatttattctgcgaagtgatcttccgtcacaggtatttattcggcgcaaagtgcgtcgggtgatgctgccaacttagtcgactacaggtcactaataccatctaagtagttgattcatagtgactggatatgttgtgttttacagtattatgtagtctgttttttatgcaaaatctaatttaatatattgatatttatatcattttacgtttctcgttcaacttttctatacaaagttgccccgacctgcaggcatgcaagcttggcactggccgtcgttttacaacgtcgtgactgggaaaaccctggcgttacccaacttaatcgccttgcagcacatccccctttcgccagctggcgtaatagcgaagaggcccgcaccgatcgcccttcccaacagttgcgcagcctgaatggcgaatggcgcctgatgcggtattttctccttacgcatctgtgcggtatttcacaccgcatatggtgcactctcagtacaatctgctctgatgccgcatagttaagccagccccgacacccgccaacacccgctgacgcgccctgacgggcttgtctgctcccggcatccgcttacagacaagctgtgaccgtctccgggagctgcatgtgtcagaggttttcaccgtcatcaccgaaacgcgcgagacgaaagggcctcgtgatacgcctatttttataggttaatgtcatgataataatggtttcttagacgtcaggtggcacttttcggggaaatgtgcgcggaacccctatttgtttatttttctaaatacattcaaatatgtatccgctcatgagacaataaccctgataaatgcttcaataatattgaaaaaggaagagtatgagtattcaacatttccgtgtcgcccttattcccttttttgcggcattttgccttcctgtttttgctcacccagaaacgctggtgaaagtaaaagatgctgaagatcagttgggtgcacgagtgggttacatcgaactggatctcaacagcggtaagatccttgagagttttcgccccgaagaacgttttccaatgatgagcacttttaaagttctgctatgtggcgcggtattatcccgtattgacgccgggcaagagcaactcggtcgccgcatacactattctcagaatgacttggttgagtactcaccagtcacagaaaagcatcttacggatggcatgacagtaagagaattatgcagtgctgccataaccatgagtgataacactgcggccaacttacttctgacaacgatcggaggaccgaaggagctaaccgcttttttgcacaacatgggggatcatgtaactcgccttgatcgttgggaaccggagctgaatgaagccataccaaacgacgagcgtgacaccacgatgcctgtagcaatggcaacaacgttgcgcaaactattaactggcgaactacttactctagcttcccggcaacaattaatagactggatggaggcggataaagttgcaggaccacttctgcgctcggcccttccggctggctggtttattgctgataaatctggagccggtgagcgtgggtctcgcggtatcattgcagcactggggccagatggtaagccctcccgtatcgtagttatctacacgacggggagtcaggcaactatggatgaacgaaatagacagatcgctgagataggtgcctcactgattaagcattggtaactgtcagaccaagtttactcatatatactttagattgatttaaaacttcatttttaatttaaaaggatctaggtgaagatcctttttgataatctcatgaccaaaatcccttaacgtgagttttcgttccactgagcgtcagaccccgtagaaaagatcaaaggatcttcttgagatcctttttttctgcgcgtaatctgctgcttgcaaacaaaaaaaccaccgctaccagcggtggtttgtttgccggatcaagagctaccaactctttttccgaaggtaactggcttcagcagagcgcagataccaaatactgtccttctagtgtagccgtagttaggccaccacttcaagaactctgtagcaccgcctacatacctcgctctgctaatcctgttaccagtggctgctgccagtggcgataagtcgtgtcttaccgggttggactcaagacgatagttaccggataaggcgcagcggtcgggctgaacggggggttcgtgcacacagcccagcttggagcgaacgacctacaccgaactgagatacctacagcgtgagctatgagaaagcgccacgcttcccgaagggagaaaggcggacaggtatccggtaagcggcagggtcggaacaggagagcgcacgagggagcttccagggggaaacgcctggtatctttatagtcctgtcgggtttcgccacctctgacttgagcgtcgatttttgtgatgctcgtcaggggggcggagcctatggaaaaacgccagcaacgcggcctttttacggttcctggccttttgctggccttttgctcacatgttctttcctgcgttatcccctgattctgtggataaccgtattaccgcctttgagtgagctgataccgctcgccgcagccgaacgaccgagcgcagcgagtcagtgagcgaggaagcggaagagcgcccaatacgcaaaccgcctctccccgcgcgttggccgattcattaatgcagctggcacgacaggtttcccgactggaaagcgggcagtgagcgcaacgcaattaatgtgagttagctcactcattaggcaccccaggctttacactttatgcttccggctcgtatgttgtgtggaattgtgagcggataacaatttcacacaggaaacagctatgaccatgattacgaattcgagctcggtac

pUC-DEST-R1R2

cgagctcggatccactagtccagtgtggtggaattctgcagatatcaacaagtttgtacaaaaaagctgaacgagaaacgtaaaatgatataaatatcaatatattaaattagattttgcataaaaaacagactacataatactgtaaaacacaacatatccagtcactatggcggccgcattaggcaccccaggctttacactttatgcttccggctcgtataatgtgtggattttgagttaggatccggcgagattttcaggagctaaggaagctaaaatggagaaaaaaatcactggatataccaccgttgatatatcccaatggcatcgtaaagaacattttgaggcatttcagtcagttgctcaatgtacctataaccagaccgttcagctggatattacggcctttttaaagaccgtaaagaaaaataagcacaagttttatccggcctttattcacattcttgcccgcctgatgaatgctcatccggaattccgtatggcaatgaaagacggtgagctggtgatatgggatagtgttcacccttgttacaccgttttccatgagcaaactgaaacgttttcatcgctctggagtgaataccacgacgatttccggcagtttctacacatatattcgcaagatgtggcgtgttacggtgaaaacctggcctatttccctaaagggtttattgagaatatgtttttcgtctcagccaatccctgggtgagtttcaccagttttgatttaaacgtggccaatatggacaacttcttcgcccccgttttcaccatgggcaaatattatacgcaaggcgacaaggtgctgatgccgctggcgattcaggttcatcatgccgtctgtgatggcttccatgtcggcagaatgcttaatgaattacaacagtactgcgatgagtggcagggcggggcgtaaagatctggatccggcttactaaaagccagataacagtatgcgtatttgcgcgctgatttttgcggtataagaatatatactgatatgtatacccgaagtatgtcaaaaagaggtgtgctatgaagcagcgtattacagtgacagttgacagcgacagctatcagttgctcaaggcatatatgatgtcaatatctccggtctggtaagcacaaccatgcagaatgaagcccgtcgtctgcgtgccgaacgctggaaagcggaaaatcaggaagggatggctgaggtcgcccggtttattgaaatgaacggctcttttgctgacgagaacagggactggtgaaatgcagtttaaggtttacacctataaaagagagagccgttatcgtctgtttgtggatgtacagagtgatattattgacacgcccgggcgacggatggtgatccccctggccagtgcacgtctgctgtcagataaagtctcccgtgaactttacccggtggtgcatatcggggatgaaagctggcgcatgatgaccaccgatatggccagtgtgccggtctccgttatcggggaagaagtggctgatctcagccaccgcgaaaatgacatcaaaaacgccattaacctgatgttctggggaatataaatgtcaggctccgttatacacagccagtctgcaggtcgaccatagtgactggatatgttgtgttttacagtattatgtagtctgttttttatgcaaaatctaatttaatatattgatatttatatcattttacgtttctcgttcagctttcttgtacaaagtggttgatatccagcacagtggcggccgctcgagtctagagtcgacctgcaggcatgcaagcttggcactggccgtcgttttacaacgtcgtgactgggaaaaccctggcgttacccaacttaatcgccttgcagcacatccccctttcgccagctggcgtaatagcgaagaggcccgcaccgatcgcccttcccaacagttgcgcagcctgaatggcgaatggcgcctgatgcggtattttctccttacgcatctgtgcggtatttcacaccgcatatggtgcactctcagtacaatctgctctgatgccgcatagttaagccagccccgacacccgccaacacccgctgacgcgccctgacgggcttgtctgctcccggcatccgcttacagacaagctgtgaccgtctccgggagctgcatgtgtcagaggttttcaccgtcatcaccgaaacgcgcgagacgaaagggcctcgtgatacgcctatttttataggttaatgtcatgataataatggtttcttagacgtcaggtggcacttttcggggaaatgtgcgcggaacccctatttgtttatttttctaaatacattcaaatatgtatccgctcatgagacaataaccctgataaatgcttcaataatattgaaaaaggaagagtatgagtattcaacatttccgtgtcgcccttattcccttttttgcggcattttgccttcctgtttttgctcacccagaaacgctggtgaaagtaaaagatgctgaagatcagttgggtgcacgagtgggttacatcgaactggatctcaacagcggtaagatccttgagagttttcgccccgaagaacgttttccaatgatgagcacttttaaagttctgctatgtggcgcggtattatcccgtattgacgccgggcaagagcaactcggtcgccgcatacactattctcagaatgacttggttgagtactcaccagtcacagaaaagcatcttacggatggcatgacagtaagagaattatgcagtgctgccataaccatgagtgataacactgcggccaacttacttctgacaacgatcggaggaccgaaggagctaaccgcttttttgcacaacatgggggatcatgtaactcgccttgatcgttgggaaccggagctgaatgaagccataccaaacgacgagcgtgacaccacgatgcctgtagcaatggcaacaacgttgcgcaaactattaactggcgaactacttactctagcttcccggcaacaattaatagactggatggaggcggataaagttgcaggaccacttctgcgctcggcccttccggctggctggtttattgctgataaatctggagccggtgagcgtgggtctcgcggtatcattgcagcactggggccagatggtaagccctcccgtatcgtagttatctacacgacggggagtcaggcaactatggatgaacgaaatagacagatcgctgagataggtgcctcactgattaagcattggtaactgtcagaccaagtttactcatatatactttagattgatttaaaacttcatttttaatttaaaaggatctaggtgaagatcctttttgataatctcatgaccaaaatcccttaacgtgagttttcgttccactgagcgtcagaccccgtagaaaagatcaaaggatcttcttgagatcctttttttctgcgcgtaatctgctgcttgcaaacaaaaaaaccaccgctaccagcggtggtttgtttgccggatcaagagctaccaactctttttccgaaggtaactggcttcagcagagcgcagataccaaatactgtccttctagtgtagccgtagttaggccaccacttcaagaactctgtagcaccgcctacatacctcgctctgctaatcctgttaccagtggctgctgccagtggcgataagtcgtgtcttaccgggttggactcaagacgatagttaccggataaggcgcagcggtcgggctgaacggggggttcgtgcacacagcccagcttggagcgaacgacctacaccgaactgagatacctacagcgtgagctatgagaaagcgccacgcttcccgaagggagaaaggcggacaggtatccggtaagcggcagggtcggaacaggagagcgcacgagggagcttccagggggaaacgcctggtatctttatagtcctgtcgggtttcgccacctctgacttgagcgtcgatttttgtgatgctcgtcaggggggcggagcctatggaaaaacgccagcaacgcggcctttttacggttcctggccttttgctggccttttgctcacatgttctttcctgcgttatcccctgattctgtggataaccgtattaccgcctttgagtgagctgataccgctcgccgcagccgaacgaccgagcgcagcgagtcagtgagcgaggaagcggaagagcgcccaatacgcaaaccgcctctccccgcgcgttggccgattcattaatgcagctggcacgacaggtttcccgactggaaagcgggcagtgagcgcaacgcaattaatgtgagttagctcactcattaggcaccccaggctttacactttatgcttccggctcgtatgttgtgtggaattgtgagcggataacaatttcacacaggaaacagctatgaccatgattacgaattcgagctcggtac

pPB-DEST-R1R2-pNeo

ctaaattgtaagcgttaatattttgttaaaattcgcgttaaatttttgttaaatcagctcattttttaaccaataggccgaaatcggcaaaatcccttataaatcaaaagaatagaccgagatagggttgagtgttgttccagtttggaacaagagtccactattaaagaacgtggactccaacgtcaaagggcgaaaaaccgtctatcagggcgatggcccactacgtgaaccatcaccctaatcaagttttttggggtcgaggtgccgtaaagcactaaatcggaaccctaaagggagcccccgatttagagcttgacggggaaagccggcgaacgtggcgagaaaggaagggaagaaagcgaaaggagcgggcgctagggcgctggcaagtgtagcggtcacgctgcgcgtaaccaccacacccgccgcgcttaatgcgccgctacagggcgcgtcccattcgccattcaggctgcgcaactgttgggaagggcgatcggtgcgggcctcttcgctattacgccagctggcgaaagggggatgtgctgcaaggcgattaagttgggtaacgccagggttttcccagtcacgacgttgtaaaacgacggccagtgaattgtaatacgactcactatagggcgaattgggtaccttaaccctagaaagatagtctgcgtaaaattgacgcatgcattcttgaaatattgctctctctttctaaatagcgcgaatccgtcgctgtgcatttaggacatctcagtcgccgcttggagctcccgtgaggcgtgcttgtcaatgcggtaagtgtcactgattttgaactataacgaccgcgtgagtcaaaatgacgcatgattatcttttacgtgacttttaagatttaactcatacgataattatattgttatttcatgttctacttacgtgataacttattatatatatattttcttgttatagatgatccactagtaacggccgccagtgtgctggaattcgcggccgctagcatcacaagtttgtacaaaaaagctgaacgagaaacgtaaaatgatataaatatcaatatattaaattagattttgcataaaaaacagactacataatactgtaaaacacaacatatccagtcactatggcggccgcattaggcaccccaggctttacactttatgcttccggctcgtataatgtgtggattttgagttaggatccgtcgagattttcaggagctaaggaagctaaaatggagaaaaaaatcactggatataccaccgttgatatatcccaatggcatcgtaaagaacattttgaggcatttcagtcagttgctcaatgtacctataaccagaccgttcagctggatattacggcctttttaaagaccgtaaagaaaaataagcacaagttttatccggcctttattcacattcttgcccgcctgatgaatgctcatccggaattccgtatggcaatgaaagacggtgagctggtgatatgggatagtgttcacccttgttacaccgttttccatgagcaaactgaaacgttttcatcgctctggagtgaataccacgacgatttccggcagtttctacacatatattcgcaagatgtggcgtgttacggtgaaaacctggcctatttccctaaagggtttattgagaatatgtttttcgtctcagccaatccctgggtgagtttcaccagttttgatttaaacgtggccaatatggacaacttcttcgcccccgttttcaccatgggcaaatattatacgcaaggcgacaaggtgctgatgccgctggcgattcaggttcatcatgccgtttgtgatggcttccatgtcggcagaatgcttaatgaattacaacagtactgcgatgagtggcagggcggggcgtaaacgcgtggatccggcttactaaaagccagataacagtatgcgtatttgcgcgctgatttttgcggtataagaatatatactgatatgtatacccgaagtatgtcaaaaagaggtatgctatgaagcagcgtattacagtgacagttgacagcgacagctatcagttgctcaaggcatatatgatgtcaatatctccggtctggtaagcacaaccatgcagaatgaagcccgtcgtctgcgtgccgaacgctggaaagcggaaaatcaggaagggatggctgaggtcgcccggtttattgaaatgaacggctcttttgctgacgagaacaggggctggtgaaatgcagtttaaggtttacacctataaaagagagagccgttatcgtctgtttgtggatgtacagagtgatattattgacacgcccgggcgacggatggtgatccccctggccagtgcacgtctgctgtcagataaagtctcccgtgaactttacccggtggtgcatatcggggatgaaagctggcgcatgatgaccaccgatatggccagtgtgccggtctccgttatcggggaagaagtggctgatctcagccaccgcgaaaatgacatcaaaaacgccattaacctgatgttctggggaatataaatgtcaggctcccttatacacagccagtctgcaggtcgaccatagtgactggatatgttgtgttttacagtattatgtagtctgttttttatgcaaaatctaatttaatatattgatatttatatcattttacgtttctcgttcagctttcttgtacaaagtggtgatgctaccggaacatcgagggatcaagctttaatgcggtagtttatcacagttaaattgctaacgcagtcaggcaccgtgtatgaaatctaacaatgcgctcatcgtcatcctcggcaccgtcaccctggatgctgtaggcataggcttggttatgccggtactgccgggcctcttgcggggaattctaccgggtaggggaggcgcttttcccaaggcagtctggagcatgcgctttagcagccccgctgggcacttggcgctacacaagtggcctctggcctcgcacacattccacatccaccggtaggcgccaaccggctccgttctttggtggccccttcgcgccaccttctactcctcccctagtcaggaagttcccccccgccccgcagctcgcgtcgtgcaggacgtgacaaatggaagtagcacgtctcactagtctcgtgcagatggacagcaccgctgagcaatggaagcgggtaggcctttggggcagcggccaatagcagctttgctccttcgctttctgggctcagaggctgggaaggggtgggtccgggggcgggctcaggggcgggctcaggggcggggcgggcgcccgaaggtcctccggaggcccggcattctgcacgcttcaaaagcgcacgtctgccgcgctgttctcctcttcctcatctccgggcctttcgacctgcagccaatatgggatcggccattgaacaagatggattgcacgcaggttctccggccgcttgggtggagaggctattcggctatgactgggcacaacagacaatcggctgctctgatgccgccgtgttccggctgtcagcgcaggggcgcccggttctttttgtcaagaccgacctgtccggtgccctgaatgaactgcaggacgaggcagcgcggctatcgtggctggccacgacgggcgttccttgcgcagctgtgctcgacgttgtcactgaagcgggaagggactggctgctattgggcgaagtgccggggcaggatctcctgtcatctcaccttgctcctgccgagaaagtatccatcatggctgatgcaatgcggcggctgcatacgcttgatccggctacctgcccattcgaccaccaagcgaaacatcgcatcgagcgagcacgtactcggatggaagccggtcttgtcgatcaggatgatctggacgaagagcatcaggggctcgcgccagccgaactgttcgccaggctcaaggcgcgcatgcccgacggcgatgatctcgtcgtgacccatggcgatgcctgcttgccgaatatcatggtggaaaatggccgcttttctggattcatcgactgtggccggctgggtgtggcggaccgctatcaggacatagcgttggctacccgtgatattgctgaagagcttggcggcgaatgggctgaccgcttcctcgtgctttacggtatcgccgctcccgattcgcagcgcatcgccttctatcgccttcttgacgagttcttctgaggggatcgatccgctgtaagtctgcagaaattgatgatctattaaacaataaagatgtccactaaaatggaagtttttcctgtcatactttgttaagaagggtgagaacagagtacctacattttgaatggaaggattggagctacgggggtgggggtggggtgggattagataaatgcctgctctttactgaaggctctttactattgctttatgataatgtttcatagttggatatcagatcctttgttactttatagaagaaattttgagtttttgtttttttttaataaataaataaacataaataaattgtttgttgaatttattattagtatgtaagtgtaaatataataaaacttaatatctattcaaattaataaataaacctcgatatacagaccgataaaacacatgcgtcaattttacgcatgattatctttaacgtacgtcacaatatgattatctttctagggttaaagagctccagcttttgttccctttagtgagggttaatttcgagcttggcgtaatcatggtcatagctgtttcctgtgtgaaattgttatccgctcacaattccacacaacatacgagccggaagcataaagtgtaaagcctggggtgcctaatgagtgagctaactcacattaattgcgttgcgctcactgcccgctttccagtcgggaaacctgtcgtgccagctgcattaatgaatcggccaacgcgcggggagaggcggtttgcgtattgggcgctcttccgcttcctcgctcactgactcgctgcgctcggtcgttcggctgcggcgagcggtatcagctcactcaaaggcggtaatacggttatccacagaatcaggggataacgcaggaaagaacatgtgagcaaaaggccagcaaaaggccaggaaccgtaaaaaggccgcgttgctggcgtttttccataggctccgcccccctgacgagcatcacaaaaatcgacgctcaagtcagaggtggcgaaacccgacaggactataaagataccaggcgtttccccctggaagctccctcgtgcgctctcctgttccgaccctgccgcttaccggatacctgtccgcctttctcccttcgggaagcgtggcgctttctcatagctcacgctgtaggtatctcagttcggtgtaggtcgttcgctccaagctgggctgtgtgcacgaaccccccgttcagcccgaccgctgcgccttatccggtaactatcgtcttgagtccaacccggtaagacacgacttatcgccactggcagcagccactggtaacaggattagcagagcgaggtatgtaggcggtgctacagagttcttgaagtggtggcctaactacggctacactagaaggacagtatttggtatctgcgctctgctgaagccagttaccttcggaaaaagagttggtagctcttgatccggcaaacaaaccaccgctggtagcggtggtttttttgtttgcaagcagcagattacgcgcagaaaaaaaggatctcaagaagatcctttgatcttttctacggggtctgacgctcagtggaacgaaaactcacgttaagggattttggtcatgagattatcaaaaaggatcttcacctagatccttttaaattaaaaatgaagttttaaatcaatctaaagtatatatgagtaaacttggtctgacagttaccaatgcttaatcagtgaggcacctatctcagcgatctgtctatttcgttcatccatagttgcctgactccccgtcgtgtagataactacgatacgggagggcttaccatctggccccagtgctgcaatgataccgcgagacccacgctcaccggctccagatttatcagcaataaaccagccagccggaagggccgagcgcagaagtggtcctgcaactttatccgcctccatccagtctattaattgttgccgggaagctagagtaagtagttcgccagttaatagtttgcgcaacgttgttgccattgctacaggcatcgtggtgtcacgctcgtcgtttggtatggcttcattcagctccggttcccaacgatcaaggcgagttacatgatcccccatgttgtgcaaaaaagcggttagctccttcggtcctccgatcgttgtcagaagtaagttggccgcagtgttatcactcatggttatggcagcactgcataattctcttactgtcatgccatccgtaagatgcttttctgtgactggtgagtactcaaccaagtcattctgagaatagtgtatgcggcgaccgagttgctcttgcccggcgtcaatacgggataataccgcgccacatagcagaactttaaaagtgctcatcattggaaaacgttcttcggggcgaaaactctcaaggatcttaccgctgttgagatccagttcgatgtaacccactcgtgcacccaactgatcttcagcatcttttactttcaccagcgtttctgggtgagcaaaaacaggaaggcaaaatgccgcaaaaaagggaataagggcgacacggaaatgttgaatactcatactcttcctttttcaatattattgaagcatttatcagggttattgtctcatgagcggatacatatttgaatgtatttagaaaaataaacaaataggggttccgcgcacatttccccgaaaagtgccac

pPB-DEST-R5R4-pNeo

ctaaattgtaagcgttaatattttgttaaaattcgcgttaaatttttgttaaatcagctcattttttaaccaataggccgaaatcggcaaaatcccttataaatcaaaagaatagaccgagatagggttgagtgttgttccagtttggaacaagagtccactattaaagaacgtggactccaacgtcaaagggcgaaaaaccgtctatcagggcgatggcccactacgtgaaccatcaccctaatcaagttttttggggtcgaggtgccgtaaagcactaaatcggaaccctaaagggagcccccgatttagagcttgacggggaaagccggcgaacgtggcgagaaaggaagggaagaaagcgaaaggagcgggcgctagggcgctggcaagtgtagcggtcacgctgcgcgtaaccaccacacccgccgcgcttaatgcgccgctacagggcgcgtcccattcgccattcaggctgcgcaactgttgggaagggcgatcggtgcgggcctcttcgctattacgccagctggcgaaagggggatgtgctgcaaggcgattaagttgggtaacgccagggttttcccagtcacgacgttgtaaaacgacggccagtgaattgtaatacgactcactatagggcgaattgggtaccttaaccctagaaagatagtctgcgtaaaattgacgcatgcattcttgaaatattgctctctctttctaaatagcgcgaatccgtcgctgtgcatttaggacatctcagtcgccgcttggagctcccgtgaggcgtgcttgtcaatgcggtaagtgtcactgattttgaactataacgaccgcgtgagtcaaaatgacgcatgattatcttttacgtgacttttaagatttaactcatacgataattatattgttatttcatgttctacttacgtgataacttattatatatatattttcttgttatagatgatccactagtaacggccgccagtgtgctggaattcgcggccgctagagtcggggcaactttgtatacaaaagttgaacgagaaacgtaaaatgatataaatatcaatatattaaattagattttgcataaaaaacagactacataatactgtaaaacacaacatatccagtcactatgaatcaactacttagatggtattagtgacctgtagtcgaccgacagccttccaaatgttcttcgggtgatgctgccaacttagtcgaccgacagccttccaaatgttcttctcaaacggaatcgtcgtatccagcctactcgctattgtcctcaatgccgtattaaatcataaaaagaaataagaaaaagaggtgcgagcctcttttttgtgtgacaaaataaaaacatctacctattcatatacgctagtgtcatagtcctgaaaatcatctgcatcaagaacaatttcacaactcttatacttttctcttacaagtcgttcggcttcatctggattttcagcctctatacttactaaacgtgataaagtttctgtaatttctactgtatcgacctgcagactggctgtgtataagggagcctgacatttatattccccagaacatcaggttaatggcgtttttgatgtcattttcgcggtggctgagatcagccacttcttccccgataacggagaccggcacactggccatatcggtggtcatcatgcgccagctttcatccccgatatgcaccaccgggtaaagttcacgggagactttatctgacagcagacgtgcactggccagggggatcaccatccgtcgcccgggcgtgtcaataatatcactctgtacatccacaaacagacgataacggctctctcttttataggtgtaaaccttaaactgcatttcaccagtccctgttctcgtcagcaaaagagccgttcatttcaataaaccgggcgacctcagccatcccttcctgattttccgctttccagcgttcggcacgcagacgacgggcttcattctgcatggttgtgcttaccagaccggagatattgacatcatatatgccttgagcaactgatagctgtcgctgtcaactgtcactgtaatacgctgcttcatagcacacctctttttgacatacttcgggtatacatatcagtatatattcttataccgcaaaaatcagcgcgcaaatacgcatactgttatctggcttttagtaagccggatccacgcgattacgccccgccctgccactcatcgcagtactgttgtaattcattaagcattctgccgacatggaagccatcacagacggcatgatgaacctgaatcgccagcggcatcagcaccttgtcgccttgcgtataatatttgcccatggtgaaaacgggggcgaagaagttgtccatattggccacgtttaaatcaaaactggtgaaactcacccagggattggctgagacgaaaaacatattctcaataaaccctttagggaaataggccaggttttcaccgtaacacgccacatcttgcgaatatatgtgtagaaactgccggaaatcgtcgtggtattcactccagagcgatgaaaacgtttcagtttgctcatggaaaacggtgtaacaagggtgaacactatcccatatcaccagctcaccgtctttcattgccatacggaattccggatgagcattcatcaggcgggcaagaatgtgaataaaggccggataaaacttgtgcttatttttctttacggtctttaaaaaggccgtaatatccagctgaacggtctggttataggtacattgagcaactgactgaaatgcctcaaaatgttctttacgatgccattgggatatatcaacggtggtatatccagtgatttttttctccattttagcttccttagctcctgaaaatctcgataactcaaaaaatacgcccggtagtgatcttatttcattatggtgaaagttggaacctcttacgtgccgatcaacgtctcattttcgccaaaagttggcccagggcttcccggtatcaacagggacaccaggatttatttattctgcgaagtgatcttccgtcacaggtatttattcggcgcaaagtgcgtcgggtgatgctgccaacttagtcgactacaggtcactaataccatctaagtagttgattcatagtgactggatatgttgtgttttacagtattatgtagtctgttttttatgcaaaatctaatttaatatattgatatttatatcattttacgtttctcgttcaacttttctatacaaagttgccccgacctgcaggcatgcaagctttaatgcggtagtttatcacagttaaattgctaacgcagtcaggcaccgtgtatgaaatctaacaatgcgctcatcgtcatcctcggcaccgtcaccctggatgctgtaggcataggcttggttatgccggtactgccgggcctcttgcggggaattctaccgggtaggggaggcgcttttcccaaggcagtctggagcatgcgctttagcagccccgctgggcacttggcgctacacaagtggcctctggcctcgcacacattccacatccaccggtaggcgccaaccggctccgttctttggtggccccttcgcgccaccttctactcctcccctagtcaggaagttcccccccgccccgcagctcgcgtcgtgcaggacgtgacaaatggaagtagcacgtctcactagtctcgtgcagatggacagcaccgctgagcaatggaagcgggtaggcctttggggcagcggccaatagcagctttgctccttcgctttctgggctcagaggctgggaaggggtgggtccgggggcgggctcaggggcgggctcaggggcggggcgggcgcccgaaggtcctccggaggcccggcattctgcacgcttcaaaagcgcacgtctgccgcgctgttctcctcttcctcatctccgggcctttcgacctgcagccaatatgggatcggccattgaacaagatggattgcacgcaggttctccggccgcttgggtggagaggctattcggctatgactgggcacaacagacaatcggctgctctgatgccgccgtgttccggctgtcagcgcaggggcgcccggttctttttgtcaagaccgacctgtccggtgccctgaatgaactgcaggacgaggcagcgcggctatcgtggctggccacgacgggcgttccttgcgcagctgtgctcgacgttgtcactgaagcgggaagggactggctgctattgggcgaagtgccggggcaggatctcctgtcatctcaccttgctcctgccgagaaagtatccatcatggctgatgcaatgcggcggctgcatacgcttgatccggctacctgcccattcgaccaccaagcgaaacatcgcatcgagcgagcacgtactcggatggaagccggtcttgtcgatcaggatgatctggacgaagagcatcaggggctcgcgccagccgaactgttcgccaggctcaaggcgcgcatgcccgacggcgatgatctcgtcgtgacccatggcgatgcctgcttgccgaatatcatggtggaaaatggccgcttttctggattcatcgactgtggccggctgggtgtggcggaccgctatcaggacatagcgttggctacccgtgatattgctgaagagcttggcggcgaatgggctgaccgcttcctcgtgctttacggtatcgccgctcccgattcgcagcgcatcgccttctatcgccttcttgacgagttcttctgaggggatcgatccgctgtaagtctgcagaaattgatgatctattaaacaataaagatgtccactaaaatggaagtttttcctgtcatactttgttaagaagggtgagaacagagtacctacattttgaatggaaggattggagctacgggggtgggggtggggtgggattagataaatgcctgctctttactgaaggctctttactattgctttatgataatgtttcatagttggatatcagatcctttgttactttatagaagaaattttgagtttttgtttttttttaataaataaataaacataaataaattgtttgttgaatttattattagtatgtaagtgtaaatataataaaacttaatatctattcaaattaataaataaacctcgatatacagaccgataaaacacatgcgtcaattttacgcatgattatctttaacgtacgtcacaatatgattatctttctagggttaaagagctccagcttttgttccctttagtgagggttaatttcgagcttggcgtaatcatggtcatagctgtttcctgtgtgaaattgttatccgctcacaattccacacaacatacgagccggaagcataaagtgtaaagcctggggtgcctaatgagtgagctaactcacattaattgcgttgcgctcactgcccgctttccagtcgggaaacctgtcgtgccagctgcattaatgaatcggccaacgcgcggggagaggcggtttgcgtattgggcgctcttccgcttcctcgctcactgactcgctgcgctcggtcgttcggctgcggcgagcggtatcagctcactcaaaggcggtaatacggttatccacagaatcaggggataacgcaggaaagaacatgtgagcaaaaggccagcaaaaggccaggaaccgtaaaaaggccgcgttgctggcgtttttccataggctccgcccccctgacgagcatcacaaaaatcgacgctcaagtcagaggtggcgaaacccgacaggactataaagataccaggcgtttccccctggaagctccctcgtgcgctctcctgttccgaccctgccgcttaccggatacctgtccgcctttctcccttcgggaagcgtggcgctttctcatagctcacgctgtaggtatctcagttcggtgtaggtcgttcgctccaagctgggctgtgtgcacgaaccccccgttcagcccgaccgctgcgccttatccggtaactatcgtcttgagtccaacccggtaagacacgacttatcgccactggcagcagccactggtaacaggattagcagagcgaggtatgtaggcggtgctacagagttcttgaagtggtggcctaactacggctacactagaaggacagtatttggtatctgcgctctgctgaagccagttaccttcggaaaaagagttggtagctcttgatccggcaaacaaaccaccgctggtagcggtggtttttttgtttgcaagcagcagattacgcgcagaaaaaaaggatctcaagaagatcctttgatcttttctacggggtctgacgctcagtggaacgaaaactcacgttaagggattttggtcatgagattatcaaaaaggatcttcacctagatccttttaaattaaaaatgaagttttaaatcaatctaaagtatatatgagtaaacttggtctgacagttaccaatgcttaatcagtgaggcacctatctcagcgatctgtctatttcgttcatccatagttgcctgactccccgtcgtgtagataactacgatacgggagggcttaccatctggccccagtgctgcaatgataccgcgagacccacgctcaccggctccagatttatcagcaataaaccagccagccggaagggccgagcgcagaagtggtcctgcaactttatccgcctccatccagtctattaattgttgccgggaagctagagtaagtagttcgccagttaatagtttgcgcaacgttgttgccattgctacaggcatcgtggtgtcacgctcgtcgtttggtatggcttcattcagctccggttcccaacgatcaaggcgagttacatgatcccccatgttgtgcaaaaaagcggttagctccttcggtcctccgatcgttgtcagaagtaagttggccgcagtgttatcactcatggttatggcagcactgcataattctcttactgtcatgccatccgtaagatgcttttctgtgactggtgagtactcaaccaagtcattctgagaatagtgtatgcggcgaccgagttgctcttgcccggcgtcaatacgggataataccgcgccacatagcagaactttaaaagtgctcatcattggaaaacgttcttcggggcgaaaactctcaaggatcttaccgctgttgagatccagttcgatgtaacccactcgtgcacccaactgatcttcagcatcttttactttcaccagcgtttctgggtgagcaaaaacaggaaggcaaaatgccgcaaaaaagggaataagggcgacacggaaatgttgaatactcatactcttcctttttcaatattattgaagcatttatcagggttattgtctcatgagcggatacatatttgaatgtatttagaaaaataaacaaataggggttccgcgcacatttccccgaaaagtgccac

pPB-DEST-R5R4-pHygTK

ctaaattgtaagcgttaatattttgttaaaattcgcgttaaatttttgttaaatcagctcattttttaaccaataggccgaaatcggcaaaatcccttataaatcaaaagaatagaccgagatagggttgagtgttgttccagtttggaacaagagtccactattaaagaacgtggactccaacgtcaaagggcgaaaaaccgtctatcagggcgatggcccactacgtgaaccatcaccctaatcaagttttttggggtcgaggtgccgtaaagcactaaatcggaaccctaaagggagcccccgatttagagcttgacggggaaagccggcgaacgtggcgagaaaggaagggaagaaagcgaaaggagcgggcgctagggcgctggcaagtgtagcggtcacgctgcgcgtaaccaccacacccgccgcgcttaatgcgccgctacagggcgcgtcccattcgccattcaggctgcgcaactgttgggaagggcgatcggtgcgggcctcttcgctattacgccagctggcgaaagggggatgtgctgcaaggcgattaagttgggtaacgccagggttttcccagtcacgacgttgtaaaacgacggccagtgaattgtaatacgactcactatagggcgaattgggtaccttaaccctagaaagatagtctgcgtaaaattgacgcatgcattcttgaaatattgctctctctttctaaatagcgcgaatccgtcgctgtgcatttaggacatctcagtcgccgcttggagctcccgtgaggcgtgcttgtcaatgcggtaagtgtcactgattttgaactataacgaccgcgtgagtcaaaatgacgcatgattatcttttacgtgacttttaagatttaactcatacgataattatattgttatttcatgttctacttacgtgataacttattatatatatattttcttgttatagatgatccactagtaacggccgccagtgtgctggaattcgcggccgctagagtcggggcaactttgtatacaaaagttgaacgagaaacgtaaaatgatataaatatcaatatattaaattagattttgcataaaaaacagactacataatactgtaaaacacaacatatccagtcactatgaatcaactacttagatggtattagtgacctgtagtcgaccgacagccttccaaatgttcttcgggtgatgctgccaacttagtcgaccgacagccttccaaatgttcttctcaaacggaatcgtcgtatccagcctactcgctattgtcctcaatgccgtattaaatcataaaaagaaataagaaaaagaggtgcgagcctcttttttgtgtgacaaaataaaaacatctacctattcatatacgctagtgtcatagtcctgaaaatcatctgcatcaagaacaatttcacaactcttatacttttctcttacaagtcgttcggcttcatctggattttcagcctctatacttactaaacgtgataaagtttctgtaatttctactgtatcgacctgcagactggctgtgtataagggagcctgacatttatattccccagaacatcaggttaatggcgtttttgatgtcattttcgcggtggctgagatcagccacttcttccccgataacggagaccggcacactggccatatcggtggtcatcatgcgccagctttcatccccgatatgcaccaccgggtaaagttcacgggagactttatctgacagcagacgtgcactggccagggggatcaccatccgtcgcccgggcgtgtcaataatatcactctgtacatccacaaacagacgataacggctctctcttttataggtgtaaaccttaaactgcatttcaccagtccctgttctcgtcagcaaaagagccgttcatttcaataaaccgggcgacctcagccatcccttcctgattttccgctttccagcgttcggcacgcagacgacgggcttcattctgcatggttgtgcttaccagaccggagatattgacatcatatatgccttgagcaactgatagctgtcgctgtcaactgtcactgtaatacgctgcttcatagcacacctctttttgacatacttcgggtatacatatcagtatatattcttataccgcaaaaatcagcgcgcaaatacgcatactgttatctggcttttagtaagccggatccacgcgattacgccccgccctgccactcatcgcagtactgttgtaattcattaagcattctgccgacatggaagccatcacagacggcatgatgaacctgaatcgccagcggcatcagcaccttgtcgccttgcgtataatatttgcccatggtgaaaacgggggcgaagaagttgtccatattggccacgtttaaatcaaaactggtgaaactcacccagggattggctgagacgaaaaacatattctcaataaaccctttagggaaataggccaggttttcaccgtaacacgccacatcttgcgaatatatgtgtagaaactgccggaaatcgtcgtggtattcactccagagcgatgaaaacgtttcagtttgctcatggaaaacggtgtaacaagggtgaacactatcccatatcaccagctcaccgtctttcattgccatacggaattccggatgagcattcatcaggcgggcaagaatgtgaataaaggccggataaaacttgtgcttatttttctttacggtctttaaaaaggccgtaatatccagctgaacggtctggttataggtacattgagcaactgactgaaatgcctcaaaatgttctttacgatgccattgggatatatcaacggtggtatatccagtgatttttttctccattttagcttccttagctcctgaaaatctcgataactcaaaaaatacgcccggtagtgatcttatttcattatggtgaaagttggaacctcttacgtgccgatcaacgtctcattttcgccaaaagttggcccagggcttcccggtatcaacagggacaccaggatttatttattctgcgaagtgatcttccgtcacaggtatttattcggcgcaaagtgcgtcgggtgatgctgccaacttagtcgactacaggtcactaataccatctaagtagttgattcatagtgactggatatgttgtgttttacagtattatgtagtctgttttttatgcaaaatctaatttaatatattgatatttatatcattttacgtttctcgttcaacttttctatacaaagttgccccgacctgcaggcatgcaagctttaatgcggtagtttatcacagttaaattgctaacgcagtcaggcaccgtgtatgaaatctaacaatgcgctcatcgtcatcctcggcaccgtcaccctggatgctgtaggcataggcttggttatgccggtactgccgggcctcttgcggggaattctaccgggtaggggaggcgcttttcccaaggcagtctggagcatgcgctttagcagccccgctgggcacttggcgctacacaagtggcctctggcctcgcacacattccacatccaccggtaggcgccaaccggctccgttctttggtggccccttcgcgccaccttctactcctcccctagtcaggaagttcccccccgccccgcagctcgcgtcgtgcaggacgtgacaaatggaagtagcacgtctcactagtctcgtgcagatggacagcaccgctgagcaatggaagcgggtaggcctttggggcagcggccaatagcagctttgctccttcgctttctgggctcagaggctgggaaggggtgggtccgggggcgggctcaggggcgggctcaggggcggggcgggcgcccgaaggtcctccggaggcccggcattctgcacgcttcaaaagcgcacgtctgccgcgctgttctcctcttcctcatctccgggcctttcgaccgatcatcaagcttgatcctcatgaaaaagcctgaactcaccgcgacgtctgtcgagaagtttctgatcgaaaagttcgacagcgtctccgacctgatgcagctctcggagggcgaagaatctcgtgctttcagcttcgatgtaggagggcGtggatatgtcctgcgggtaaatagctgcgccgatggtttctacaaagatcgttatgtttatcggcactttgcatcggccgcgctcccgattccggaagtgcttgacattggggaattcagcgagagcctgacctattgcatctcccgccgtgcacagggtgtcacgttgcaagacctgcctgaaaccgaactgcccgctgttctgcagccggtcgcggaggccatggatgcgatcgctgcggccgatcttagccagacgagcgggttcggcccattcggaccgcaaggaatcggtcaatacactacatggcgtgatttcatatgcgcgattgctgatccccatgtgtatcactggcaaactgtgatggacgacaccgtcagtgcgtccgtcgcgcaggctctcgatgagctgatgctttgggccgaggactgccccgaagtccggcacctcgtgcacgcggatttcggctccaacaatgtcctgacggacaatggccgcataacagcggtcattgactggagcgaggcgatgttcggggattcccaatacgaggtcgccaacatcttcttctggaggccgtggttggcttgtatggagcagcagacgcgctacttcgagcggaggcatccggagcttgcaggatcgccgcggctccgggcgtatatgctccGcattggtcttgaccaactctatcagagcttggttgacggcaatttcgatgatgcagcttgggcgcagggtcgatgcgacgcaatcgtccgatccggagccgggactgtcgggcgtacacaaatcgcccgcagaagcgcggccgtctggaccgatggctgtgtagaagtcgcgtctgcgttcgaccaggctgcgcgttctcgcggccatagcaaccgacgtacggcgttgcgccctcgccggcagcaagaagccacggaagtccgcccggagcagaaaatgcccacgctactgcgggtttatatagacggtccccacgggatggggaaaaccaccaccacgcaactgctggtggccctgggttcgcgcgacgatatcgtctacgtacccgagccgatgacttactggcgggtgctgggggcttccgagacaatcgcgaacatctacaccacacaacaccgcctcgaccagggtgagatatcggccggggacgcggcggtggtaatgacaagcgcccagataacaatgggcatgccttatgccgtgaccgacgccgttctggctcctcatatcgggggggaggctgggagctcacatgccccgcccccggccctcaccctcatcttcgaccgccatcccatcgccgccctcctgtgctacccggccgcgcggtaccttatgggcagcatgaccccccaggccgtgctggcgttcgtggccctcatcccgccgaccttgcccggcaccaacatcgtgcttggggcccttccggaggacagacacatcgaccgcctggccaaacgccagcgccccggcgagcggctggacctggctatgctggctgcgattcgccgcgtttacgggctacttgccaatacggtgcggtatctgcagtgcggcgggtcgtggcgggaggactggggacagctttcggggacggccgtgccgccccagggtgccgagccccagagcaacgcgggcccacgaccccatatcggggacacgttatttaccctgtttcgggcccccgagttgctggcccccaacggcgacctgtataacgtgtttgcctgggccttggacgtcttggccaaacgcctccgttccatgcacgtctttatcctggattacgaccaatcgcccgccggctgccgggacgccctgctgcaacttacctccgggatggtccagacccacgtcaccacccccggctccataccgacgatatgcgacctggcgcgcacgtttgcccgggagatgggggaggctaactgagctctagcttatcgactcgccgatagtggaaaccgacgccccagcactcgtccgagggcaaaggaatagtcgagaaattgatgatctattaaacaataaagatgtccacatggaagtttttcctgtcatactttgttaagaagggtgagaacagagtacctacattttgaatggaaggattggagctacgggggtgggggtggggtgggattagataaatgcctgctctttactgaaggctctttactattgctttatgataatgtttcatagttggatatcataatttaaacaagcaaaaccaaattaagggccagctcattcctcccactcatgatctatagatccatcagatcctttgttactttatagaagaaattttgagtttttgtttttttttaataaataaataaacataaataaattgtttgttgaatttattattagtatgtaagtgtaaatataataaaacttaatatctattcaaattaataaataaacctcgatatacagaccgataaaacacatgcgtcaattttacgcatgattatctttaacgtacgtcacaatatgattatctttctagggttaaagagctccagcttttgttccctttagtgagggttaatttcgagcttggcgtaatcatggtcatagctgtttcctgtgtgaaattgttatccgctcacaattccacacaacatacgagccggaagcataaagtgtaaagcctggggtgcctaatgagtgagctaactcacattaattgcgttgcgctcactgcccgctttccagtcgggaaacctgtcgtgccagctgcattaatgaatcggccaacgcgcggggagaggcggtttgcgtattgggcgctcttccgcttcctcgctcactgactcgctgcgctcggtcgttcggctgcggcgagcggtatcagctcactcaaaggcggtaatacggttatccacagaatcaggggataacgcaggaaagaacatgtgagcaaaaggccagcaaaaggccaggaaccgtaaaaaggccgcgttgctggcgtttttccataggctccgcccccctgacgagcatcacaaaaatcgacgctcaagtcagaggtggcgaaacccgacaggactataaagataccaggcgtttccccctggaagctccctcgtgcgctctcctgttccgaccctgccgcttaccggatacctgtccgcctttctcccttcgggaagcgtggcgctttctcatagctcacgctgtaggtatctcagttcggtgtaggtcgttcgctccaagctgggctgtgtgcacgaaccccccgttcagcccgaccgctgcgccttatccggtaactatcgtcttgagtccaacccggtaagacacgacttatcgccactggcagcagccactggtaacaggattagcagagcgaggtatgtaggcggtgctacagagttcttgaagtggtggcctaactacggctacactagaaggacagtatttggtatctgcgctctgctgaagccagttaccttcggaaaaagagttggtagctcttgatccggcaaacaaaccaccgctggtagcggtggtttttttgtttgcaagcagcagattacgcgcagaaaaaaaggatctcaagaagatcctttgatcttttctacggggtctgacgctcagtggaacgaaaactcacgttaagggattttggtcatgagattatcaaaaaggatcttcacctagatccttttaaattaaaaatgaagttttaaatcaatctaaagtatatatgagtaaacttggtctgacagttaccaatgcttaatcagtgaggcacctatctcagcgatctgtctatttcgttcatccatagttgcctgactccccgtcgtgtagataactacgatacgggagggcttaccatctggccccagtgctgcaatgataccgcgagacccacgctcaccggctccagatttatcagcaataaaccagccagccggaagggccgagcgcagaagtggtcctgcaactttatccgcctccatccagtctattaattgttgccgggaagctagagtaagtagttcgccagttaatagtttgcgcaacgttgttgccattgctacaggcatcgtggtgtcacgctcgtcgtttggtatggcttcattcagctccggttcccaacgatcaaggcgagttacatgatcccccatgttgtgcaaaaaagcggttagctccttcggtcctccgatcgttgtcagaagtaagttggccgcagtgttatcactcatggttatggcagcactgcataattctcttactgtcatgccatccgtaagatgcttttctgtgactggtgagtactcaaccaagtcattctgagaatagtgtatgcggcgaccgagttgctcttgcccggcgtcaatacgggataataccgcgccacatagcagaactttaaaagtgctcatcattggaaaacgttcttcggggcgaaaactctcaaggatcttaccgctgttgagatccagttcgatgtaacccactcgtgcacccaactgatcttcagcatcttttactttcaccagcgtttctgggtgagcaaaaacaggaaggcaaaatgccgcaaaaaagggaataagggcgacacggaaatgttgaatactcatactcttcctttttcaatattattgaagcatttatcagggttattgtctcatgagcggatacatatttgaatgtatttagaaaaataaacaaataggggttccgcgcacatttccccgaaaagtgccac

pPB-DEST-R5R4-pPuroTK

ctaaattgtaagcgttaatattttgttaaaattcgcgttaaatttttgttaaatcagctcattttttaaccaataggccgaaatcggcaaaatcccttataaatcaaaagaatagaccgagatagggttgagtgttgttccagtttggaacaagagtccactattaaagaacgtggactccaacgtcaaagggcgaaaaaccgtctatcagggcgatggcccactacgtgaaccatcaccctaatcaagttttttggggtcgaggtgccgtaaagcactaaatcggaaccctaaagggagcccccgatttagagcttgacggggaaagccggcgaacgtggcgagaaaggaagggaagaaagcgaaaggagcgggcgctagggcgctggcaagtgtagcggtcacgctgcgcgtaaccaccacacccgccgcgcttaatgcgccgctacagggcgcgtcccattcgccattcaggctgcgcaactgttgggaagggcgatcggtgcgggcctcttcgctattacgccagctggcgaaagggggatgtgctgcaaggcgattaagttgggtaacgccagggttttcccagtcacgacgttgtaaaacgacggccagtgaattgtaatacgactcactatagggcgaattgggtaccttaaccctagaaagatagtctgcgtaaaattgacgcatgcattcttgaaatattgctctctctttctaaatagcgcgaatccgtcgctgtgcatttaggacatctcagtcgccgcttggagctcccgtgaggcgtgcttgtcaatgcggtaagtgtcactgattttgaactataacgaccgcgtgagtcaaaatgacgcatgattatcttttacgtgacttttaagatttaactcatacgataattatattgttatttcatgttctacttacgtgataacttattatatatatattttcttgttatagatgatccactagtaacggccgccagtgtgctggaattcgcggccgctagagtcggggcaactttgtatacaaaagttgaacgagaaacgtaaaatgatataaatatcaatatattaaattagattttgcataaaaaacagactacataatactgtaaaacacaacatatccagtcactatgaatcaactacttagatggtattagtgacctgtagtcgaccgacagccttccaaatgttcttcgggtgatgctgccaacttagtcgaccgacagccttccaaatgttcttctcaaacggaatcgtcgtatccagcctactcgctattgtcctcaatgccgtattaaatcataaaaagaaataagaaaaagaggtgcgagcctcttttttgtgtgacaaaataaaaacatctacctattcatatacgctagtgtcatagtcctgaaaatcatctgcatcaagaacaatttcacaactcttatacttttctcttacaagtcgttcggcttcatctggattttcagcctctatacttactaaacgtgataaagtttctgtaatttctactgtatcgacctgcagactggctgtgtataagggagcctgacatttatattccccagaacatcaggttaatggcgtttttgatgtcattttcgcggtggctgagatcagccacttcttccccgataacggagaccggcacactggccatatcggtggtcatcatgcgccagctttcatccccgatatgcaccaccgggtaaagttcacgggagactttatctgacagcagacgtgcactggccagggggatcaccatccgtcgcccgggcgtgtcaataatatcactctgtacatccacaaacagacgataacggctctctcttttataggtgtaaaccttaaactgcatttcaccagtccctgttctcgtcagcaaaagagccgttcatttcaataaaccgggcgacctcagccatcccttcctgattttccgctttccagcgttcggcacgcagacgacgggcttcattctgcatggttgtgcttaccagaccggagatattgacatcatatatgccttgagcaactgatagctgtcgctgtcaactgtcactgtaatacgctgcttcatagcacacctctttttgacatacttcgggtatacatatcagtatatattcttataccgcaaaaatcagcgcgcaaatacgcatactgttatctggcttttagtaagccggatccacgcgattacgccccgccctgccactcatcgcagtactgttgtaattcattaagcattctgccgacatggaagccatcacagacggcatgatgaacctgaatcgccagcggcatcagcaccttgtcgccttgcgtataatatttgcccatggtgaaaacgggggcgaagaagttgtccatattggccacgtttaaatcaaaactggtgaaactcacccagggattggctgagacgaaaaacatattctcaataaaccctttagggaaataggccaggttttcaccgtaacacgccacatcttgcgaatatatgtgtagaaactgccggaaatcgtcgtggtattcactccagagcgatgaaaacgtttcagtttgctcatggaaaacggtgtaacaagggtgaacactatcccatatcaccagctcaccgtctttcattgccatacggaattccggatgagcattcatcaggcgggcaagaatgtgaataaaggccggataaaacttgtgcttatttttctttacggtctttaaaaaggccgtaatatccagctgaacggtctggttataggtacattgagcaactgactgaaatgcctcaaaatgttctttacgatgccattgggatatatcaacggtggtatatccagtgatttttttctccattttagcttccttagctcctgaaaatctcgataactcaaaaaatacgcccggtagtgatcttatttcattatggtgaaagttggaacctcttacgtgccgatcaacgtctcattttcgccaaaagttggcccagggcttcccggtatcaacagggacaccaggatttatttattctgcgaagtgatcttccgtcacaggtatttattcggcgcaaagtgcgtcgggtgatgctgccaacttagtcgactacaggtcactaataccatctaagtagttgattcatagtgactggatatgttgtgttttacagtattatgtagtctgttttttatgcaaaatctaatttaatatattgatatttatatcattttacgtttctcgttcaacttttctatacaaagttgccccgacctgcaggcatgcaagctttaatgcggtagtttatcacagttaaattgctaacgcagtcaggcaccgtgtatgaaatctaacaatgcgctcatcgtcatcctcggcaccgtcaccctggatgctgtaggcataggcttggttatgccggtactgccgggcctcttgcggggaattctaccgggtaggggaggcgcttttcccaaggcagtctggagcatgcgctttagcagccccgctgggcacttggcgctacacaagtggcctctggcctcgcacacattccacatccaccggtaggcgccaaccggctccgttctttggtggccccttcgcgccaccttctactcctcccctagtcaggaagttcccccccgccccgcagctcgcgtcgtgcaggacgtgacaaatggaagtagcacgtctcactagtctcgtgcagatggacagcaccgctgagcaatggaagcgggtaggcctttggggcagcggccaatagcagctttgctccttcgctttctgggctcagaggctgggaaggggtgggtccgggggcgggctcaggggcgggctcaggggcggggcgggcgcccgaaggtcctccggaggcccggcattctgcacgcttcaaaagcgcacgtctgccgcgctgttctcctcttcctcatctccgggcctttcgaccgatcatcaagcttgatcctcatgaccgagtacaagcccacggtgcgcctcgccacccgcgacgacgtccccagggccgtacgcaccctcgccgccgcgttcgccgactaccccgccacgcgccacaccgtcgatccggaccgccacatcgagcgggtcaccgagctgcaagaactcttcctcacgcgcgtcgggctcgacatcggcaaggtgtgggtcgcggacgacggcgccgcggtggcggtctggaccacgccggagagcgtcgaagcgggggcggtgttcgccgagatcggcccgcgcatggccgagttgagcggttcccggctggccgcgcagcaacagatggaaggcctcctggcgccgcaccggcccaaggagcccgcgtggttcctggccaccgtcggcgtctcgcccgaccaccagggcaagggtctgggcagcgccgtcgtgctccccggagtggaggcggccgagcgcgccggggtgcccgccttcctggagacctccgcgccccgcaacctccccttctacgagcggctcggcttcaccgtcaccgccgacgtcgaggtgcccgaaggaccgcgcacctggtgcatgacccgcaagcccggtgccggatccatgcccacgctactgcgggtttatatagacggtccccacgggatggggaaaaccaccaccacgcaactgctggtggccctgggttcgcgcgacgatatcgtctacgtacccgagccgatgacttactggcgggtgctgggggcttccgagacaatcgcgaacatctacaccacacaacaccgcctcgaccagggtgagatatcggccggggacgcggcggtggtaatgacaagcgcccagataacaatgggcatgccttatgccgtgaccgacgccgttctggctcctcatatcgggggggaggctgggagctcacatgccccgcccccggccctcaccctcatcttcgaccgccatcccatcgccgccctcctgtgctacccggccgcgcggtaccttatgggcagcatgaccccccaggccgtgctggcgttcgtggccctcatcccgccgaccttgcccggcaccaacatcgtgcttggggcccttccggaggacagacacatcgaccgcctggccaaacgccagcgccccggcgagcggctggacctggctatgctggctgcgattcgccgcgtttacgggctacttgccaatacggtgcggtatctgcagtgcggcgggtcgtggcgggaggactggggacagctttcggggacggccgtgccgccccagggtgccgagccccagagcaacgcgggcccacgaccccatatcggggacacgttatttaccctgtttcgggcccccgagttgctggcccccaacggcgacctgtataacgtgtttgcctgggccttggacgtcttggccaaacgcctccgttccatgcacgtctttatcctggattacgaccaatcgcccgccggctgccgggacgccctgctgcaacttacctccgggatggtccagacccacgtcaccacccccggctccataccgacgatatgcgacctggcgcgcacgtttgcccgggagatgggggaggctaactgagctctagcttatcgactcgccgatagtggaaaccgacgccccagcactcgtccgagggcaaaggaatagtcgagaaattgatgatctattaaacaataaagatgtccacatggaagtttttcctgtcatactttgttaagaagggtgagaacagagtacctacattttgaatggaaggattggagctacgggggtgggggtggggtgggattagataaatgcctgctctttactgaaggctctttactattgctttatgataatgtttcatagttggatatcataatttaaacaagcaaaaccaaattaagggccagctcattcctcccactcatgatctatagatccatcagatcctttgttactttatagaagaaattttgagtttttgtttttttttaataaataaataaacataaataaattgtttgttgaatttattattagtatgtaagtgtaaatataataaaacttaatatctattcaaattaataaataaacctcgatatacagaccgataaaacacatgcgtcaattttacgcatgattatctttaacgtacgtcacaatatgattatctttctagggttaaagagctccagcttttgttccctttagtgagggttaatttcgagcttggcgtaatcatggtcatagctgtttcctgtgtgaaattgttatccgctcacaattccacacaacatacgagccggaagcataaagtgtaaagcctggggtgcctaatgagtgagctaactcacattaattgcgttgcgctcactgcccgctttccagtcgggaaacctgtcgtgccagctgcattaatgaatcggccaacgcgcggggagaggcggtttgcgtattgggcgctcttccgcttcctcgctcactgactcgctgcgctcggtcgttcggctgcggcgagcggtatcagctcactcaaaggcggtaatacggttatccacagaatcaggggataacgcaggaaagaacatgtgagcaaaaggccagcaaaaggccaggaaccgtaaaaaggccgcgttgctggcgtttttccataggctccgcccccctgacgagcatcacaaaaatcgacgctcaagtcagaggtggcgaaacccgacaggactataaagataccaggcgtttccccctggaagctccctcgtgcgctctcctgttccgaccctgccgcttaccggatacctgtccgcctttctcccttcgggaagcgtggcgctttctcatagctcacgctgtaggtatctcagttcggtgtaggtcgttcgctccaagctgggctgtgtgcacgaaccccccgttcagcccgaccgctgcgccttatccggtaactatcgtcttgagtccaacccggtaagacacgacttatcgccactggcagcagccactggtaacaggattagcagagcgaggtatgtaggcggtgctacagagttcttgaagtggtggcctaactacggctacactagaaggacagtatttggtatctgcgctctgctgaagccagttaccttcggaaaaagagttggtagctcttgatccggcaaacaaaccaccgctggtagcggtggtttttttgtttgcaagcagcagattacgcgcagaaaaaaaggatctcaagaagatcctttgatcttttctacggggtctgacgctcagtggaacgaaaactcacgttaagggattttggtcatgagattatcaaaaaggatcttcacctagatccttttaaattaaaaatgaagttttaaatcaatctaaagtatatatgagtaaacttggtctgacagttaccaatgcttaatcagtgaggcacctatctcagcgatctgtctatttcgttcatccatagttgcctgactccccgtcgtgtagataactacgatacgggagggcttaccatctggccccagtgctgcaatgataccgcgagacccacgctcaccggctccagatttatcagcaataaaccagccagccggaagggccgagcgcagaagtggtcctgcaactttatccgcctccatccagtctattaattgttgccgggaagctagagtaagtagttcgccagttaatagtttgcgcaacgttgttgccattgctacaggcatcgtggtgtcacgctcgtcgtttggtatggcttcattcagctccggttcccaacgatcaaggcgagttacatgatcccccatgttgtgcaaaaaagcggttagctccttcggtcctccgatcgttgtcagaagtaagttggccgcagtgttatcactcatggttatggcagcactgcataattctcttactgtcatgccatccgtaagatgcttttctgtgactggtgagtactcaaccaagtcattctgagaatagtgtatgcggcgaccgagttgctcttgcccggcgtcaatacgggataataccgcgccacatagcagaactttaaaagtgctcatcattggaaaacgttcttcggggcgaaaactctcaaggatcttaccgctgttgagatccagttcgatgtaacccactcgtgcacccaactgatcttcagcatcttttactttcaccagcgtttctgggtgagcaaaaacaggaaggcaaaatgccgcaaaaaagggaataagggcgacacggaaatgttgaatactcatactcttcctttttcaatattattgaagcatttatcagggttattgtctcatgagcggatacatatttgaatgtatttagaaaaataaacaaataggggttccgcgcacatttccccgaaaagtgccac

pENTR-R2L4

tctatacaaagttggcattataagaaagcattgcttatcaatttgttgcaacgaacaggtcactatcagtcaaaataaaatcattatttgccatccagctgcagctctggcccgtgtctcaaaatctctgatgttacattgcacaagataaaaatatatcatcatgaacaataaaactgtctgcttacataaacagtaatacaaggggtgttatgagccatattcaacgggaaacgtcgaggccgcgattaaattccaacatggatgctgatttatatgggtataaatgggctcgcgataatgtcgggcaatcaggtgcgacaatctatcgcttgtatgggaagcccgatgcgccagagttgtttctgaaacatggcaaaggtagcgttgccaatgatgttacagatgagatggtcagactaaactggctgacggaatttatgcctcttccgaccatcaagcattttatccgtactcctgatgatgcatggttactcaccactgcgatccccggaaaaacagcattccaggtattagaagaatatcctgattcaggtgaaaatattgttgatgcgctggcagtgttcctgcgccggttgcattcgattcctgtttgtaattgtccttttaacagcgatcgcgtatttcgtctcgctcaggcgcaatcacgaatgaataacggtttggttgatgcgagtgattttgatgacgagcgtaatggctggcctgttgaacaagtctggaaagaaatgcataaacttttgccattctcaccggattcagtcgtcactcatggtgatttctcacttgataaccttatttttgacgaggggaaattaataggttgtattgatgttggacgagtcggaatcgcagaccgataccaggatcttgccatcctatggaactgcctcggtgagttttctccttcattacagaaacggctttttcaaaaatatggtattgataatcctgatatgaataaattgcagtttcatttgatgctcgatgagtttttctaatcagaattggttaattggttgtaacactggcagagcattacgctgacttgacgggacggcgcaagctcatgaccaaaatcccttaacgtgagttttcgttccactgagcgtcagaccccgtagaaaagatcaaaggatcttcttgagatcctttttttctgcgcgtaatctgctgcttgcaaacaaaaaaaccaccgctaccagcggtggtttgtttgccggatcaagagctaccaactctttttccgaaggtaactggcttcagcagagcgcagataccaaatactgtccttctagtgtagccgtagttaggccaccacttcaagaactctgtagcaccgcctacatacctcgctctgctaatcctgttaccagtggctgctgccagtggcgataagtcgtgtcttaccgggttggactcaagacgatagttaccggataaggcgcagcggtcgggctgaacggggggttcgtgcacacagcccagcttggagcgaacgacctacaccgaactgagatacctacagcgtgagctatgagaaagcgccacgcttcccgaagggagaaaggcggacaggtatccggtaagcggcagggtcggaacaggagagcgcacgagggagcttccagggggaaacgcctggtatctttatagtcctgtcgggtttcgccacctctgacttgagcgtcgatttttgtgatgctcgtcaggggggcggagcctatggaaaaacgccagcaacgcggcctttttacggttcctggccttttgctggccttttgctcacatgttctttcctgcgttatcccctgattctgtggataaccgtattaccgctagccaggaagagtttgtagaaacgcaaaaaggccatccgtcaggatggccttctgcttagtttgatgcctggcagtttatggcgggcgtcctgcccgccaccctccgggccgttgcttcacaacgttcaaatccgctcccggcggatttgtcctactcaggagagcgttcaccgacaaacaacagataaaacgaaaggcccagtcttccgactgagcctttcgttttatttgatgcctggcagttccctactctcgcgttaacgctagcatggatctcgggccctgcagctctagagctcgaattctacaggtcactaataccatctaagtagttggttcatagtgactgcatatgttgtgttttacagtattatgtagtctgttttttatgcaaaatctaatttaatatattgatatttatatcattttacgtttctcgttcaactttcttgtacaaagtggtctcgagttaacaacttt

pENTR-R2-pA-L4

tcgagttaacaacttttctatacaaagttggcattataagaaagcattgcttatcaatttgttgcaacgaacaggtcactatcagtcaaaataaaatcattatttgccatccagctgcagctctggcccgtgtctcaaaatctctgatgttacattgcacaagataaaaatatatcatcatgaacaataaaactgtctgcttacataaacagtaatacaaggggtgttatgagccatattcaacgggaaacgtcgaggccgcgattaaattccaacatggatgctgatttatatgggtataaatgggctcgcgataatgtcgggcaatcaggtgcgacaatctatcgcttgtatgggaagcccgatgcgccagagttgtttctgaaacatggcaaaggtagcgttgccaatgatgttacagatgagatggtcagactaaactggctgacggaatttatgcctcttccgaccatcaagcattttatccgtactcctgatgatgcatggttactcaccactgcgatccccggaaaaacagcattccaggtattagaagaatatcctgattcaggtgaaaatattgttgatgcgctggcagtgttcctgcgccggttgcattcgattcctgtttgtaattgtccttttaacagcgatcgcgtatttcgtctcgctcaggcgcaatcacgaatgaataacggtttggttgatgcgagtgattttgatgacgagcgtaatggctggcctgttgaacaagtctggaaagaaatgcataaacttttgccattctcaccggattcagtcgtcactcatggtgatttctcacttgataaccttatttttgacgaggggaaattaataggttgtattgatgttggacgagtcggaatcgcagaccgataccaggatcttgccatcctatggaactgcctcggtgagttttctccttcattacagaaacggctttttcaaaaatatggtattgataatcctgatatgaataaattgcagtttcatttgatgctcgatgagtttttctaatcagaattggttaattggttgtaacactggcagagcattacgctgacttgacgggacggcgcaagctcatgaccaaaatcccttaacgtgagttttcgttccactgagcgtcagaccccgtagaaaagatcaaaggatcttcttgagatcctttttttctgcgcgtaatctgctgcttgcaaacaaaaaaaccaccgctaccagcggtggtttgtttgccggatcaagagctaccaactctttttccgaaggtaactggcttcagcagagcgcagataccaaatactgtccttctagtgtagccgtagttaggccaccacttcaagaactctgtagcaccgcctacatacctcgctctgctaatcctgttaccagtggctgctgccagtggcgataagtcgtgtcttaccgggttggactcaagacgatagttaccggataaggcgcagcggtcgggctgaacggggggttcgtgcacacagcccagcttggagcgaacgacctacaccgaactgagatacctacagcgtgagcattgagaaagcgccacgcttcccgaagggagaaaggcggacaggtatccggtaagcggcagggtcggaacaggagagcgcacgagggagcttccagggggaaacgcctggtatctttatagtcctgtcgggtttcgccacctctgacttgagcgtcgatttttgtgatgctcgtcaggggggcggagcctatggaaaaacgccagcaacgcggcctttttacggttcctggccttttgctggccttttgctcacatgttctttcctgcgttatcccctgattctgtggataaccgtattaccgcctttgagtgagctgataccgctcgccgcagccgaacgaccgagcgcagcgagtcagtgagcgaggaagcggaagagcgcccaatacgcaaaccgcctctccccgcgcgttggccgattcattaatgcagctggcacgacaggtttcccgactggaaagcgggcagtgagcgcaacgcaattaatacgcgtaccgctagccaggaagagtttgtagaaacgcaaaaaggccatccgtcaggatggccttctgcttagtttgatgcctggcagtttatggcgggcgtcctgcccgccaccctccgggccgttgcttcacaacgttcaaatccgctcccggcggatttgtcctactcaggagagcgttcaccgacaaacaacagataaaacgaaaggcccagtcttccgactgagcctttcgttttatttgatgcctggcagttccctactctcgcgttaacgctagcatggatgttttcccagtcacgacgttgtaaaacgacggccagtcttaagctcgggccctgcagctctagagctcgaattctacaggtcactaataccatctaagtagttgattcatagtgactgcatatgttgtgttttacagtattatgtagtctgttttttatgcaaaatctaatttaatatattgatatttatatcattttacgtttctcgttcaactttcttgtacaaagtggtctagagaattcgtcgacagatctaacttgtttattgcagcttataatggttacaaataaagcaatagcatcacaaatttcacaaataaagcatttttttcactgcattctagttgtggtttgtccaaactcatcaatgtatcttatcatgtctggatcc

pENTR-R2-WPRE-pA-L4

cgcgttaacgctagcatggatgttttcccagtcacgacgttgtaaaacgacggccagtcttaagctcgggccctgcagctctagagctcgaattctacaggtcactaataccatctaagtagttgGttcatagtgactgcatatgttgtgttttacagtattatgtagtctgttttttatgcaaaatctaatttaatatattgatatttatatcattttacgtttctcgttcaactttcttgtacaaagtggtctagagaattcggtcatatgataatcaacctctggattacaaaatttgtgaaagattgactggtattcttaactatgttgctccttttacgctatgtggatacgctgctttaatgcctttgtatcatgctattgcttcccgtatggctttcattttctcctccttgtataaatcctggttgctgtctctttatgaggagttgtggcccgttgtcaggcaacgtggcgtggtgtgcactgtgtttgctgacgcaacccccactggttggggcattgccaccacctgtcagctcctttccgggactttcgctttccccctccctattgccacggcggaactcatcgccgcctgccttgcccgctgctggacaggggctcggctgttgggcactgacaattccgtggtgttgtcggggaagctgacgtcctttccatggctgctcgcctgtgttgccacctggattctgcgcgggacgtccttctgctacgtcccttcggccctcaatccagcggaccttccttcccgcggcctgctgccggctctgcggcctcttccgcgtcttcgccttcgccctcagacgagtcggatctccctttgggccgcctccccgcatcggtaaattcagatctaacttgtttattgcagcttataatggttacaaataaagcaatagcatcacaaatttcacaaataaagcatttttttcactgcattctagttgtggtttgtccaaactcatcaatgtatcttatcatgtctggatcctcgagttaacaacttttctatacaaagttggcattataagaaagcattgcttatcaatttgttgcaacgaacaggtcactatcagtcaaaataaaatcattatttgccatccagctgcagctctggcccgtgtctcaaaatctctgatgttacattgcacaagataaaaatatatcatcatgaacaataaaactgtctgcttacataaacagtaatacaaggggtgttatgagccatattcaacgggaaacgtcgaggccgcgattaaattccaacatggatgctgatttatatgggtataaatgggctcgcgataatgtcgggcaatcaggtgcgacaatctatcgcttgtatgggaagcccgatgcgccagagttgtttctgaaacatggcaaaggtagcgttgccaatgatgttacagatgagatggtcagactaaactggctgacggaatttatgcctcttccgaccatcaagcattttatccgtactcctgatgatgcatggttactcaccactgcgatccccggaaaaacagcattccaggtattagaagaatatcctgattcaggtgaaaatattgttgatgcgctggcagtgttcctgcgccggttgcattcgattcctgtttgtaattgtccttttaacagcgatcgcgtatttcgtctcgctcaggcgcaatcacgaatgaataacggtttggttgatgcgagtgattttgatgacgagcgtaatggctggcctgttgaacaagtctggaaagaaatgcataaacttttgccattctcaccggattcagtcgtcactcatggtgatttctcacttgataaccttatttttgacgaggggaaattaataggttgtattgatgttggacgagtcggaatcgcagaccgataccaggatcttgccatcctatggaactgcctcggtgagttttctccttcattacagaaacggctttttcaaaaatatggtattgataatcctgatatgaataaattgcagtttcatttgatgctcgatgagtttttctaatcagaattggttaattggttgtaacactggcagagcattacgctgacttgacgggacggcgcaagctcatgaccaaaatcccttaacgtgagttttcgttccactgagcgtcagaccccgtagaaaagatcaaaggatcttcttgagatcctttttttctgcgcgtaatctgctgcttgcaaacaaaaaaaccaccgctaccagcggtggtttgtttgccggatcaagagctaccaactctttttccgaaggtaactggcttcagcagagcgcagataccaaatactgtccttctagtgtagccgtagttaggccaccacttcaagaactctgtagcaccgcctacatacctcgctctgctaatcctgttaccagtggctgctgccagtggcgataagtcgtgtcttaccgggttggactcaagacgatagttaccggataaggcgcagcggtcgggctgaacggggggttcgtgcacacagcccagcttggagcgaacgacctacaccgaactgagatacctacagcgtgagcattgagaaagcgccacgcttcccgaagggagaaaggcggacaggtatccggtaagcggcagggtcggaacaggagagcgcacgagggagcttccagggggaaacgcctggtatctttatagtcctgtcgggtttcgccacctctgacttgagcgtcgatttttgtgatgctcgtcaggggggcggagcctatggaaaaacgccagcaacgcggcctttttacggttcctggccttttgctggccttttgctcacatgttctttcctgcgttatcccctgattctgtggataaccgtattaccgcctttgagtgagctgataccgctcgccgcagccgaacgaccgagcgcagcgagtcagtgagcgaggaagcggaagagcgcccaatacgcaaaccgcctctccccgcgcgttggccgattcattaatgcagctggcacgacaggtttcccgactggaaagcgggcagtgagcgcaacgcaattaatacgcgtaccgctagccaggaagagtttgtagaaacgcaaaaaggccatccgtcaggatggccttctgcttagtttgatgcctggcagtttatggcgggcgtcctgcccgccaccctccgggccgttgcttcacaacgttcaaatccgctcccggcggatttgtcctactcaggagagcgttcaccgacaaacaacagataaaacgaaaggcccagtcttccgactgagcctttcgttttatttgatgcctggcagttccctactct

pDONR-P5P4

tcgaccgacagccttccaaatgttcttctcaaacggaatcgtcgtatccagcctactcgctattgtcctcaatgccgtattaaatcataaaaagaaataagaaaaagaggtgcgagcctcttttttgtgtgacaaaataaaaacatctacctattcatatacgctagtgtcatagtcctgaaaatcatctgcatcaagaacaatttcacaactcttatacttttctcttacaagtcgttcggcttcatctggattttcagcctctatacttactaaacgtgataaagtttctgtaatttctactgtatcgacctgcagactggctgtgtataagggagcctgacatttatattccccagaacatcaggttaatggcgtttttgatgtcattttcgcggtggctgagatcagccacttcttccccgataacggagaccggcacactggccatatcggtggtcatcatgcgccagctttcatccccgatatgcaccaccgggtaaagttcacgggagactttatctgacagcagacgtgcactggccagggggatcaccatccgtcgcccgggcgtgtcaataatatcactctgtacatccacaaacagacgataacggctctctcttttataggtgtaaaccttaaactgcatttcaccagtccctgttctcgtcagcaaaagagccgttcatttcaataaaccgggcgacctcagccatcccttcctgattttccgctttccagcgttcggcacgcagacgacgggcttcattctgcatggttgtgcttaccagaccggagatattgacatcatatatgccttgagcaactgatagctgtcgctgtcaactgtcactgtaatacgctgcttcatagcacacctctttttgacatacttcgggtatacatatcagtatatattcttataccgcaaaaatcagcgcgcaaatacgcatactgttatctggcttttagtaagccggatccacgcgattacgccccgccctgccactcatcgcagtactgttgtaattcattaagcattctgccgacatggaagccatcacagacggcatgatgaacctgaatcgccagcggcatcagcaccttgtcgccttgcgtataatatttgcccatggtgaaaacgggggcgaagaagttgtccatattggccacgtttaaatcaaaactggtgaaactcacccagggattggctgagacgaaaaacatattctcaataaaccctttagggaaataggccaggttttcaccgtaacacgccacatcttgcgaatatatgtgtagaaactgccggaaatcgtcgtggtattcactccagagcgatgaaaacgtttcagtttgctcatggaaaacggtgtaacaagggtgaacactatcccatatcaccagctcaccgtctttcattgccatacggaattccggatgagcattcatcaggcgggcaagaatgtgaataaaggccggataaaacttgtgcttatttttctttacggtctttaaaaaggccgtaatatccagctgaacggtctggttataggtacattgagcaactgactgaaatgcctcaaaatgttctttacgatgccattgggatatatcaacggtggtatatccagtgatttttttctccattttagcttccttagctcctgaaaatctcgataactcaaaaaatacgcccggtagtgatcttatttcattatggtgaaagttggaacctcttacgtgccgatcaacgtctcattttcgccaaaagttggcccagggcttcccggtatcaacagggacaccaggatttatttattctgcgaagtgatcttccgtcacaggtatttattcggcgcaaagtgcgtcgggtgatgctgccaacttagtcgactacaggtcactaataccatctaagtagttgattcatagtgactggatatgttgtgttttacagtattatgtagtctgttttttatgcaaaatctaatttaatatattgatatttatatcattttacgtttctcgttcaacttttctatacaaagttggcattataagaaagcattgcttatcaatttgttgcaacgaacaggtcactatcagtcaaaataaaatcattatttgccatccagctgcagctctggcccgtgtctcaaaatctctgatgttacattgcacaagataaaaatatatcatcatgaacaataaaactgtctgcttacataaacagtaatacaaggggtgttatgagccatattcaacgggaaacgtcgaggccgcgattaaattccaacatggatgctgatttatatgggtataaatgggctcgcgataatgtcgggcaatcaggtgcgacaatctatcgcttgtatgggaagcccgatgcgccagagttgtttctgaaacatggcaaaggtagcgttgccaatgatgttacagatgagatggtcagactaaactggctgacggaatttatgcctcttccgaccatcaagcattttatccgtactcctgatgatgcatggttactcaccactgcgatccccggaaaaacagcattccaggtattagaagaatatcctgattcaggtgaaaatattgttgatgcgctggcagtgttcctgcgccggttgcattcgattcctgtttgtaattgtccttttaacagcgatcgcgtatttcgtctcgctcaggcgcaatcacgaatgaataacggtttggttgatgcgagtgattttgatgacgagcgtaatggctggcctgttgaacaagtctggaaagaaatgcataaacttttgccattctcaccggattcagtcgtcactcatggtgatttctcacttgataaccttatttttgacgaggggaaattaataggttgtattgatgttggacgagtcggaatcgcagaccgataccaggatcttgccatcctatggaactgcctcggtgagttttctccttcattacagaaacggctttttcaaaaatatggtattgataatcctgatatgaataaattgcagtttcatttgatgctcgatgagtttttctaatcagaattggttaattggttgtaacactggcagagcattacgctgacttgacgggacggcgcaagctcatgaccaaaatcccttaacgtgagttttcgttccactgagcgtcagaccccgtagaaaagatcaaaggatcttcttgagatcctttttttctgcgcgtaatctgctgcttgcaaacaaaaaaaccaccgctaccagcggtggtttgtttgccggatcaagagctaccaactctttttccgaaggtaactggcttcagcagagcgcagataccaaatactgtccttctagtgtagccgtagttaggccaccacttcaagaactctgtagcaccgcctacatacctcgctctgctaatcctgttaccagtggctgctgccagtggcgataagtcgtgtcttaccgggttggactcaagacgatagttaccggataaggcgcagcggtcgggctgaacggggggttcgtgcacacagcccagcttggagcgaacgacctacaccgaactgagatacctacagcgtgagctatgagaaagcgccacgcttcccgaagggagaaaggcggacaggtatccggtaagcggcagggtcggaacaggagagcgcacgagggagcttccagggggaaacgcctggtatctttatagtcctgtcgggtttcgccacctctgacttgagcgtcgatttttgtgatgctcgtcaggggggcggagcctatggaaaaacgccagcaacgcggcctttttacggttcctggccttttgctggccttttgctcacatgttctttcctgcgttatcccctgattctgtggataaccgtattaccgctagccaggaagagtttgtagaaacgcaaaaaggccatccgtcaggatggccttctgcttagtttgatgcctggcagtttatggcgggcgtcctgcccgccaccctccgggccgttgcttcacaacgttcaaatccgctcccggcggatttgtcctactcaggagagcgttcaccgacaaacaacagataaaacgaaaggcccagtcttccgactgagcctttcgttttatttgatgcctggcagttccctactctcgcgttaacgctagcatggatctcgggccccaaataatgattttattttgactgatagtgacctgttcgttgcaacaaattgatgagcaatgcttttttataatgccaactttgtatacaaaagttgaacgagaaacgtaaaatgatataaatatcaatatattaaattagattttgcataaaaaacagactacataatactgtaaaacacaacatatccagtcactatgaatcaactacttagatggtattagtgacctgtag
